# Micronutrient Deficiencies Associated with a Gluten-Free Diet in Patients with Celiac Disease and Non-Celiac Gluten or Wheat Sensitivity: A Systematic Review and Meta-Analysis

**DOI:** 10.3390/jcm14144848

**Published:** 2025-07-08

**Authors:** Lindsey A. Russell, Paige Alliston, David Armstrong, Elena F. Verdu, Paul Moayyedi, Maria Ines Pinto-Sanchez

**Affiliations:** 1 Farncombe Family Digestive Health Research Institute, Hamilton, ON L8S 4L8, Canada; russell5@ccf.org (L.A.R.); armstro@mcmaster.ca (D.A.); verdue@mcmaster.ca (E.F.V.); moayyep@mcmaster.ca (P.M.); 2 Division of Gastroenterology, McMaster University, Hamilton, ON L8S 4L8, Canada; 3 Digestive Disease & Surgery Institute, Cleveland Clinic, Cleveland, OH 44195, USA; 4 Faculty of Health Sciences, School of Nursing, McMaster University, Hamilton, ON L8S 4L8, Canada; allistop@mcmaster.ca

**Keywords:** wheat-related diseases, celiac disease, micronutrient deficiency, gluten-free diet

## Abstract

**Background:** A gluten-free diet (GFD) has been shown to be nutritionally inadequate for those with wheat-related disorders. However, the differences in findings and the absence of quantitative analysis limits the interpretation of previous reviews. **Objectives:** We conducted a systematic review and meta-analysis to identify the risk of micronutrient deficiencies in patients with celiac disease (CeD) and non-celiac gluten or wheat sensitivity (NCWS). **Methods:** We searched the Cochrane Central Register of Controlled Trials, MEDLINE, EMBASE, and Web of Science (Ovid) databases. The risk of bias was determined using the ROBINS-1, and the quality of evidence was assessed using the GRADE approach. **Results** We identified 7940 studies; 46 observational studies (11 cohort, 9 cross-sectional, and 26 case–control) were eligible for analysis. CeD patients had an increased risk of vitamin D and E deficiencies compared with the non-CeD controls. CeD on a GFD had a decreased risk of vitamin D, B12, E, calcium, and iron deficiencies compared with untreated CeD. NCWS had an increased risk of vitamin B12, folate, and iron deficiency compared to the controls. The overall quality of evidence was rated very low. **Conclusions:** The risk of various micronutrient deficiencies is increased in CeD but is decreased for some after a GFD. Adequately powered studies with a rigorous methodology are needed to inform the risk of nutrient deficiencies in patients with CeD and NCWS. Protocol registration: Prospero-CRD42022313508.

## 1. Introduction

Wheat-related disorders (WRDs) include non-celiac wheat sensitivity (NCWS), celiac disease (CeD), and wheat allergy (WA) [1]. WRDs are common conditions, with a prevalence of CeD estimated to be ~1–2% of the worldwide population [2] and up to 6% for NCWS [3,4].

A strict gluten-free diet (GFD) is the only available treatment for people with CeD, an autoimmune condition triggered by gluten in genetically predisposed individuals [1,5]. Adopting a GFD has become more popular in people without CeD, and annual growth for gluten-free products in the US is projected to increase yearly [6].

A GFD can lead to the inadequate intake of macro- and micronutrients (vitamins and minerals) in those who may already have deficiencies due to malabsorption in active CeD [7]. Indeed, micronutrient deficiencies may persist in CeD patients on a GFD with documented resolution of villous atrophy, suggesting the nutritional inadequacy of a GFD [8]. In studies on children [7,9] and adults [10] with CeD and NCWS [11], adopting a GFD showed lower dietary intakes of various micronutrients, further contributing to the problem.

Prior systematic reviews have focused on the nutritional adequacy of a GFD in CeD [7,9,10]; however, the absence of quantitative analysis limits the interpretation of the results. There are no systematic reviews that investigate micronutrient deficiencies in non-CeD populations and the impact of the duration of and adherence to a GFD. Therefore, we conducted a systematic review and meta-analysis to identify the risk of micronutrient deficiencies in patients adopting a GFD for WRDs; however, we found that only studies on CeD met the predetermined criteria. We explored factors including the duration of a GFD, dietary adherence, and the presence of gastrointestinal or extraintestinal symptoms that might impact the risk of developing micronutrient deficiencies.

## 2. Methods

We conducted a systematic review and meta-analysis according to the Preferred Reporting Items for Systematic Reviews and Meta-Analyses Statement 2020 (PRISMA) guidance [12]. The relevant databases were searched, including the Cochrane Central Register of Controlled Trials (CENTRAL) (2000-), the Database of Abstracts of Reviews of Effectiveness (DARE) (1994-), MEDLINE (Ovid) (1946-), EMBASE (Ovid) (1974-), Web of Science (Ovid) (1900-), and CINAHL (1982-), and the gray literature (ex. conference reports, technical reports, and dissertations) was searched using SIGLE (1982-) up to April 2024. A registered Research Librarian at McMaster University was consulted to help develop an appropriate and inclusive search strategy. Conference abstracts were included, and a recursive bibliography search was conducted. Duplicates were detected manually and removed ahead of literature screening. Please refer to Supplementary Tables S1 and S2 for a detailed overview of the subject headings that were used and a sample search strategy.

We included studies evaluating micronutrients in adults or children (over 2 years) with CeD or NCWS. For CeD diagnosis, we used any well-defined criteria available (duodenal biopsy and/or serology-compatible and HLA DQ2/8-positive when available). Controls included CeD patients not on a GFD (untreated CeD) or non-celiac (non-CeD) controls (either populations without CeD or when CeD was excluded by either a duodenal biopsy or specific CeD serology). Micronutrients included vitamins A, B1-12, C, D, E, and K; folic acid or folate; zinc; copper; selenium; chromium; calcium; magnesium; and phosphorus.

Ferritin is a protein that stores iron and therefore was included as a surrogate marker for iron deficiency. NCWS was defined as a self-report of gastrointestinal or extraintestinal symptoms triggered by gluten-containing food, in the absence of CeD.

We considered the following outcomes: micronutrient deficiencies (proportion of study population with confirmed vitamin/mineral deficiency and specific levels), gastrointestinal (GI) symptoms (proportion of patients with diarrhea, abdominal pain, constipation, bloating), extraintestinal symptoms (skin, tiredness, anemia), and quality of life (any scale). The protocol was registered in Prospero (CRD42022313508).

### 2.1. Type of Studies

We included observational studies (cohort, cross-sectional, or case–control) and randomized, placebo-controlled trials (RCTs) until 4 April 2024. We considered cross-over studies only if results were available before the cross-over so the study could be evaluated as a parallel group. Publications were considered regardless of language and publication status. Abstracts were included only if we could obtain further details from the investigators. If information was missing from a study, the authors were contacted repeatedly to provide details. Studies were excluded if they were case reports or case series, had no control comparison group, duplicated publications, or had missing data to fulfil the inclusion criteria or comorbidities for diseases other than CeD (e.g., type 1 diabetes mellitus) that might have led to nutrient deficiencies. The search strategies and online databases searched are outlined in Supplementary Tables S1 and S2. Refer to Table 1 for the PICOS criteria.

### 2.2. Study Selection

Two authors (LR and PA) screened the titles and abstracts. After removing duplicates, abstract and full-text screening data were collected in Excel (Microsoft, Washington, DC, USA). The agreement was determined by discussions on the rationale between the two reviewers, and if discrepancies remained unresolved on the disagreements discussed, a third reviewer (MIPS) was consulted for the final decision. Both reviewers extracted data independently from the qualifying full texts. The data extraction form included the study design, funding sources (if applicable), location, patient demographics, CeD and NCWS diagnosis, control type (CeD not on a GFD or non-CeD), duration of GFD, levels of micronutrients, and specimen type (serum/blood or erythrocyte). The proportions of patients with micronutrient deficiency were recorded as mean ± SD, n/N, or % as applicable. For quantifiable data, units were standardized where applicable to ensure consistency across studies. If values were recorded in the median, minimum–maximum range, or 1st and 3rd quartiles, then they were converted to mean ± SD as previously described [13] (see equations in Supplementary Table S4). Data were entered into RevMan 5.4 [14] (The Cochrane Collaboration, Copenhagen) for further analysis.

### 2.3. Assessment of Risk of Bias for Included Studies

Both authors independently assessed the risk of bias for each study using the Cochrane Risk of Bias Tool [13] for RCTs and the ROBINS-I (Risk of Bias in Non-Randomised Studies of Interventions) for observational studies [15]. To explore the possibility of a risk of publication bias, a funnel plot and statistical tests for asymmetry were evaluated when more than 10 studies were included in a meta-analysis [16].

### 2.4. Measurement of Treatment Effect

Information regarding the number of participants who did or did not develop micronutrient deficiencies was reported as n/N. Quantitative analyses was performed with RevMan 5.414. Dichotomous outcomes were summarized using an odds ratio (OR) with associated 95% confidence intervals (CIs) [14] and continuous outcomes as mean differences (MDs) with 95% CIs. Data were pooled using a random effects model [13]. Statistically significant heterogeneity was determined by I^2^ (I^2^ > 25%) [13]. Subgroup analyses were performed by the study design, duration of the GFD (short-term < 2 years vs. long-term GFD ≥ 2 years) [13], symptomatic vs. asymptomatic populations, studies conducted in North America vs. other countries, and pediatric vs. adult populations where applicable. If the length of the GFD was not clear in a study, the authors were contacted and discussions between reviewers were had to determine which length of a GFD was appropriate; if the length of the GFD was unclear, it was considered to be a short-term GFD. The GRADE approach [17] was used to determine the quality of the evidence.

## 3. Results

**Figure 1:**
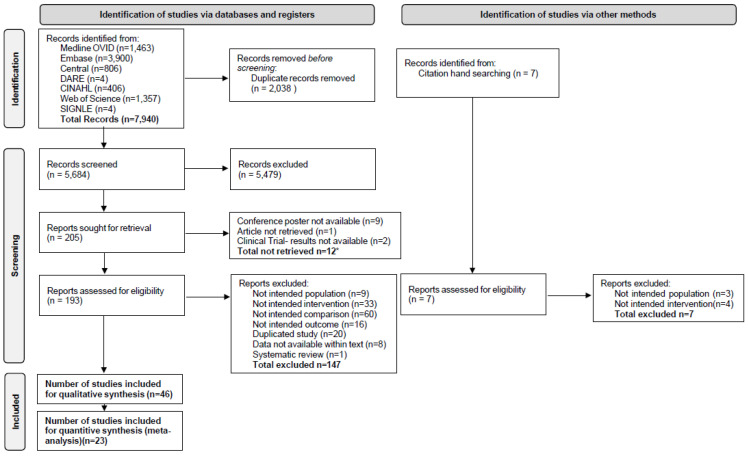
Flow chart of literature search process. *—attempted retrieval was not successful.

The literature search identified 7940 records, and 7 additional records were identified through a manual search of references. A total of 5684 records remained after removing duplicates, and 193 records were eligible for full-text screening (Figure 1). After a full-text review, 10 studies, mostly conference papers, were not retrieved despite contacting the authors (see Supplementary Table S4). A total of 147 papers was excluded; the specific reasons for exclusion are outlined in Supplementary Table S4. Overall, 46 papers met the inclusion and exclusion criteria (Table 1 and Table 2, Supplementary Table S5), of which 23 were used for quantitative analysis. The assessment of the risk of bias in the included studies is outlined in Table 3.

### 3.1. Characteristics of Included Studies

All 46 included studies were observational studies on populations outside of North America, with 11 cohort [18,19,20,21,22,23,24,25,26,27,28], 9 cross-sectional [29,30,31,32,33,34,35,36,37], and 26 case–control [38,39,40,41,42,43,44,45,46,47,48,49,50,51,52,53,54,55,56,57,58,59,60,61,62,63] study designs. There were no RCTs that met the criteria for this review. All studies assessed CeD patients, and one study assessed NCWS patients [52]. One study, published as an abstract [50], was supplemented by additional data provided by the authors. The population that was involved comprised adults in 19 studies [23,24,27,30,33,35,37,38,42,43,45,47,51,52,53,54,55,60,62], children in 25 studies [18,19,21,22,25,26,28,29,31,32,34,36,39,40,41,44,46,48,49,50,56,58,59,61,63], and a mix in 2 studies [20,57].

**Table 2:** Characteristics of included studies.

Study	Year	Language	Participants (n)		Adults vs. Children	Method of CeD Diagnosis	Length of GFD	Micronutrients Assessed	Notes
			CeD on GFD	Control (n)					
Dichotomous Data Available									
Anand et al. [38] (UK)	1977	English	36	CeD not on GFD (34) Non-CeD (131)	Adults	Biopsies	Short-term *	Iron	
Ballestero-Fernández et al. [30], 2021 (Spain)	2021	English	64	Non-CeD (74)	Adults	Medical diagnosis (serology +/− biopsy)	Short-term *	Calcium (Ca), iron, folate, vitamin D	-Only vitamin D had dichotomous data provided
Bayrak et al. [31] (Turkey)	2020	English	103	Non-CeD (135)	Children	Biopsies and serology	Long-term	Ferritin, folate, vitamin B12, vitamin 25(OH) D	
Choudhary et al. [32] (India)	2017	English	36	CeD not on GFD (36)	Children	Biopsies and serology.	Short-term *	Ca	
Ciacci et al. [42] (Italy)	2020	English	55	CeD not on GFD (50)	Adults	Biopsies and serology	Long-term	Vitamin 25(OH)D and 1,25(OH)D, Ca	-Reported severe and mild vitamin D deficiency
Elli et al. [47] (Italy)	2015	English	64	Non-CeD (74)	Adults	Medical diagnosis (serology +/− biopsy)	Long-term	Iron, ferritin	-Dichotomous data for iron in CeD only
González et al. [35] (Argentina)	1995	English	12	CeD not on GFD (20)	Adult Females	Biopsies and serology	Long-term	Vitamin 25(OH)D, Ca	-No lab data for non-CeD
Högberg et al. [19] (Sweden)	2009	English	14	CeD not on GFD (11)	Children	Biopsies	Short-term	Zinc	
Hozyasz et al. [20] (Poland)	2003	Polish	12	CeD not on GFD (18)	Both	Biopsies and anti-endomysium ABs	Long-term	Vitamin A, vitamin E	
Kavak et al. [49] (Turkey)	2003	English	34	CeD not on GFD (34)	Children	Biopsies and serology	Short-term *	Vitamin 25(OH)D Ca	-No lab data for non-CeD
Keaveny et al. [23] (Ireland)	1996	English	16	CeD not on GFD (19)	Adults	Biopsies	Long-term	Ionized Ca, vitamin 25(OH)D and 1,25(OH)D	-In total, 4/19 CeD not on a GFD on low-dose vitamin D supplement
Kemppainen et al. [24] (Finland)	1995	English	42	CeD not on GFD (40)	Adults	Biopsies and unclear diagnosis were excluded	Short-term *	Iron, ferritin, Ca, folate, vitamin B12, magnesium (Mg)	-No lab data for controls -Continuous data only for Ca and Mg **
Klimov et al. [50] (Russia)	2017	English	37	CeD not on GFD (22) Non-CeD (14)	Children	Biopsies and serology	Unknown **	Vitamin 25(OH)D	-Conference poster
Margoni et al. [25] (Greece)	2012	English	36	CeD not on GFD (45)	Children	Biopsies and anti-TTG IgA /EMA IgA ABs	Long-term	Vitamin 25(OH)D, Ca	
Mazure et al. [51] (Argentina)	1994	English	14	CED not on GFD (20)	Adults	Biopsies and serology	Long-term	Vitamin 25(OH)D, Ca	- No lab data for non-CeD
McGrogan et al. [26] (Scotland, UK)	2021	English	44	CeD not on GFD (25)	Children	Biopsies and anti-TTG IgA antibodies	Short-term *	Vitamin A/B2/B6/B12/C/K/D/E, zinc, folate, ferritin, Mg, copper, selenium	-Not all participants had all the micronutrients measured
Manseuto et al. [52] (Italy)	2023	English	174 NCWS: 244	Non-CeD (124)	Adults	Biopsies and serology NCWS: no CED dx	Short-term	Ferritin, folate, iron, vitamin B12	-Measured both CeD and NCWS
Piatek-Guziewicz et al. [54] (Poland)	May 2017	English	92	CeD not on GFD (53) Non-CeD (52)	Adults	Biopsies and serology	Long-term	Ferritin, vitamin D vitamin E	-Vitamin E deficiency within CeD groups was not available **
Piatek-Guziewicz et al. [55] (Poland)	Nov 2017	English	31	CeD not on GFD (29) Non-CeD (25)	Adults	Biopsies and serology	Long-term	Vitamin D	
Romańczuk et al. [57] (Poland)	2016	English	48	CeD not on GFD (7) Non-CeD (20)	Both	Biopsies and serology	Unknown **	Vitamin E	
Selbuz et al. [58] (Turkey)	2021	Turkish	22	CeD not on GFD (34)	Children	Biopsies and serology	Short-term *	Zinc; iron folate; Ca Mg; Vitamin A, B12, E, D	- No lab data for non-CeD
Szaflarska-Poplawska et al. [28] (Poland)	2022	English	48	Non-CeD (50)	Children	Biopsies and serology	Long-term	Calcium, folate, magnesium, vitamin B1/B2/B6/B12	
Ünal et al. [61] (Turkey)	2012	Polish	17	Non-CeD (20)	Children	Biopsies and serology	Short-term	Selenium	
Uyanikoglu et al. [62] (Turkey)	2021	English	40	CeD not on GFD (40) Non-CeD (40)	Adults	Biopsies and serology	Unknown **	Vitamin 25(OH)D and 1,25(OH)D	-Reported severe and mild vitamin D deficiency
Continuous Data Only ***									
Ballestero-Fernández et al. [29] (Spain)	2019	English	67	Non-CeD (66)	Children	Medical diagnosis (serology +/− biopsy)	Short-term *	Iron, folate, Ca, vitamin D	-Calculated mean from median
Björck et al. [39] (Sweden)	2017	English	30	Non-CeD (57)	Children	Biopsies and serology	Long-term	25 (OH) vitamin D	
Boda et al. [40] (Hungary)	1989	Hungarian	25	CeD not on GFD (24) Non-CeD (15)	Children	Biopsies	Short-term	Selenium	
Bulut et al. [41] (Turkey)	2023	English	31	CeD not on GFD (18)	Children	Biopsies and serology	Short-term	Vitamin D, Ca	
Corazza et al. [43] (Italy)	1988	English	18	CeD not on GFD (30) Non-CeD (30)	Adults	Biopsies	Short-term *	Zinc, copper	- Lab data for copper not reported
Corazza et al. [33] (Italy)	1995	English	14	CeD not on GFD (17) Non-CeD (24)	Adults	Medical Diagnosis (biopsies or positive serology and family history)	Short-term *	Vitamin 25(OH)D and 1,25(OH)D, Ca	
Cortigiani et al. [44] (Italy)	1989	Italian	36	CeD not on GFD (37) Non-CeD (51)	Both (Age <20)	Biopsies	Short-term *	Selenium	
Dickey et al. [45] (Ireland)	2008	English	41	CeD not on GFD (35) Non-CeD (200)	Adults	Biopsies and serology	Short-term *	Folate, vitamin B2/ B6/B12	
Efe et al. [46] (Turkey)	2023	English	50	Non-CeD (72)	Children	Biopsies and serology	Short-term *	Ferritin, vitamin D, B12, iron	
El Amrousy et al. [34] (Egypt)	2024	English	40	Non-CeD (40)	Children	Biopsies and serology	Short-term	Ferritin, vitamin D	
Henker et al. [18] (Germany)	1985	German	63	CeD not on GFD (48)	Children	Biopsies	Short-term *	Zinc	
Isikay et al. [36] (Turkey)	2018	English	226	Non-CeD (268)	Children	Biopsies and/or EMA IgA and anti-TTG IgA antibodies	Short-term *	Ferritin, folate, vitamin B12 and 25(OH)D	
Kalayci et al. [48] (Turkey)	2001	English	16	CeD not on GFD (16) Non-CeD (82)	Children	Biopsies and serology	Long-term	Ca	
Kalita et al. [21] (Poland)	2002	Polish	31	CeD not on GFD (9) Non-CeD (27)	Children	Biopsies and anti-EMA IgA antibodies	Unknown **	Selenium	
Karkoszka et al. [22] (Poland)	2000	Polish	33	CeD not on GFD (40)	Children	Biopsies and resolution of villous atrophy post-GFD	Long-term	Ca	- GFD adherence confirmed by serology
Pazianas et al. [53] (England)	2005	English	24	Non-CeD (20)	Adult Females	Biopsies	Long-term	Vitamin 25(OH)D, Ca	
Reinken et al. [56] (Austria)	1976	English	6	CeD not on GFD (15) Non-CeD (20)	Children	Biopsies	Short-term *	Vitamin B6	
Sabel’nikova et al. [27] (Russia)	2013	Russian	109	CeD not on GFD (33)	Adults	Biopsies and serum anti- gliadin and TTG IGA ABs	Unknown **	Iron, Ca	
Singhal et al. [59] (India)	2008	English	7	CeD not on GFD (23) Non-CeD (27)	Children	Biopsies and serology	Short-term *	Zinc	
Szymczak et al. [60] (Poland)	2012	English	19	CeD not on GFD (16) Non-CeD (36)	Adults	Biopsies	Short-term *	Vitamin 25(OH)D and 1,25(OH)D, Ca	
Valente et al. [37] (Brazil)	2015	English	40	Non-CeD (40)	Adults	Biopsies	Short-term *	Folate, vitamin B6, vitamin B12	
Volkan et al. [63] (Turkey)	2018	English	21	CeD not on GFD (26) Non-CeD (30)	Children	Biopsies and serology	Short-term *	Ferritin, folate, Mg, Ca, vitamin B12, vitamin K, vitamin 25(OH)D	

* Classified as short-term due to a lack of a clear GFD cutoff. ** Authors were contacted to provide further information. *** Authors were contacted to provide further dichotomous data.

**Table 3:** Risk of bias of included studies with ROBINS-1 [15]. Green—low risk; yellow—moderate risk; red—severe risk.

Author	Year	Type of Study	Bias Due to Confounding	Bias Due to Selection of Participants in the Study	Bias in Classification of Interventions	Bias Due to Deviation from Intervention	Bias Due to Missing Data	Bias in Measurements of Outcomes	Bias in Selection of the Reported Result	Overall Bias
Henker	1985 [18]	Cohort								
Högberg	2009 [19]									
Hozyasz	2003 [20]									
Kalita	2002 [21]									
Karkoszka	2000 [22]									
Keaveny	1996 [23]									
Kemppainen	1995 [24]									
Margoni	2012 [25]									
McGrogan	2021 [26]									
Sabel’nikova	2013 [27]									
Szaflarska-Poplawska,	2022 [28]									
Ballestero-Fernández	2019 [29]	Cross-sectional								
Ballestero-Fernández	2021 [30]									
Bayrak	2020 [31]									
Choudhary	2017 [32]									
Corazza	1995 [43]									
González	1995 [35]									
Isikay	2018 [36]									
Valente	2015 [37]									
Anand	1977 [38]	Case–Control								
Björck	2017 [39]									
Boda	1989 [40]									
Bulut	2023 [41]									
Ciacci	2020 [42]									
Corazza	1988 [43]									
Cortigiani	1989 [44]									
Dickey	2008 [45]									
Efe	2023 [46]									
El Amrousy	2024 [34]									
Elli	2015 [47]									
Kalayci	2001 [48]									
Kavak	2003 [49]									
Klimov	2017 [50]									
Manseuto	2023 [52]									
Mazure	1994 [51]									
Pazianas	2005 [53]									
Piatek-Guziewicz	05-2017 [54]									
Piatek-Guziewicz	11-2017 [55]									
Reinken	1976 [56]									
Romańczuk	2016 [57]									
Selbuz	2021 [58]									
Singhal	2008 [59]									
Szymczak	2012 [60]									
Ünal	2012 [61]									
Uyanikoglu	2021 [62]									
Volkan	2018 [63]									

Further details on the characteristics of the included studies are shown in Table 2 and Supplementary Table S5.

None of the included studies compared whether the presence of GI symptoms affected the prevalence of micronutrient deficiencies or provided data on adherence to a GFD (non-compliant GFD vs. strict GFD) for analysis. There were 15 studies that assessed CeD patients on a long-term GFD [20,22,23,25,28,31,35,39,42,47,48,51,53,54,55], and 26 studies that assessed CeD patients on a short-term GFD [18,19,24,26,29,30,32,33,34,36,37,38,40,41,43,44,45,46,49,52,56,58,59,60,61,63], and 5 that did not report the duration of the GFD, which was classified as a short-length GFD. One study [23] reported that a proportion of CeD patients not on a GFD (4/19) were taking different doses of vitamin D supplementation, with 3 taking < 400 IU daily and 1 taking > 400 IU daily.

### 3.2. Micronutrient Deficiencies in CeD

Of the 41 studies included, 29 involved non-CeD controls [21,28,29,30,31,33,34,36,37,39,40,43,44,45,46,47,48,50,52,53,54,55,56,57,59,60,61,62,63] and 34 studies compared treated CeD patients (on a GFD) with untreated CeD (not on a GFD) [18,19,20,21,22,23,24,25,26,27,31,32,33,35,38,40,41,42,43,44,45,48,49,50,51,54,55,56,57,58,59,60,62,63]. The majority of the studies had data reported as continuous outcomes (serum levels of micronutrients), 3 studies provided raw data available for analysis [20,31,38], and 24 studies reported the proportions of participants with micronutrient deficiency [19,20,23,24,25,26,28,30,32,35,38,39,42,47,49,50,51,52,54,55,57,58,61,62] (Table 2, Supplementary Tables S5 and S6). The pooled data on studies reporting vitamin or mineral deficiencies as dichotomous outcomes are described below, and those reporting vitamin and minerals levels as continuous data are provided as supplementary data.

### 3.3. Vitamin A

Vitamin A was assessed in three studies [20,26,58] with a total of 97 participants. All studies compared treated with untreated CeD. Two studies [20,26] used high-performance liquid chromatography (HPLC) to determine vitamin A levels, and one study [58] did not disclose how vitamin A was measured. One cohort study [20] reported no vitamin A deficiency in either group (Supplementary Figure S1A). The risk of vitamin A deficiency was similar between groups (OR = 1.1; 95%CI = 0.25, 4.86; I^2^ = 14%) (Supplementary Figure S1A, Table S4). The quality of evidence was rated very low due to the risk of bias, imprecision, and inconsistency in the findings (Table 2 and Table 3).

### 3.4. Vitamin B1

There were two studies [26,28] evaluating vitamin B1 in CeD. One cohort study [26] compared vitamin B1 in treated vs. untreated CeD (n = 64) and found no significant difference in vitamin B1 deficiency between groups (OR = 0.20; 95%CI = 0.04, 1.13) (Supplementary Figure S2A, Table S4). Another cohort study [28] assessed vitamin B1 deficiency in CeD on a GFD compared to non-CeD controls and found no significant risk of B1 deficiency in treated CeD (Supplementary Figure S2B, Table S5). The quality of evidence was rated very low due to the risk of bias and imprecision (Table 2 and Table 3).

### 3.5. Vitamin B2

Vitamin B2 was assessed in three studies, one cohort [26], and two case–control [28,45] studies with a total of 440 participants. The methodology to determine the levels of vitamin B2 differed; one study [26] controlled serum concentrations by hemoglobin, and the other study [45] by erythrocyte glutathione reductase activation coefficient (EGRAC) activity (Table 2). Only one study [26] provided dichotomous data and reported no vitamin B2 deficiency in either of both groups (Table 4). One study [28] found no significant increased risk of vitamin B2 deficiency in CeD on a GFD compared to non-CeD controls (Supplementary S3A). The quality of evidence was rated very low due to the risk of bias (Table 3).

### 3.6. Vitamin B6

Three studies assessed vitamin B6 in a total of 251 participants, two studies [26,56] compared treated vs. untreated CeD, and three studies [28,37,56] compared treated CeD with non-CeD controls. Vitamin B6 levels were measured in erythrocytes by HPLC controlled for hemoglobin [26] and measured in serum by pyridoxal phosphate (PALP) (ng/mL) [56] and pyridoxal-5-phosphate (PLP) (pmol/g) [37] by chromatography. One study [26] found similar rates of vitamin B6 deficiency in treated and untreated CeD patients (OR = 0.18; 95%CI = 0.02, 1.83) (Supplementary Figure S4A, Table S4). In addition, there were no events of vitamin B6 deficiency in one study [28] comparing CeD on a GFD with non-CeD controls (Table 5). The quality of evidence was rated very low due to the risk of bias and imprecision (Table 2 and Table 3).

### 3.7. Vitamin B12

Eight studies assessed serum vitamin B12 in a total of 1750 participants, five studies compared treated and untreated CeD [24,26,45,58,63], and six studies compared treated CeD with non-CeD controls [31,36,37,45,52,63]. The pooled results of three studies [24,26,58] showed significantly lower odds of vitamin B12 deficiency in treated CeD compared with in untreated CeD (OR = 0.09; 95%CI = 0.01,0.68; I^2^ = 0%) (Table 4; Supplementary Figure S5A).

The odds of vitamin B12 deficiency were similar between treated CeD compared with the non-CeD controls in three studies [28,31,52] (OR = 3.30; 95% CI = 0.19, 61.78; I^2^ = 92%) (Table 5; Supplementary Figure S5B). The overall quality of evidence was deemed to be very low due to the risk of bias in the study methodology and imprecision (Table 3).

### 3.8. Vitamin C

Only one study [26] assessed vitamin C, providing data on 37 cases of treated CeD on a GFD over 1 year with no patients having vitamin C deficiency [26]. Risk calculation was not possible due to the lack of data available for extraction in a control group. The quality of data was rated very low due to the risk of bias and imprecision (Table 3).

### 3.9. Vitamin D

A total of 21 studies assessed vitamin D/vitamin 25 (OH) D status in a total of 2367 participants, with 15 comparing treated and untreated CeD [23,25,26,33,35,42,49,50,51,54,55,58,60,62,63] and 13 comparing treated CeD with non-CeD controls [29,30,31,33,36,39,50,53,54,55,60,62,63].

**Figure 2:**
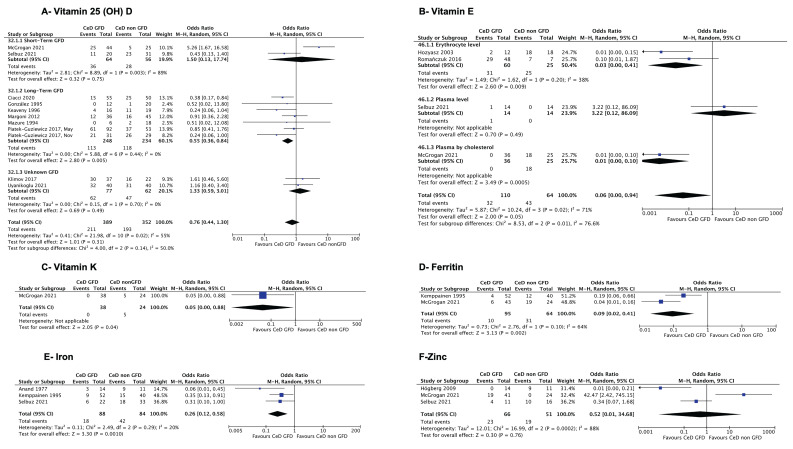
Forest plot of micronutrient deficiencies in CeD on a GFD compared to CeD not on a GFD. (**A**) Vitamin 25 (OH) deficiency subgrouped by the duration of the GFD. (**B**) Vitamin E deficiency subgrouped by the study design. (**C**) Vitamin K deficiency as controlled by triglycerides. (**D**) Low ferritin. (**E**) Iron deficiency. (**F**) Zinc deficiency. The figure with the corresponding risk of bias is within Supplemental Figure S19. [19,20,23,24,25,26,35,38,42,50,51,54,55,57,58,62].

There was no significant difference in the proportion of patients with vitamin D deficiency between CeD on a GFD and not on a GFD in 11 pooled studies [23,25,26,35,42,50,51,54,55,58,62] (OR = 0.76; 95% CI: 0.44, 1.30; I^2^ = 55%) (Supplementary Figure S6A(i)). The funnel plot did not show significant asymmetry (Egger’s test intercept = −0.03; 95% CI: −1.66, 1.59; t= −0.05; *p* = 0.964) (Supplementary Figure S6A(ii)). Subgroup analysis by GFD duration demonstrated a significant decrease in the proportion of patients with vitamin D deficiency in CeD on a long-term GFD (OR = 0.55; 95% CI: 0.36, 0.84; I^2^ = 0%) (Figure 2A; Table 4) and by population age (OR = 0.55; 95% CI: 0.34, 0.87; I^2^ = 9%) and resolved heterogeneity (Supplementary Figure S6C). Sensitivity analysis removing the one study [23] that reported low-dose vitamin D supplementation in 4/19 of the group with CeD not on a GFD did not affect the results. The quality of evidence was rated very low due to the risk of bias and inconsistency (Table 3).

**Figure 3:**
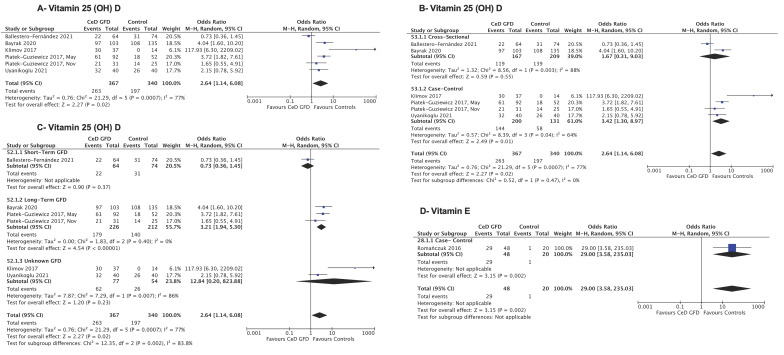
Forest plots’ comparison of micronutrient deficiencies in CeD on a GFD compared to non- CeD controls. (**A**) Vitamin D deficiency, (**B**) vitamin D deficiency stratified by study design, (**C**) vitamin D deficiency stratified by the duration of the GFD length, (**D**) vitamin E deficiency in a case–control study. The figure with the corresponding risk of bias is within Supplemental Figure S20. [30,31,50,54,55,57,62].

Patients with treated CeD had significantly higher odds of vitamin D deficiency compared with non-CeD controls in six pooled studies [30,31,50,54,55,62] (OR = 2.64; 95%CI: 1.14, 6.08; I^2^ = 77%) (Figure 3A; Table 5). Heterogeneity was not resolved after subgroup analysis by study design (Figure 3B) or by age (Supplementary Figure S6D). Subgroup analysis by the length of the GFD resolved heterogeneity in the long-term GFD (OR = 3.21; 95% CI: 1.94, 5.30; I^2^ = 0%) (Figure 3C). The quality of evidence was rated very low due to the risk of bias, imprecision, and inconsistency (Table 2 and Table 4).

Only one study [42] assessed 1,25(OH) vitamin D deficiency in treated vs. untreated CeD. There was no difference in the odds of 1,25(OH) vitamin D deficiency between groups (OR = 0.30; 95%CI: 0.01, 7.47) (Table 4; Supplementary Figure S7A). The quality of evidence was rated very low due to the risk of bias (Table 3).

### 3.10. Vitamin E

Five studies [20,26,54,57,58] assessed serum vitamin E levels in treated vs. untreated CeD, and two studies [54,57] compared treated CeD with non-CeD controls. Vitamin E was assessed by different methods, including chromatography controlled for plasma cholesterol (μmol/mmol) [20,26], plasma levels alone [20], levels in erythrocytes (μmol/L) [20,57], and serum levels by spectrophotography [54]. One study did not provide information on the methodology used to measure vitamin E [58].

There were lower odds of vitamin E deficiency in treated CeD compared to in untreated CeD (OR = 0.06; 95% CI: 0.00, 0.94; I^2^ = 71%) (Supplementary Figure S8A). Subgroup analysis by this method did not resolve heterogeneity (Figure 2B). The quality of evidence was rated very low due to the risk of bias (Table 3).

Compared to non-CeD controls, one study [57] found higher odds of vitamin E deficiency in treated CeD (OR = 29; 95% CI: 3.58, 235.03) (Figure 3D). The remaining case–control study [54] reported that >60% of CeD patients had vitamin E deficiency, which included both CeD patients on and off a GFD compared to 3.7% of controls. Authors were contacted to obtain more data; however, no response was received. The quality of evidence was rated very low due to the risk of bias and imprecision (Table 2 and Table 4).

### 3.11. Vitamin K

Two studies [26,63] assessed serum vitamin K levels in a total of 109 participants, with one cohort [26] comparing treated vs. untreated CeD and one case–control [63] comparing treated CeD to both untreated CeD and non-CeD controls. The cohort study [26] showed significantly lower odds of vitamin K deficiency in treated CeD compared to in untreated CeD (OR = 0.05; 95%CI: 0.00, 0.88; Figure 2C). The quality of evidence was rated very low due to the risk of bias (Table 3).

### 3.12. Calcium

A total of 20 studies assessed serum calcium levels, and one study [23] assessed ionized calcium in a total of 1350 participants. Fifteen studies [22,23,24,25,27,32,33,35,42,48,49,51,58,60,63] evaluated calcium levels in treated CeD compared to in untreated CeD, and eight studies [28,29,30,33,48,53,60,63] evaluated calcium levels in treated CeD compared to in non-CeD controls. Seven pooled studies [25,32,35,42,49,51,58] showed a significantly lower rate of calcium deficiency in treated CeD compared with in untreated CeD (OR = 0.33; 95% CI: 0.17, 0.64; I^2^ = 46%) (Table 4; Supplementary Figure S10A). Subgroup analysis by study design resolved the heterogeneity in the pooled analysis (Supplementary Figure S10B–D). The quality of evidence was rated to be very low due to the risk of bias and inconsistency (Table 3).

There was no difference in the odds of calcium deficiency between treated CeD compared to non-CeD controls based on one study [28] (OR = 0.34; 95% CI: 0.01, 8.56) (Table 5, Supplementary Figure S10E). The quality of evidence was rated very low due to the risk of bias (Table 3).

### 3.13. Copper

One cohort study [26] with 65 participants assessed serum copper levels in treated CeD compared with in untreated CeD. The odds of copper deficiency were higher in treated CeD, (OR = 3.94; 95% CI: 0.45, 34.93) (Table 4, Supplementary Figure S11A). However, the quality of evidence was rated very low due to the risk of bias and imprecision (Table 2 and Table 3).

### 3.14. Folate

Eleven studies assessed serum folate levels, and one study [24] assessed folate levels in erythrocytes in a total of 2096 participants, four studies [26,45,58,63] compared treated CeD with untreated CeD, and nine studies [28,29,30,31,36,37,45,52,63] used non-CeD controls. There was no difference in the odds of serum folate deficiency between treated CeD and untreated CeD in two studies [26,58] (OR = 0.21; 95% CI: 0.01, 3.85) (Table 4; Supplementary Figure S12A). However, there were significantly lower odds of erythrocyte folate deficiency in treated CeD compared to in untreated CeD in one study [24] (OR = 0.07; 95% CI: 0.02, 0.35) (Supplementary Figure S12B). The quality of evidence was rated very low due to the risk of bias (Table 3).

There was no difference in the odds of serum folate deficiency between treated CeD compared to non-CeD controls in the three studies [28,31,52] (OR = 7.16; 95% CI: 0.61, 84.15; I^2^ = 88%) (Table 5, Supplementary Figure S12C). The quality of evidence was rated very low due to the risk of bias and imprecision (Table 3).

### 3.15. Iron Status—Ferritin

Eight studies assessed serum ferritin levels in a total of 1573 participants, four studies [24,26,54,63] compared treated CeD with untreated CeD, and six studies [31,36,47,52,54,63] compared treated CeD with non-CeD controls. The results from two pooled studies [24,26] found significantly decreased odds of low ferritin levels in treated CeD compared with in untreated CeD (OR = 0.09; 95% CI: 0.02, 0.41; I^2^ = 64%) (Figure 2D, Table 4). The quality of evidence was rated very low due to the risk of bias (Table 3).

Two studies [31,52] comparing treated CeD with non-CeD controls found no significant difference in the odds of low ferritin (OR = 3.82; 95% CI: 0.53, 27.63) (Table 5, Supplementary Figure S13A). The quality of evidence was rated very low due to the risk of bias and imprecision (Table 3).

### 3.16. Iron

A total of nine included studies assessed serum iron levels in a total of 1235 participants, four studies [24,27,38,58] compared treated CeD with untreated CeD, and five studies [29,30,31,47,52] used non-CeD controls. Three pooled studies [24,38,58] showed significantly lower odds of iron deficiency in treated CeD compared to in untreated CeD (OR = 0.26; 95% CI: 0.12, 0.58; I^2^ = 20%) (Table 4, Figure 2E). The quality of evidence was rated very low due to the risk of bias and study design (Table 3).

Two pooled studies [31,52] found no difference in the odds of iron deficiency in treated CeD compared to in non-CeD controls (OR = 14.58; 95% CI: 0.01, 28,105.67) (Table 5, Supplementary Figure S14A). The quality of evidence was rated very low due to the risk of bias (Table 3).

### 3.17. Magnesium

A total of five studies assessed magnesium deficiency in a total of 300 participants, four studies [24,26,58,63] compared treated CeD with untreated CeD, and two studies [28,63] used non-CeD controls. There was no significant difference in the rate of magnesium deficiency in two studies [26,58] comparing treated and untreated CeD (OR = 0.40; 95% CI: 0.05, 3.25; I^2^ = 0%) (Table 4, Supplementary Figure S15A). In addition, there were no events of magnesium deficiency in one study [28] comparing CeD on a GFD with non-CeD controls (Table 5). The quality of evidence was rated very low due to the risk of bias and imprecision (Table 3).

### 3.18. Selenium

Four studies [21,26,44,61] assessed serum selenium, and one study [40] assessed selenium in erythrocytes in a total of 279 participants. Four studies [21,26,40,44] compared treated CeD with untreated CeD, and four studies [21,40,44,61] compared treated CeD with non-CeD controls. There was no significant difference in the odds of selenium deficiency in treated CeD compared with in untreated CeD in one cohort study [26] (OR = 1.19; 95% CI: 0.20, 7.03) (Table 4, Supplementary Figure S16A). The quality of evidence was rated very low due to the risk of bias (Table 3).

There was no difference in the odds of serum selenium deficiency in treated CeD compared to in non-CeD controls in one case–control study [61] (OR = 0.35; 95% CI: 0.03, 3.77) (Table 5; Supplementary Figure S16B). The quality of evidence was rated very low due to the risk of bias and imprecision (Table 3).

### 3.19. Zinc

Six studies assessed serum zinc levels in a total of 336 participants, all studies [18,19,26,43,58,59] compared treated CeD with untreated CeD, and two studies [43,59] used non-CeD controls. A study [18] assessing zinc levels in treated and untreated CeD reported the proportion of patients with zinc levels under two standard deviations of the mean but did not report the proportion of the population with zinc deficiency. The authors were contacted for clarification, but no response was received. Three pooled studies [19,26,58] showed no significant differences in the odds of zinc deficiency between treated and untreated CeD (OR = 0.52; 95% CI: 0.01, 34.68; I^2^ = 88%; Figure 2F). Subgroup analysis by study design did not resolve heterogeneity (Supplementary Figure S17). The quality of evidence was rated very low due to the risk of bias, inconsistency, and imprecision (Table 3).

### 3.20. Micronutrient Deficiencies in NCWS

One study assessed micronutrient deficiencies in 244 patients with NCWS [52] on a gluten-free diet including vitamin B12, folate, and iron. This study found significantly increased odds for all four deficiencies in NCWS compared to in controls on a normal diet (Supplementary Figure S19, Table 6). The quality of evidence was rated very low due to the risk of bias, inconsistency, and imprecision (Table 6).

## 4. Discussion

In this comprehensive systematic review and meta-analysis, we found an increased risk of various micronutrient deficiencies in patients with CeD and in patients with NCWS adopting a GFD. Data from over 20 studies on CeD and one on NCWS showed that patients with treated CeD had an increased risk for vitamin A, vitamin D, iron, folate, and copper deficiency, and patients with NCWS were at a higher risk of folate, iron, and vitamin B12 deficiency compared to non-CeD controls. This is the first systematic review with meta-analysis to explore multiple micronutrient deficiencies in both adults and pediatric populations with CeD and NCWS.

The largest body of evidence found for this review investigated micronutrients related to bone metabolism, such as vitamin D and calcium in CeD, but no studies were found on NCWS. Vitamin D deficiency was more common in treated CeD compared to both in untreated CeD and non-CeD controls, which is consistent with a previous systematic review on a pediatric population with CeD [64]. The association between vitamin D deficiency and CeD could be related to multiple factors including malabsorption secondary to villous atrophy in active CeD [65] and environmental factors including a GFD and sunlight exposure [39,42,53]. Interestingly, vitamin D deficiency was increasingly common in CeD on a long-term GFD, suggesting that dietary restriction may contribute to vitamin D deficiency [66]. Considering the high risk of vitamin D deficiency, ongoing monitoring and replacement of vitamin D seem appropriate in a proportion of CeD cases with vitamin D deficiency; however, it is unclear how often monitoring is required or if it is necessary for all CeD patients. Furthermore, secondary hyperparathyroidism is responsible for the hyperconversion of 25-vitamin D into 1,25-vitamin D, and therefore the determination of serum levels of both vitamin metabolites is important to confirm the diagnosis of vitamin D deficiency [65,67]. Unlike vitamin D, the rates of calcium deficiency were similar in treated and untreated CeD compared to in non-CeD controls. Although calcium absorption is presumed to be impaired in CeD [65], assessing calcium deficiency is challenging as serum calcium is typically held in the normal range due to bone resorption [65], and therefore the routine measurement of calcium is not often recommended in CeD.

Other markers, like bone density tests, can also be used to further investigate the extent of calcium deficiency [5,25]. This review found a significantly decreased risk of developing iron deficiency in CeD on a short-term GFD compared to CeD not on a GFD, which is expected as untreated CeD can lead to villous atrophy and impaired iron absorption [68,69]. Although a recovery of enteropathy in less than 2 years is unlikely, iron deficiency anemia can also persist despite mucosal healing, suggesting other possible mechanisms, including impaired iron absorption, occult blood loss, or a pro-inflammatory state [68], as well as a lower intake of food rich in iron after initiating a GFD [7,9,10], or an increased intake of food high in phytates, known inhibitors of iron absorption [70]. The latter mechanisms could explain the increased risk of iron deficiency in NCWS adopting a GFD [52]. Regardless of the reason for iron deficiency, the recent American College of Gastroenterology (ACG) guidelines [68] suggest monitoring iron levels in CeD patients if they are abnormal initially and supplementing as needed with either oral supplements or intravenous iron infusion [69]. There are no guidelines addressing recommendations on how to treat iron deficiency in NCWS.

CeD on a GFD had a lower risk of vitamin E and vitamin K deficiency when compared to untreated CeD, which is expected given the improvement in fat-soluble vitamin absorption with treatment. There were no studies evaluating risk of vitamin E or K deficiency in NCWS. The included studies evaluating vitamin K [26,63] deficiency in CeD only measured isolated serum levels of vitamin K and did not measure INR, which reflects the clinical relevance of vitamin K deficiency in CeD. Furthermore, given the high heterogeneity between studies, there is a need for larger studies with good quality to understand the prevalence of vitamin E and vitamin K deficiency, and whether supplementation is necessary in CeD and NCWS.

Other micronutrients, including vitamin A, vitamin B12, copper, magnesium, and selenium were not significantly lower in treated CeD compared with in untreated CeD. The results are inconsistent with previous reports of a lower intake of these micronutrients by individuals adopting a GFD [7,9,10]; however, the body of evidence is scarce, and therefore further studies are needed to confirm the findings. Treated CeD and NCGS were at higher risk of folate deficiency, which is likely related to the lower folic acid content of gluten-free food [71]. Furthermore, this review showed that the risk of zinc deficiency may be lower in treated CeD compared to in untreated CeD; however, significant imprecision and inconsistency in the data reduces confidence in the results. These results are consistent with a recent review focused on the pediatric population [72], reporting that untreated pediatric CeD patients had significantly lower serum zinc levels which subsequently returned to normal upon adopting a GFD. Furthermore, an increased risk of zinc deficiency was reported in adult patients with and without CeD who adopt a GFD, suggesting that diet may play a role beyond malabsorption [73]. Further larger studies with a more rigorous design may help clarify the prevalence and severity of zinc deficiency in pediatric and adult patients with CeD and NCWS compared to in a non-celiac population.

Our review found decreased levels of vitamin D, vitamin E, selenium, iron, and zinc in CeD compared to in non-CeD controls. However, the clinical significance of comparing the mean levels of micronutrients is unclear, as the levels may still be in the normal reference range despite differences between groups. In addition, the various methodologies used to determine micronutrient levels lead to increased heterogeneity in results, which makes pooling data challenging.

The methodology used in this review was rigorous, which is a strength of this review. We attempted to contact authors when data were missing and reduce the risk of bias as much as possible and created summary tables on the evidence of nutrients. We conducted subgroup analyses to explore sources of heterogeneity; however, only subgroup analyses by a GFD and by population age resolved heterogeneity in CeD patients with vitamin D deficiency. Reasons for the increased heterogeneity in other analyses remain unclear and are possibly related to the study design or definition of CeD, which can be diagnosed with different tools. Based on our analysis, the data are not robust enough to draft definitive recommendations as the quality of the evidence was consistently rated low or very low, creating major limitations in the interpretation of the results. In addition, most studies reported micronutrient levels without providing a cutoff for normality; therefore, data on micronutrient deficiency are limited. Furthermore, all the studies assessed in this review were from populations outside North America which may limit generalizability to North Americans. Finally, other factors including dietary intake and environmental and socioeconomic circumstances, which may influence nutrient deficiencies, were not controlled for in many studies.

Patients with NCWS adopting a GFD are at an increased risk of micronutrient deficiencies, which is concerning as the prevalence of individuals adopting a GFD is increasing [74]; there is insufficient awareness of this condition and there are no guidelines on what to recommend for micronutrient monitoring and supplementation.

In conclusion, patients with CeD are at a higher risk of multiple micronutrient deficiencies, although some returned to normal after treatment with GFD. Micronutrient deficiencies are reported in patients with NCWS adopting a GFD; however, the body of evidence is small. Future large, rigorously designed studies evaluating micronutrient deficiencies in CeD, NCWS, and other populations adopting a GFD are needed to improve the certainty of the evidence and to guide recommendations on monitoring and treating nutrient deficiencies in patients adopting a GFD.

## Figures and Tables

**Table 1:** PICOS Criteria.

PICOS Criteria	Inclusion Criteria
Participants	Adults or children with a confirmed diagnosis of celiac disease or NCWS
Interventions	Assessment of micronutrient status
Comparisons	Celiac patients not on a GFD, non-celiac controls
Outcomes	Micronutrient deficiencies; gastrointestinal (GI) symptoms); extraintestinal symptoms; quality of life
Study Design	Observational studies and randomized controlled trials

**Table 4:** Summary of findings table for micronutrient deficiencies in CeD on a GFD compared to CeD not on a GFD.

Micronutrient Deficiencies Associated with Gluten-Free Diet Population: CeD Intervention: Gluten-Free Diet Comparison: CeD Not on Gluten-Free Diet						
Outcomes	Micronutrient	Absolute Comparative Risks		Relative Effect (95% CI)	No. of Participants (Studies)	Quality of the Evidence (GRADE)
		CeD Non-GFD	CeD GFD			
Micronutrient Deficiency	**Vitamin A**	7/47 (14.9%)	10/50 (20%) **12 more per 1000** (from 107 fewer to 311 more)	**OR 1.10** (0.25 to 4.86)	97 (3)^1^	⨁◯◯◯ * VERY LOW due to risk of bias and inconsistency
	**Vitamin B1**	5/24 (20.8%)	2/40 (5%) **158 fewer per 1000** (from 198 fewer to 21 more)	**OR 0.20** (0.04 to 1.13)	64 (1)^2^	⨁◯◯◯ VERY LOW due to risk of bias
	**Vitamin B2**	0/24 (0%)	0/42 (0%) **0 fewer per 1000** (from 60 fewer to 60 more)	Not estimable	66 (1)^2^	⨁◯◯◯ VERY LOW † due to risk of bias
	**Vitamin B6**	3/24 (12.5%)	1/40 (2.5%) **100 fewer per 1000** (from 122 fewer to 82 more)	**OR 0.18** (0.02 to 1.83)	64 (1)^2^	⨁◯◯◯ VERY LOW due to risk of bias
	**Vitamin B12**	10/95 (10.5%)	0/115 (0%) **95 fewer per 1000** (from 104 fewer to 31 fewer)	**OR 0.09** (0.01 to 0.68)	210 (3)^1^	⨁◯◯◯ VERY LOW due to risk of bias
	**25(OH)** **Vitamin D**	193/352 (54.8%)	211/389 (54.2%) **68 fewer per 1000** (from 200 fewer to 64 more)	**OR 0.76** (0.44 to 1.30)	741 (11)^4^	⨁◯◯◯ VERY LOW due to risk of bias and inconsistency
	**1,25(OH)** **Vitamin D**	1/50 (2%)	0/55 (0%) **14 fewer per 1000** (from 20 fewer to 112 more)	**OR 0.30** (0.01 to 7.47)	105 (1)^3^	⨁◯◯◯ VERY LOW due to risk of bias
	**Vitamin E**	43/64 (67.3%)	32/110 (29.1%) **562 fewer per 1000** (from 14 fewer to 0)	OR 0.06 ** (0.00 to 0.94)	174 (4)^1^	⨁◯◯◯ * † VERY LOW due to risk of bias and inconsistency
	**Vitamin K**	5/24 (20.8%)	0/38 (0%) **195 fewer per 1000** (from 20 fewer to 0)	OR 0.05 ** (0.00 to 0.88)	62 (1)^2^	⨁◯◯◯ VERY LOW due to risk of bias
	**Calcium**	40/234 (17.1%)	14/197 (7.1%) **107 fewer per 1000** (from 137 fewer to 54 fewer)	OR 0.33 ** (0.17 to 0.64)	431 (7)^4^	⨁◯◯◯ VERY LOW due to risk of bias and inconsistency
	**Copper**	1/24 (4.2%)	6/41 (14.6%) **105 more per 1000** (from 22 fewer to 561 more)	**OR 3.94** (0.45 to 34.93)	65 (1)^2^	⨁◯◯◯ VERY LOW due to risk of bias, and imprecision
	**Folate**	3/36 (8.3%)	0/31 (0%) **66 fewer per 1000** (from 82 fewer to 178 more)	**OR 0.19** (0.01 to 3.89)	67 (2)^1^	⨁◯◯◯ VERY LOW due to risk of bias and inconsistency
	**Ferritin**	31/64 (48.4%)	10/95 (10.5%) **406 fewer per 1000** (from 466 fewer to 206 fewer)	OR 0.09 ** (0.02 to 0.41)	159 (2)^2^	⨁◯◯◯ VERY LOW due to risk of bias
	**Iron**	42/84 (50%)	18/88 (20.5%) **294 fewer per 1000** (from 393 fewer to 133 fewer)	OR 0.26 ** (0.12 to 0.58)	172 (3)^2^	⨁⨁◯◯ LOW due to study design
	**Magnesium**	1/59 (1.7%)	3/50 (1.7%) **35 fewer per 1000** (from 57 fewer to 112 more)	**OR 0.40** (0.05 to 3.25)	109 (2)^1^	⨁◯◯◯ VERY LOW due to risk of bias and imprecision
	**Selenium**	2/24 (8.3%)	4/41 (9.8%) **14 more per 1000** (from 65 fewer to 307 more)	**OR 1.19** (0.20 to 7.03)	65 (1)^2^	⨁◯◯◯ VERY LOW due to risk of bias
	**Zinc**	19/51 (37.3%)	24/66 (36.4%) **130 fewer per 1000** (from 367 fewer to 585 more)	**OR 0.54** (0.01 to 37.98)	117 (3)^1^	⨁◯◯◯ †† VERY LOW due to risk of bias, inconsistency, and imprecision

CI: confidence interval; OR: odds ratio; MD: mean difference; GRADE: Working Group grades of evidence. High quality: further research is very unlikely to change our confidence in the estimate of effect. Moderate quality: further research is likely to have an important impact on our confidence in the estimate of effect and may change the estimate. Low quality: further research is very likely to have an important impact on our confidence in the estimate of effect and is likely to change the estimate. Very low quality: we are very uncertain about the estimate. ⨁◯ symbols are used to determine the GRADE methodology for level of quality of evidence * Different methodology used to measure vitamin. ** *p* < 0.05. † Case–control study assessed was not significant and increased heterogeneity leading to not-combining studies. †† Significant heterogeneity. ^1^ Studies were a mix of a cohort and case–control study design. ^2^ Cohort study design. ^3^ Case–control study design. ^4^ Studies were a mix of a cohort, cross-sectional, and case–control study design.

**Table 5:** Summary of findings table for micronutrient deficiencies in CeD on a GFD compared to non-CeD.

Micronutrient Deficiencies Associated with Gluten-Free Diet Population: CeD Intervention: Gluten-Free Diet Comparison: Non-CeD Controls						
Outcomes	Micronutrient	Absolute Comparative Risks		Relative Effect (95% CI)	No. of Participants (Studies)	Quality of the Evidence (GRADE)
		Non-CeD Controls	CeD GFD			
Micronutrient Deficiency	**Vitamin B1**	1/50 (2.0%)	7/48 (14.6%) **126 more per 1000** (from 0 fewer to 571 more)	**OR 8.37** (0.99 to 70.82)	98 (1)^1^	⨁◯◯◯ VERY LOW due to risk of bias
	**Vitamin B2**	4/50 (8.0%)	4/48 (8.3%) **4 more per 1000** (from 59 fewer to 199 more)	**OR 1.05** (0.25 to 4.44)	98 (1)^1^	⨁◯◯◯ VERY LOW † due to risk of bias
	**Vitamin B6**	0/50 (0%)	0/48 (0%) **0 fewer per 1000** (from 0 fewer to 0 fewer)	Not estimable	98 (1)^1^	⨁◯◯◯ VERY LOW due to risk of bias
	**Vitamin B12**	30/309 (9.7%)	83/325 (25.5%) **170 more per 1000** (from 77 fewer to 772 more)	**OR 3.39** (0.19 to 61.78)	634 (3)^3^	⨁◯◯◯ VERY LOW due to risk of bias
	**25(OH)** **Vitamin D**	Overall			707 (6)	⨁◯◯◯ VERY LOW due to risk of bias and imprecision and inconsistency
		197/340 (57.9%)	263/367 (71.7%) **205 more per 1000** (from 32 fewer to 314 more)	OR 2.64 ** (1.14 to 6.08)		
		Long-Term GFD				
		140/212 (66.0%)	179/226 (79.2%) **202 more per 1000** (from 130 more to 251 more)	OR 3.21 ** (1.94 to 5.30)		
	**Vitamin E**	1/20 (5.0%)	29/48 (60.4%) **554 more per 1000** (from 109 more to 875 more)	OR 29.00 ** (3.58 to 235.0)	68^2^ (1)	⨁◯◯◯ * VERY LOW due to risk of bias and imprecision
	**Calcium**	1/50 (2.0%)	0/48 (0%) **13 fewer per 1000** (from 20 fewer to 129 more)	**OR 0.34** (0.01 to 8.56)	98 (1)^1^	⨁◯◯◯ VERY LOW due to risk of bias and inconsistency
	**Folate**	12/309 (3.9%)	96/325 (29.5%) **186 more per 1000** (from 15 fewer to 734 more)	**OR 7.16** (0.61 to 84.15)	634 (3)^3^	⨁◯◯◯ VERY LOW due to risk of bias
	**Ferritin**	78/259 (30.1%)	162/277 (58.5%) **321 more per 1000** (from 115 fewer to 621 more)	**OR 1.40** (0.84 to 2.35)	536 (2)^3^	⨁◯◯◯ VERY LOW due to risk of bias
	**Iron**	28/259 (22.4%)	130/277 (46.9%) **584 more per 1000** (from 221 fewer to 776 more)	**OR 14.58** (0.01 to 28,105.67)	536 (2)^3^	⨁◯◯◯ VERY LOW due to risk of bias
	**Magnesium**	0/50 (0%)	0/48 (0%) **0 fewer per 1000** (from 0 fewer to 0 fewer)	**Not estimable**	98 (1)^1^	⨁◯◯◯ VERY LOW due to risk of bias and imprecision
	**Selenium**	3/20 (15%)	1/17 (5.9%) **92 fewer per 1000** (from 145 fewer to 250 more)	**OR 0.35** (0.03 to 3.77)	37 (1)^2^	⨁◯◯◯ VERY LOW due to risk of bias and imprecision

CI: confidence interval; OR: odds ratio; MD: mean difference; GRADE Working Group grades of evidence. High quality: further research is very unlikely to change our confidence in the estimate of effect. Moderate quality: further research is likely to have an important impact on our confidence in the estimate of effect and may change the estimate. Low quality: further research is very likely to have an important impact on our confidence in the estimate of effect and is likely to change the estimate. Very low quality: we are very uncertain about the estimate. ⨁◯ symbols are used to determine the GRADE methodology for level of quality of evidence * Different methodology used to measure vitamin. ** *p* < 0.05. † Case–control study assessed was not significant and increased heterogeneity leading to not-combining studies. ^1^ Cohort study design. ^2^ Case–control study design. ^3^ Case–control and Cross-sectional study design.

**Table 6:** Summary of findings table for micronutrient deficiencies in NCWS compared to healthy controls.

Micronutrient Deficiencies Associated with Gluten-Free Diet Population: NCWS Intervention: Gluten-Free Diet Comparison: Healthy Controls on Regular Diet						
Outcomes	Micronutrient	Absolute Comparative Risks		Relative Effect (95% CI)	No. of Participants (Studies)	Quality of the Evidence (GRADE)
		Healthy Controls	NCWS			
Micronutrient Deficiency	**Vitamin B12**	2/124 (1.6%)	30/244 (12.3%) **77 more per 1000** (from 16 more to 358 more)	OR 8.55 ** (2.01 to 36.40)	368 (1)^1^	⨁◯◯◯ VERY LOW due to risk of bias
	**Folate**	0/124 (0%)	34/244 (13.9%) **0 fewer per 1000** (from 0 fewer to 0 more)	OR 40.81 ** (2.48 to 671.51)	368 (1)^1^	⨁◯◯◯ VERY LOW due to risk of bias
	**Ferritin**	16/124 (12.9%)	99/244 (40.6%) **277 more per 1000** (from 147 more to 421 more)	OR 4.61 ** (2.57 to 8.26)	368 (1)^1^	⨁◯◯◯ VERY LOW due to risk of bias
	**Iron**	0/124 (0%)	47/244 (19.3%) **0 fewer per 1000** (from 0 fewer to 0 more)	OR 59.89 ** (3.66 to 980.26)	368 (1)^1^	⨁◯◯◯ VERY LOW due to risk of bias

CI: confidence interval; OR: odds ratio; MD: mean difference; GRADE Working Group grades of evidence. High quality: further research is very unlikely to change our confidence in the estimate of effect. Moderate quality: further research is likely to have an important impact on our confidence in the estimate of an effect and may change the estimate. Low quality: further research is very likely to have an important impact on our confidence in the estimate of effect and is likely to change the estimate. Very low quality: we are very uncertain about the estimate. ⨁◯ symbols are used to determine the GRADE methodology for level of quality of evidence ** *p* < 0.05. ^1^ Case–control study design.

## Data Availability

Data are available within the manuscript and Supplementary Materials; additional data will be shared upon request.

## Supplementary Materials

The following supporting information can be downloaded at https://www.mdpi.com/article/10.3390/jcm14144848/s1. Table S1: Search strategy terms; Table S2: Search strategy used for EMBASE (OVID) (1974-04/2024); Table S3: Equations used to estimate mean ($\bar{X}$) and standard deviation (σ) from Median (m); Table S4: Studies excluded after full text review and primary reason for exclusion; Table S5: Detailed Characteristics of included studies; Table S6: Summary of all studies evaluating micronutrient levels in CeD on GFD compared to either control group; Table S7: Summary table of nutrient deficiencies in treated CeD compared with untreated CeD or non-celiac controls and NCWS; Table S8: Summary table of nutrients in found in each included study. Supplementary Figure S1A–B: Vitamin A; Supplementary Figure S2A–D: Vitamin B2; Supplementary Figure S3A–D: Vitamin B2; Supplementary Figure S4A–D: Vitamin B6: Supplementary Figure S5A–G: Vitamin B12; Supplementary Figure S6A–L: Vitamin D; Supplementary Figure S7A-E: 1,25-OH Vitamin D; Supplementary Figure S8A–H: Vitamin E; Supplementary Figure S9A–B: Vitamin K; Supplementary Figure S10A–M: Calcium; Supplementary Figure S11A–B: Copper; Supplementary Figure S12A–J: Folate; Supplementary Figure S13A–F: Ferritin; Supplementary Figure S14A–H: Iron; Supplementary Figure S15A–E: Magnesium; Supplementary Figure S16A–I: Selenium; Supplementary Figure S17A–D: Zinc; Supplementary Figure S18A–H: NCWS data; Supplementary Figure S19—Forest plot of micronutrient deficiencies in CeD on GFD compared to CeD not on GFD with corresponding risk of Bias. **A-** Vitamin 25 (OH) deficiency sub-grouped by duration of GFD **B-** Vitamin E deficiency sub-grouped by study design **C-** Vitamin K deficiency as controlled by triglycerides **D-** Ferritin deficiency **E-** Iron deficiency **F-** Zinc deficiency; Supplementary Figure S20: Forest plots comparison of micronutrient deficiencies in CeD on GFD compared to non-CeD controls with corresponding risk of Bias. **A-**Vitamin D deficiency **B-**Vitamin D deficiency stratified by study design **C-**Vitamin D deficiency stratified by duration of GFD length, **D-** Vitamin E deficiency in case-control study
