# Supplementary material for: Micronutrient Deficiencies Associated with a Gluten-Free Diet in Patients with Celiac Disease and Non-Celiac Gluten or Wheat Sensitivity: A Systematic Review and Meta-Analysis
Source: J Clin Med. 2025 Jul 8;14(14):4848. doi: 10.3390/jcm14144848 (PMC12296119; doi:10.3390/jcm14144848)
Supplement: Supplementary file 1 [file jcm-14-04848-s001.zip › jcm-3722504-supplementary.pdf]

Supplementary Material

Supplementary Table ST1- Search strategy terms

Studies were identified from the following databases: Cochrane Central Register of Controlled Trials (CENTRAL) (2000-), the Database of Abstracts of Reviews of Effectiveness (DARE) (1994-), MEDLINE (Ovid) (1946-), EMBASE (Ovid) (1974-), Web of Science (Ovid) (1900-), and CINAHL (1982-) and the gray literature (ex. conferences reports, technical reports, and dissertations) was searched using SIGLE (1982-) up to April 4, 2024. A registered Research Librarian at McMaster University was consulted to help develop appropriate and inclusive search strategy. Conference abstracts were included and recursive bibliography search was conducted where applicable at the discretion of the reviewers. Patients with wheat related disorders and CeD were identified with the medical subject heading and text term "celiac disease" "celiac sprue" "coeliac disease" "gluten sensitive enteropath*" or "CeD patients" and "wheat allergy" "wheat hypersensitiv*" "wheat senstivit*" "non-celiac gluten sensitivit*" "ncgs" and gluten free diet were identified by "gluten free diet" "gluten*" "celiac disease/Th (therapy)". All micronutrient deficiencies were searched with subject headings and within text (outlined in Supplementary Table 2).

Supplementary Table ST2- Search strategy used for EMBASE (Ovid) (1974- 04/2024)

1.	celiac disease/
2.	(celiac disease* or celiac sprue or coeliac disease* or gluten enteropathy* or gluten sensitive enteropath* or CeD patients).mp. [mp=title, abstract, heading word, drug trade name, original title, device manufacturer, drug manufacturer, device trade name, keyword, floating subheading word, candidate term word]
3.	wheat allergy/
4.	(wheat allerg* or wheat hypersensitiv* or wheat sensitivit* or non-celiac gluten sensitivit* or non-coeliac gluten sensitivit* or ncgs?ws).mp. [mp=title, abstract, heading word, drug trade name, original title, device manufacturer, drug manufacturer, device trade name, keyword, floating subheading word, candidate term word]
5.	or/1-4
6.	gluten free diet/
7.	(gluten-free diet* or gluten*).mp. [mp=title, abstract, heading word, drug trade name, original title, device manufacturer, drug manufacturer, device trade name, keyword, floating subheading word, candidate term word]
8.	or/6-7
9.	5 and 8
10.	celiac disease/th [Therapy]
11.	9 or 10
12.	malnutrition/
13.	vitamin b deficiency/ or vitamin deficiency/ or b12 deficiency/ or biotin deficiency/ or folic acid deficiency/ or nicotinic acid deficiency/ or riboflavin deficiency/ or thiamine deficiency/
14.	vitamin deficiency/ or ascorbic acid deficiency/ or retinol deficiency/ or vitamin b deficiency/ or vitamin d deficiency/ or vitamin k deficiency/
15.	choline deficiency/
16.	pyridoxine deficiency/
17.	pernicious anemia/
18.	osteomalacia/
19.	hypophosphatemia/
20.	alpha tocopherol deficiency/
21.	nicotinamide/
22.	nicotinic acid/
23.	calcium/
24.	hypocalcemia/ or calcium deficiency/
25.	magnesium deficiency/ or hypomagnesemia/
26.	folic acid/
27.	(Nutrition* deficienc* or hypophosphatemia or avitaminosis or biotin* or niacin or vitamin B3 or calcium or magnesium or ascorbic acid or vitamin a or retinol or vitamin b or choline or folic acid or riboflavin or thiamine or vitamin b?1 or vitamin b?6 or vitamin b?12 or anemi? or vitamin d or vitamin b?9 or cobalamin or vitamin e or vitamin k).mp. [mp=title, abstract, heading word, drug trade name, original title, device manufacturer, drug manufacturer, device trade name, keyword, floating subheading word, candidate term word]
28.	zinc/
29.	iron/
30.	selenium/
31.	selenium deficiency/
32.	zinc deficiency/
33.	iron deficiency/ or hypoferremia/
34.	chromium/
35.	chromium deficiency/
36.	trace element/
37.	nutritional status/
38.	(Zinc or zinc deficiency or iron or iron deficiency or hypoferremia or chromium or chromium deficiency or copper or copper deficiency or micronutrient* or nutritional status or trace element* or selenium or selenium deficiency).mp. [mp=title, abstract, heading word, drug trade name, original title, device manufacturer, drug manufacturer, device trade name, keyword, floating subheading word, candidate term word]
39.	copper deficiency/ or hypocupremia/
40.	copper/
41.	or/12-40
42.	11 and 41

Supplementary Table ST3- Equations used to estimate mean ($\overline{X}$) and standard deviation (σ) from Median (m) and either **(A)** minimum (a) and maximum (b) range[18] or **(B)** 1st and 3rd quartiles (q1/q3)[16,17]

(A)

$$\overline{X} = \frac{a + 2m + b}{4} \quad \text{and} \quad \sigma = \frac{b - a}{4}$$

(B)

$$\overline{X} = \frac{q1 + m + q3}{3} \quad \text{and} \quad \sigma = \frac{q3 - q1}{1.35}$$

Supplementary Table ST4- Studies excluded after full text review and primary reason for exclusion

Excluded Study	Year	Reason
Äärelä et al[1]	2016	Not intended outcome
Abdalla et al[2]	2017	Not intended intervention
Abu Daya et al[3]	2013	Not intended intervention
Adam et al[4]	2019	Not intended comparison
Ahlawat et al[5]	2019	Not intended intervention
Aksan et al[6]	2017	Not intended outcome
Al Hussani et al[7]	2024	Not intended intervention
Alzaben et al[8]	2016	Not intended outcome
Alzaben et al[9]	2015	Not intended outcome
Ãnal et al[10]	2012	Data not retrieved- abstract only*
Andrealli et al[11]	2012	Not intended comparison
Abdul-Aziz et al[12]	2017	Not intended intervention
Armagan et al[13]	2005	Not intended intervention
Baccini et al[14]	2006	Not intended intervention
Balaban et al[15]	2016	Not intended comparison
Barone et al[16]	2016	Not intended outcome
Bayrak et al[17]	2018	Conference abstract of included study
Bel'mer et al[18]	2014	Not intended outcome
Borovik et al[19]	2020	Not intended population
Bozbulut et al[20]	2019	Not intended outcome
Campagna et al[21]	2020	Not intended population
Cardigan et al[22]	2019	Not intended intervention
Carroccio et al[23]	1998	Not intended comparison
Carvalho et al[24]	2003	Data not available in article *
Casella et al[25]	2011	Conference Abstract- no poster available*
Chouliaras et al[26]	2011	Conference abstract of Margoni, 2012 (included)
Christhy et al[27]	2017	Not intended intervention
Cikrikcioglu et al[28]	2011	Not intended intervention
Corazza et al [29]	1996	Not intended outcome
Corazza et al[30]	1995	Repeat study
De Marchi et al[31]	2013	Not intended comparison
Delco, et al[32]	1999	Not intended outcome
Deora et al[33]	2017	Not intended comparison
Deora et al[34]	2016	Conference abstract- Repeat study
Dinu et al[35]	2017	Not intended comparison
Drabinska et al[36]	2018	Not intended comparison
Efthymakis et al[37]	2020	Conference abstract- poster not retrieved*
Efthymakis et al[38]	2015	Not intended comparison
El-Hodhod et al[39]	2012	Not intended intervention
El-Shaheed et al[40]	2019	Not intended intervention

Excluded Study	Year	Reason
El-Shaheed et al[41]	2018	Not intended intervention
Elli et al[42]	2018	Not intended comparison
Clinical trial EUCTR2019-003125- 21-FR[43]	2020	Clinical trial with data not available (ongoing)
Fallah et al.[44]	2024	Data not reported in article-not able to retrieve*
Fathi et al[45]	2013	Not intended intervention
Ferretti et al[46]	2003	Not intended comparison
Feurstein et al[47]	2016	Conference abstract- poster not retrieved*
Fickling et al[48]	2001	Not intended comparison
Francavilla et al[49]	2014	Not intended intervention
Geisel et al[50]	2012	Not intended outcome
Gutch et al[51]	2016	Not intended intervention
Haapalahti et al[52]	2005	Not intended intervention
Habel et al[53]	2017	Not intended intervention
Hadithi et al[54]	2010	Not intended intervention
Hallert et al[55]	2002	Not intended comparison
Hallert et al[56]	2009	Not intended comparison or intervention
Harper et al[57]	2007	Not intended intervention
Harper et al[58]	2007	Repeat study
Hassan, et al.[59]	2021	Full article not available- contacted publisher*
Henker & Gabsch[60]	1985	Repeat study
Hinks et al[61]	1984	Data not reported in article-not able to retrieve*
Hjelt & Krasilnikoff[62]	1990	Not intended comparison
Hjelt & Krasilnikoff[63]	1986	Not intended outcome
Hozyasz et al[64]	2003	Repeat
Huratdo[65]	2021	Not intended population
Idris Elzein & Elxein[66]	2019	Not intended intervention
Imam et al[67]	2014	Not intended intervention
Clinical Trial IRCT20170926036426N4[68]	2019	Clinical trial with data not available (ongoing)
Clinical Trial ISRCTN42241118[69]	2006	Not intended intervention
Jamnik et al[70]	2018	Not intended intervention
Jivraq et al[71]	2022	Not intended comparison
Kamal et al[72]	2018	Not intended population or comparison
Kapur et al[73]	2003	Not intended intervention
Kassim & Ross[74]	2018	Conference abstract poster not available*
Kemppainen et al[75]	1999	Not intended comparison
Kemppainen et al[76]	1998	Not intended comparison
Klimov et al[77]	2019	Not intended comparison
Krysiak et al[78]	2019	Not intended population
Kuryaninova et al[79]	2018	Conference abstract- full poster not available*
Lasta et al[80]	2014	Conference abstract- full poster not available*
Lee & Clarke[81]	2017	Not intended comparison
Lerner et al[82]	2012	Not intended intervention
Lheure et al[83]	2017	Not intended comparison
Li et al [84]	2023	Not intended comparison

Excluded Study	Year	Reason
Love et al[85]	2018	Not intended comparison
Lu et al[86]	2021	Systematic Review
Lucendo et al[87]	2011	Not intended comparison
Lucendo et al[88]	2011	Not intended comparison
Mackinder et al[89]	2015	Not intended comparison
Mager, et al[90]	2011	Not intended comparison
Marild et al[91]	2017	Not intended intervention
Martinon-Torres et al[92]	2019	Not intended comparison and intervention
Masood & Shaikh[93]	2014	Not intended intervention
Mazza et al[94]	2019	Not intended population
McFarlane et al[95]	1995	Not intended comparison
Mishra et al[96]	2019	Not intended comparison
Mitchell & Robinson[97]	2002	Not intended comparison
Molteni et al[98]	1995	Not intended comparison
Moreno et al[99]	2018	Not intended comparison
Murray et al[100]	2013	Not intended comparison or intervention
Muzzo et al[101]	2000	Not intended outcome
Clinical Trial NCT03274648[102]	2017	Not intended population
Negi et al[103]	2018	Not intended intervention
O'Leary et al[104]	2004	Not intended comparison
O'Morain et al[105]	2021	Not intended comparison
Packer et al[106]	1978	Not intended comparison
Pham-Short et al[107]	2019	Not intended population
Piatek-Guziewicz et al[108]	2017	Repeat study
Piran Arce et al[109]	2017	Not intended comparison
Piran Arce et al[109]	2017	Repeat study
Quero et al[110]	2015	Not intended comparison
Radzikowski et al[111]	1989	Not intended comparison
Radzikowski et al[111]	1989	Repeat study
Rajalahti et al[112]	2017	Not intended comparison
Rajalahti et al[113]	2016	Conference abstract of study Rajalahti et al[103]
Rajalahti et al[114]	2017	Repeat study
Rawal et al[115]	2010	Repeat study of Negi et al[95]
Rea et al[116]	1998	Not intended comparison
Rea et al[117]	1996	Not intended comparison
Romanczuk et al[118]	2016	Repeat study
Rujner et al[119]	2004	Not intended intervention
Rujner et al[120]	2001	Repeat study
Rujner et al[121]	2001	Repeat study
Saibeni et al[122]	2005	Not intended comparison
Salazar Quero et al[101]	2015	Repeat study of Muzzo et al[101]
Sategna-Guidetti et al[123]	2000	Not intended comparison
Schosler, et al[124]	2016	Not intended comparison
Scott & Losowsky[125]	1976	Data not available in main text*

**Excluded Study**	**Year**	**Reason**
Scricciolo et al[126]	2018	Not intended comparison or population
Sdepanian et al[127]	2003	Data not available in main text*
Sdepanian, et al.[128]	2004	Conference abstract no poster available*
Selby et al[129]	1999	Not intended comparison
Setty-Shah et al[130]	2014	Not intended population
Sgaramella et al[131]	2020	Not intended intervention
Shahriari et al[132]	2018	Not intended population
Sharma et al[133]	2013	Not intended outcome
Shaveta, et al.[134]	2011	Not intended comparison
Shelley, et al.[135]	2016	Not intended comparison
Siddiqui et al. [136]	2023	Not intended outcome
Simmons et al[137]	2011	Not intended population
Soliman et al[138]	2019	Not intended comparison
Soliman et al[139]	2019	Conference abstract of Soliman et al[126]
Solomons, et al.[140]	1976	Not intended outcome
Stahlberg, et al. [141]	1991	Not intended comparison
Stein et al[142]	2015	Not intended intervention
Szathmari et al[143]	1997	Not intended comparison
Taylor et al[144]	2020	Not intended outcome
Tetzlaff et al[145]	2020	Conference abstract- full poster not available*
Thong et al[146]	2018	Not intended population
Tolone et al[147]	2019	Conference abstract-full poster not available*
Tran et al[148]	2011	Not intended comparison
Tuna Kirsaclioglu et al[149]	2016	Not intended comparison
Unalp-Arida et al[150]	2016	Not intended intervention
Van Megen et al[151]	2023	Not intended comparison
Varkonyi et al[152]	1992	Not intended outcome
Ventura et al[153]	2023	Data not available in main text*
Villanueva, et al.[154]	2012	Not intended intervention
Vilppula et al[155]	2011	Not intended comparison
Volkan, et al.[156]	2017	Not intended comparison
Volkan, et al.[157]	2016	Conference abstract of Volkan et al (included)
Volkan et al[158]	2016	Conference abstract of Bayrak et al[31]
Weintraub et al[159]	2017	Not intended intervention
Weir & Hourihane[160]	1974	Not intended comparison
Wierdsma et al[161]	2013	Not intended intervention
Wright et al[162]	2017	Not intended comparison
Yuce et al[163]	2004	Not intended intervention

*Multiple attempts were made to contact authors for data retrieval

References for excluded studies

1. Aarela L, Nurminen S, Kivela L, et al. Prevalence and associated factors of abnormal liver values in children with celiac disease. Dig Liver Dis 2016;48:1023-9.
2. Abdalla A, Saifullah SM, Osman M, et al. Prevalence of occult celiac disease in females with iron deficiency in the United States: an NHANES analysis. J Community Hosp Intern Med Perspect 2017;7:347-50.
3. Abu Daya H, Lebwohl B, Lewis SK, Green PH. Celiac disease patients presenting with anemia have more severe disease than those presenting with diarrhea. Clin Gastroenterol Hepatol 2013;11:1472-7.
4. Adam UU, Melgies M, Kadir S, Henriksen L, Lynch D. Coeliac disease in Caucasian and South Asian patients in the North West of England. J Hum Nutr Diet 2019;32:525-30.
5. Ahlawat R, Weinstein T, Markowitz J, Kohn N, Pettei MJ. Should We Assess Vitamin D Status in Pediatric Patients With Celiac Disease? J Pediatr Gastroenterol Nutr 2019;69:449-54.
6. Aksan A, Aksan S, Hauser W, Stein J, editors. P2456 - First Results From the Celiac Disease Health-Related Quality of Life Questionnaire in Turkey: Potential Impact of Nutrition Status on Health-Related Quality of Life in Adult Patients With Celiac Disease. 82nd Annual Scientific Meeting of the American College of Gastroenterology; 2017; United States: Am J Gastroenterol.
7. Al-Hussani, Troncone R, Alobaid S, Bashir MS. Status of vitamins and minerals in children with screening-identified celiac disease: A case-control study. J Pediatr Gastroenterol Nutr; 2024; 78; 677-684
8. Alzaben AS, Mager D, Marcon MA, et al., editors. Diet quality and quality of life in children and adolescents with celiac disease. World Congress of Pediatric Gastroenterology, Hepatology and Nutrition; 2016; Montreal, QC Canada: Journal of Pediatric Gastroenterology and Nutrition.
9. Alzaben AS, Turner J, Shirton L, et al. Assessing Nutritional Quality and Adherence to the Gluten-free Diet in Children and Adolescents with Celiac Disease. Can J Diet Pract Res 2015;76:56-63.
10. Ãnal F, Umman C, Cebe Tok A, et al. Plasma selenium levels in celiac disease pateints on a gluten-free diet. Journal of Current Pediatrics 2012;10:55-8.
11. Andrealli A, Luchino M, Ribaldone D, et al. Evaluation of bone metabolism at diagnosis of celiac disease and during follow-up. 18th National Congress of Digestive Diseases Italian Federation of Societies of Digestive Diseases- FISMAD 2012 March 2012; Naples, Italy: Elsevier; 2012. p. S159-S60.
12. Aziz DA, Kahlid M, Memon F, Sadiq K. Spectrum of Celiac disease in Paediatric population: Experience of Tertiary Care Center from Pakistan. Pak J Med Sci 2017;33:1301-6.
13. Armagan O, Uz T, Tascioglu F, et al. Serological screening for celiac disease in premenopausal women with idiopathic osteoporosis. Clin Rheumatol 2005;24:239-43.
14. Baccini F, Spiriti MA, Vannella L, et al. Unawareness of gastrointestinal symptomatology in adult coeliac patients with unexplained iron-deficiency anaemia presentation. Alimentary pharmacology & therapeutics 2006;23:915-21.
15. Balaban DV, Popp A, Robu G, et al., editors. Nutritional deficiencies in adult patients with celiac disease. XXXVI National Congress of Gastroenterology, Hepatology and Digestive Endoscopy; 2016; Cluj-Napoca, Romania.

16. Barone M, Della Valle N, Rosania R, et al. A comparison of the nutritional status between adult celiac patients on a long-term, strictly gluten-free diet and healthy subjects. Eur J Clin Nutr 2016;70:23-7.
17. Bayrak NA, Volkan B, Haliloglu B, Kara SS, Cayir A. Pubertal development in children with celiac disease: one year follow up. Journal of Pediatric Gastroenterology and Nutrition; 2018. p. S393-S4.
18. Bel'mer SV, Mitina EV, Karpina LM, Smetanina NS. [Iron deficiency anemia and anemia in chronic celiac disease in children]. Eksp Klin Gastroenterol 2014;23-9.
19. Borovik TE, Roslavtseva EA, Fisenko AP, et al. Celiac disease in children with skin pathology: A multidisciplinary problem. Pediatriya- Zhurnal Im G N Speranskogo 2020;99:255-64.
20. Bozbulut NE, Bozbulut R, Koksal E, Dalgic B, editors. Determination of growth and dietary adequacy status for Celiac disease. 52nd Annual Meeting of the European Society of Paediatric Gastroneterology Hepatology and Nutriiton, ESPGHAN 2019; 2019 May 2019; United Kingdom: Journal of Pediatric Gastroenterology and Nutrition.
21. Campagna G, Tatangelo R, La Fratta I, et al. Insights in the Evaluation of Gluten Dietary Avoidance in Healthy Subjects. J Am Coll Nutr 2020;39:178-86.
22. Cardigan T, Buchanan E, Duncan H, et al., editors. Outcomes of high dose of vitamin D in children with vitamin D deficiency and coeliac disease. 52nd Annual Meeting of the European Society of Paediatric Gastroenterology Hepatology and Nutrition, ESPGHAN 2019; 2019 May 2019; United Kingdom: Journal of Pediatric Gastroenterology and Nutrition.
23. Carroccio A, Iannitto E, Cavataio F, et al. Sideropenic anemia and celiac disease: one study, two points of view. Dig Dis Sci 1998;43:673-8.
24. Carvalho CN, Sdepanian VL, de Morais MB, Fagundes Neto U. [Celiac disease under treatment: evaluation of bone mineral density]. J Pediatr (Rio J) 2003;79:303-8.
25. Casella S, Zanini B, Lanzarotto F, et al. Cognitive impairment during gluten free diet (GFD) in elderly coeliac patients. Digestive Disease Week (DDW); Chicago, IL United States: Gastroenterology; 2011. p. S440.
26. Chouliaras G, Margoni D, Duscas G, et al. Bone health in children with celiac disease assessed by dual x-ray absorptiometry: Effect of gluten-free diet and predictive value of serum biochemical indices. European Society for Paediatric Gastroenterology, Hepatology, and Nutrition Annual Meeting 2011; Sorrento Italy: Journal of Pediatric Gastroenterology and Nutrition; 2011. p. E129-E30.
27. Christhy SM, Singh N. Nutritional staus of celiac and non-celiac children from Rajasthan, India. Nutrition & Food Science 2017;47:240-53.
28. Cikrikcioglu MA, Halac G, Hursitoglu M, et al. Prevalence of gluten sensitive enteropathy antibodies in restless legs syndrome. Acta Neurol Belg 2011;111:282-6.
29. Corazza GR, Di Sario A, Cecchetti L, et al. Influence of pattern of clinical presentation and of gluten-free diet on bone mass and metabolism in adult coeliac disease. Bone 1996;18:525-30.
30. Corazza GR, Di Sario A, Cecchetti L, et al. Bone mass and metabolism in patients with celiac disease. Gastroenterology 1995;109:122-8.
31. De Marchi S, Chiarioni G, Prior M, Arosio E. Young adults with coeliac disease may be at increased risk of early atherosclerosis. Alimentary pharmacology & therapeutics 2013;38:162-9.

32. Delco F, El-Serag HB, Sonnenberg A. Celiac sprue among US military veterans: associated disorders and clinical manifestations. Dig Dis Sci 1999;44:966-72.
33. Deora V, Aylward N, Sokoro A, El-Matary W. Serum Vitamins and Minerals at Diagnosis and Follow-up in Children With Celiac Disease. J Pediatr Gastroenterol Nutr 2017;65:185-9.
34. Deora V, El-Matary W, Aylward N, Sokoro AR. Micronutrient deficiences at diagnosis in children newly diagnosed with celiac disease. World Congress of Pediatric Gastroenterology, Hepatology and Nutrition 2016; Canada: Journal of Pediatric Gastroenterology and Nutrition; 2016. p. S313.
35. Dinu M, Macchia D, Pagliai G, et al. Symptomatic efficacy of buckwheat products in Non-Celiac Gluten Sensitivity (NCGS). Asia Pac J Clin Nutr 2017;26:630-6.
36. Drabinska N, Krupa-Kozak U, Abramowicz P, Jarocka-Cyrta E. Beneficial Effect of Oligofructose-Enriched Inulin on Vitamin D and E Status in Children with Celiac Disease on a Long-Term Gluten-Free Diet: A Preliminary Randomized, Placebo-Controlled Nutritional Intervention Study. Nutrients 2018;10:
37. Efthymakis K, De Felicibus E, Laterza F, et al. Prevalence of anemia and iron deficiency in celiac disease after effective gluten exclusion. 26th National Congress of Digestive Diseases Extraordinary edition; Virtual, Online: Digestive and Liver Disease; 2020. p. S91-S2.
38. Efthymakis K, Milano A, Laterza F, Serio M, Neri M. Iron deficiency anemia despite effective gluten-free diet in celiac disease: Diagnostic role of small bowel capsule endoscopy. Dig Liver Dis 2017;49:412-6.
39. El-Hodhod MA, El-Agouza IA, Abdel-Al H, Kabil NS, Bayomi KA. Screening for celiac disease in children with dental enamel defects. ISRN Pediatr 2012;2012:763783.
40. Shaheed AAE, Arab AEE, Kassas GME, et al. An innovative effective nutritional therapy for vitamin D deficiency in children with celiac disease. Biomed Pharmacol J 2019;12:
41. Abd El-Shaheed A, El-Arab AE, Abou-Zekri M, et al. A novel gluten-free meal as a nutritional therapy for Iron deficiency anemia in children with celiac disease. Bioscience Research 2018;15:207-14.
42. Elli L, Ferretti F, Branchi F, et al. Sucrosomial Iron Supplementation in Anemic Patients with Celiac Disease Not Tolerating Oral Ferrous Sulfate: A Prospective Study. Nutrients 2018;10:330.
43. EUCTR2019-003125-21-FR. EFFECT OF INTRAVENOUS IRON SUPPLEMENTATION ON CELIAC DISEASE REMISSION IN PATIENTS WITH IRON DEFICIENCY AND INTESTINAL VILLOUS ATROPHY: a RANDOMIZED TRIAL: WHO International Clinical Trials Registry Program; 2020 [Available from: http://www.who.int/trialsearch/Trial2.aspx?TrialID=EUCTR2019-003125-21-FR. Accessed on November 16, 2020
44. Fallah S, Asri N, Nikzamir A, Ahmadipour S, Sadeghi A, Rostami K. Rostami-Nejad M. Investigating the Impact of Vitamin A and Amino Acids on Immune Responses in Celiac Disease Patients. Diseases; 2024; 12; 10.3390/diseases12010013
45. Fathi F, Ektefa F, Tafazzoli M, et al. The concentration of serum zinc in celiac patients compared to healthy subjects in Tehran. Gastroenterol Hepatol Bed Bench 2013;6:92-5.
46. Ferretti J, Mazure R, Tanoue P, et al. Analysis of the structure and strength of bones in celiac disease patients. The American journal of gastroenterology 2003;98:382-90.
47. Feurstein J, Kogler H, Innerhofer A, et al., editors. Micronutrients in children with coeliac disease. 54th Annual meeting of the Austrian Society for Pediatrics and Adolescent Medicine; 2016; Feldkirch, Austria.

48. Fickling WE, McFarlane XA, Bhalla AK, Robertson DA. The clinical impact of metabolic bone disease in coeliac disease. Postgrad Med J 2001;77:33-6.
49. Francavilla R, Cristofori F, Castellaneta S, et al. Clinical, serologic, and histologic features of gluten sensitivity in children. J Pediatr 2014;164:463-7 e1.
50. Martin J, Geisel T, Maresch C, Krieger K, Stein J. Inadequate nutrient intake in patients with celiac disease: results from a German dietary survey. Digestion 2013;87:240-6.
51. Gutch M, Avinash A, Sukriti K, et al. Impact of gluten-free diet on several growth parameters in children with type 1 diabetes melitus and celiac disease in western Uttar Pradesh, India. Journal of the ASEAN Federation of Endocrine Societies 2016;31:5-9.
52. Haapalahti M, Kulmala P, Karttunen TJ, et al. Nutritional status in adolescents and young adults with screen-detected celiac disease. J Pediatr Gastroenterol Nutr 2005;40:566-70.
53. Habel L, Frankenfeld CL, Gallo S, Moshfegh AJ, Slavin M. Differences in nutritional intake and bone health among adults with and without celiac disease: Data from the national health and nutrition examination survey, 2009-2012. Journal of American College of Nutrition 2019;39:112-21.
54. Hadithi M, Mulder CJ, Stam F, et al. Effect of B vitamin supplementation on plasma homocysteine levels in celiac disease. World J Gastroenterol 2009;15:955-60.
55. Hallert C, Grant C, Grehn S, et al. Evidence of poor vitamin status in coeliac patients on a gluten-free diet for 10 years. Alimentary pharmacology & therapeutics 2002;16:1333-9.
56. Hallert C, Svensson M, Tholstrup J, Hultberg B. Clinical trial: B vitamins improve health in patients with coeliac disease living on a gluten-free diet. Alimentary pharmacology & therapeutics 2009;29:811-6.
57. Harper JW, Holleran SF, Ramakrishnan R, Bhagat G, Green PH. Anemia in celiac disease is multifactorial in etiology. Am J Hematol 2007;82:996-1000.
58. Hassan RARA, Al-Attabi MRS, Abdul-Redha MA. Evaluation of some serum micronutrients in patients with celiac diesase in Wasit Province, Iraq. Biochemical and Cellular Archives 2021;21:1111-7.
59. Henker J, Gabsch HC. [Serum zinc levels in children with celiac disease]. Helv Paediatr Acta 1985;40:47-53.
60. Hinks LJ, Inwards KD, Lloyd B, Clayton BE. Body content of selenium in coeliac disease. Br Med J (Clin Res Ed) 1984;288:1862-3.
61. Hjelt K, Krasilnikoff PA. The impact of gluten on haematological status, dietary intakes of haemopoietic nutrients and vitamin B12 and folic acid absorption in children with coeliac disease. Acta Paediatr Scand 1990;79:911-9.
62. Hjelt K, Krasilnikoff PA. Vitamin B12 absorption capacity in healthy children. J Pediatr Gastroenterol Nutr 1986;5:274-7.
63. Hozyasz KK, Chelchowska M, Laskowska-Klita T. [Vitamin E levels in patients with celiac disease]. Med Wieku Rozwoj 2003;7:593-604.
64. A MDH, Cifuentes L, Al-Ward R, et al. Post-bariatric Surgery Outcomes and Complications in Patients with Celiac Disease: a Matched Case-Control Study. Obes Surg 2021;31:4405-18.
65. Idris H, Elzein AM, Elxein HO. Evaluation of zinc and copper levels in sudanese patients with celiac disease in red sea state. Pakistan Journal of Medical and Health Sciences 2019;13:1120-3.
66. Imam MH, Ghazzawi Y, Murray JA, Absah I. Is it necessary to assess for fat-soluble vitamin deficiencies in pediatric patients with newly diagnosed celiac disease? J Pediatr Gastroenterol Nutr 2014;59:225-8.

67. IRCT20170926036426N4. The effect of interaction education on patients with celiac disease: WHO International Clinical Trials Registry Program; 2019 [Available from: http://www.who.int/trialsearch/Trial2.aspx?TrialID=IRCT20170926036426N4. Accessed on November 16, 2020
68. ISRCTN42241118. Vitamin supplementation in adult coeliac disease patients: WHO International Clinical Trials Registry Program; 2006 [Available from: https://trialsearch.who.int/?TrialID=ISRCTN42241118. Accessed on November 16, 2020
69. Jamnik J, Jenkins DJ, El-Sohemy A. Biomarkers of cardiometabolic health and nutritional status in individuals with positive celiac disease serology. Nutr Health 2018;24:37-45.
70. Jivraj A, Hutchinson JM, Ching E, Marwaha A, Verdu EF, Armstrong D, Pinto-Sanchez MI. Micronutrient deficiences are frequent in adult patients with and without celiac disease on a gluten-free diet, regardless of duration and adherence to the diet. Nutrition; 2022; 103-104;111809
71. Kamal S, Aldossari KK, Ghoraba D, et al. Clinicopathological and immunological characteristics and outcome of concomitant coeliac disease and non-alcoholic fatty liver disease in adults: a large prospective longitudinal study. BMJ Open Gastroenterol 2018;5:e000150.
72. Kapur G, Patwari AK, Narayan S, Anand VK. Iron supplementation in children with celiac disease. Indian J Pediatr 2003;70:955-8.
73. Kassim M, Ross S, editors. Vitamin D monitoring in coeliac disease- where do we start? Royal College of Paediatrics and Child Health Annual Conference, RCPCH 2018; 2018; United Kingdom: Archives of Disease in Childhood.
74. Kemppainen T, Kroger H, Janatuinen E, et al. Bone recovery after a gluten-free diet: a 5-year follow-up study. Bone 1999;25:355-60.
75. Kemppainen TA, Kosma VM, Janatuinen EK, et al. Nutritional status of newly diagnosed celiac disease patients before and after the institution of a celiac disease diet--association with the grade of mucosal villous atrophy. Am J Clin Nutr 1998;67:482-7.
76. Klimov L, Abramskaya L, Zakharova I, et al. Level of Vitamins D, parathormone, bone tissue metabolites in children with coeliac disease and bone fractures. 52nd Annual Meeting of the European Society for Paediatric Gastroenterology Hepatology and Nutrition, ESPGHAN 2019; United Kingdom: Journal of Pediatric Gastroenterology and Nutrition; 2019. p. 158.
77. Krysiak R, Szkrobka W, Okopien B. The Effect of Gluten-Free Diet on Thyroid Autoimmunity in Drug-Naive Women with Hashimoto's Thyroiditis: A Pilot Study. Exp Clin Endocrinol Diabetes 2019;127:417-22.
78. Kuryaninova V, Stoyan M, Klimov L, Zakharova I, Zavyalova E. Dynamics of iron-deficient conditions in children with celiac disease on the background of gluten-free diet. 51st Annual Meeting European Society for Paediatric Gastroenterology, Hepatology and Nutrition, ESPGHAN 2018; Switzerland: Journal of Pediatiric Gastroenterology and Nutrition; 2018. p. 209.
79. Lasta LM, Gentili MP, Capiel C, et al. Effect of gluten-free diet on bone mineral density in children and adolescents with celiac disease. 13th International Congress of Pediatric Laboratory Medicine, ICPLM 2014; Instanbul, Turkey: Clinical Biochemistry; 2014. p. 775.
80. Lee J, Clarke K. Clinical parameters and quality of life in patients with celiac disease using CD-QOL scores (Celiac disease-specific assessment instrument for quality of life). Scand J Gastroenterol 2017;52:1235-9.

81. Lerner A, Shapira Y, Agmon-Levin N, et al. The clinical significance of 25OH-Vitamin D status in celiac disease. Clin Rev Allergy Immunol 2012;42:322-30.
82. Lheure C, Ingen-Housz-Oro S, Guignard S, et al. Dermatitis herpetiformis and bone mineral density: analysis of a French cohort of 53 patients. Eur J Dermatol 2017;27:353-8.
83. Li Z, Zhang L, Li L, Du Z. Evaluation of serum levels of copper and zinc in patients with celiac disease seropositivity:Findings from the national healthy and nutrition examination survey. Biol Trace Elem Res; 2023; 201; 683-688
84. Love A, Silvester JA, Rigaux L, et al. Prevalence of nutritional deficienies in follow-up of Adults diagnosed with celiac disease: A Prospective Study. DDW 2018- Digestive Disease Week; United States: Gastroenterology; 2018. p. S667-S78.
85. Lu C, Zhou W, He X, Zhou X, Yu C. Vitamin D status and vitamin D receptor genotypes in celiac disease: a meta-analysis. Crit Rev Food Sci Nutr 2021;61:2098-106.
86. Lucendo A, Garcia Manzanares A, Tenias-Burillo J, et al. Does gluten-free diet resolve vitamin and mineral deficiencies associated to onset celiac disease? Do patients need supplementation? 11th European Nutrition Conference, FENS 2011; Madrid, Spain: Annals of Nutrition and Metabolism; 2011. p. 67.
87. Lucendo A, Garcia-Manzanares A, Conde Garcia M, et al. Nutritional status and determining factors in adult-onset celiac disease. 11th European Nutrition Conference, FENS 2011; Madrid Spain: Annals of Nutrition and Metabolism; 2011. p. 2.
88. Mackinder M, Wong SC, Tsiountsioura M, et al. Children with coeliac disease on gluten free diet have normal bone mass, geometry and muscle mass. 54th Annual Meeting of the European Society for Paediatric Endocrinology, ESPE 2015; Barcelona, Spain: Hormone Research in Paediatrics; 2015. p. 155-6.
89. Mager DR, Qiao J, Turner J. Vitamin D and K status influences bone mineral density and bone accrual in children and adolescents with celiac disease. Eur J Clin Nutr 2012;66:488-95.
90. Marild K, Tapia G, Haugen M, et al. Maternal and neonatal vitamin D status, genotype and childhood celiac disease. PLoS One 2017;12:e0179080.
91. Martinon-Torres N, Curjeiras-Martinez V, De Lamas-Perez C, Vazquez-Cobela R, Leis-Trabazo MR. Quantifying the risk of overnutrition: Nutritional status in coeliac disease at diagnosis and after a five year follow-up in a tertiary hospital. 52nd Annual Meeting of the European Society for Paediatric Gastroenterology Hepatology and Nutrition, ESPGHAN 2019; United Kingdom: Journal of Pediatric Gastroenterology and Nutrition; 2019. p. 165.
92. Masood N, Ali Shaikh I. Clinical presentations and biochemical profile in adult celiac disease patients in Hyderabad: Pakistan. Pak J Med Sci 2014;30:287-90.
93. Mazza GA, Marrazzo S, Gangemi P, et al. Oral iron absorption test with ferrous bisglycinate chelate in children with celiac disease. Minerva Pediatr 2019;71:139-43.
94. McFarlane XA, Marsham J, Reeves D, Bhalla AK, Robertson DAF. Subclinical nutrional deficiency in treated coeliac disease and nutritional content of the gluten free diet. Journal of Human Nutrition and Dietetics 1995;8:231-7.
95. Mishra A, Sharma M, Sarma P, et al. Etiology of anemia in patients with celiac disease. Asia Pacific Digestive Week, APDW 2019; India: Journal of Gastroenterology and Hepatology; 2019. p. 641.
96. Mitchell RM, Robinson TJ. Monitoring dietary compliance in coeliac disease using red cell distribution width. Int J Clin Pract 2002;56:249-50.

97. Molteni N, Bardella MT, Vezzoli G, Pozzoli E, Bianchi P. Intestinal calcium absorption as shown by stable strontium test in celiac disease before and after gluten-free diet. The American journal of gastroenterology 1995;90:2025-8.
98. Moreno ML, Vazquez H, Sugai E, et al. Serum immunological mediators are associated with anemia in patients with celiac disease. A prospective follow-up assessment. DDW 2018 - Digestive Disease Week; United States: Gastroenterology; 2018. p. S-681.
99. Murray JA, McLachlan S, Adams PC, et al. Association between celiac disease and iron deficiency in Caucasians, but not non-Caucasians. Clin Gastroenterol Hepatol 2013;11:808-14.
100. Muzzo S, Burrows R, Burgueo M, et al. Effect of calcium and vitamin D supplementation on bone mineral density of celiac children. Nutrition Research 2000;20:1241-7.
101. NCT03274648. Dietary Interventions and Butyrate Production in Behçet's Patients: NIH US National Library of Medicine; 2017 [Available from: https://clinicaltrials.gov/ct2/show/NCT03274648. Accessed on November 16, 2020
102. Negi K, Kumar R, Sharma L, et al. Serum zinc, copper and iron status of children with coeliac disease on three months of gluten-free diet with or without four weeks of zinc supplements: a randomised controlled trial. Trop Doct 2018;48:112-6.
103. O'Leary C, Wieneke P, Healy M, et al. Celiac disease and the transition from childhood to adulthood: a 28-year follow-up. The American journal of gastroenterology 2004;99:2437-41.
104. O'Morain N, Shannon E, McManus J, et al. Coeliac disease enteropathy and symptoms may be aggravated by angiotensin receptor blockers in patients on a gluten-free diet. United European Gastroenterol J 2021;9:973-9.
105. Packer SM, Charlton V, Keeling JW, et al. Gluten challenge in treated coeliac disease. Arch Dis Child 1978;53:449-55.
106. Pham-Short A, Donaghue KC, Ambler G, et al. Abnormal Cortical and Trabecular Bone in Youth With Type 1 Diabetes and Celiac Disease. Diabetes Care 2019;42:1489-95.
107. Piatek-Guziewicz A, Ptak-Belowska A, Przybylska-Felus M, et al. Intestinal parameters of oxidative imbalance in celiac adults with extraintestinal manifestations. World J Gastroenterol 2017;23:7849-62.
108. Piran Arce MF, Aballay LR, Leporati JL, Navarro A, Forneris M. Blood iron levels in accordance with adherence to a gluten-free diet in celiac school aged children. Nutr Hosp 2017;35:25-32.
109. Salazar Quero JC, Espín Jaime B, Rodríguez Martínes A, et al. Nutritional assessment of gluten-free diet. Is gluten-free diet deficient in some nutrient? An Pediatr (Barc) 2015;83:33-9.
110. Radzikowski A, Wojnar M, Kulus M, Zalewski T. [Evaluation of the effect of gluten-free diet on nutritional status of children with florid celiac disease]. Pediatr Pol 1989;64:150-4.
111. Rajalahti T, Repo M, Kivela L, et al. Anemia in Pediatric Celiac Disease: Association With Clinical and Histological Features and Response to Gluten-free Diet. J Pediatr Gastroenterol Nutr 2017;64:e1-e6.
112. Rajalahti T, Repo M, Kivela L, et al. Anaemia in children with celiac disease: Association with the clinical, serological and histological findings and response to the gluten-free diet. 49th Annual Meeting of the European Society for Paediatric Gastroenterology, Hepatology and Nutrition, ESPGHAN 2016; Athens, Greece: Journal of Pediatric Gastroenterology and Nutrition; 2016. p. 219.

113. Rawal P, Thapa BR, Prasad R, et al. Zinc supplementation to patients with celiac disease--is it required? J Trop Pediatr 2010;56:391-7.

114. Rea F, Polito C, Iovene A, et al. Effect of gluten-free diet on bone mineral metabolism of celiac children. Nutrition Research 1998;18:1661-6.

115. Rea F, Polito C, Marotta A, et al. Restoration of body composition in celiac children after one year of gluten-free diet. J Pediatr Gastroenterol Nutr 1996;23:408-12.

116. Romanczuk B, Szaflarska-Poplawska A, Chelchowska M, Hozyasz KK. Analysis of the concentration of vitamin E in erythrocytes of patients with celiac disease. Prz Gastroenterol 2016;11:282-5.

117. Rujner J, Socha J, Syczewska M, et al. Magnesium status in children and adolescents with coeliac disease without malabsorption symptoms. Clin Nutr 2004;23:1074-9.

118. Rujner J, Socha J, Wojtasik A, et al. [Magnesium status in children and adolescents with celiac disease]. Wiad Lek 2001;54:277-85.

119. Rujner J, Wojtasik A, Syczewska M, et al. [Reasons for magnesium deficiency in children with coeliac disease]. Wiad Lek 2001;54:522-31.

120. Saibeni S, Lecchi A, Meucci G, et al. Prevalence of hyperhomocysteinemia in adult gluten-sensitive enteropathy at diagnosis: role of B12, folate, and genetics. Clin Gastroenterol Hepatol 2005;3:574-80.

121. Sategna-Guidetti C, Grosso SB, Grosso S, et al. The effects of 1-year gluten withdrawal on bone mass, bone metabolism and nutritional status in newly-diagnosed adult coeliac disease patients. Alimentary pharmacology & therapeutics 2000;14:35-43.

122. Schosler L, Christensen LA, Hvas CL. Symptoms and findings in adult-onset celiac disease in a historical Danish patient cohort. Scand J Gastroenterol 2016;51:288-94.

123. Scott BB, Losowsky MS. Depressed cell-mediated immunity in coeliac disease. Gut 1976;17:900-5.

124. Scricciolo A, Ferretti F, Roncoroni L, et al. Effects of a dietary approach to iron deficiency in women affected by celiac disease. 24th National Congress of Digestive Diseases; Italy: Digestive and Liver Disease; 2018. p. e235-e6.

125. Sdepanian VL, de Miranda Carvalho CN, de Morais MB, Colugnati FA, Fagundes-Neto U. Bone mineral density of the lumbar spine in children and adolescents with celiac disease on a gluten-free diet in Sao Paulo, Brazil. J Pediatr Gastroenterol Nutr 2003;37:571-6.

126. Sdepanian VL, Morais MB, Fagundes Neto U. Dietary transgression in patients with celiac disease as a risk factor for iron deficiency. Journal of Pediatric Gastroenterology and Nutrition 2004;39:S209-S10.

127. Selby PL, Davies M, Adams JE, Mawer EB. Bone loss in celiac disease is related to secondary hyperparathyroidism. J Bone Miner Res 1999;14:652-7.

128. Setty-Shah N, Maranda L, Nwosu BU. Increased risk for vitamin d deficiency in obese children with both celiac disease and type 1 diabetes. Gastroenterol Res Pract 2014;2014:561351.

129. Barera G, Maruca K, Sgaramella P, Di Stefano M, Mora S. Short-term, low dose vitamin D supplementation in young patients with celiac disease: a pilot study. Eur J Gastroenterol Hepatol 2020;32:663-4.

130. Shahriari M, Honar N, Yousefi A, Javaherizadeh H. Association of Potential Celiac Disease and Refractory Iron Deficiency Anemia in Children and Adolescents. Arq Gastroenterol 2018;55:78-81.

131. Sharma M, Singh P, Agnihotri A, et al. Celiac disease: a disease with varied manifestations in adults and adolescents. J Dig Dis 2013;14:518-25.
132. Shaveta B, Vandana M, Sandeep P. Impact of gluten free diet on health and nutritional status of adults with celiac disease. Asian Pacific Disgestive Week 2011; Singapore, Singapore: Journal of Gastroenterology and Hepatology; 2011. p. 196.
133. Shelley L, Jacobs A, Daveson AJM. Nutrient deficiencies are common at the time of diagnosing coeliac disease in an Austrailian cohort. Australian Gastroenterology Week 2016; Australia: Journal of Gastroenterology and Heptaology (Australia); 2016. p. 164.
134. Siddiqui F, Uqaili AA, Memon S, Shah T, Shaikh SN, Memon AR. Association of serum albumin, globin, and transferrin levels in children of poorly managed celiac disease. Bio Med Res Int 2023; 5081303
135. Simmons JH, Klingensmith GJ, McFann K, et al. Celiac autoimmunity in children with type 1 diabetes: a two-year follow-up. J Pediatr 2011;158:276-81 e1.
136. Soliman AT, Laham M, Jour C, et al. Linear growth of children with celiac disease after the first two years on gluten- free diet: a controlled study. Acta Biomed 2019;90:20-7.
137. Soliman AT, Laham M, Jour C, et al. Linear growth of children with celiac disease after the first two years on gluten- free diet: a controlled study. 58th Annual Meeting of the European Society for Paediatric Endocrinology, ESPE 2019; Austria: Hormone Research in Paediatrics; 2019. p. 451.
138. Solomons NW, Rosenberg IH, Sandstead HH. Zinc nutrition in celiac sprue. Am J Clin Nutr 1976;29:371-5.
139. Stahlberg MR, Savilahti E, Siimes MA. Iron deficiency in coeliac disease is mild and it is detected and corrected by gluten-free diet. Acta Paediatr Scand 1991;80:190-3.
140. Stein EM, Rogers H, Leib A, et al. Abnormal Skeletal Strength and Microarchitecture in Women With Celiac Disease. J Clin Endocrinol Metab 2015;100:2347-53.
141. Szathmari M, Tulassay T, Arato A, et al. [Mineral content in bones of children with symptomless celiac disease and gluten-free diet]. Orv Hetil 1997;138:3233-8.
142. Taylor A, Johnson BH, Cappell K, et al. ASSESSING SEVERITY AND HEALTHCARE RESOURCE UTILIZATION IN CELIAC DISEASE: A U.S. CLAIMS DATA ANALYSIS. Digestive Disease Week, DDW 2019; United States: Gastroenterology; 2019. p. S916-7.
143. Tetzlaff WF, Bottero A, Neder D, et al. Antioxidant paraoxonase 1 activity in children with celiac disease. Impact of gluten free diet. EAS 2020 Congress; Virtual, Online: Atherosclerosis; 2020. p. e153.
144. Thong EP, Wong P, Dev A, et al. Increased prevalence of fracture and hypoglycaemia in young adults with concomitant type 1 diabetes mellitus and coeliac disease. Clin Endocrinol (Oxf) 2018;88:37-43.
145. Tolone C, Caprio AM, Casertano M, Belfiore I, Strisiciuglio C. Celiac patients with low adherence to gluten free diet can have negative serology, but their nutritional status is impaired. 52nd Annual Meeting of the European Society for Paediatric Gastroenterology Hepatology and Nutrition, ESPGHAN 2019; United Kingdom: Journal of Pediatric Gastroenterology and Nutrition; 2019. p. 190.
146. Tran CD, Katsikeros R, Manton N, et al. Zinc homeostasis and gut function in children with celiac disease. Am J Clin Nutr 2011;94:1026-32.
147. Tuna Kirsaclioglu C, Kuloglu Z, Tanca A, et al. Bone mineral density and growth in children with coeliac disease on a gluten free-diet. Turk J Med Sci 2016;46:1816-21.

148. Unalp-Arida A, Ruhl CE, Choung RS, Brantner TL, Murray JA. Lower Prevalence of Celiac Disease and Gluten-Related Disorders in Persons Living in Southern vs Northern Latitudes of the United States. Gastroenterology 2017;152:1922-32 e2.
149. Van Megen F, Fossli M, Skodje GI, Carlsen MH, Andersen LF, Veierod MB, Lundin KEA, Henriksen C. Nutritional assessment of women with celiac disaease compared to the general population. Clin Nutr ESPEN 2023; 54: 251-257
150. Varkonyi A, Boda M, Szokefalvi-Nagy Z, Nyilasi B. Determination of hair trace elements in childhood celiac disease and in cystic fibrosis. Acta Paediatr Hung 1992;32:159-65.
151. Ventura I, Rodriquez B, Suescum S, Revert F, Revert-Ros F, Moreno MA, Prieto-Ruiz JA, Perez-Bermejo M. More than three years for normalisation of routine laboratory values after gluten withdrawl in paediatric Coeliac Patients. Children (Basel); 2023; 10 ; 9
152. Villanueva J, Maranda L, Nwosu BU. Is vitamin D deficiency a feature of pediatric celiac disease? J Pediatr Endocrinol Metab 2012;25:607-10.
153. Vilppula A, Kaukinen K, Luostarinen L, et al. Clinical benefit of gluten-free diet in screen-detected older celiac disease patients. BMC Gastroenterol 2011;11:136.
154. Volkan B, Aykut Bayrak N, Cayir A. Bone mineral density and vitamin D treatment in patients with coeliac disease. 50th Annual Meeting of the European Society for Paediatric Gastroenterology, Hepatology and Nutrition, ESPGHAN 2017; Czechia: Journal of Pediatric Gastroenterology and Nutrition; 2017. p. 234-5.
155. Volkan B, Fettah A, Islek A, et al. Bone mineral density and vitamin K in children with celiac disease: Is there a link?. 49th Annual Meeting of the European Society for Paediatric Gastroenterology, Hepatology and Nutrition, ESPGHAN 2016; Athens Greece: Journal of Pediatric Gastroenterology and Nutrition; 2016. p. 171-2.
156. Volkan B, Bayrak NA, Haliloglu B, Kara SS, Cayir A. Pubertal development in children diagnosed with Celiac Disease. 49th Annual Meeting of the European Society for Paediatric Gastroenterology, Hepatology and Nutrition, ESPGHAN 2016; Athens, Greece: Journal of Pediatric Gastroenterology and Nutrition; 2016. p. 173.
157. Weintraub Y, Ben-Tov A, Dotan G, et al. Vitamin A levels are comparable between children with newly diagnosed coeliac disease and non-coeliac controls. Acta Paediatr 2019;108:2095-9.
158. Weir DG, Hourihane DO. Coeliac disease during the teenage period: the value of serial serum folate estimations. Gut 1974;15:450-7.
159. Wierdsma NJ, van Bokhorst-de van der Schueren MA, Berkenpas M, Mulder CJ, van Bodegraven AA. Vitamin and mineral deficiencies are highly prevalent in newly diagnosed celiac disease patients. Nutrients 2013;5:3975-92.
160. Wright RA, Dyner L, Rosa D, Cagnasso C, Olivera Carrion M. Nutritional status regarding iron and zinc in celiac children and mineral contribution of mixes flours. 21st International Congress of Nutrition, ICN 2017; Argentina: Annals of Nutrition and Metabolism; 2017. p. 989.
161. Yuce A, Demir H, Temizel IN, Kocak N. Serum carnitine and selenium levels in children with celiac disease. Indian J Gastroenterol 2004;23:87-8.

Supplementary Table ST5 - Detailed Characteristics of included studies

Study	Year	Language	Methods	Participants	Study Outcomes	Micronutrient Assessed	Notes
DICHOTOMOUS DATA AVAILABLE							
Anand et al.[38] (UK)	1977	English	Assessed absorption of iron by total body counter of radioactive $^{59}Fe^{++}$. CeD confirmed by biopsies. Assessed absorption of iron by Oxford total body counter of radioactive $^{59}Fe^{++}$	27 adults with CeD<br>-Group 1: untreated CeD. n=34; 11 had iron levels measured<br>-Group 2: CeD on GFD from 6 moths to 10 years. n=36; 14 had iron levels<br>-Controls without malabsorptive syndrome however 96 had iron deficiency anaemia. n=131	-To assess the absorption of inorganic $^{59}Fe^{++}$ in untreated and treated CeD and controls in comparison to haemoglobin iron	Iron	-Eligible for analysis: Group 1 and 2<br>-Data not available to establish the cut off of 2 years
Ballestero-Fernández et al.[30], 2021 (Spain)	2021	English	CeD confirmed by medical diagnosis. Micronutrients were assessed by routine clinical laboratory methods	64 adults with CeD belonging to Coeliac and Gluten Sensitive Association on GFD for over 1 year (n=64)<br>Controls recruited from the general population without diagnosis of chronic disease, no digestive symptoms and not taking nutritional supplements and all controls had screening negative anti-TTG. n=74	1- To assess nutritional status in adults with CeD on long-term GFD compared to healthy controls<br>2- To assess micronutrient measurements in CeD compared to controls and divided by sex	Calcium<br>Iron<br>Folate<br>Vitamin D	-Only Vitamin D had dichotomous data provided
Bayrak et al.[31] (Turkey)	2020	English	CeD confirmed by biopsies and serology. Micronutrients were assessed by routine clinical laboratory methods	228 children with CeD *<br>-Group 1: CeD on GFD from 3mo to 1 year. n=72<br>-Group 2: CeD on GFD for 1-3 years. n=53<br>-Group 3: CeD on GFD for over 3 years. n-103<br>-Controls were age + sex matched with negative serology for CeD. n=135	1- To assess age of menarche and pubertal development related to GFD in CeD through hormone measurement, Tanner stage and anthropometric measurements.	Ferritin<br>Folate<br>Vitamin B12<br>Vitamin 25(OH) D	-Eligible for analysis: Group 3 and controls
Choudhary et al.[32] (India)	2017	English	CeD confirmed by biopsies and serology. Micronutrients were assessed by routine clinical laboratory methods	72 children with CeD<br>- Group 1: untreated CeD newly diagnosed. n=36<br>- Group 2: CeD on GFD for over one year. n=36	1- To assess BMD and biochemical indices in CeD newly diagnosed compared to CeD on GFD for >1year	Calcium	
Ciacci et al.[42] (Italy)	2020	English	CeD confirmed by biopsies and serology. Calcium was measured by Architect assay. Vitamin 25(OH)D and 1-25(OH)D were measured by chemiluminescent assay. Vitamin 25(OH) D had both calibrated and non-calibrated to NIST-SRM972a reported	105 adults with CeD<br>Group1: untreated CeD. n=50<br>Group 2: CeD on GFD for mean of 10±8.8 years. n=55	1- To assess vitamin D levels in CeD in relationship to BMD and quantitative computed tomography	Calcium<br>Vitamin 25(OH)D<br>Vitamin 1-25(OH)D	-Reported severe and mild Vitamin D deficiency
Elli et al.[47] (Italy)	2015	English	CeD confirmed by medical assessment. Micronutrients were assessed by routine clinical laboratory methods	38 adults with CeD on GFD for mean of 9.0 years (±8.0). 16/39 CeD had iron deficiency anaemia<br>Controls: sex and age matched healthy volunteers. n=76	1- To evaluate the role of *TMPRSS8* variant *rs855791* in treated CeD with iron deficiency anaemia against non-iron deficient anaemia CeD and non CeD controls	Iron<br>Ferritin	-Dichotomous data for iron in CeD only
González et al.[35] (Argentina)	1995	English	CeD confirmed by biopsies and serology Calcium measured by atomic absorption, Vitamin D by protein competition assay of cytosol from rats	32 adult women with CeD<br>Group 1: untreated CeD. n=20<br>Group 2: CeD on GFD for mean 16 years (range 2-26). n=12<br>Controls were women who had BMD for another study. n=85	1- To assess extent of alterations in bone mass and body composition in untreated CeD compared to controls<br>2- To assess if alterations are corrected with GFD	Calcium<br>Vitamin 25(OH)D	-Eligible for analysis: Group 1 and 2
Högberg et al.[19] (Sweden)	2009	English	Serum zinc levels by atomic absorption spectroscopy compared to level of enteropathy on duodenal biopsies. CeD diagnosis confirmed by biopsies	59 children divided into 5 groups<br>Group 1: CeD untreated (Marsh 2-3) n=11<br>Group 2: CeD not confirmed (Marsh 0-1) n=16<br>Group 3: CeD on GFD for mean of 12 months (Marsh 0-1) n=14<br>Group 4: CeD on gluten challenge of mean 3 months (Marsh 2-3) n=12<br>Group 5: CeD on gluten challenge for mean 4 months (Marsh 0-1) n=6	1- To assess serum zinc profile of children at different stages of investigation for CeD	Zinc	-Eligible for analysis: Group 1 and 3
Hozyasz et al.[20] (Poland)	2003	Polish	Assessed vitamin E in plasma levels and by cholesterol, erythrocytes, and plasma retinol. Vitamin E and retinol levels in erythrocytes determined by liquid chromatography with UC radiation detector. CeD confirmed by biopsies and anti-Endomysium antibodies.	30 Adults and children with CeD were divided into 2 groups<br>Group 1: 18 CeD not on GFD<br>Group 2: 12 CeD on GFD for at least 2 years and negative Anti-Endomysium antibodies	1- To assess vitamin E status in patients with CeD	Vitamin A<br>Vitamin E (three methods; plasma, controlled by plasma cholesterol and in erythrocytes)	
Kavak et al.[49] (Turkey)	2003	English	CeD confirmed by biopsies and serology. Calcium was measured by standard laboratory methods and Vitamin25(OH)D by radioimmunoassay	62 children with CeD<br>Group1: untreated CeD. n=34<br>Group 2: CeD on GFD for over 1 year. n=28<br>Controls: age and sex matched healthy children with only BMD. n=64	1- To assess BMD in children with CeD at diagnosis and treated for 1 year	Calcium<br>Vitamin 25(OH)D	-Eligible for analysis: Group 1 and 2
Keaveny et al.[23] (Ireland)	1996	English	CeD confirmed by biopsies. Ionized calcium measured by ion-selective electrode with a Radiometer CAI2 analyzer. Vitamin 25(OH)D measured by radioimmunoassay and Vitamin 1, 25 (OH) D by competitive protein binding assay	51 adults with CeD<br>Group 1: Newly diagnosed CeD. Four participants were on low dose vitamin D supplements. n=19<br>Group 2: Treated CeD on GFD, mean GFD 6.7 years (1-35). n=16<br>Group 3: Refractory CeD on GFD on immunosuppressives. n=16	1- To assess bone remodelling indices and secondary hyperparathyroidism in adult CeD patients at different stages	ionized Calcium<br>Vitamin 25(OH)D<br>Vitamin 1-25(OH)D	-Eligible for analysis: Group 1 and 2<br>-4/19 CeD not on GFD were taking low dose vitamin D (3 <400IU 1>400IU)

Kemppainen et al.[24]<br>(Finland)	1995	English	CeD confirmed by biopsies and unclear diagnosis were excluded. Micronutrients were assessed by routine clinical laboratory methods	82 adults with CeD<br>-Group 1: Untreated CeD n= 40<br>-Group 2: Treated CeD on GFD >1yr with normalization of villous atrophy. n=42<br>-Control subjects recruited from two population based surveys (men from osteoporosis study/women from a prior case-control study) for BMD only	1- To assessed nutritional intake and nutritional status of newly diagnosed CeD compared to CeD on GFD<br>2- To compare anthropometric characteristics of CeD with control	Iron<br>Ferritin<br>Folate<br>Vitamin B12<br>Calcium<br>Magnesium	-Eligible for analysis: Group 1 and 2<br>-Calcium and magnesium reported continuous data only**
Klimov et al.[50]<br>(Russia)	2017	English	CeD confirmed by biopsies and serology. Vitamin 25(OH) D levels by enzyme immunoassays	77 children with CeD<br>Group1: untreated CeD. n=22<br>Group 2: CeD on strict GFD with unclear length of time. n=37<br>Group 3: CeD non-compliant GFD. n=18<br>Control: healthy children. n=14	1- To assess Vitamin D status in children with untreated and treated CeD compared to controls	Vitamin 25(OH)D	Conference poster<br>-Eligible for analysis: Group 1 and 2 and control
Margoni et al.[25]<br>(Greece)	2012	English	CeD confirmed by biopsies and Anti-TTG/EMA antibodies. Micronutrients were assessed by routine clinical laboratory methods	81 children with CeD<br>-Group 1: Newly diagnosed CeD. n=45<br>-Group 2: Ced on GFD >12 months (mean 77.7 ± 54.3). n=36<br>Prospective repeated bloodwork on 16 patients from Group 1	1- To assess status of bone health in newly diagnosed CeD<br>2- Effect of strict GFD on evolution of bone health parameters	Calcium<br>Vitamin 25(OH)D	
Mazure et al.[51]<br>(Argentina)	1994	English	CeD confirmed by biopsies and serology. Calcium was measured by atomic absorption and Vitamin 25 (OH) D by protein competition assay	42 adults with CeD<br>Group1: Asymptomatic CeD. n=8,<br>Group 2: Untreated symptomatic CeD, n=20<br>Group 3: CeD on GFD for mean 16 years (range 2-26 years). n=14<br>Controls: healthy adults age and sex matched who had BMD only. n=153	1- To assess BMD in asymptomatic CeD patients compared to controls and symptomatic CeD patients	Calcium<br>Vitamin 25(OH)D	-Eligible for analysis: Group 2 and 3
McGrogan et al.[26]<br>(Scotland, UK)	2021	English	CeD confirmed by biopsies and Anti-TTG levels. Vitamin A, B1,B2, B6, E, K by high-performance liquid chromatography (HPLC). Vitamin B2 and B6 in erythrocytes corrected by haemoglobin. Plasma Vitamin E corrected by total cholesterol and Vitamin K by triglycerides. Vitamin C measured by electrochemical detection. Zinc, copper, selenium by mass spectrometry. Ferritin, folate, B12 and magnesium measurement by routine clinical laboratory methods	106 children with CeD<br>Group1: Newly diagnosed untreated CeD. n=25<br>Group2: CeD on GFD for 6 months or less. n=21<br>Group3: CeD on GFD for 7-12 months. n=16<br>Group 4: CeD on GFD for over 12 months. n=44<br>-Group2 and 3 were combined data from a prospective and cross-sectional cohorts<br>-Not all participants had all the micronutrient measured	1- To asses micronutrient deficiencies in children with CeD<br>2- To assess changes in micronutrient deficiencies post GFD and if any are persistent despite GFD	Vitamin A, B1,B2, B6, B12, C, K, D, E<br>Folate<br>Magnesium<br>Copper<br>Zinc<br>Selenium<br>Ferritin	-Eligible for analysis: Group 1 and 4
Manseuto et al.[52]<br>(Italy)	2023	English	CeD confirmed by biopsies and serology. NCWS confirmed by absence serology and biopsy in setting of +HLA DQ2/8 Micronutrients were assessed by routine clinical laboratory methods	174 adults with CeD<br>-244 NCWS on GFD for 12 months<br>Controls: Adults with IBS and functional dyspepsia	1- To assess anemia in NCWS compared to CeD and healthy controls	Vitamin B12<br>Ferritin<br>Iron<br>Folate	
Piatek-Guziewicz et al.[54]<br>(Poland)	May 2017	English	CeD confirmed by biopsies and serology. Vitamin E measured by spectrophotometry. Ferritin and Vitamin D were assessed by routine clinical laboratory methods	145 adults with CeD<br>Group 1: untreated CeD. n=53<br>Group 2: CeD on GFD for over 2 years. n=92<br>Controls: Adults with functional GI disorders with negative CeD serology and biopsies. n=52	1- To evaluate oxidative stress imbalance in CeD by concentrations of nitric oxide and anti-oxidant parameters	Ferritin<br>Vitamin D<br>Vitamin E	-Eligible for analysis: Group 1, 2 and controls<br>-Vitamin E deficiency within CeD groups was not available **
Piatek-Guziewicz et al.[55]<br>(Poland)	Nov 2017	English	CeD confirmed by biopsies and serology. Micronutrients were assessed by routine clinical laboratory methods	60 adults with CeD<br>Group 1: untreated CeD. n=29<br>Group 2: CeD on GFD for over 2 years. n=31<br>Control: Functional GI disorders with negative CeD serology/biopsies. n=25	1- To determine the involvement of oxidative imbalance and mechanism of mucosal injury in CeD with non-classical symptoms and extraintestinal manifestations	Vitamin D	-Eligible for analysis: Group 1, 2 and controls
Romańczuk et al.[57]<br>(Poland)	2016	English	CeD confirmed by biopsies and serology. Vitamin E levels in erythrocytes determined by high-performance liquid chromatography with ultraviolet detector.	77 adults and children with CeD<br>Group 1: Untreated CeD. n=7<br>Group 2: CeD with non-compliance to GFD diet. n=22<br>Group 3: CeD on strict GFD for unclear amount of time. n=48<br>Controls: were healthy with negative serological markers of CeD (n=20)	1- To assess Vitamin E in erythrocytes in patients with untreated and treated CeD and controls.	Vitamin E	-Eligible for analysis: Group 1, 3 and controls
Selbuz et al.[58]<br>(Turkey)	2021	Turkish	CeD confirmed by biopsies and serology. Micronutrients were assessed by routine clinical laboratory methods.	70 children with CeD<br>Group 1: untreated CeD. n=34<br>Group 2: CeD on GFD for 3.8 ±3.4 years. n=22<br>Group 3: CeD not compliant with GFD. n=14<br>Controls: Healthy children sex and age matched n=57. No bloodwork for controls	1- To assess nutritional and growth status in children with CeD compared to healthy controls	Calcium, Zinc<br>Folic Acid, Iron<br>Vitamin A, B12, E,<br>Vitamin D<br>Magnesium	-Eligible for analysis: Group 1 and 2
Szaflarska-Poplawska et al.[28]<br>(Poland)	2022	English	CeD confirmed by biopsies and serology. folate was measured by chemiluminescence immunoassay, B vitamins by HPLC and others by routine laboratory methods	48 children on GFD for 5.02 ± 3.87 years<br>50 age matched controls	1- To assess micronutrient dietary intake and serum levels of CeD on GFD compared to controls on regular diet	Biopsies and serology	
Ünal et al.[61]<br>(Turkey)	2012	Polish	CeD confirmed by biopsies and serology	17 Children with CeD on GFD for 11.11±1.98 months<br>Controls: healthy age and sex matched (n=20)	1- To assess selenium levels in children with CeD on GFD compared to controls	Selenium	

			Selenium levels measured by atomic spectrometer				
Uyanikoglu et al.[62] (Turkey)	2021	English	CeD confirmed by biopsies and serology. Vitamin 25(OH)D and Vitamin 1-25(OH)D levels determined by liquid chromatography with tandem mass spectrometry	80 Adults with CeD<br>Group 1: untreated CeD. n=40<br>Group 2: CeD on GFD for unclear amount of time. n=40<br>Controls: were healthy adults that were age and sex matched. n=40	1- To assess 25(OH) vitamin D and 1,25(OH) vitamin D levels in untreated and treated adult CeD patients compared to controls	Vitamin 25(OH)D<br>Vitamin 1-25(OH)D	-Eligible for analysis: Group 1, 2 and controls<br>-Able to determine severe and mild Vitamin D deficiency
Continuous Data Only ***							
Ballestero-Fernández et al.[29] (Spain)	2019	English	CeD confirmed by medical diagnosis. Micronutrients were assessed by routine clinical laboratory methods	70 children with CeD belonging to Coeliac and Gluten Sensitive Association on GFD for over 1 year. 67 had bloodwork done<br>Controls recruited from the general population without diagnosis of chronic disease, no digestive symptoms and not taking nutritional supplements. n=67 and 66 had bloodwork done	1- To assess nutritional status in children with CeD on long-term GFD compared to healthy controls<br>2- To assess micronutrient measurements in CeD compared to controls and divided by sex and age	Calcium<br>Iron<br>Folate<br>Vitamin D	-Calculated mean form median
Björck et al.[39] (Sweden)	2017	English	CeD confirmed by biopsies and serology. Vitamin 25(OH) D was measured using liquid chromatography-mass spectrometry	101 children with CeD<br>Group 1: CeD not on GFD. n=71<br>Group 2: CeD on GFD of mean or 6.9±1.1 years. n=30<br>Control 1: age matched controls to group 1. n=142<br>Control 2: Age matched controls to group 2. n=60, 57 had bloodwork<br>Controls were serological negative children but matched to HLA-DQ allele, sex and age	1- To assess whether bone mass and metabolism are impaired in CeD and genetically at-risk children with screening detected CeD	25 (OH) vitamin D	-Eligible for analysis: Group 2 and Control 2
Boda et al.[40] (Hungary)	1989	Hungarian	CeD confirmed by biopsies. Selenium in erythrocyte concentration measured by fluorimetry	49 children with CeD<br>Group 1: Untreated CeD. n=24<br>Group 2: CeD on GFD for mean of 17.5 months, n=25<br>Controls: Age matched with no hematological conditions or CeD. n=15	1- To assess selenium erythrocyte concentration in untreated and treated CeD compared to controls	Selenium	Eligible for analysis: Group 1, 2 and control
Bulut et al.[41] (Turkey)	2023	English	CeD confirmed by biopsies and serology. Micronutrients were assessed by routine clinical laboratory methods	49 Children with CeD<br>Group 1: untreated CeD. n=18<br>Group 2: CeD on GFD for mean of 6 months. n=31	1- To assess bone structure and integrity between CeD on and off CGF	Vitamin 25(OH)D<br>Calcium	GFD confirmed by TTG levels
Corazza et al.[43] (Italy)	1988	English	CeD confirmed by biopsies. Serum zinc levels were measured by atomic absorption spectrophotometry	48 adults with CeD<br>Group 1: untreated CeD. n=30<br>Group 2: treated CeD with GFD for 26±15 months. n=18<br>Controls: healthy volunteers that were age and sex matched. n=30	1- To assess cell-mediated immunity, nutrition status and zinc levels in adult CeD	Zinc<br>Copper	-Eligible for analysis: Group 1, 2 and control<br>-Data for Copper not in text
Corazza et al.[33] (Italy)	1995	English	CeD confirmed by biopsies or positive serology with family history. Serum 25-OH Vitamin D measured by radioimmunoassay.	31 adults with CeD<br>Group 1: untreated CeD. n=17<br>Group 2: CeD on GFD for median of 2.4 years. n=14<br>Controls: Healthy adult volunteers. n=24	1- To assess bone metabolism in CeD compared to controls	Calcium<br>Vitamin 25(OH)D<br>Vitamin 1-25(OH)D	
Cortigiani et al.[44] (Italy)	1989	Italian	Assessed serum selenium levels by atomic absorption spectrophotometer in CeD untreated, treated and controls. CeD diagnosed by biopsies	73 children and young adults (up to age 20) with CeD<br>Group 1: untreated CeD. n=37<br>Group 2: CeD on GFD for at least a year. n=36<br>-9 subjects were studied on both at diagnosis and on GFD<br>Control: healthy children. n=51	1- To assess selenium levels in untreated and treated CeD compared to controls	Selenium	-Eligible for analysis: Group 1, 2 and control
Dickey et al.[45] (Ireland)	2008	English	CeD confirmed by biopsies and serology. Measurements of Vitamin B6 by pyridoxal 5-phosphate (PLP), Vitamin B2 by erythrocyte glutathione reductase activation coefficient (EGRAC), and Vitamin B12 and folate by microbiological assay	100 adults with CeD<br>Group1: untreated CeD. n=35<br>Group 2: CeD on GFD for >1year with normalized villous atrophy. n=41<br>Group 3: CeD on GFD for >1year with persistent villous atrophy. n=24<br>Controls: age and gender matched who were participating in other B-vitamin studies with no history of gastrointestinal disorders. n=200	1- To assess nutritional status and B vitamin levels between untreated and treated CeD and healthy controls	Folate<br>Vitamin B2<br>Vitamin B6<br>Vitamin B12	-Eligible for analysis: Group 1, 2 and control
Efe et al.[46] (Turkey)	2023	English	CeD confirmed by biopsies and serology. Micronutrients were assessed by routine clinical laboratory methods	85 children with CED<br>Group1: poor GFD adherence (n=35) over last 3 months<br>Group 2: good GFD adherence (n=50) (confirmed by diet and serology)<br>Controls: Healthy volunteers with no psychiatric or physical illness (n=72)	1- To assess ADHD traits on CeD patients and associated micronutrients	Vitamin 25 (OH)D, Vitamin B12, Iron, Ferritin	-Eligible for analysis: Group 2 and control
El Amrousy et al. (Eygpt)	2024	English	CeD confirmed by biopsies and serology. Micronutrients were assessed by routine clinical laboratory methods	40 children with CED on GFD<br>Controls: healthy age and sex matched (n=40)	1- To assess cardiac function for CeD patients and associated micronutrients	Ferritin, Vitamin 25 (OH)D	
Henker et al.[18] (Germany)	1985	German	Serum zinc levels at different stages of disease by atomic absorption spectrophotometry. CeD confirmed by biopsies	78 children with CeD divided into 5 groups:<br>Group1: 26 children with active untreated CeD, total villous atrophy on gluten<br>Group 2: 22 children with total villus atrophy with ongoing diet errors with gluten<br>Group 3: 6 children with partial villous atrophy post gluten challenge<br>Group 4: 23 children on GFD for 6 months<br>Group 5: 63 children on GFD for more than 6 months	1- To assess zinc concentrations in patients with coeliac disease compared to different stages of disease<br>2- To assess relationship of zinc concentrations to albumin, iron, alkaline phosphatase and body length and weight	Zinc	-Eligible for analysis: Group 1/2 and Group 5

Isikay et al.[36] (Turkey)	2018	English	CeD confirmed by intestinal biopsies and/or EMA IgA and anti TTG IgA. Micronutrients were assessed by routine clinical laboratory methods	226 CeD children on GFD for 3 months to 5 years<br>Controls were hospitalized patients with upper airway infection with no chronic diseases n=268	1- To assess the prevalence of restless leg syndrome in CeD compared to non CeD controls<br>2- To investigate associated factors for restless leg syndrome	Ferritin<br>Folate<br>Vitamin B12<br>Vitamin 25(OH)D	
Kalayci et al.[48] (Turkey)	2001	English	CeD diagnosed by biopsies and serology. Calcium was measured by a timed-endpoint biuret method corrected for albumin were appropriate	32 children with CeD<br>Group1: untreated CeD. n=16<br>Group2: CeD on GFD for 3.36 (range 1.6-7) years. n=16.<br>Controls: children with upper respiratory infection, sex-age matched. n=82	1- To assess prevalence of osteopenia and relationship between BMD and metabolites in untreated and treated CeD in children compared to controls	Calcium	-Eligible for analysis: Group 1, 2 and control
Kalita et al.[21] (Poland)	2002	Polish	Concentration of selenium in CeD and healthy controls. CeD confirmed by both intestinal biopsies and anti-EMA. Serum selenium concentration by electrothermal atomic absorption	114 children with CeD at various stages<br>Group 1: New diagnosed of CeD (n=9)<br>Group 2: CeD on GFD with negative anti EMA IgA n=31<br>Group 3: untreated CeD n=54<br>Group 4: CeD during gluten challenge. n=20<br>Control group: non-CeD or other gastrointestinal diseases. n=27	1- To evaluate serum selenium levels in children with CeD	Selenium	-Eligible for analysis: Group 1, 2, and controls<br>-Duration of GFD not reported
Karkoszka et al.[22] (Poland)	2000	Polish	CeD confirmed by duodenal biopsies with atrophy and resolution post GFD. Total plasma calcium determined by flame photometry.	73 children with CeD<br>Group 1: CeD strictly on GFD for mean 11.7±0.6 years (n=33). GFD adherence confirmed by negative anti-EMA antibodies<br>Group 2: untreated CeD (n=40)	1- To assess influence of GFD on bone mineral density (BMD) with BMI and serum bone indices in CeD	Calcium	
Pazianas et al.[53] (England)	2005	English	CeD confirmed by biopsies. calcium measured by standard laboratory method, Vitamin 25(OH)D by radioimmunoassay	24 adult females with CeD on GFD for 4.7±3.1 years<br>Controls: healthy female hospital staff volunteers. n=20	1- To assess calcium absorption and BMD in CeD after long-term GFD	Calcium<br>Vitamin 25(OH)D	
Reinken, et al.[56] (Austria)	1976	English	CeD confirmed by biopsies. Vitamin B6 measured by activity of pyridoxal phosphate (PALP) and pyridoxal-kinase (PALK) through specific assays	20 children with CeD<br>Group 1: untreated CeD. n=15<br>Group 2: CeD on GFD for 0.5-5 years (n=6)<br>Controls: children with normal biopsies but history of cow's milk allergies, recurring diarrhea, salmonella case and suspected but not confirmed CeD. n=20	1- To assess Vitamin B6 concentrations in adult untreated and treated CeD compared to controls	Vitamin B6	-Eligible for analysis: Group 1, 2 and controls<br>-Data not available to establish the cut-off of 2 years
Sabel'nikova et al.[27] (Russia)	2013	Russian	CeD confirmed by biopsies and serum anti-gliadin and TTG antibodies.	Total 87 adult patients with CeD<br>Group1: Not adhering to GFD. n=24; 33 blood samples<br>Group2: CeD on strict GFD. n= 63; 109 blood samples	- To elaborate recommendations for rehabilitation of patients with CeD on long term follow up based on GFD adherence	Iron<br>Calcium	-Follow up 6 to 31 years
Singhal et al.[59] (India)	2008	English	CeD confirmed by biopsies and serology. Zinc levels determined by atomic absorption spectrophotometry	30 children with CeD<br>Group 1: untreated CeD. n=23<br>Group 2: CeD on GFD for over 3 months. n=7<br>Controls: children with chronic diarrhea negative workup for CeD (n=27)	1- To assess zinc levels in untreated and treated CeD	Zinc	-Eligible for analysis: Group 1, 2 and controls
Szymczak et al.[60] (Poland)	2012	English	CeD confirmed by biopsies. Vitamin 25(OH) and 1-25(OH)D were measured by radioimmunoassay (RIA). Calcium was assessed by routine laboratory methods	35 adults with CeD<br>Group 1: untreated CeD. n=16<br>Group 2: CeD on GFD for over 1 year. n=19<br>Controls: healthy adults with normal BMI. n=36	1- To assess BMD and calcium deficiencies in adult CeD<br>2- To assess if GFD sufficient in BMD restoration or if supplementation is required	Calcium<br>Vitamin 25(OH)D<br>Vitamin 1-25(OH)D	-Eligible for analysis: Group 1, 2 and controls
Valente et al.[37] (Brazil)	2015	English	Assessed B vitamins, nutritional status in CeD on GFD with controls. Vitamin B6 by PLP chromatography. CeD diagnosed by biopsies	40 adults with CeD on GFD for at least 6 months<br>Controls were healthy subjects recruited, age and sex matched (n=40)	1- To assess nutritional status and levels of B vitamins in homocysteine metabolism<br>2- To determine CeD patients have adequate dietary intake of B vitamins	Folate<br>Vitamin B6<br>Vitamin B12	Data not available to establish the cut off of 2 years
Volkan et al.[63] (Turkey)	2018	English	CeD confirmed by biopsies and serology. Micronutrients were assessed by routine clinical laboratory methods. Vitamin K were assessed by enzyme-linked immunosorbent assay	72 children with CeD<br>Group1: Untreated CeD. n=26<br>Group 2: CeD on GFD with poor compliance. n=25<br>Group 3: CeD on GFD for with good compliance. n=21<br>Controls: healthy age and sex matched children. n=30	1- To investigate BMD in children with CeD<br>2- To Evaluate association between vitamin K levels and osteoporosis	Calcium<br>Ferritin, folate<br>Vitamin B12, K<br>Magnesium<br>Vitamin 25(OH)D	- Eligible for analysis: Group 1, 3 and controls<br>-Mean follow up time of 2.7 years, unclear cut off for length of GFD

Supplementary Table ST6 - Summary of All Studies Evaluating Micronutrient Levels in CeD on GFD compared to either Control Group

Study, Year	Country	Study design	Age Category	N	GFD length	Vit A	Vit B1	Vit B2	Vit B6	Vit B12	Vit D	Vit 1,25-OH D	Vit E	Vit K	Calcium	Copper	Ferritin	Folate	Iron	Mg	Selenium	Zinc
**CED not on GFD compared to GFD**																						
Anand 1977	UK	2	NR	25	Short-Term*														**			
Bayrak 2020	Turkey	4	Children	175	Long-Term																	
Boda 1989	Hungary	2	Children	49	Short-Term																	
Bulut 2023	Turkey	2	Children	49	Short-Tem						**				**							
Choudhary 2017	India	4	Children	72	Short-Term*										**							
Ciacci 2020	Italy	2	Adults	105	Long-Term																	
Corazza 1988	Italy	2	Adults	48	Short-Term*																	**
Corazza 1995	Italy	4	Adults	31	Short-Term*						**				**							
Cortigiani 1989	Italy	2	Children	73	Short-Term*																**	
Dickey 2008	Ireland	2	NR	76	Short-Term*													**				
González 1995	Argentina	4	Adults	32	Long-Term																	
Henker 1985	Germany	3	Children	111	Short-Term																	**
Högberg 2009	Sweden	3	Children	25	Short-Term																	**
Hozyasz 2003	Poland	3	Both	30	Long-Term								**									
Kalayci 2001	Turkey	2	Children	32	Long-Term										**							
Kalita 2002	Poland	3	Children	40	NR (Short-																**	
Karkoszka 2000	Poland	3	Children	73	Long-Term										**							
Kavak 2003	Turkey	2	Children	62	Short-Term*										**							
Keaveny 1996	Ireland	3	Adults	35	Long-Term							**										
Kemppainen 1995	Finland	3	Adults	88	Short-Term*					**												
Klimov 2017	Russia	2	Children	59	NR (Short-																	
Margoni 2012	Greece	3	Children	81	Long-Term																	
Mazure 1994	Argentina	2	Adults	34	Long-Term																	
McGrogan 2021	UK	3	Children	69	Short-Term*		**		**				**	**			**			**		
Piatek-Guziewicz 2017 May	Poland	2	Adults	145	Long-Term																	
Piatek-Guziewicz, 2017 Nov	Poland	2	Adults	60	Long-Term																	
Reinken 1976	Austria	2	Children	20	Short-Term*				**													
Romańczuk 2016	Poland	3	Both	55	NR (Short-																	
Sabel'nikova 2013	Russia	3	Adults	142	NR (Short-										**				**			
Selbuz 2021	Turkey	2	Children	56	Short-Term*																	
Singhal 2008	India	2	Children	30	Short-Term																	
Szymczak 2012	Poland	2	Adults	35	Short-Term*						**				**							
Uyanikoglu 2021	Turkey	2	Adults	80	NR (Short-																	
Volkan 2018	Turkey	2	Children	47	Short-Term*					**					**			**				
**Non CeD controls compared to CED on GFD**																						
Ballestero-Fernández 2019	Spain	4	Children	137	Short-Term*																	
Ballestero-Fernández 2021	Spain	4	Adults	138	Short-Term*																	
Bayrak 2020	Turkey	4	Children	238	Long-Term																	
Björk, 2017	Sweden	2	Children	90	Long-Term																	
Boda, 1989	Hungary	2	Children	40	Short-Term																**	
Corazza 1988	Italy	2	Adults	48	Short-Term*																	**
Corazza, 1995	Italy	4	Adults	38	Short-Term*							**			**							
Cortigiani, 1989	Italy	2	Children	87	Short-Term*																**	
Dickey 2008	Ireland	2	NR	241	Short-Term*			**														
Efe, 2023	Turkey	2	Children	122	Short-Term					**	**						**		**			
El Amrousy, 2024	Egypt	3	Children	80	Short-Term						**						**					
Elli 2015	Italy	2	Adults	114	Long-Term														**			
Isikay 2018	Turkey	4	Children	494	Short-Term*					**							**	**				
Kalayci, 2001	Turkey	2	Children	98	Long-Term																	
Kalita, 2002	Poland	3	Children	58	NR (Short-																**	
Klimov 2017	Russia	2	Children	51	NR (Short-						**											
Mansueto, 2023	Italy	2	Adults	298	Short-Term*					**								**	**			
Pazianas 2005	UK	2	Adults	44	Long-Term																	
Piatek-Guziewicz, 2017 May	Poland	2	Adults	144	Long-Term						**		**									
Piatek-Guziewicz, 2017 Nov	Poland	2	Adults	56	Long-Term																	
Reinken, 1976	Austria	2	Children	16	Short-Term*																	
Romańczuk, 2016	Poland	3	Both	68	NR (Short-								**									
Singhal 2008	India	2	Children	34	Short-Term																	
Szaflarska-Poplawska, 2022	Poland	4	Children	98	Long-Term		**	**							**			**		**		
Szymczak, 2012	Poland	2	Adults	55	Short-Term*																	
Ünal 2012	Turkey	2	Children	37	Short-Term																**	
Uyanikoglu, 2021	Turkey	2	Adults	80	NR (Short-							**										
Valente 2015	Brazil	4	Adults	59	Short-Term													**				
Volkan, 2018	Turkey	2	Children	51	Short-Term*															**		
**Non CeD controls compared to NCWS**																						
Mansueto, 2023	Italy	2	Adults	368	Short-Term*					**							**	**	**			

NOTE: 1=RCT; 2=Case control ; 3=Cohort ; 4= Cross-sectional. Green= increased in mean micronutrient levels in patients on GFD compared to control, Red= decreased in mean micronutrient levels in patients on GFD compared to control, Yellow= no change in mean micronutrient levels in patients on GFD compared to control, Grey- only dichotomous data provided or incomplete, blank is not measured within the study. CeD- Celiac Disease GFD= Gluten free diet. Vitamin C was not included as was not compared to controls[29]

*- Determined to be short term due to lack of definitive length of GFD over 2 years in text

**- Statistically significant difference ($p<0.05$)

<u>Supplementary data:</u> Studies evaluating levels of vitamins and minerals reported as continuous outcomes.

Vitamin A

Serum vitamin A levels were available for two cohort studies that showed no significant difference between groups (Supplementary Figure S1B).

Vitamin B1

Serum levels of vitamin B1 was assessed in one study[31]. Serum levels of vitamin B1 was significantly higher in CeD on GFD compared to CeD not on GFD (Mean Difference; MD=265; 95%CI=215,315) (Supplementary Figure S2C, Supplementary Table 6). One study[28] assessed Vitamin B1 in CeD on GFD compared to non CED controls and found as significantly decrease in Vitamin B1 in CeD on GFD (MD= -6.20; 95%CI= -10.88, -1.52) (Supplementary Figure S2D). The quality of data was determined to be low due to high risk of bias (Table 2).

Vitamin B2

A case-control study[47] comparing CeD on GFD with both and CeD not on GFD and non-CeD controls, provided EGRAC activity as a marker of serum vitamin B2 levels and found no significant difference between CeD on GFD and CeD not on GFD (Supplementary Figure S3B) although the CeD on GFD group had significantly lower EGRAC than non-CeD controls (MD=-0.08; 95%CI=-0.12,-0.04; Table 1, Supplementary Figure S3C, and Supplementary Table 6). Another study[28] found significantly higher serum vitamin B2 levels measured by HPLC in CeD on GFD compared to non-CeD controls (MD= 0.67; 95%CI= 0.27, 1.08) (Supplementary Figure S3D).

Vitamin B6

Vitamin B6 levels were measured by 4 studies using different methodology for assessment and therefore the studies were not combined for analysis. One study[31] found significantly lower levels of serum Vitamin B6 measured in hemoglobin (MD=-

1024; 95%CI=-1399.79, -648.21) (Supplementary Figure S4B). The remaining two studies, measuring vitamin B6 in serum pyridoxal phosphate (PALP) (ng/ml)[56] and pyridoxal-5-phosphate (PLP) (pmol/g)[40] showed levels of vitamin B6 were higher in CeD on GFD compared with CeD not on GFD (MD=4.11; 95%CI=0.90,7.32) but were similar to non-CeD controls by all methods, including HPLC by another study (REF) (Supplementary Figure S4C, 4D and Supplementary Table 6)

Vitamin B12

Two studies[47,63] assessing serum vitamin B12 deficiency in CeD on GFD compared to CeD not on GFD, and four studies [39,40,47,63] assessing CeD on GFD compared to non-CeD controls had continuous data only (Table 1, Supplementary Table 6). Serum vitamin B12 levels were significantly higher in CeD on GFD compared to CeD not on GFD in four studies [29,31,47,63] (MD=68.83; 95%CI=32.85,104.80; I2=0%) (Supplementary Figure S5C). Serum vitamin B12 levels were not significantly different in CeD on GFD compared to non-CeD controls in seven studies (MD =-4.18; 95%CI=-25.72, 17.36; I2=81%) (Supplementary Figure S5D). Sub group analysis based on study design, GFD length and population did not resolve heterogeneity (Supplementary Figure S5E-G). However of note, sensitivity analysis with eliminating one study[52] where means were calculated from the median did affect the final result.

Vitamin D

Fourteen studies demonstrated significantly higher serum Vitamin D levels in CeD on GFD compared to CeD not on GFD (MD=2.62; 95%CI=1.68, 3.56; I^2=51%) (Supplementary Figure S6Ei) and the associated funnel plot did not show significant asymmetry (Egger's test intercept =-0.44; 95%CI=-1.40- 0.52; t=-1.03, p=0.33) (Supplementary Figure S6Fii). On subgroup analysis, CeD on Long-Term GFD resolved heterogeneity (Supplementary Figure S6G). Fifteen studies did show a significant decrease in serum levels of Vitamin D in CeD on GFD compared to non-CeD controls (MD=-3.33; 95%CI=-5.72, -0.93; I^2=87%) (Supplementary Figure S6I). Subgroup analyses by study design, GFD duration and age did not resolve heterogeneity (Supplementary Figure S6F-L).

Five studies provided continuous data only for serum levels of 1,25(OH) vitamin D in CeD on GFD compared to CeD not on GFD or non-CeD controls. The characteristics are summarized in both Table 1 and Supplementary Table 6. Four studies showed significantly lower serum levels of 1,25(OH) vitamin D in CeD on GFD compared to CeD not on GFD (MD=-10.07; 95%CI=-19.06,-1.09; I2=87%) (Supplementary Figure S7B). Sensitivity analysis by removing the one study[23] that reported low dose vitamin D supplementation in 4/19 of CeD not on GFD resolved heterogeneity (MD= -5.78; 95% CI= -10.11, 01.45; I2=0%) but did not change overall results (Supplementary Figure S7C). The levels of 1,25(OH) vitamin D were not significantly different when comparing CeD on GFD to non-CeD controls (Supplementary Figure S7D and 7E). The quality of evidence was determined to be very low due to risk of bias (Table 2).

Vitamin E

Two studies assessed proportion of Vitamin E deficiency by erythrocyte level demonstrated a significantly lower risk in CeD on GFD compared to CeD not on GFD (OR = 0.03; 95%CI=0.00-0.41; I2=38%; Supplementary Figure S8B). One study that assessed serum vitamin E levels by chromatography demonstrated a significant higher level in CeD on GFD compared to CeD not on GFD (MD=5.44; 95%CI=0.68,10.20) (Supplementary Figure S8C). The other studies assessing vitamin E levels by different methodology and comparing both CeD on GFD to CeD not on GFD did not show overall significant differences in levels (Supplementary Figure S8D-8G). Both studies comparing CeD on GFD to non-CeD controls demonstrated a significantly lower levels in CeD on GFD (MD=-4.91; 95%CI=-14.3,4.47; I2=80%; Supplementary Figure S8H). Subgroup analysis by methodology or length of GFD did not resolve heterogeneity. The quality of evidence was determined to be very low due to risk of bias (Table 2).

Vitamin K

Levels of Vitamin K was assessed in 2 studies; one measured plasma levels of vitamin K controlled for triglycerides, and the remaining case-control study measured plasma levels of vitamin K not controlled for triglycerides. Pooled data showed significantly higher serum

levels of vitamin K between CeD on GFD compared to either CeD not on GFD or non-CeD controls (MD=0.53; 95%CI=0.37,0.69; Supplementary Figure S9A and 9B).

Calcium

Ten studies that assessed serum levels of calcium in CeD on GFD compared to CeD not on GFD presented continuous data. In addition, all studies comparing serum calcium levels of CeD on GFD to non-CeD controls reported continuous data. The characteristics of these studies are summarized in Table 1 and Supplementary Table 6. Two studies reported median levels of calcium were in the normal range in both CeD on GFD and non-CeD controls, however the interquartile range (IQR) did include levels below the normal range. Authors were contacted to provide dichotomous data. There were significantly higher calcium levels in CeD not on GFD compared with CeD on GFD (MD=0.07;95%=0.04-0.10; I2=94%) in 14 studies (Supplementary Figure S10Fi). Subgroup analysis by study design or length of GFD, or age did not resolve the heterogeneity or changed the results (Supplementary Figure S10G-10I). The asymmetric funnel plot of pooled studies suggested publication bias (Egger's test intercept=-1.466; 95%CI=-3.971,1.04; t=-1.23, p=0.226) (Supplementary Figure S10Fii). Eight studies assessing serum calcium levels between CeD on GFD and non-CeD controls showed similar calcium levels between groups (MD=-0.02; 95%CI=-0.07,0.03; I2=88%), with high heterogeneity that did not resolve with planned subgroup analyses (Supplementary Figures 10J-10M). The quality of evidence was determined to be very low due to risk of bias and inconsistency (Table 2).

Copper

One cohort study assessed serum copper levels in CeD on GFD compared with CED not on GFD in 65 participants. There was no difference in serum copper levels (MD=-0.43; 95%CI=-2.2,1.34) (Supplementary Figure S11B)

Folate

Two studies that assessed folate serum levels in CeD on GFD compared to CeD not on GFD reported only continuous data. An additional six studies comparing serum folate

levels of CeD on GFD to non-CeD controls presented only continuous data. Two studies mentioned that the median levels of folate were in the normal range in both CeD on GFD and non-CeD controls, however the IQR did include levels below the normal range. Authors were contacted to provide more data for dichotomous analysis. The characteristics of these studies are summarized in Table 1 and Supplementary Table 6. One cohort study reported higher erythrocyte folate levels in CeD on GFD compared to CeD not on GFD (MD=174; 95%CI=83, 262). Three studies showed no difference in serum folate levels comparing CeD on GFD to CeD not on GFD (MD=4.82; 95%CI=-1.57,11.21; I2=53%) (Supplementary Figure S12D). All studies were short term GFD. Subgroup analysis by study design and age did not resolve heterogeneity (Supplementary Figure S12E-F). In addition, there was also no significant difference in serum folate levels between CeD on GFD and non-CeD controls in nine pooled studies (MD=-3.63; 95%CI=-8.21,0.94; I2=94%) (Supplementary Figure S12G). Subgroup analysis by study design and population did not resolve heterogeneity (Supplementary Figure S12H-12J). The quality of evidence was determined to be very low due to risk of bias (Table 2).

Ferritin

Two studies that assessed serum ferritin levels in CeD on GFD compared to CeD not on GFD presented continuous data. In addition, the remaining four studies comparing serum ferritin levels of CeD on GFD to non-CeD controls reported continuous data. The characteristics of these studies are summarized in Table 1 and Supplementary Table 6. Four studies demonstrated a significantly higher serum ferritin levels in CeD on GFD compared to CeD not on GFD (MD=10.82, 95%CI= 5.97,15.71; I2=0%) (Supplementary Figure S13B). Eight studies showed a significantly decreased level of ferritin in CeD on GFD compared to non-CeD controls (MD=-13.45; 95%CI=-25.39,-1.50; I2=98%) (Supplementary Figure S13C). The heterogeneity did resolve with both study design and length of GFD, but not population (Supplementary Figure S13D-F). The quality of evidence was determined to be very low due to risk of bias (Table 2).

Iron

One study that assessed serum iron levels in CeD on GFD compared to CeD not on GFD reported only continuous data. For comparison to non-CeD controls, one study[47] suggested 42% (16/38) of patients with CeD on GFD had iron deficiency but did not report the status of the non-CeD control group. The other remaining two studies reported normal median serum iron levels for both CeD on GFD and non-CeD controls, although the IQR did include levels below the normal range. Authors were contacted for clarification but no response was received. The characteristics of these studies are summarized in Table 1 and Supplementary Table 6. Three pooled studies did not find a significant difference in serum iron levels and subgroup analysis with study design, GFD length and population did not resolve heterogeneity between CeD on GFD and not on GFD (Supplementary 14B-14D). Six studies assessing CeD on GFD to non-CeD controls found significant decrease in serum iron levels in CeD on CFD (MD= -11.72, 95%CI= -21.28, -2.17, I2= 90%) (Supplementary Figure S14E). Subgroup analysis by study design, length of GFD and population did not resolve heterogeneity (Supplementary Figure S14F-14H).

Magnesium

Three pooled studies demonstrated no significant difference in serum magnesium levels between CeD on GFD compared to CeD not on GFD (MD=0.02, 95%CI=-0.02,0.06, I2=79%) (Supplementary Figure S15B). All studies were on short-term GFD. Subgroup analysis by study design and population did not improve heterogeneity or change the results (Supplementary Figure S15C, 15D). Two studies demonstrated no significant differences in the levels of serum magnesium in CeD on GFD compared to non-CeD controls (MD= -0.02; 95%CI=-0.27,0.22, I2=96%) (Supplementary Figure S15E). Heterogeneity did not resolve with subgroup analysis for study design or length of GFD (data not shown). The quality of evidence was determined to be very low due to risk of bias and imprecision (Table 2).

Selenium

Three studies assessing serum selenium levels in comparing CeD on GFD to both CeD not on GFD and non-CeD controls provided continuous data only. There was no difference in serum selenium levels between CeD on GFD compared to CeD not on GFD

(MD=1.61; 95%CI=-0.39,3.61; I2=97%) (Supplementary Figure S16C). Subgroup analysis by study design only, as all studies were in children on short-term GFD, did not resolve heterogeneity (Supplementary Figure S16D). In comparing CeD on GFD to non-CeD controls, there was no significant difference in serum selenium levels (MD=0.38; 95%CI=-1.95,2.71; I2=95%) (Supplementary Figure S16E). Subgroup analysis by study design and GFD length did not resolve heterogeneity (Supplementary Figure S16F, 16G). In comparing levels of selenium in erythrocyte data in one study; there was no difference in selenium levels between CeD on GFD compared to CeD not on GFD (Supplementary Figure S16H) but significantly lower levels in CeD on GFD compared to non CeD controls (Supplementary Figure S16I). The quality of evidence was determined to be very low due to risk of bias, inconsistency, and imprecision (Table 2).

Zinc

Two studies assessing serum zinc levels in CeD on GFD compared to both CeD not on GFD and non-CeD controls provided continuous data only (Summarized in Table 1 and Supplementary Table 5). Five studies demonstrated a significantly increased mean serum zinc level in CeD on GFD compared to CeD not on GFD (MD=3.01; 95%CI=0.38,5.64; I2=94%) (Supplementary Figure S17B). Subgroup analysis by study design resolved the heterogeneity (Supplementary Figure S17C). Zinc levels were significantly lower in CeD on GFD compared to non-CeD controls in two pooled studies (MD=-1.88; 95%CI=-3.06,-0.69; I2=0%) (Supplementary Figure S17D). The quality of evidence was determined to be very low due to risk of bias, inconsistency, and imprecision (Table 2).

Supplementary Figures

Supplementary Figure S1. Vitamin A

S1A- Forest plot comparison of Vitamin A deficiency between CeD on GFD with CeD not on GFD

	CeD GFD		CeD non GFD			Odds Ratio
Study or Subgroup	Events	Total	Events	Total	Weight	M-H, Random, 95% CI
Hozyasz 2003	0	12	0	18		Not estimable
McGrogan 2021	10	36	5	25	83.6%	1.54 [0.45, 5.22]
Selbuz 2021	0	2	2	4	16.4%	0.20 [0.01, 6.66]
**Total (95% CI)**		**50**		**47**	**100.0%**	**1.10 [0.25, 4.86]**
Total events	10		7			

Heterogeneity: $Tau^2 = 0.30$; $Chi^2 = 1.17$, df = 1 (P = 0.28); $I^2 = 14\%$
Test for overall effect: Z = 0.13 (P = 0.90)

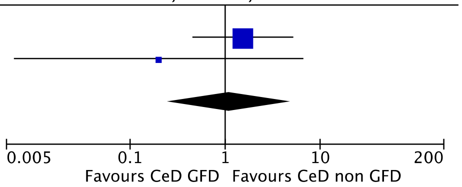

S1B- Forest plot comparison of means of serum Vitamin A levels between CeD on GFD with CeD not on GFD in cohort studies

	CeD GFD			CeD non GFD				Mean Difference
Study or Subgroup	Mean	SD	Total	Mean	SD	Total	Weight	IV, Random, 95% CI
Hozyasz 2003	1.95	0.72	12	1.74	0.57	18	17.3%	0.21 [−0.28, 0.70]
McGrogan 2021	0.96	0.28	36	1.04	0.32	25	82.7%	−0.08 [−0.24, 0.08]
**Total (95% CI)**			**48**			**43**	**100.0%**	**−0.03 [−0.24, 0.19]**

Heterogeneity: $Tau^2 = 0.01$; $Chi^2 = 1.25$, df = 1 (P = 0.26); $I^2 = 20\%$
Test for overall effect: Z = 0.27 (P = 0.79)

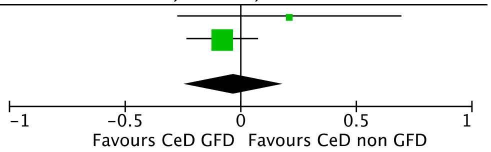

Supplementary Figure S2. Vitamin B1

S2A- Forest plot comparison of Vitamin B1 deficiency between CeD on GFD with CeD not on GFD in a cohort study

	CeD GFD		CeD non GFD			Odds Ratio
Study or Subgroup	Events	Total	Events	Total	Weight	M-H, Random, 95% CI
McGrogan 2021	2	40	5	24	100.0%	0.20 [0.04, 1.13]
**Total (95% CI)**		**40**		**24**	**100.0%**	**0.20 [0.04, 1.13]**
Total events	2		5			

Heterogeneity: Not applicable
Test for overall effect: Z = 1.82 (P = 0.07)

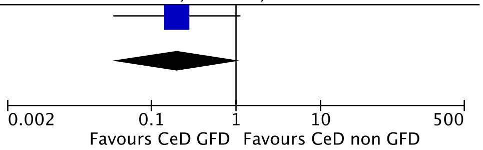

S2B- Forest plot comparison of Vitamin B1 deficiency between CeD on GFD with non-CeD controls in a cohort study

	CeD GFD		Controls			Odds Ratio
Study or Subgroup	Events	Total	Events	Total	Weight	M-H, Random, 95% CI
Szaflarska-Poplawska 2022	7	48	1	50	100.0%	8.37 [0.99, 70.82]
**Total (95% CI)**		**48**		**50**	**100.0%**	**8.37 [0.99, 70.82]**
Total events	7		1			

Heterogeneity: Not applicable
Test for overall effect: Z = 1.95 (P = 0.05)

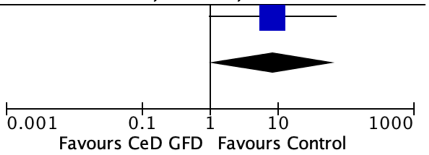

S2C- Forest plot comparison of means of serum Vitamin B1 levels between CeD on GFD with CeD not on GFD in a cohort study

	CeD GFD			CeD non GFD				Mean Difference
Study or Subgroup	Mean	SD	Total	Mean	SD	Total	Weight	IV, Random, 95% CI
McGrogan 2021	496	112	40	231	91.6	24	100.0%	265.00 [214.53, 315.47]
**Total (95% CI)**			**40**			**24**	**100.0%**	**265.00 [214.53, 315.47]**

Heterogeneity: Not applicable
Test for overall effect: Z = 10.29 (P < 0.00001)

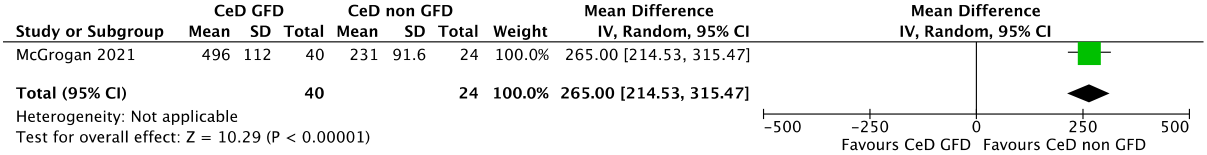

S2D- Forest plot comparison of means of serum Vitamin B1 levels between CeD on GFD with non-CeD controls in a cohort study

	Experimental			Control				Mean Difference
Study or Subgroup	Mean	SD	Total	Mean	SD	Total	Weight	IV, Random, 95% CI
Szaflarska-Poplawska 2022	62.8	12.3	48	69	11.3	50	100.0%	−6.20 [−10.88, −1.52]
**Total (95% CI)**			**48**			**50**	**100.0%**	**−6.20 [−10.88, −1.52]**

Heterogeneity: Not applicable
Test for overall effect: Z = 2.60 (P = 0.009)

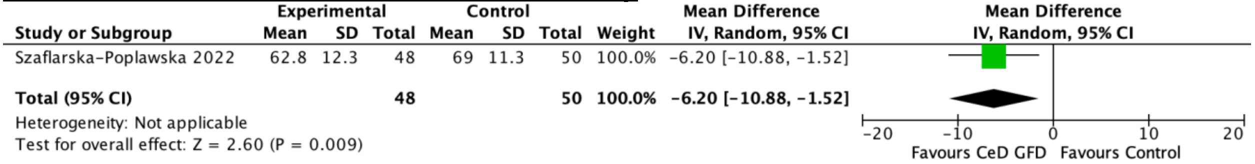

Supplementary Figure S3. Vitamin B2

S3A- Forest plot comparison of Vitamin B2 deficiency between CeD on GFD with non-CeD controls in a cohort study

	CeD GFD		Controls			Odds Ratio
Study or Subgroup	Events	Total	Events	Total	Weight	M-H, Random, 95% CI
Szaflarska-Poplawska 2022	4	48	4	50	100.0%	1.05 [0.25, 4.44]
**Total (95% CI)**		**48**		**50**	**100.0%**	**1.05 [0.25, 4.44]**
Total events	4		4			

Heterogeneity: Not applicable
Test for overall effect: Z = 0.06 (P = 0.95)

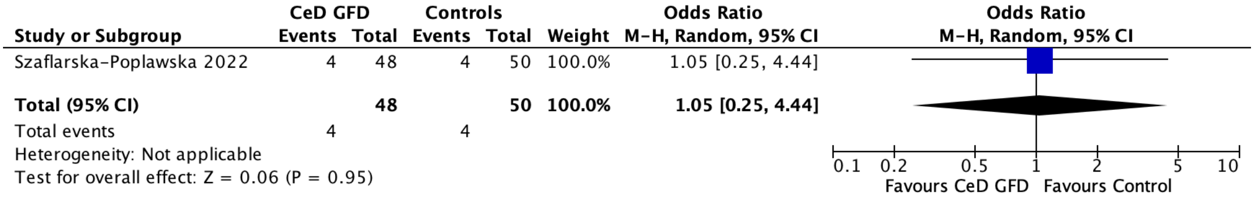

S3B- Forest plot comparison of means of serum Vitamin B2 levels between CeD on GFD with CeD not on GFD in a cohort study

	CeD GFD			CeD non GFD				Mean Difference
Study or Subgroup	Mean	SD	Total	Mean	SD	Total	Weight	IV, Random, 95% CI
McGrogan 2021	2.21	0.34	42	1.96	0.8	24	100.0%	0.25 [−0.09, 0.59]
**Total (95% CI)**			**42**			**24**	**100.0%**	**0.25 [−0.09, 0.59]**

Heterogeneity: Not applicable
Test for overall effect: Z = 1.46 (P = 0.14)

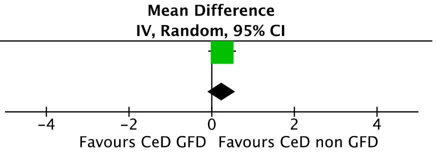

S3C- Forest plot comparison of means of erythrocyte glutathione reductase reactivation coefficient (EGRAC) as a measure of Vitamin B2 levels between CeD on GFD with CeD not on GFD

	CeD GFD			Control				Mean Difference
Study or Subgroup	Mean	SD	Total	Mean	SD	Total	Weight	IV, Random, 95% CI
**76.2.1 CeD GFD vs non GFD**								
Dickey 2008	1.28	0.13	41	1.35	0.19	35	100.0%	−0.07 [−0.14, 0.00]
**Subtotal (95% CI)**			**41**			**35**	**100.0%**	**−0.07 [−0.14, 0.00]**
Heterogeneity: Not applicable								
Test for overall effect: Z = 1.84 (P = 0.07)								
**Total (95% CI)**			**41**			**35**	**100.0%**	**−0.07 [−0.14, 0.00]**

Heterogeneity: Not applicable
Test for overall effect: Z = 1.84 (P = 0.07)
Test for subgroup differences: Not applicable

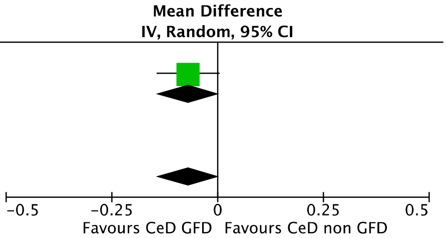

S3D- Forest plot comparison of means of erythrocyte glutathione reductase reactivation coefficient (EGRAC) and HPLC as a measure of Vitamin B2 levels between CeD on GFD with non-CeD controls

	CeD GFD			Controls				Std. Mean Difference
**Study or Subgroup**	**Mean**	**SD**	**Total**	**Mean**	**SD**	**Total**	**Weight**	**IV, Random, 95% CI**
**8.1.1 EGRAC**								
Dickey 2008	1.28	0.13	41	1.36	0.15	200	50.4%	−0.54 [−0.88, −0.20]
**Subtotal (95% CI)**			**41**			**200**	**50.4%**	**−0.54 [−0.88, −0.20]**
Heterogeneity: Not applicable								
Test for overall effect: Z = 3.13 (P = 0.002)								
**8.1.2 HPLC**								
Szaflarska−Poplawska 2022	254.7	47.7	48	226.8	33.4	50	49.6%	0.67 [0.27, 1.08]
**Subtotal (95% CI)**			**48**			**50**	**49.6%**	**0.67 [0.27, 1.08]**
Heterogeneity: Not applicable								
Test for overall effect: Z = 3.24 (P = 0.001)								
**Total (95% CI)**			**89**			**250**	**100.0%**	**0.06 [−1.13, 1.25]**
Heterogeneity: $Tau^2 = 0.70$; $Chi^2 = 20.24$, df = 1 (P < 0.00001); $I^2 = 95\%$								
Test for overall effect: Z = 0.10 (P = 0.92)								
Test for subgroup differences: $Chi^2 = 20.24$, df = 1 (P < 0.00001), $I^2 = 95.1\%$								

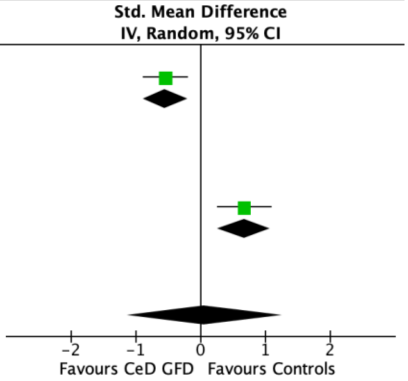

Supplementary Figure S4. Vitamin B6

S4A- Forest plot comparison of Vitamin B6 deficiency between CeD on GFD to CeD not on GFD

	CeD GFD		CeD non GFD			Odds Ratio
**Study or Subgroup**	**Events**	**Total**	**Events**	**Total**	**Weight**	**M−H, Random, 95% CI**
McGrogan 2021	1	40	3	24	100.0%	0.18 [0.02, 1.83]
**Total (95% CI)**		**40**		**24**	**100.0%**	**0.18 [0.02, 1.83]**
Total events	1		3			
Heterogeneity: Not applicable						
Test for overall effect: Z = 1.45 (P = 0.15)						

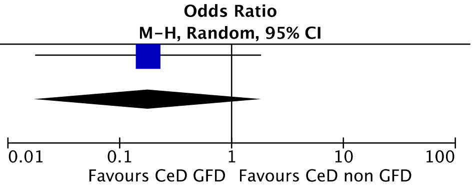

S4B- Forest plot comparison of means of serum Vitamin B6/Hb (pmol/g) between CeD on GFD to CeD not on GFD

	CeD GFD			CeD non GFD				Mean Difference
**Study or Subgroup**	**Mean**	**SD**	**Total**	**Mean**	**SD**	**Total**	**Weight**	**IV, Random, 95% CI**
**9.1.1 Vitamin B6/Hb (pmol/g)**								
McGrogan 2021	455	96.9	40	1,479	936.3	24	100.0%	−1024.00 [−1399.79, −648.21]
**Subtotal (95% CI)**			**40**			**24**	**100.0%**	**−1024.00 [−1399.79, −648.21]**
Heterogeneity: Not applicable								
Test for overall effect: Z = 5.34 (P < 0.00001)								
**Total (95% CI)**			**40**			**24**	**100.0%**	**−1024.00 [−1399.79, −648.21]**
Heterogeneity: Not applicable								
Test for overall effect: Z = 5.34 (P < 0.00001)								
Test for subgroup differences: Not applicable								

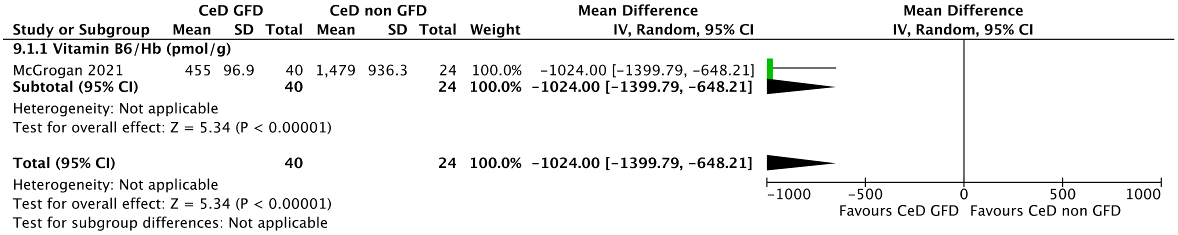

S4C- Forest plot comparison of means of serum of pyridoxal phosphate (PALP) as a surrogate of Vitamin B6 levels between CeD on GFD to CeD not on GFD

Study or Subgroup	CeD GFD Mean	CeD GFD SD	CeD GFD Total	CeD non GFD Mean	CeD non GFD SD	CeD non GFD Total	Weight	Mean Difference IV, Random, 95% CI
**39.1.2 PALP (ng/ml)**								
Reinken 1976	6.1	3.88	6	1.99	1.53	14	100.0%	4.11 [0.90, 7.32]
**Subtotal (95% CI)**			**6**			**14**	**100.0%**	**4.11 [0.90, 7.32]**
Heterogeneity: Not applicable								
Test for overall effect: Z = 2.51 (P = 0.01)								
**Total (95% CI)**			**6**			**14**	**100.0%**	**4.11 [0.90, 7.32]**
Heterogeneity: Not applicable								
Test for overall effect: Z = 2.51 (P = 0.01)								
Test for subgroup differences: Not applicable								

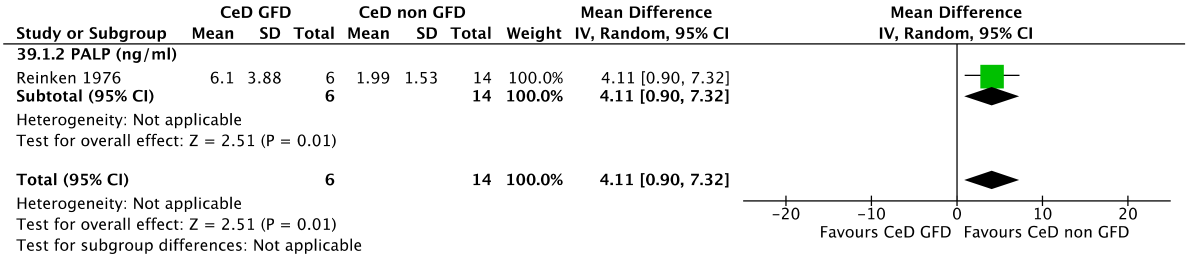

S4D- Forest plot comparison of levels of pyridoxal phosphate (PALP), PLP activity. and HPLC between CeD on GFD and non-CeD controls

Study or Subgroup	CeD GFD Mean	CeD GFD SD	CeD GFD Total	Controls Mean	Controls SD	Controls Total	Weight	Mean Difference IV, Random, 95% CI
**9.1.1 PALP (ng/ml)**								
Reinken 1976	6.1	3.88	48	6.35	2.51	20	85.0%	−0.25 [−1.80, 1.30]
**Subtotal (95% CI)**			**48**			**20**	**85.0%**	**−0.25 [−1.80, 1.30]**
Heterogeneity: Not applicable								
Test for overall effect: Z = 0.32 (P = 0.75)								
**9.1.2 pyridoxal-5-phosphate (PLP)-nmol/L**								
Valente 2015	130.3	35.8	20	130.3	52.3	39	0.4%	0.00 [−22.71, 22.71]
**Subtotal (95% CI)**			**20**			**39**	**0.4%**	**0.00 [−22.71, 22.71]**
Heterogeneity: Not applicable								
Test for overall effect: Z = 0.00 (P = 1.00)								
**9.1.3 HPLC**								
Szaflarska-Poplawska 2022	30	8.1	48	29.9	10.7	50	14.6%	0.10 [−3.65, 3.85]
**Subtotal (95% CI)**			**48**			**50**	**14.6%**	**0.10 [−3.65, 3.85]**
Heterogeneity: Not applicable								
Test for overall effect: Z = 0.05 (P = 0.96)								
**Total (95% CI)**			**116**			**109**	**100.0%**	**−0.20 [−1.63, 1.23]**
Heterogeneity: $Tau^2 = 0.00$; $Chi^2 = 0.03$, df = 2 (P = 0.99); $I^2 = 0\%$								
Test for overall effect: Z = 0.27 (P = 0.79)								
Test for subgroup differences: $Chi^2 = 0.03$, df = 2 (P = 0.99), $I^2 = 0\%$								

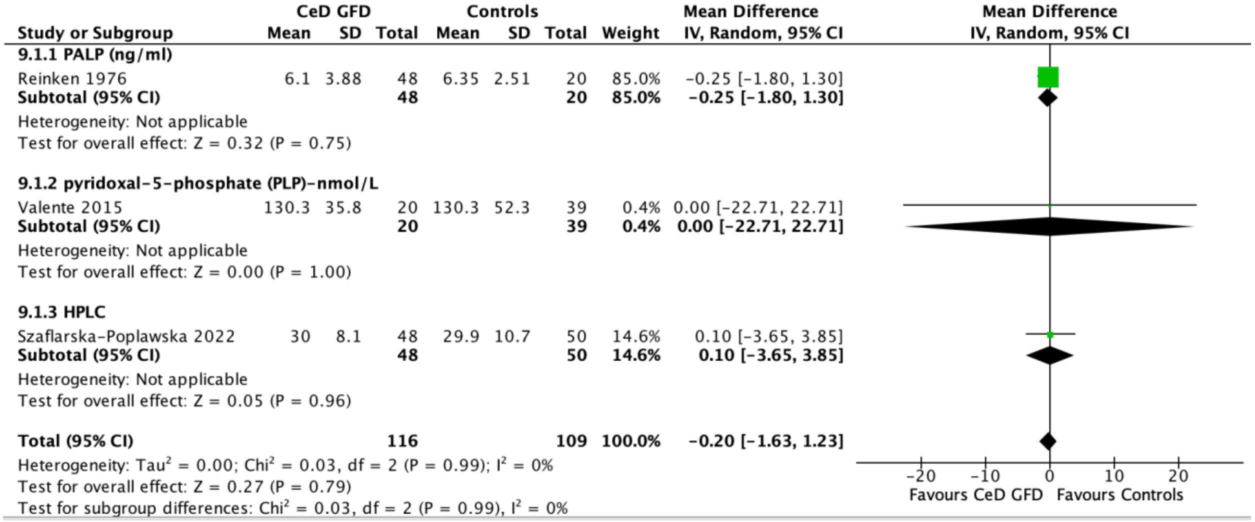

Supplementary Figure S5. Vitamin B12

S5A- Forest plot comparison of Vitamin B12 deficiency between CeD on GFD to CeD not on GFD

Study or Subgroup	CeD GFD Events	CeD GFD Total	CeD non GFD Events	CeD non GFD Total	Weight	Odds Ratio M-H, Random, 95% CI
Kemppainen 1995	0	52	6	40	51.1%	0.05 [0.00, 0.93]
McGrogan 2021	0	42	0	23		Not estimable
Selbuz 2021	0	21	4	32	48.9%	0.15 [0.01, 2.89]
**Total (95% CI)**		**115**		**95**	**100.0%**	**0.09 [0.01, 0.68]**
Total events	0		10			
Heterogeneity: $Tau^2 = 0.00$; $Chi^2 = 0.26$, df = 1 (P = 0.61); $I^2 = 0\%$						
Test for overall effect: Z = 2.32 (P = 0.02)						

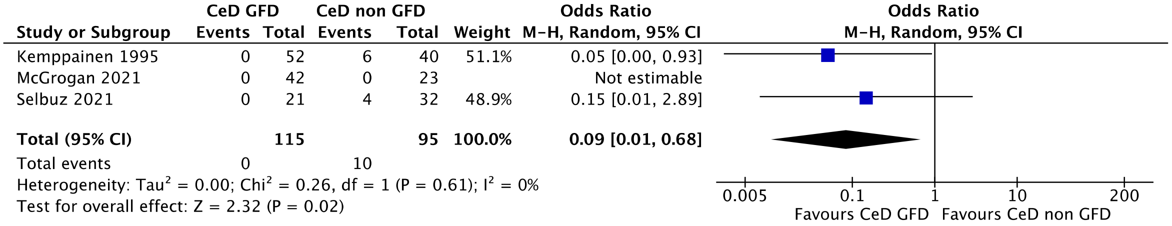

S5B- Forest plot comparison of Vitamin B12 deficiency between CeD on GFD to non-CeD controls

Study or Subgroup	CeD GFD Events	CeD GFD Total	Controls Events	Controls Total	Weight	Odds Ratio M-H, Random, 95% CI
Bayrak 2020	20	103	27	135	37.2%	0.96 [0.51, 1.84]
Mansueto 2023	62	174	2	124	34.7%	33.77 [8.07, 141.28]
Szaflarska-Poplawska 2022	1	48	1	50	28.0%	1.04 [0.06, 17.15]
**Total (95% CI)**		**325**		**309**	**100.0%**	**3.39 [0.19, 61.78]**
Total events	83		30			

Heterogeneity: $Tau^2 = 5.78$; $Chi^2 = 24.70$, df = 2 ($P < 0.00001$); $I^2 = 92\%$
Test for overall effect: Z = 0.82 (P = 0.41)

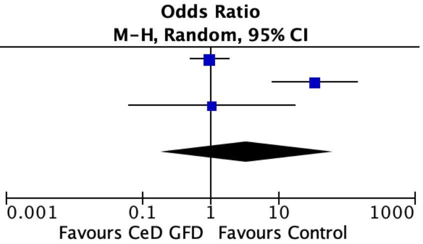

S5C- Forest plot comparison of means of serum Vitamin B12 levels between CeD on GFD to CeD not on GFD. Of note all studies are short term GFD.

Study or Subgroup	CeD GFD Mean	CeD GFD SD	CeD GFD Total	CeD non GFD Mean	CeD non GFD SD	CeD non GFD Total	Weight	Mean Difference IV, Random, 95% CI
Dickey 2008	276.6	137.1	41	248.6	170.9	35	26.1%	28.00 [-42.48, 98.48]
Kemppainen 1995	412	141	51	314	148	37	34.3%	98.00 [36.59, 159.41]
McGrogan 2021	509.96	216.23	42	443.54	160.15	23	15.1%	66.42 [-26.10, 158.94]
Volkan 2018	319.74	144.83	21	246.86	98.89	26	24.5%	72.88 [0.20, 145.56]
**Total (95% CI)**			**155**			**121**	**100.0%**	**68.83 [32.85, 104.80]**

Heterogeneity: $Tau^2 = 0.00$; $Chi^2 = 2.17$, df = 3 (P = 0.54); $I^2 = 0\%$
Test for overall effect: Z = 3.75 (P = 0.0002)

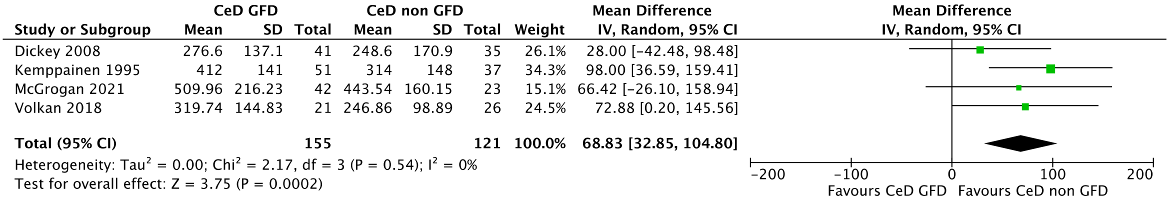

S5D- Forest plot comparison of means of serum Vitamin B12 levels between CeD on GFD and non-CeD controls

Study or Subgroup	CeD GFD Mean	CeD GFD SD	CeD GFD Total	Controls Mean	Controls SD	Controls Total	Weight	Mean Difference IV, Random, 95% CI
Bayrak 2020	267.58	141.49	103	268.04	150.68	135	12.7%	-0.46 [-37.78, 36.86]
Dickey 2008	276.6	137.1	41	274.1	104.6	200	11.0%	2.50 [-41.90, 46.90]
Efe 2023	371.3	14.3	50	354.4	10.7	72	20.4%	16.90 [12.23, 21.57]
Isikay 2018	259.86	91.57	226	241.73	91.75	268	18.5%	18.13 [1.91, 34.35]
Mansueto 2023	320	153.5	174	394.3	124.8	124	14.3%	-74.30 [-105.97, -42.63]
Szaflarska-Poplawska 2022	415.3	157.6	48	479.7	246.4	50	5.2%	-64.40 [-145.96, 17.16]
Valente 2015	200.07	65.68	20	189.89	66.86	39	13.2%	10.18 [-25.44, 45.80]
Volkan 2018	319.74	144.83	21	290.03	168.26	30	4.8%	29.71 [-56.67, 116.09]
**Total (95% CI)**			**683**			**918**	**100.0%**	**-4.18 [-25.72, 17.36]**

Heterogeneity: $Tau^2 = 586.00$; $Chi^2 = 36.08$, df = 7 ($P < 0.00001$); $I^2 = 81\%$
Test for overall effect: Z = 0.38 (P = 0.70)

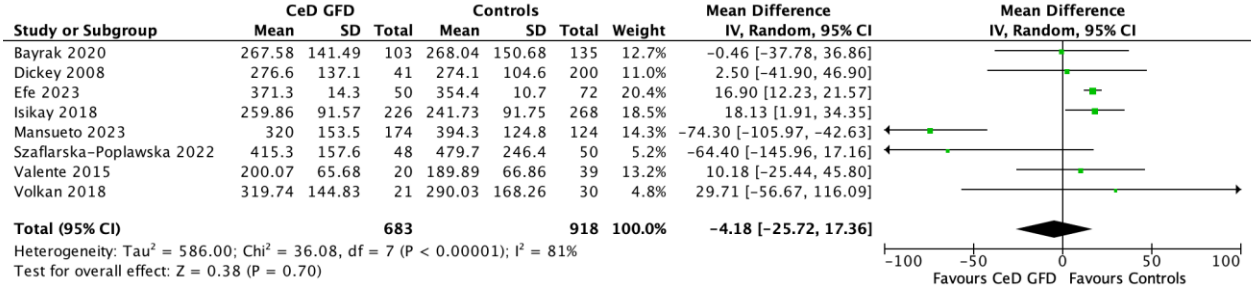

S5E- Forest plot comparison of means of serum Vitamin B12 levels between CeD on GFD and non-CeD controls based on study design

Study or Subgroup	CeD GFD Mean	CeD GFD SD	CeD GFD Total	Controls Mean	Controls SD	Controls Total	Weight	Mean Difference IV, Random, 95% CI
**22.1.1 Cohort**								
Szaflarska-Poplawska 2022	415.3	157.6	48	479.7	246.4	50	5.2%	-64.40 [-145.96, 17.16]
**Subtotal (95% CI)**			**48**			**50**	**5.2%**	**-64.40 [-145.96, 17.16]**
Heterogeneity: Not applicable								
Test for overall effect: Z = 1.55 (P = 0.12)								
**22.1.2 Cross-sectional**								
Bayrak 2020	267.58	141.49	103	268.04	150.68	135	12.7%	-0.46 [-37.78, 36.86]
Isikay 2018	259.86	91.57	226	241.73	91.75	268	18.5%	18.13 [1.91, 34.35]
Valente 2015	200.07	65.68	20	189.89	66.86	39	13.2%	10.18 [-25.44, 45.80]
**Subtotal (95% CI)**			**349**			**442**	**44.4%**	**14.43 [0.70, 28.16]**
Heterogeneity: $Tau^2 = 0.00$; $Chi^2 = 0.87$, df = 2 (P = 0.65); $I^2 = 0\%$								
Test for overall effect: Z = 2.06 (P = 0.04)								
**22.1.3 Case- Control**								
Dickey 2008	276.6	137.1	41	274.1	104.6	200	11.0%	2.50 [-41.90, 46.90]
Efe 2023	371.3	14.3	50	354.4	10.7	72	20.4%	16.90 [12.23, 21.57]
Mansueto 2023	320	153.5	174	394.3	124.8	124	14.3%	-74.30 [-105.97, -42.63]
Volkan 2018	319.74	144.83	21	290.03	168.26	30	4.8%	29.71 [-56.67, 116.09]
**Subtotal (95% CI)**			**286**			**426**	**50.4%**	**-9.72 [-62.11, 42.67]**
Heterogeneity: $Tau^2 = 2330.99$; $Chi^2 = 31.60$, df = 3 (P < 0.00001); $I^2 = 91\%$								
Test for overall effect: Z = 0.36 (P = 0.72)								
**Total (95% CI)**			**683**			**918**	**100.0%**	**-4.18 [-25.72, 17.36]**
Heterogeneity: $Tau^2 = 586.00$; $Chi^2 = 36.08$, df = 7 (P < 0.00001); $I^2 = 81\%$								
Test for overall effect: Z = 0.38 (P = 0.70)								
Test for subgroup differences: $Chi^2 = 4.12$, df = 2 (P = 0.13), $I^2 = 51.5\%$								

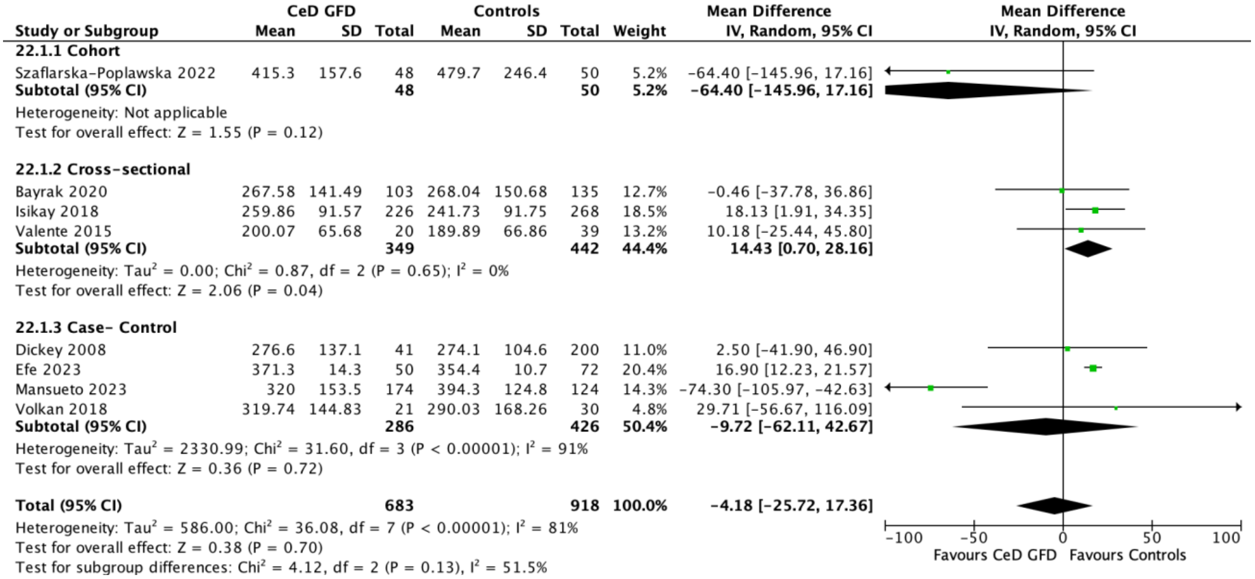

S5F- Forest plot comparison of means of serum Vitamin B12 levels between CeD on GFD and non-CeD controls based on GFD length

Study or Subgroup	CeD GFD Mean	CeD GFD SD	CeD GFD Total	Controls Mean	Controls SD	Controls Total	Weight	Mean Difference IV, Random, 95% CI
**23.1.1 Short-term GFD**								
Efe 2023	371.3	14.3	50	354.4	10.7	72	22.4%	16.90 [12.23, 21.57]
Isikay 2018	259.86	91.57	226	241.73	91.75	268	20.4%	18.13 [1.91, 34.35]
Mansueto 2023	320	153.5	174	394.3	124.8	124	16.1%	-74.30 [-105.97, -42.63]
Valente 2015	200.07	65.68	20	189.89	66.86	39	14.9%	10.18 [-25.44, 45.80]
Volkan 2018	319.74	144.83	21	290.03	168.26	30	5.6%	29.71 [-56.67, 116.09]
**Subtotal (95% CI)**			**491**			**533**	**79.4%**	**-1.67 [-28.81, 25.48]**
Heterogeneity: $Tau^2 = 682.49$; $Chi^2 = 31.51$, df = 4 (P < 0.00001); $I^2 = 87\%$								
Test for overall effect: Z = 0.12 (P = 0.90)								
**23.1.2 Long-term GFD**								
Bayrak 2020	267.58	141.49	103	268.04	150.68	135	14.5%	-0.46 [-37.78, 36.86]
Szaflarska-Poplawska 2022	415.3	157.6	48	479.7	246.4	50	6.1%	-64.40 [-145.96, 17.16]
**Subtotal (95% CI)**			**151**			**185**	**20.6%**	**-21.72 [-80.77, 37.32]**
Heterogeneity: $Tau^2 = 997.03$; $Chi^2 = 1.95$, df = 1 (P = 0.16); $I^2 = 49\%$								
Test for overall effect: Z = 0.72 (P = 0.47)								
**Total (95% CI)**			**642**			**718**	**100.0%**	**-5.28 [-28.93, 18.37]**
Heterogeneity: $Tau^2 = 644.74$; $Chi^2 = 35.79$, df = 6 (P < 0.00001); $I^2 = 83\%$								
Test for overall effect: Z = 0.44 (P = 0.66)								
Test for subgroup differences: $Chi^2 = 0.37$, df = 1 (P = 0.55), $I^2 = 0\%$								

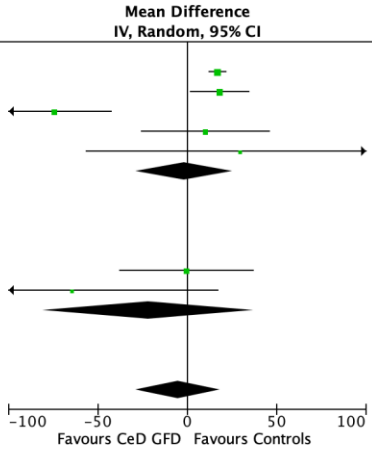

S5G- Forest plot comparison of means of serum Vitamin B12 levels between CeD on GFD and non-CeD controls based on population

Study or Subgroup	CeD GFD Mean	CeD GFD SD	CeD GFD Total	Controls Mean	Controls SD	Controls Total	Weight	Mean Difference IV, Random, 95% CI
**31.1.2 Children**								
Bayrak 2020	267.58	141.49	103	268.04	150.68	135	12.7%	-0.46 [-37.78, 36.86]
Efe 2023	371.3	14.3	50	354.4	10.7	72	20.4%	16.90 [12.23, 21.57]
Isikay 2018	259.86	91.57	226	241.73	91.75	268	18.5%	18.13 [1.91, 34.35]
Mansueto 2023	320	153.5	174	394.2	124.8	124	14.3%	-74.20 [-105.87, -42.53]
Volkan 2018	319.74	144.83	21	290.03	168.26	30	4.8%	29.71 [-56.67, 116.09]
**Subtotal (95% CI)**			**574**			**629**	**70.6%**	**-3.72 [-31.28, 23.84]**
Heterogeneity: $Tau^2 = 703.83$; $Chi^2 = 32.03$, df = 4 (P < 0.00001); $I^2 = 88\%$								
Test for overall effect: Z = 0.26 (P = 0.79)								
**31.1.3 Adults**								
Dickey 2008	276.6	137.1	41	274.1	104.6	200	11.0%	2.50 [-41.90, 46.90]
Szaflarska-Poplawska 2022	415.3	157.6	48	479.7	246.4	50	5.2%	-64.40 [-145.96, 17.16]
Valente 2015	200.07	65.68	20	189.89	66.86	39	13.2%	10.18 [-25.44, 45.80]
**Subtotal (95% CI)**			**109**			**289**	**29.4%**	**-3.08 [-35.52, 29.36]**
Heterogeneity: $Tau^2 = 228.03$; $Chi^2 = 2.72$, df = 2 (P = 0.26); $I^2 = 26\%$								
Test for overall effect: Z = 0.19 (P = 0.85)								
**Total (95% CI)**			**683**			**918**	**100.0%**	**-4.16 [-25.68, 17.36]**
Heterogeneity: $Tau^2 = 584.62$; $Chi^2 = 36.01$, df = 7 (P < 0.00001); $I^2 = 81\%$								
Test for overall effect: Z = 0.38 (P = 0.70)								
Test for subgroup differences: $Chi^2 = 0.00$, df = 1 (P = 0.98), $I^2 = 0\%$								

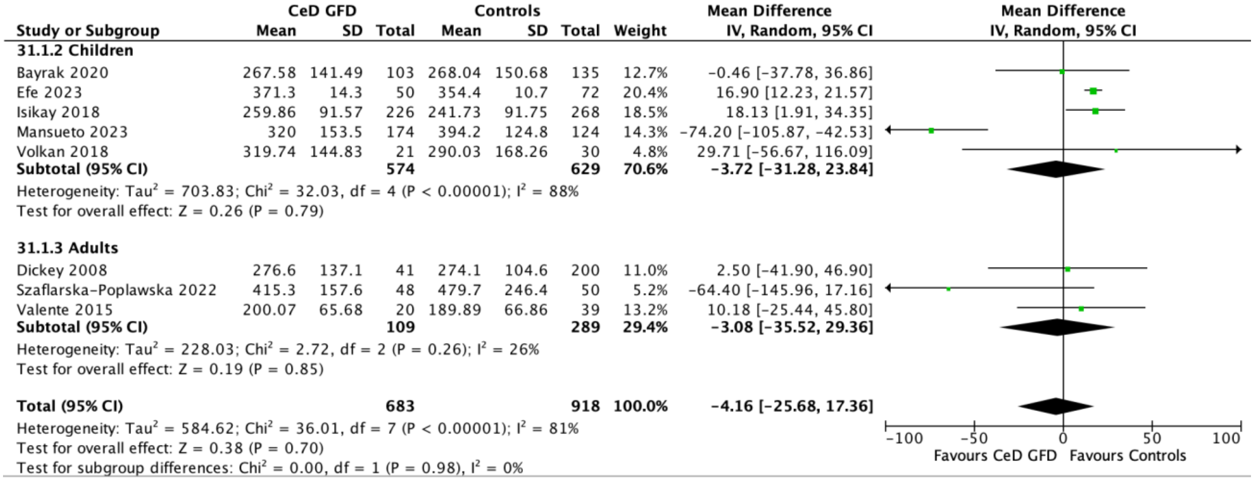

Supplementary Figure S6. Vitamin D

S6Ai- Forest plot comparison of Vitamin D deficiency between CeD on GFD and CeD not on GFD

	CeD GFD		CeD CeD non GFD			Odds Ratio
**Study or Subgroup**	**Events**	**Total**	**Events**	**Total**	**Weight**	**M-H, Random, 95% CI**
Ciacci 2020	15	55	25	50	13.1%	0.38 [0.17, 0.84]
González 1995	0	12	1	20	2.4%	0.52 [0.02, 13.80]
Keaveny 1996	4	16	11	19	7.9%	0.24 [0.06, 1.04]
Klimov 2017	30	37	16	22	9.3%	1.61 [0.46, 5.60]
Margoni 2012	12	36	16	45	12.0%	0.91 [0.36, 2.28]
Mazure 1994	0	6	2	18	2.5%	0.51 [0.02, 12.08]
McGrogan 2021	25	44	5	25	10.1%	5.26 [1.67, 16.58]
Piatek-Guziewicz 2017, May	61	92	37	53	13.9%	0.85 [0.41, 1.76]
Piatek-Guziewicz 2017, Nov	21	31	26	29	8.2%	0.24 [0.06, 1.00]
Selbuz 2021	11	20	23	31	9.8%	0.43 [0.13, 1.40]
Uyanikoglu 2021	32	40	31	40	10.7%	1.16 [0.40, 3.40]
**Total (95% CI)**		**389**		**352**	**100.0%**	**0.76 [0.44, 1.30]**
Total events	211		193			

Heterogeneity: $Tau^2 = 0.41$; $Chi^2 = 21.98$, df = 10 (P = 0.02); $I^2 = 55\%$
Test for overall effect: Z = 1.01 (P = 0.31)

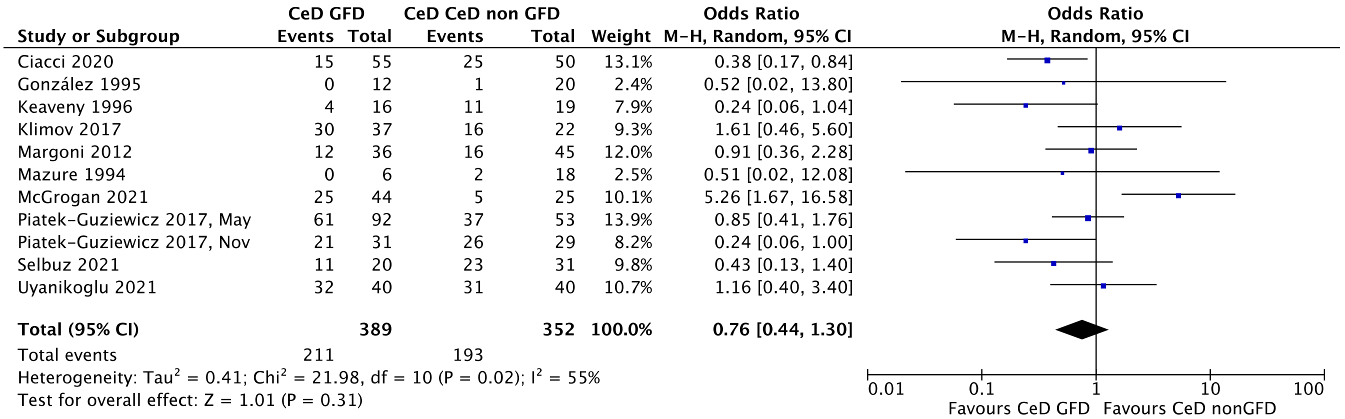

S6Aii- Funnel plot of studies within Ai comparing Vitamin D deficiency between CeD on GFD and CeD not on GFD

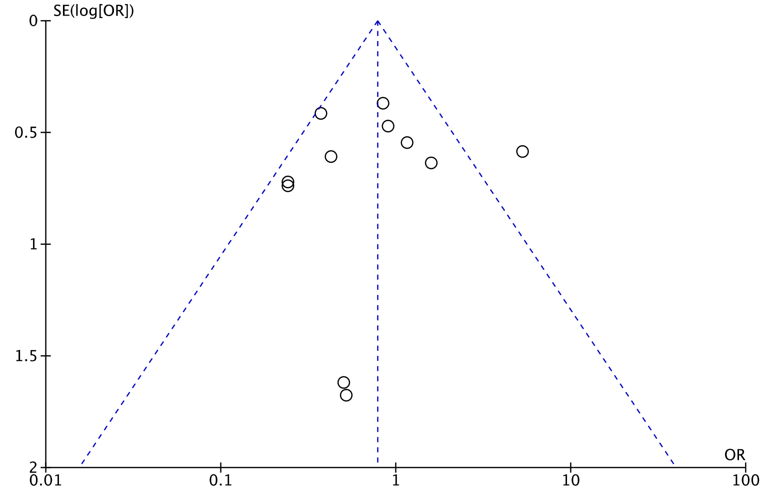

S6B- Forest plot comparison of Vitamin D deficiency between CeD on GFD and CeD not on GFD by study design

Study or Subgroup	CeD GFD Events	CeD GFD Total	CeD CeD non GFD Events	CeD CeD non GFD Total	Weight	Odds Ratio M-H, Random, 95% CI
**1.1.1 Cohort**						
Keaveny 1996	4	16	11	19	7.9%	0.24 [0.06, 1.04]
Margoni 2012	12	36	16	45	12.0%	0.91 [0.36, 2.28]
McGrogan 2021	25	44	5	25	10.1%	5.26 [1.67, 16.58]
**Subtotal (95% CI)**		**96**		**89**	**30.1%**	**1.10 [0.22, 5.41]**
Total events	41		32			
Heterogeneity: Tau^2 = 1.63; Chi^2 = 11.40, df = 2 (P = 0.003); I^2 = 82%						
Test for overall effect: Z = 0.11 (P = 0.91)						
**1.1.2 Cross-sectional**						
González 1995	0	12	1	20	2.4%	0.52 [0.02, 13.80]
**Subtotal (95% CI)**		**12**		**20**	**2.4%**	**0.52 [0.02, 13.80]**
Total events	0		1			
Heterogeneity: Not applicable						
Test for overall effect: Z = 0.39 (P = 0.70)						
**1.1.3 Case-Control**						
Ciacci 2020	15	55	25	50	13.1%	0.38 [0.17, 0.84]
Klimov 2017	30	37	16	22	9.3%	1.61 [0.46, 5.60]
Mazure 1994	0	6	2	18	2.5%	0.51 [0.02, 12.08]
Piatek-Guziewicz 2017, May	61	92	37	53	13.9%	0.85 [0.41, 1.76]
Piatek-Guziewicz 2017, Nov	21	31	26	29	8.2%	0.24 [0.06, 1.00]
Selbuz 2021	11	20	23	31	9.8%	0.43 [0.13, 1.40]
Uyanikoglu 2021	32	40	31	40	10.7%	1.16 [0.40, 3.40]
**Subtotal (95% CI)**		**281**		**243**	**67.5%**	**0.64 [0.40, 1.04]**
Total events	170		160			
Heterogeneity: Tau^2 = 0.10; Chi^2 = 7.82, df = 6 (P = 0.25); I^2 = 23%						
Test for overall effect: Z = 1.82 (P = 0.07)						
**Total (95% CI)**		**389**		**352**	**100.0%**	**0.76 [0.44, 1.30]**
Total events	211		193			
Heterogeneity: Tau^2 = 0.41; Chi^2 = 21.98, df = 10 (P = 0.02); I^2 = 55%						
Test for overall effect: Z = 1.01 (P = 0.31)						
Test for subgroup differences: Chi^2 = 0.42, df = 2 (P = 0.81), I^2 = 0%						

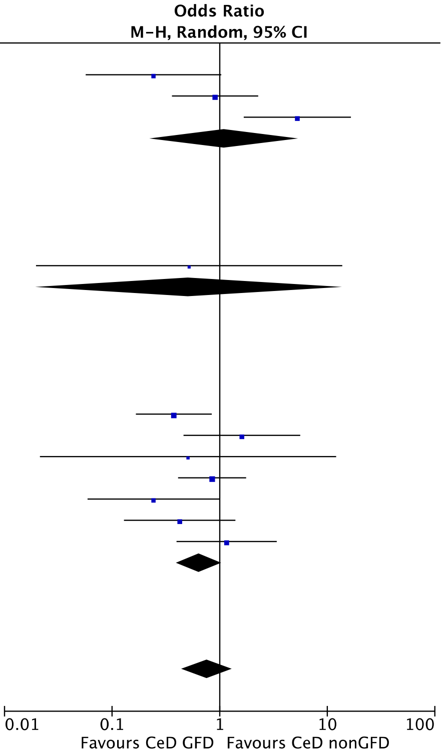

S6C- Forest plot comparison of Vitamin D deficiency between CeD on GFD and CeD not on GFD by population

Study or Subgroup	CeD GFD Events	CeD GFD Total	CeD CeD non GFD Events	CeD CeD non GFD Total	Weight	Odds Ratio M-H, Random, 95% CI
**36.1.1 Children**						
Klimov 2017	30	37	16	22	9.3%	1.61 [0.46, 5.60]
Margoni 2012	12	36	16	45	12.0%	0.91 [0.36, 2.28]
McGrogan 2021	25	44	5	25	10.1%	5.26 [1.67, 16.58]
Selbuz 2021	11	20	23	31	9.8%	0.43 [0.13, 1.40]
**Subtotal (95% CI)**		**137**		**123**	**41.3%**	**1.33 [0.48, 3.67]**
Total events	78		60			
Heterogeneity: Tau^2 = 0.74; Chi^2 = 9.80, df = 3 (P = 0.02); I^2 = 69%						
Test for overall effect: Z = 0.56 (P = 0.58)						
**36.1.3 Adults**						
Ciacci 2020	15	55	25	50	13.1%	0.38 [0.17, 0.84]
González 1995	0	12	1	20	2.4%	0.52 [0.02, 13.80]
Keaveny 1996	4	16	11	19	7.9%	0.24 [0.06, 1.04]
Mazure 1994	0	6	2	18	2.5%	0.51 [0.02, 12.08]
Piatek-Guziewicz 2017, May	61	92	37	53	13.9%	0.85 [0.41, 1.76]
Piatek-Guziewicz 2017, Nov	21	31	26	29	8.2%	0.24 [0.06, 1.00]
Uyanikoglu 2021	32	40	31	40	10.7%	1.16 [0.40, 3.40]
**Subtotal (95% CI)**		**252**		**229**	**58.7%**	**0.55 [0.34, 0.87]**
Total events	133		133			
Heterogeneity: Tau^2 = 0.04; Chi^2 = 6.61, df = 6 (P = 0.36); I^2 = 9%						
Test for overall effect: Z = 2.52 (P = 0.01)						
**Total (95% CI)**		**389**		**352**	**100.0%**	**0.76 [0.44, 1.30]**
Total events	211		193			
Heterogeneity: Tau^2 = 0.41; Chi^2 = 21.98, df = 10 (P = 0.02); I^2 = 55%						
Test for overall effect: Z = 1.01 (P = 0.31)						
Test for subgroup differences: Chi^2 = 2.44, df = 1 (P = 0.12), I^2 = 59.1%						

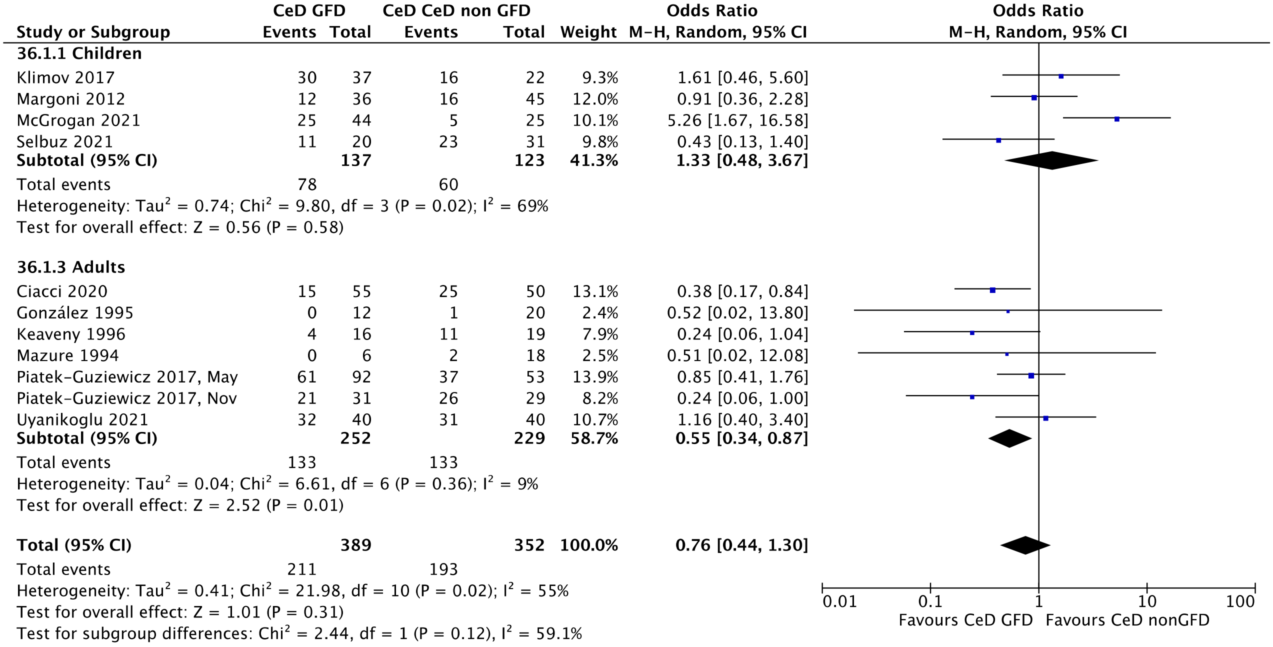

S6D- Forest plot comparison of Vitamin D deficiency between CeD on GFD and non-CeD controls by population

Study or Subgroup	CeD GFD Events	CeD GFD Total	Control Events	Control Total	Weight	Odds Ratio M-H, Random, 95% CI
**51.1.1 Children**						
Bayrak 2020	97	103	108	135	18.5%	4.04 [1.60, 10.20]
Klimov 2017	30	37	0	14	6.1%	117.93 [6.30, 2209.02]
**Subtotal (95% CI)**		**140**		**149**	**24.5%**	**16.47 [0.58, 470.17]**
Total events	127		108			
Heterogeneity: Tau² = 4.79; Chi² = 4.90, df = 1 (P = 0.03); I² = 80%						
Test for overall effect: Z = 1.64 (P = 0.10)						
**51.1.2 Adults**						
Ballestero-Fernández 2021	22	64	31	74	20.5%	0.73 [0.36, 1.45]
Piatek-Guziewicz 2017, May	61	92	18	52	20.3%	3.72 [1.82, 7.61]
Piatek-Guziewicz 2017, Nov	21	31	14	25	17.0%	1.65 [0.55, 4.91]
Uyanikoglu 2021	32	40	26	40	17.7%	2.15 [0.78, 5.92]
**Subtotal (95% CI)**		**227**		**191**	**75.5%**	**1.74 [0.78, 3.89]**
Total events	136		89			
Heterogeneity: Tau² = 0.47; Chi² = 10.58, df = 3 (P = 0.01); I² = 72%						
Test for overall effect: Z = 1.36 (P = 0.18)						
**Total (95% CI)**		**367**		**340**	**100.0%**	**2.64 [1.14, 6.08]**
Total events	263		197			
Heterogeneity: Tau² = 0.76; Chi² = 21.29, df = 5 (P = 0.0007); I² = 77%						
Test for overall effect: Z = 2.27 (P = 0.02)						
Test for subgroup differences: Chi² = 1.63, df = 1 (P = 0.20), I² = 38.7%						

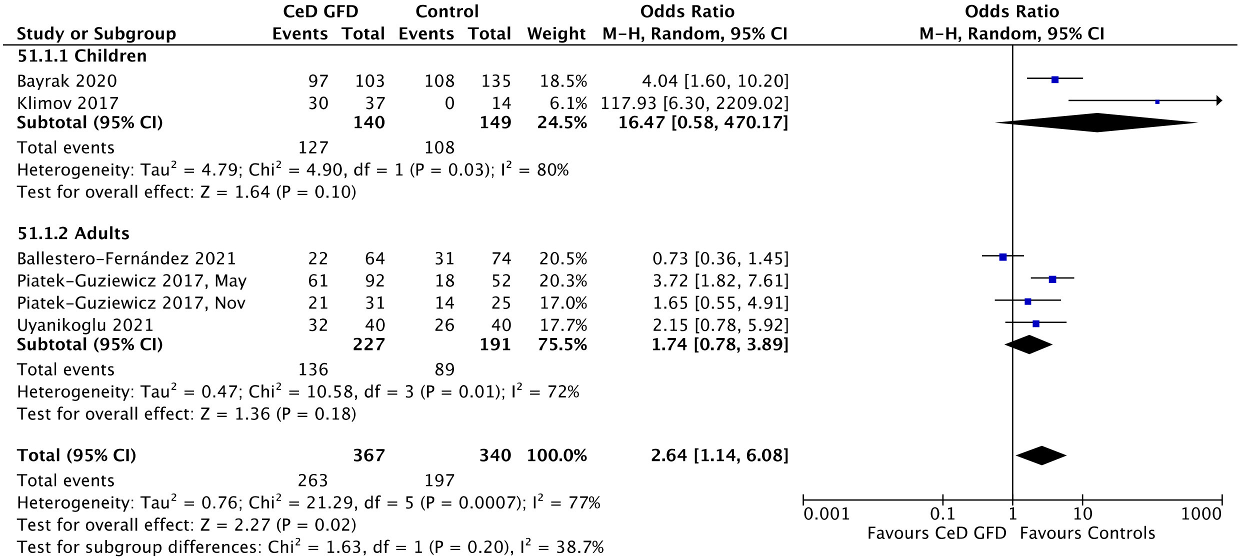

S6Ei- Forest plot comparison of means of serum Vitamin D levels between CeD on GFD and CeD not on GFD

Study or Subgroup	CeD GFD Mean	CeD GFD SD	CeD GFD Total	CeD non GFD Mean	CeD non GFD SD	CeD non GFD Total	Weight	Mean Difference IV, Fixed, 95% CI
Bulut, 2023	23.04	1.19	31	20.53	2.75	18	49.6%	2.51 [1.17, 3.85]
Corazza 1995	26.5	10.5	14	15.5	6	17	2.3%	11.00 [4.80, 17.20]
González 1995	26	11	12	23	13	20	1.2%	3.00 [-5.44, 11.44]
Kavak 2003	31.95	16.03	28	30.1	15.9	34	1.4%	1.85 [-6.14, 9.84]
Keaveny 1996	27.36	2.8	16	25.36	4.5	19	14.8%	2.00 [-0.44, 4.44]
Klimov 2017	22.5	9.19	37	21.7	13.93	22	2.1%	0.80 [-5.73, 7.33]
Margoni 2012	29.3	17.3	36	24.8	10.5	45	2.1%	4.50 [-1.93, 10.93]
Mazure 1994	25.8	5.6	14	22.7	3.5	20	8.1%	3.10 [-0.21, 6.41]
McGrogan 2021	20.32	7.24	40	24.4	9.25	25	4.9%	-4.08 [-8.34, 0.18]
Piatek-Guziewicz 2017, May	24.4	10.8	92	20.8	10.3	53	7.1%	3.60 [0.06, 7.14]
Piatek-Guziewicz 2017, Nov	25.2	11.2	31	19.4	9	21	2.9%	5.80 [0.29, 11.31]
Szymczak 2012	37.8	13.8	19	19.9	18.8	16	0.7%	17.90 [6.79, 29.01]
Uyanikoglu 2021	129.82	84.42	40	134.33	44.35	50	0.1%	-4.51 [-33.42, 24.40]
Volkan 2018	19.76	10.67	21	17.88	9.19	26	2.7%	1.88 [-3.89, 7.65]
**Total (95% CI)**			**431**			**386**	**100.0%**	**2.62 [1.68, 3.56]**
Heterogeneity: Chi² = 26.68, df = 13 (P = 0.01); I² = 51%								
Test for overall effect: Z = 5.45 (P < 0.00001)								

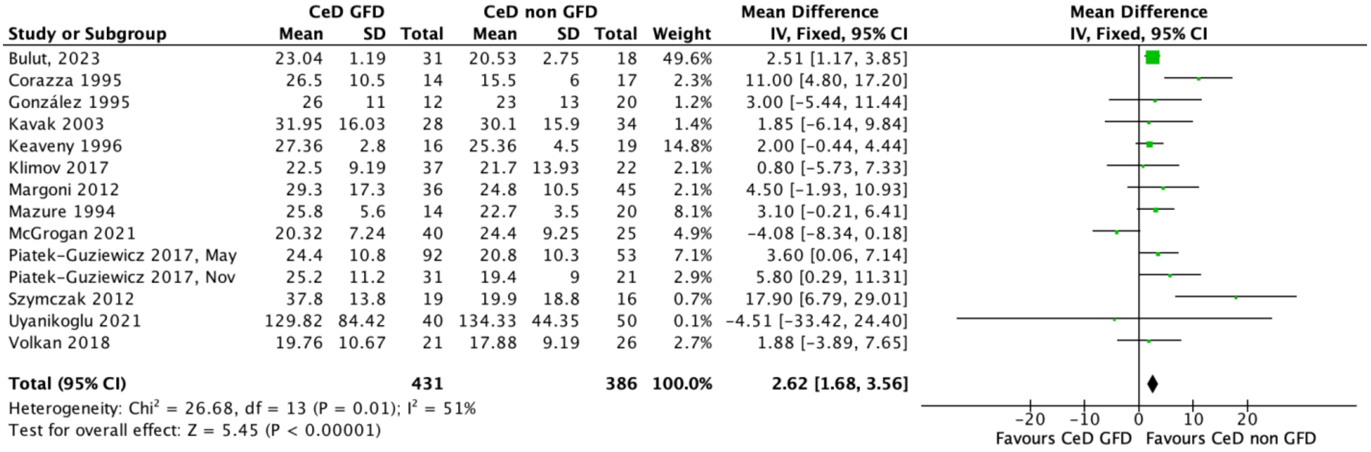

S6Eii- Funnel plot of studies within Fi comparing Vitamin D serum levels between CeD on GFD and CeD not on GFD

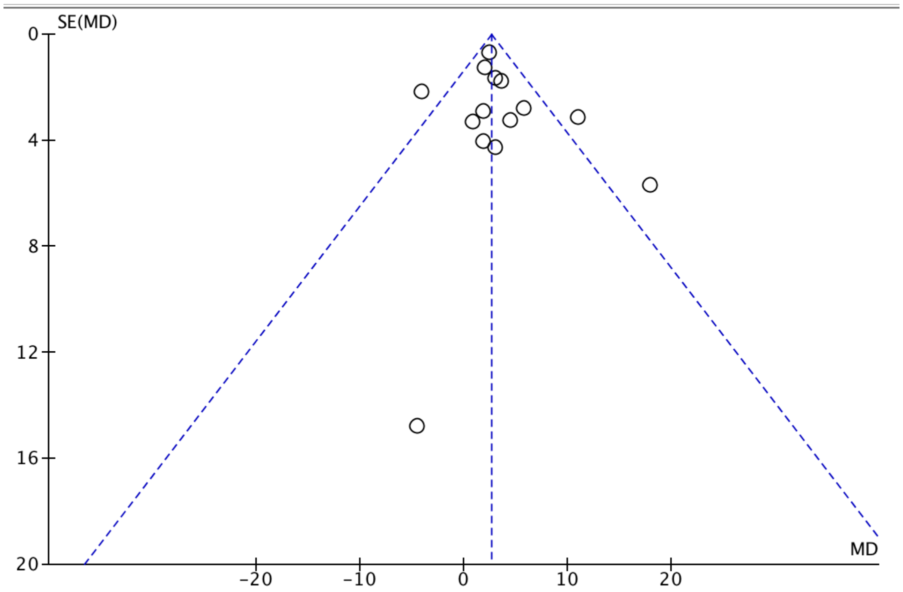

S6F- Forest plot comparison of means of serum Vitamin D levels between CeD on GFD and CeD not on GFD by study design

Study or Subgroup	CeD GFD Mean	SD	Total	CeD non GFD Mean	SD	Total	Weight	Mean Difference IV, Random, 95% CI
**21.1.2 Cohort**								
Keaveny 1996	27.36	2.8	16	25.36	4.5	19	13.6%	2.00 [−0.44, 4.44]
Margoni 2012	29.3	17.3	36	24.8	10.5	45	5.4%	4.50 [−1.93, 10.93]
McGrogan 2021	20.32	7.24	40	24.4	9.25	25	8.9%	−4.08 [−8.34, 0.18]
**Subtotal (95% CI)**			**92**			**89**	**27.9%**	**0.57 [−4.01, 5.15]**
Heterogeneity: $Tau^2 = 11.49$; $Chi^2 = 7.22$, df = 2 (P = 0.03); $I^2 = 72\%$								
Test for overall effect: Z = 0.24 (P = 0.81)								
**21.1.3 Cross-sectional**								
Corazza 1995	26.5	10.5	14	15.5	6	17	5.6%	11.00 [4.80, 17.20]
González 1995	26	11	12	23	13	20	3.5%	3.00 [−5.44, 11.44]
**Subtotal (95% CI)**			**26**			**37**	**9.2%**	**7.53 [−0.24, 15.30]**
Heterogeneity: $Tau^2 = 17.74$; $Chi^2 = 2.24$, df = 1 (P = 0.13); $I^2 = 55\%$								
Test for overall effect: Z = 1.90 (P = 0.06)								
**21.1.4 Case-Control**								
Bulut, 2023	23.04	1.19	31	20.53	2.75	18	16.7%	2.51 [1.17, 3.85]
Kavak 2003	31.95	16.03	28	30.1	15.9	34	3.9%	1.85 [−6.14, 9.84]
Klimov 2017	22.5	9.19	37	21.7	13.93	22	5.2%	0.80 [−5.73, 7.33]
Mazure 1994	25.8	5.6	14	22.7	3.5	20	11.2%	3.10 [−0.21, 6.41]
Piatek-Guziewicz 2017, May	24.4	10.8	92	20.8	10.3	53	10.6%	3.60 [0.06, 7.14]
Piatek-Guziewicz 2017, Nov	25.2	11.2	31	19.4	9	21	6.6%	5.80 [0.29, 11.31]
Szymczak 2012	37.8	13.8	19	19.9	18.8	16	2.2%	17.90 [6.79, 29.01]
Uyanikoglu 2021	129.82	84.42	40	134.33	44.35	50	0.4%	−4.51 [−33.42, 24.40]
Volkan 2018	19.76	10.67	21	17.88	9.19	26	6.2%	1.88 [−3.89, 7.65]
**Subtotal (95% CI)**			**313**			**260**	**62.9%**	**3.09 [1.59, 4.60]**
Heterogeneity: $Tau^2 = 0.81$; $Chi^2 = 9.39$, df = 8 (P = 0.31); $I^2 = 15\%$								
Test for overall effect: Z = 4.04 (P < 0.0001)								
**Total (95% CI)**			**431**			**386**	**100.0%**	**3.02 [1.25, 4.79]**
Heterogeneity: $Tau^2 = 4.41$; $Chi^2 = 26.68$, df = 13 (P = 0.01); $I^2 = 51\%$								
Test for overall effect: Z = 3.35 (P = 0.0008)								
Test for subgroup differences: $Chi^2 = 2.41$, df = 2 (P = 0.30), $I^2 = 16.9\%$								

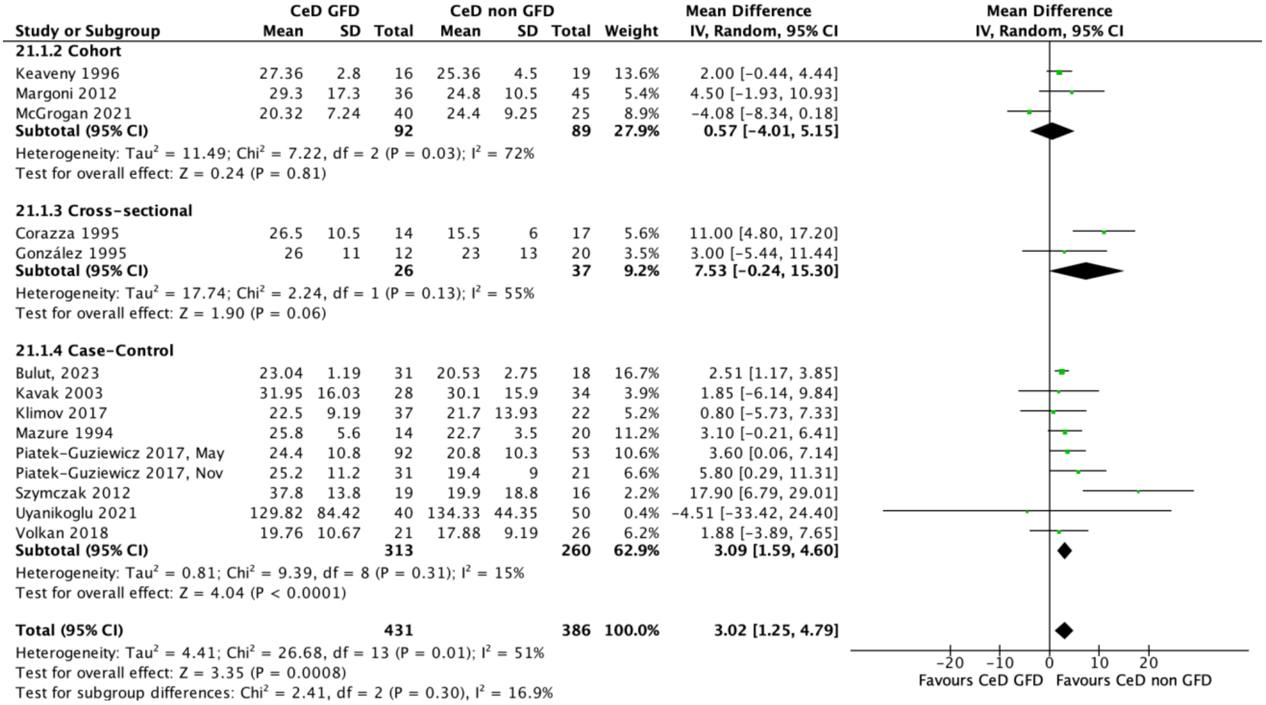

S6G- Forest plot comparison of means of serum Vitamin D levels between CeD on GFD and CeD not on GFD by GFD length

	CeD GFD			CeD non GFD				Mean Difference
Study or Subgroup	Mean	SD	Total	Mean	SD	Total	Weight	IV, Random, 95% CI
**64.1.1 Short-Term GFD**								
Bulut, 2023	23.04	1.19	31	20.53	2.75	18	16.7%	2.51 [1.17, 3.85]
Corazza 1995	26.5	10.5	14	15.5	6	17	5.6%	11.00 [4.80, 17.20]
Kavak 2003	31.95	16.03	28	30.1	15.9	34	3.9%	1.85 [-6.14, 9.84]
McGrogan 2021	20.32	7.24	40	24.4	9.25	25	8.9%	-4.08 [-8.34, 0.18]
Szymczak 2012	37.8	13.8	19	19.9	18.8	16	2.2%	17.90 [6.79, 29.01]
Volkan 2018	19.76	10.67	21	17.88	9.19	26	6.2%	1.88 [-3.89, 7.65]
**Subtotal (95% CI)**			**153**			**136**	**43.5%**	**3.87 [-0.53, 8.26]**
Heterogeneity: Tau^2 = 20.73; Chi^2 = 23.82, df = 5 (P = 0.0002); I^2 = 79%								
Test for overall effect: Z = 1.72 (P = 0.08)								
**64.1.2 Long-Term GFD**								
González 1995	26	11	12	23	13	20	3.5%	3.00 [-5.44, 11.44]
Keaveny 1996	27.36	2.8	16	25.36	4.5	19	13.6%	2.00 [-0.44, 4.44]
Margoni 2012	29.3	17.3	36	24.8	10.5	45	5.4%	4.50 [-1.93, 10.93]
Mazure 1994	25.8	5.6	14	22.7	3.5	20	11.2%	3.10 [-0.21, 6.41]
Piatek-Guziewicz 2017, May	24.4	10.8	92	20.8	10.3	53	10.6%	3.60 [0.06, 7.14]
Piatek-Guziewicz 2017, Nov	25.2	11.2	31	19.4	9	21	6.6%	5.80 [0.29, 11.31]
**Subtotal (95% CI)**			**201**			**178**	**50.9%**	**3.04 [1.48, 4.61]**
Heterogeneity: Tau^2 = 0.00; Chi^2 = 1.95, df = 5 (P = 0.86); I^2 = 0%								
Test for overall effect: Z = 3.82 (P = 0.0001)								
**64.1.3 Unknown GFD**								
Klimov 2017	22.5	9.19	37	21.7	13.93	22	5.2%	0.80 [-5.73, 7.33]
Uyanikoglu 2021	129.82	84.42	40	134.33	44.35	50	0.4%	-4.51 [-33.42, 24.40]
**Subtotal (95% CI)**			**77**			**72**	**5.6%**	**0.54 [-5.83, 6.91]**
Heterogeneity: Tau^2 = 0.00; Chi^2 = 0.12, df = 1 (P = 0.73); I^2 = 0%								
Test for overall effect: Z = 0.17 (P = 0.87)								
**Total (95% CI)**			**431**			**386**	**100.0%**	**3.02 [1.25, 4.79]**
Heterogeneity: Tau^2 = 4.41; Chi^2 = 26.68, df = 13 (P = 0.01); I^2 = 51%								
Test for overall effect: Z = 3.35 (P = 0.0008)								
Test for subgroup differences: Chi^2 = 0.72, df = 2 (P = 0.70), I^2 = 0%								

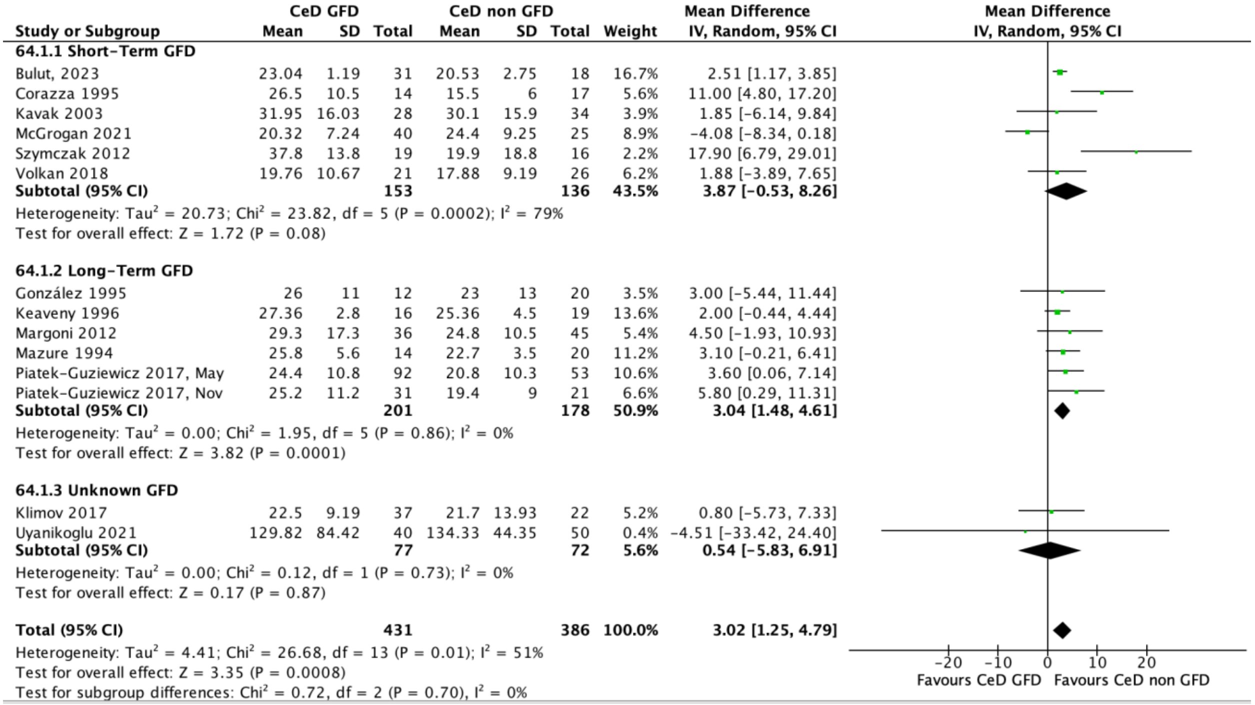

S6H- Forest plot comparison of means of serum Vitamin D levels between CeD on GFD and CeD not on GFD by population

	CeD GFD			CeD non GFD				Mean Difference
Study or Subgroup	Mean	SD	Total	Mean	SD	Total	Weight	IV, Random, 95% CI
**49.1.2 Children**								
Bulut, 2023	23.04	1.19	31	20.53	2.75	18	16.7%	2.51 [1.17, 3.85]
Kavak 2003	31.95	16.03	28	30.1	15.9	34	3.9%	1.85 [-6.14, 9.84]
Klimov 2017	22.5	9.19	37	21.7	13.93	22	5.2%	0.80 [-5.73, 7.33]
Margoni 2012	29.3	17.3	36	24.8	10.5	45	5.4%	4.50 [-1.93, 10.93]
McGrogan 2021	20.32	7.24	40	24.4	9.25	25	8.9%	-4.08 [-8.34, 0.18]
Volkan 2018	19.76	10.67	21	17.88	9.19	26	6.2%	1.88 [-3.89, 7.65]
**Subtotal (95% CI)**			**193**			**170**	**46.2%**	**1.15 [-1.40, 3.70]**
Heterogeneity: Tau^2 = 4.12; Chi^2 = 9.08, df = 5 (P = 0.11); I^2 = 45%								
Test for overall effect: Z = 0.88 (P = 0.38)								
**49.1.3 Adults**								
Corazza 1995	26.5	10.5	14	15.5	6	17	5.6%	11.00 [4.80, 17.20]
González 1995	26	11	12	23	13	20	3.5%	3.00 [-5.44, 11.44]
Keaveny 1996	27.36	2.8	16	25.36	4.5	19	13.6%	2.00 [-0.44, 4.44]
Mazure 1994	25.8	5.6	14	22.7	3.5	20	11.2%	3.10 [-0.21, 6.41]
Piatek-Guziewicz 2017, May	24.4	10.8	92	20.8	10.3	53	10.6%	3.60 [0.06, 7.14]
Piatek-Guziewicz 2017, Nov	25.2	11.2	31	19.4	9	21	6.6%	5.80 [0.29, 11.31]
Szymczak 2012	37.8	13.8	19	19.9	18.8	16	2.2%	17.90 [6.79, 29.01]
Uyanikoglu 2021	129.82	84.42	40	134.33	44.35	50	0.4%	-4.51 [-33.42, 24.40]
**Subtotal (95% CI)**			**238**			**216**	**53.8%**	**4.81 [2.16, 7.46]**
Heterogeneity: Tau^2 = 6.21; Chi^2 = 14.49, df = 7 (P = 0.04); I^2 = 52%								
Test for overall effect: Z = 3.56 (P = 0.0004)								
**Total (95% CI)**			**431**			**386**	**100.0%**	**3.02 [1.25, 4.79]**
Heterogeneity: Tau^2 = 4.41; Chi^2 = 26.68, df = 13 (P = 0.01); I^2 = 51%								
Test for overall effect: Z = 3.35 (P = 0.0008)								
Test for subgroup differences: Chi^2 = 3.82, df = 1 (P = 0.05), I^2 = 73.8%								

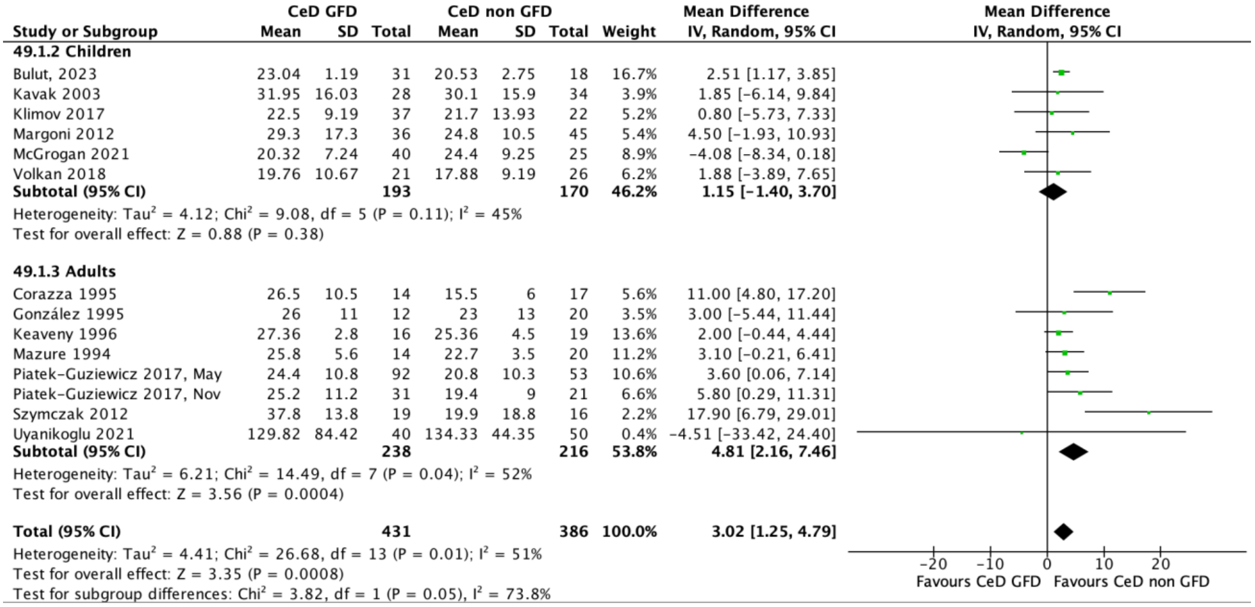

S6I- Forest plot comparison of means of serum Vitamin D levels between CeD on GFD and non-CeD controls

Study or Subgroup	CeD GFD Mean	CeD GFD SD	CeD GFD Total	Controls Mean	Controls SD	Controls Total	Weight	Mean Difference IV, Random, 95% CI
Ballestero-Fernández 2019	28.2	8.22	70	29.53	5.11	67	8.9%	−1.33 [−3.61, 0.95]
Ballestero-Fernández 2021	39.7	26.22	64	40.9	33.04	74	3.7%	−1.20 [−11.10, 8.70]
Bayrak 2020	18.6	7.7	103	18.93	9.41	35	8.1%	−0.33 [−3.78, 3.12]
Björck 2017	30.25	14.62	30	26.64	12.23	60	6.0%	3.61 [−2.47, 9.69]
Corazza 1995	26.5	10.5	14	25.25	4.75	24	6.2%	1.25 [−4.57, 7.07]
Efe 2023	15.8	0.61	50	18.3	4.5	72	9.5%	−2.50 [−3.55, −1.45]
El Amrousy 2024	21.3	4.5	40	30.1	5.1	40	9.0%	−8.80 [−10.91, −6.69]
Isikay 2018	12.39	12.8	226	14.33	3.8	268	9.2%	−1.94 [−3.67, −0.21]
Klimov 2017	22.5	9.19	37	46.2	7.93	14	6.7%	−23.70 [−28.80, −18.60]
Pazianas 2005	18.43	15.22	24	16.83	8.41	20	5.2%	1.60 [−5.52, 8.72]
Piatek-Guziewicz 2017, May	24.4	10.8	92	28.1	5.5	52	8.7%	−3.70 [−6.37, −1.03]
Piatek-Guziewicz 2017, Nov	25.2	11.2	31	29.7	5.1	25	7.3%	−4.50 [−8.92, −0.08]
Szymczak 2012	37.8	13.8	19	39.5	19	36	4.2%	−1.70 [−10.48, 7.08]
Uyanikoglu 2021	129.82	84.42	40	118.43	48	40	0.6%	11.39 [−18.70, 41.48]
Volkan 2018	19.76	10.67	41	20.63	10.58	30	6.8%	−0.87 [−5.87, 4.13]
**Total (95% CI)**			**881**			**857**	**100.0%**	**−3.33 [−5.72, −0.93]**

Heterogeneity: $Tau^2 = 15.41$; $Chi^2 = 109.41$, df = 14 (P < 0.00001); $I^2 = 87\%$
Test for overall effect: Z = 2.72 (P = 0.006)

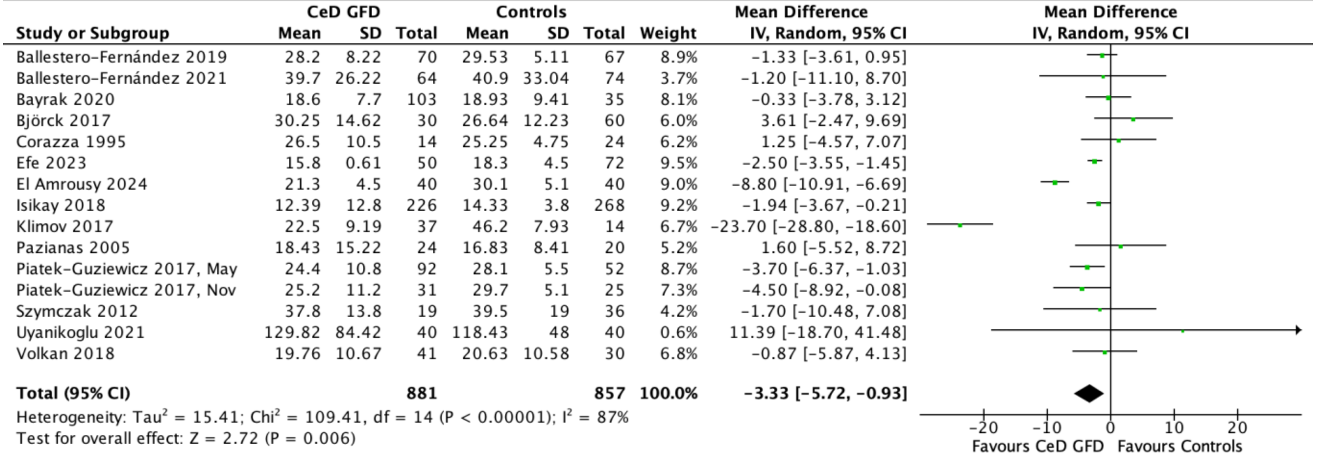

S6J- Forest plot comparison of means of serum Vitamin D levels between CeD on GFD and non-CeD controls by study design

Study or Subgroup	CeD GFD Mean	CeD GFD SD	CeD GFD Total	Controls Mean	Controls SD	Controls Total	Weight	Mean Difference IV, Random, 95% CI
**18.1.2 Cross-sectional**								
Ballestero-Fernández 2019	28.2	8.22	67	29.53	5.11	66	9.3%	−1.33 [−3.65, 0.99]
Ballestero-Fernández 2021	39.7	26.22	64	40.9	33.04	74	3.2%	−1.20 [−11.10, 8.70]
Bayrak 2020	18.6	7.7	103	18.93	9.41	35	8.2%	−0.33 [−3.78, 3.12]
Corazza 1995	26.5	10.5	14	25.25	4.75	24	5.8%	1.25 [−4.57, 7.07]
El Amrousy 2024	21.3	4.5	40	30.1	5.1	40	9.5%	−8.80 [−10.91, −6.69]
Isikay 2018	12.39	12.8	226	14.33	3.8	268	9.8%	−1.94 [−3.67, −0.21]
**Subtotal (95% CI)**			**514**			**507**	**45.7%**	**−2.45 [−5.78, 0.88]**
Heterogeneity: $Tau^2 = 13.03$; $Chi^2 = 36.66$, df = 5 (P < 0.00001); $I^2 = 86\%$								
Test for overall effect: Z = 1.44 (P = 0.15)								
**18.1.3 Case-Control**								
Björck 2017	30.25	14.62	30	26.64	12.23	57	5.6%	3.61 [−2.51, 9.73]
Efe 2023	15.8	0.61	50	18.3	0.8	72	10.4%	−2.50 [−2.75, −2.25]
Klimov 2017	22.5	9.19	37	46.2	7.93	14	6.5%	−23.70 [−28.80, −18.60]
Pazianas 2005	18.43	15.22	24	16.83	8.41	20	4.8%	1.60 [−5.52, 8.72]
Piatek-Guziewicz 2017, May	24.4	10.8	92	28.1	5.5	52	9.0%	−3.70 [−6.37, −1.03]
Piatek-Guziewicz 2017, Nov	25.2	11.2	31	29.7	5.1	25	7.2%	−4.50 [−8.92, −0.08]
Szymczak 2012	37.8	13.8	19	39.5	19	36	3.7%	−1.70 [−10.48, 7.08]
Uyanikoglu 2021	129.82	84.42	40	118.43	48	40	0.5%	11.39 [−18.70, 41.48]
Volkan 2018	19.76	10.67	41	20.63	10.58	30	6.6%	−0.87 [−5.87, 4.13]
**Subtotal (95% CI)**			**364**			**346**	**54.3%**	**−3.95 [−7.91, 0.02]**
Heterogeneity: $Tau^2 = 26.35$; $Chi^2 = 74.14$, df = 8 (P < 0.00001); $I^2 = 89\%$								
Test for overall effect: Z = 1.95 (P = 0.05)								
**Total (95% CI)**			**878**			**853**	**100.0%**	**−3.39 [−5.50, −1.27]**

Heterogeneity: $Tau^2 = 11.16$; $Chi^2 = 112.67$, df = 14 (P < 0.00001); $I^2 = 88\%$
Test for overall effect: Z = 3.13 (P = 0.002)
Test for subgroup differences: $Chi^2 = 0.32$, df = 1 (P = 0.57), $I^2 = 0\%$

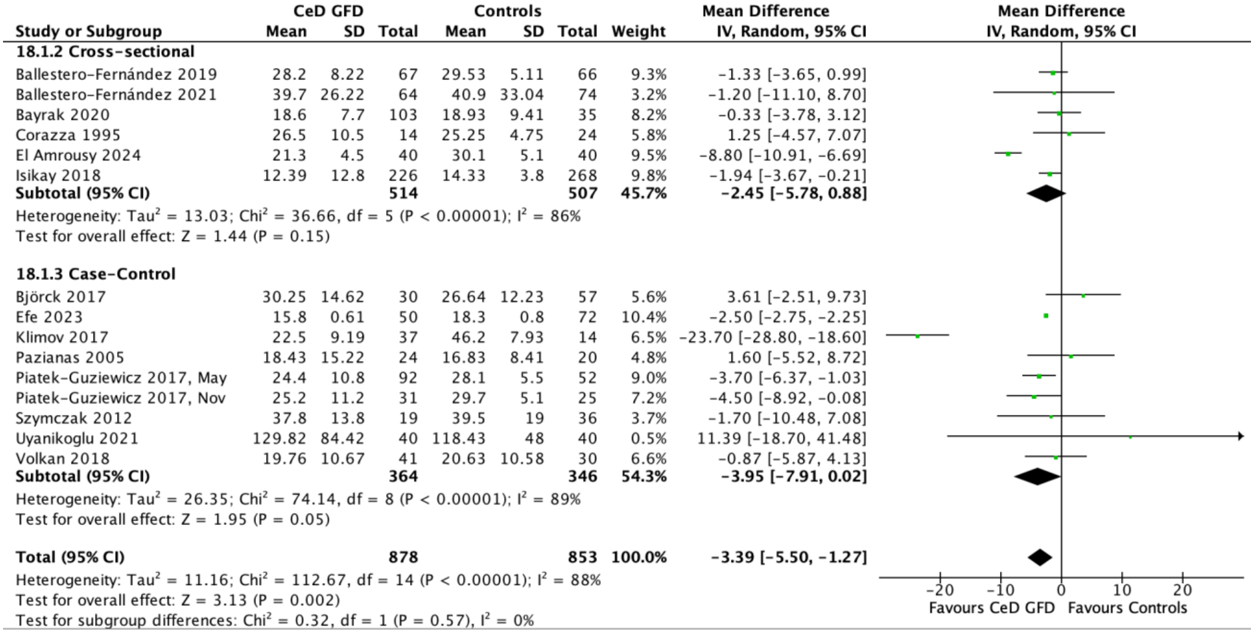

S6K- Forest plot comparison of means of serum Vitamin D levels between CeD on GFD and non-CeD controls by GFD length

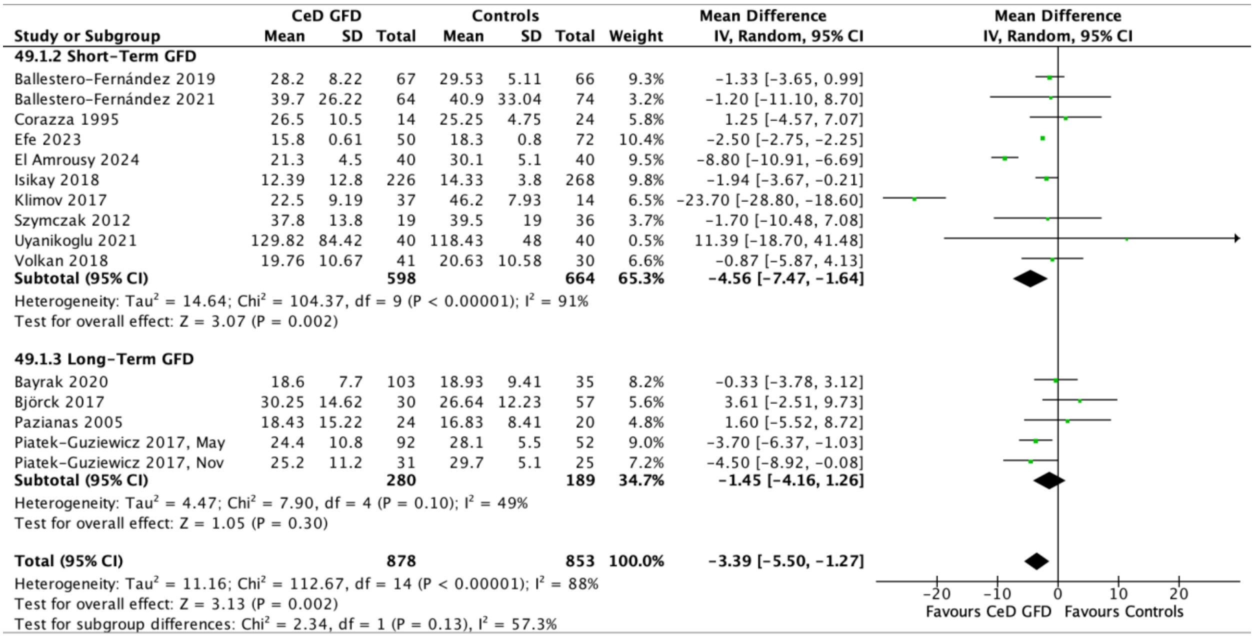

Study or Subgroup	CeD GFD Mean	CeD GFD SD	CeD GFD Total	Controls Mean	Controls SD	Controls Total	Weight	Mean Difference IV, Random, 95% CI
**49.1.2 Short-Term GFD**								
Ballestero-Fernández 2019	28.2	8.22	67	29.53	5.11	66	9.3%	−1.33 [−3.65, 0.99]
Ballestero-Fernández 2021	39.7	26.22	64	40.9	33.04	74	3.2%	−1.20 [−11.10, 8.70]
Corazza 1995	26.5	10.5	14	25.25	4.75	24	5.8%	1.25 [−4.57, 7.07]
Efe 2023	15.8	0.61	50	18.3	0.8	72	10.4%	−2.50 [−2.75, −2.25]
El Amrousy 2024	21.3	4.5	40	30.1	5.1	40	9.5%	−8.80 [−10.91, −6.69]
Isikay 2018	12.39	12.8	226	14.33	3.8	268	9.8%	−1.94 [−3.67, −0.21]
Klimov 2017	22.5	9.19	37	46.2	7.93	14	6.5%	−23.70 [−28.80, −18.60]
Szymczak 2012	37.8	13.8	19	39.5	19	36	3.7%	−1.70 [−10.48, 7.08]
Uyanikoglu 2021	129.82	84.42	40	118.43	48	40	0.5%	11.39 [−18.70, 41.48]
Volkan 2018	19.76	10.67	41	20.63	10.58	30	6.6%	−0.87 [−5.87, 4.13]
**Subtotal (95% CI)**			**598**			**664**	**65.3%**	**−4.56 [−7.47, −1.64]**
Heterogeneity: Tau² = 14.64; Chi² = 104.37, df = 9 (P < 0.00001); I² = 91%								
Test for overall effect: Z = 3.07 (P = 0.002)								
**49.1.3 Long-Term GFD**								
Bayrak 2020	18.6	7.7	103	18.93	9.41	35	8.2%	−0.33 [−3.78, 3.12]
Björck 2017	30.25	14.62	30	26.64	12.23	57	5.6%	3.61 [−2.51, 9.73]
Pazianas 2005	18.43	15.22	24	16.83	8.41	20	4.8%	1.60 [−5.52, 8.72]
Piatek-Guziewicz 2017, May	24.4	10.8	92	28.1	5.5	52	9.0%	−3.70 [−6.37, −1.03]
Piatek-Guziewicz 2017, Nov	25.2	11.2	31	29.7	5.1	25	7.2%	−4.50 [−8.92, −0.08]
**Subtotal (95% CI)**			**280**			**189**	**34.7%**	**−1.45 [−4.16, 1.26]**
Heterogeneity: Tau² = 4.47; Chi² = 7.90, df = 4 (P = 0.10); I² = 49%								
Test for overall effect: Z = 1.05 (P = 0.30)								
**Total (95% CI)**			**878**			**853**	**100.0%**	**−3.39 [−5.50, −1.27]**
Heterogeneity: Tau² = 11.16; Chi² = 112.67, df = 14 (P < 0.00001); I² = 88%								
Test for overall effect: Z = 3.13 (P = 0.002)								
Test for subgroup differences: Chi² = 2.34, df = 1 (P = 0.13), I² = 57.3%								

S6L- Forest plot comparison of means of serum Vitamin D levels between CeD on GFD and non-CeD controls by population

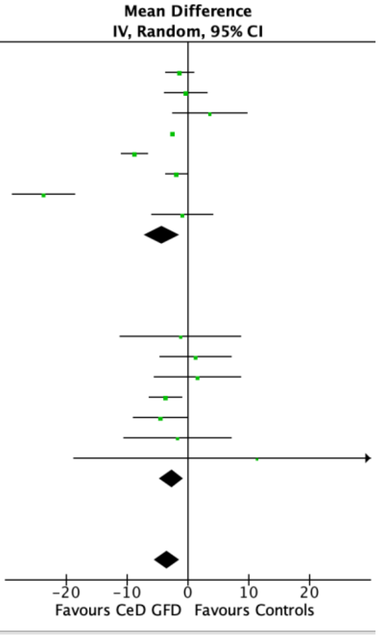

Study or Subgroup	CeD GFD Mean	CeD GFD SD	CeD GFD Total	Controls Mean	Controls SD	Controls Total	Weight	Mean Difference IV, Random, 95% CI
**36.1.2 Children**								
Ballestero-Fernández 2019	28.2	8.22	67	29.53	5.11	66	9.3%	−1.33 [−3.65, 0.99]
Bayrak 2020	18.6	7.7	103	18.93	9.41	35	8.2%	−0.33 [−3.78, 3.12]
Björck 2017	30.25	14.62	30	26.64	12.23	57	5.6%	3.61 [−2.51, 9.73]
Efe 2023	15.8	0.61	50	18.3	0.8	72	10.4%	−2.50 [−2.75, −2.25]
El Amrousy 2024	21.3	4.5	40	30.1	5.1	40	9.5%	−8.80 [−10.91, −6.69]
Isikay 2018	12.39	12.8	226	14.33	3.8	268	9.8%	−1.94 [−3.67, −0.21]
Klimov 2017	22.5	9.19	37	46.2	7.93	14	6.5%	−23.70 [−28.80, −18.60]
Volkan 2018	19.76	10.67	41	20.63	10.58	30	6.6%	−0.87 [−5.87, 4.13]
**Subtotal (95% CI)**			**594**			**582**	**65.8%**	**−4.34 [−7.25, −1.43]**
Heterogeneity: Tau² = 14.48; Chi² = 107.34, df = 7 (P < 0.00001); I² = 93%								
Test for overall effect: Z = 2.93 (P = 0.003)								
**36.1.3 Adults**								
Ballestero-Fernández 2021	39.7	26.22	64	40.9	33.04	74	3.2%	−1.20 [−11.10, 8.70]
Corazza 1995	26.5	10.5	14	25.25	4.75	24	5.8%	1.25 [−4.57, 7.07]
Pazianas 2005	18.43	15.22	24	16.83	8.41	20	4.8%	1.60 [−5.52, 8.72]
Piatek-Guziewicz 2017, May	24.4	10.8	92	28.1	5.5	52	9.0%	−3.70 [−6.37, −1.03]
Piatek-Guziewicz 2017, Nov	25.2	11.2	31	29.7	5.1	25	7.2%	−4.50 [−8.92, −0.08]
Szymczak 2012	37.8	13.8	19	39.5	19	36	3.7%	−1.70 [−10.48, 7.08]
Uyanikoglu 2021	129.82	84.42	40	118.43	48	40	0.5%	11.39 [−18.70, 41.48]
**Subtotal (95% CI)**			**284**			**271**	**34.2%**	**−2.65 [−4.59, −0.71]**
Heterogeneity: Tau² = 0.00; Chi² = 5.33, df = 6 (P = 0.50); I² = 0%								
Test for overall effect: Z = 2.68 (P = 0.007)								
**Total (95% CI)**			**878**			**853**	**100.0%**	**−3.39 [−5.50, −1.27]**
Heterogeneity: Tau² = 11.16; Chi² = 112.67, df = 14 (P < 0.00001); I² = 88%								
Test for overall effect: Z = 3.13 (P = 0.002)								
Test for subgroup differences: Chi² = 0.90, df = 1 (P = 0.34), I² = 0%								

Supplementary Figure S7. 1,25-OH Vitamin D

S7A- Forest plot comparison of Vitamin 1,25 OH D deficiency between CeD on GFD and CeD not on GFD

Study or Subgroup	CeD GFD Events	CeD GFD Total	CeD non GFD Events	CeD non GFD Total	Weight	Odds Ratio M-H, Fixed, 95% CI
Ciacci 2020	0	55	1	50	100.0%	0.30 [0.01, 7.47]
**Total (95% CI)**		**55**		**50**	**100.0%**	**0.30 [0.01, 7.47]**
Total events	0		1			

Heterogeneity: Not applicable
Test for overall effect: Z = 0.74 (P = 0.46)

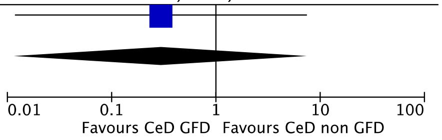

S7B- Forest plot comparison of means of serum 1,25-OH Vitamin D levels between CeD on GFD to CeD not on GFD

Study or Subgroup	CeD GFD Mean	CeD GFD SD	CeD GFD Total	CeD non GFD Mean	CeD non GFD SD	CeD non GFD Total	Weight	Mean Difference IV, Random, 95% CI
Corazza 1995	47	9.5	14	54.25	10.25	17	25.1%	−7.25 [−14.21, −0.29]
Keaveny 1996	40.05	3.25	16	59.55	7.34	19	28.1%	−19.50 [−23.16, −15.84]
Szymczak 2012	35.8	12.7	19	45.2	17.5	16	21.3%	−9.40 [−19.70, 0.90]
Uyanikoglu 2021	18.93	12.4	40	21.95	17.09	40	25.5%	−3.02 [−9.56, 3.52]
**Total (95% CI)**			**89**			**92**	**100.0%**	**−10.07 [−19.06, −1.09]**

Heterogeneity: $Tau^2 = 71.12$; $Chi^2 = 23.82$, df = 3 (P < 0.0001); $I^2 = 87\%$
Test for overall effect: Z = 2.20 (P = 0.03)

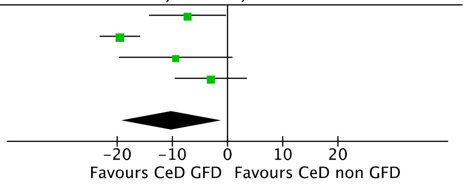

S7C- Forest plot comparison of means of serum 1,25-OH Vitamin D levels between CeD on GFD to CeD not on GFD sensitivity analysis removing Keaveny et al 1996.

Study or Subgroup	CeD GFD Mean	CeD GFD SD	CeD GFD Total	CeD non GFD Mean	CeD non GFD SD	CeD non GFD Total	Weight	Mean Difference IV, Random, 95% CI
Corazza 1995	47	9.5	14	54.25	10.25	17	38.6%	−7.25 [−14.21, −0.29]
Szymczak 2012	35.8	12.7	19	45.2	17.5	16	17.6%	−9.40 [−19.70, 0.90]
Uyanikoglu 2021	18.93	12.4	40	21.95	17.09	40	43.7%	−3.02 [−9.56, 3.52]
**Total (95% CI)**			**73**			**73**	**100.0%**	**−5.78 [−10.11, −1.45]**

Heterogeneity: $Tau^2 = 0.00$; $Chi^2 = 1.33$, df = 2 (P = 0.51); $I^2 = 0\%$
Test for overall effect: Z = 2.62 (P = 0.009)

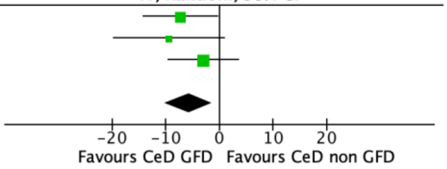

S7D- Forest plot comparison of means of serum 1,25-OH Vitamin D levels in patients with CeD on GFD to non-CeD controls

Study or Subgroup	CeD GFD Mean	CeD GFD SD	CeD GFD Total	Controls Mean	Controls SD	Controls Total	Weight	Mean Difference IV, Random, 95% CI
Corazza 1995	47	9.5	14	26.5	5.5	24	33.9%	20.50 [15.06, 25.94]
Szymczak 2012	35.8	12.7	19	36.6	14.5	36	33.2%	−0.80 [−8.22, 6.62]
Uyanikoglu 2021	18.93	12.4	40	31.31	22.9	40	32.9%	−12.38 [−20.45, −4.31]
**Total (95% CI)**			**73**			**100**	**100.0%**	**2.61 [−17.35, 22.57]**

Heterogeneity: $Tau^2 = 298.13$; $Chi^2 = 50.03$, df = 2 (P < 0.00001); $I^2 = 96\%$
Test for overall effect: Z = 0.26 (P = 0.80)

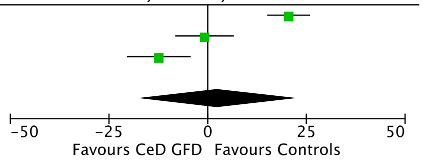

S7E- Forest plot comparison of means of serum 1,25-OH Vitamin D levels in patients with CeD on GFD to non-CeD controls by study design. All studies assessed short term GFD in adults

Study or Subgroup	CeD GFD Mean	CeD GFD SD	CeD GFD Total	Controls Mean	Controls SD	Controls Total	Weight	Mean Difference IV, Random, 95% CI
**4.1.1 Cross-sectional**								
Corazza 1995	47	9.5	14	26.5	5.5	24	33.9%	20.50 [15.06, 25.94]
**Subtotal (95% CI)**			**14**			**24**	**33.9%**	**20.50 [15.06, 25.94]**
Heterogeneity: Not applicable								
Test for overall effect: Z = 7.38 (P < 0.00001)								
**4.1.2 Case-Control**								
Szymczak 2012	35.8	12.7	19	36.6	14.5	36	33.2%	−0.80 [−8.22, 6.62]
Uyanikoglu 2021	18.93	12.4	40	31.31	22.9	40	32.9%	−12.38 [−20.45, −4.31]
**Subtotal (95% CI)**			**59**			**76**	**66.1%**	**−6.48 [−17.82, 4.87]**
Heterogeneity: $Tau^2 = 51.41$; $Chi^2 = 4.29$, df = 1 (P = 0.04); $I^2 = 77\%$								
Test for overall effect: Z = 1.12 (P = 0.26)								
**Total (95% CI)**			**73**			**100**	**100.0%**	**2.61 [−17.35, 22.57]**
Heterogeneity: $Tau^2 = 298.13$; $Chi^2 = 50.03$, df = 2 (P < 0.00001); $I^2 = 96\%$								
Test for overall effect: Z = 0.26 (P = 0.80)								
Test for subgroup differences: $Chi^2 = 17.66$, df = 1 (P < 0.0001), $I^2 = 94.3\%$								

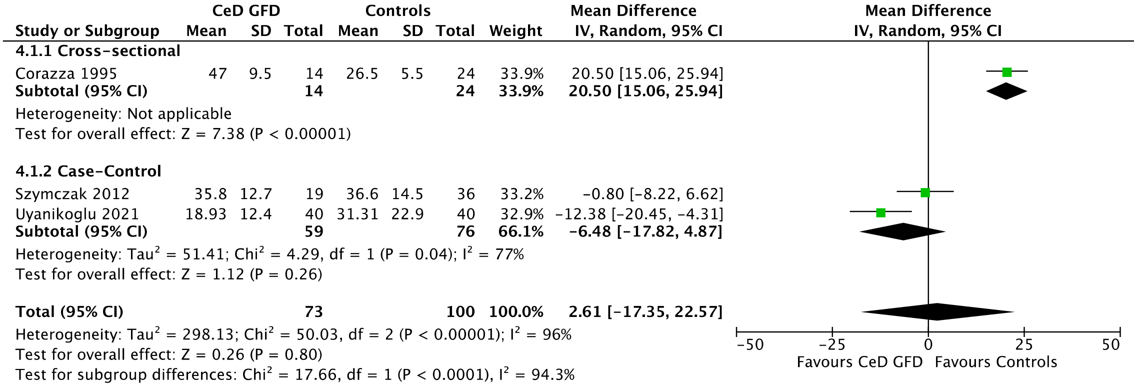

Supplementary Figure S8. Vitamin E

S8A- Forest plot comparison of serum Vitamin E deficiency between CeD on GFD and CeD not on GFD

Study or Subgroup	CeD GFD Events	CeD GFD Total	CeD non GFD Events	CeD non GFD Total	Weight	Odds Ratio M-H, Random, 95% CI
Hozyasz 2003	2	12	18	18	24.7%	0.01 [0.00, 0.15]
McGrogan 2021	0	36	18	25	25.7%	0.01 [0.00, 0.10]
Romańczuk 2016	29	48	7	7	25.7%	0.10 [0.01, 1.87]
Selbuz 2021	1	14	0	14	23.9%	3.22 [0.12, 86.09]
**Total (95% CI)**		**110**		**64**	**100.0%**	**0.06 [0.00, 0.94]**
Total events	32		43			
Heterogeneity: $Tau^2 = 5.87$; $Chi^2 = 10.24$, df = 3 (P = 0.02); $I^2 = 71\%$						
Test for overall effect: Z = 2.00 (P = 0.05)						

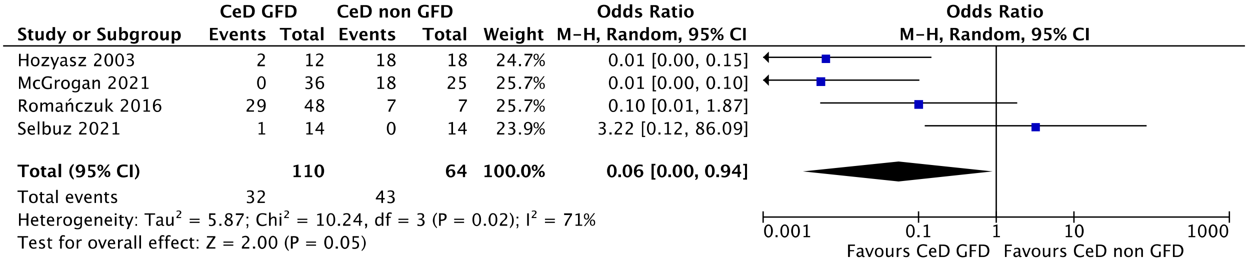

S8B- Forest plot comparison of Vitamin E in erythrocyte deficiency between CeD on GFD and CeD not on GFD by study design and GFD length

Study or Subgroup	CeD GFD Events	CeD GFD Total	CeD non GFD Events	CeD non GFD Total	Weight	Odds Ratio M-H, Random, 95% CI
**47.1.1 Cohort/Long-Term GFD**						
Hozyasz 2003	2	12	18	18	47.9%	0.01 [0.00, 0.15]
**Subtotal (95% CI)**		**12**		**18**	**47.9%**	**0.01 [0.00, 0.15]**
Total events	2		18			
Heterogeneity: Not applicable						
Test for overall effect: Z = 3.16 (P = 0.002)						
**47.1.2 Case-Control/Unknown GFD**						
Romańczuk 2016	29	48	7	7	52.1%	0.10 [0.01, 1.87]
**Subtotal (95% CI)**		**48**		**7**	**52.1%**	**0.10 [0.01, 1.87]**
Total events	29		7			
Heterogeneity: Not applicable						
Test for overall effect: Z = 1.54 (P = 0.12)						
**Total (95% CI)**		**60**		**25**	**100.0%**	**0.03 [0.00, 0.41]**
Total events	31		25			
Heterogeneity: $Tau^2 = 1.49$; $Chi^2 = 1.62$, df = 1 (P = 0.20); $I^2 = 38\%$						
Test for overall effect: Z = 2.60 (P = 0.009)						
Test for subgroup differences: $Chi^2 = 1.59$, df = 1 (P = 0.21), $I^2 = 37.0\%$						

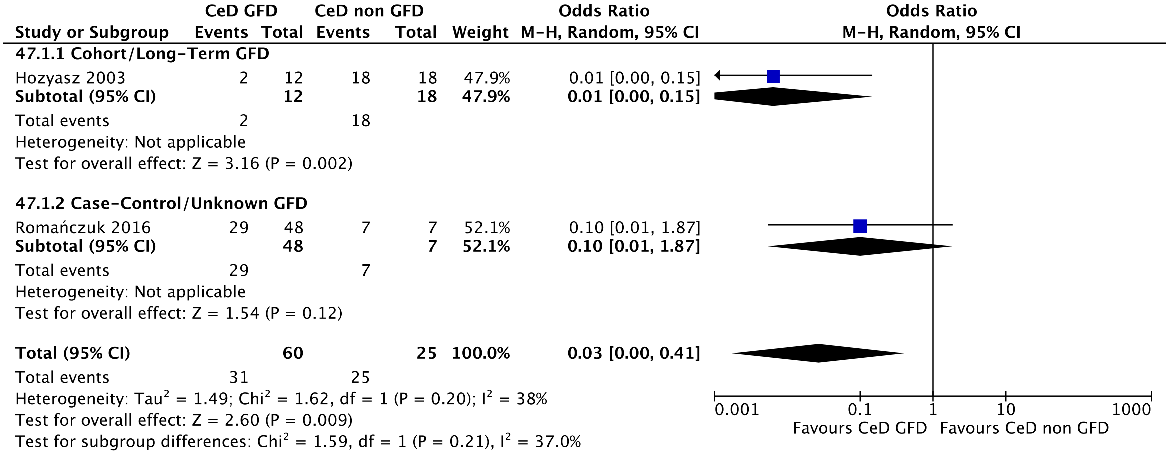

S8C- Forest plot comparison of means of serum Vitamin E levels between CeD on GFD to CeD not on GFD by methodology

Study or Subgroup	CeD GFD Mean	SD	Total	CeD non GFD Mean	SD	Total	Weight	Mean Difference IV, Random, 95% CI
**51.1.1 Chromatography**								
Hozyasz 2003	19.13	7.83	12	13.69	3.76	18	88.9%	5.44 [0.68, 10.20]
**Subtotal (95% CI)**			**12**			**18**	**88.9%**	**5.44 [0.68, 10.20]**
Heterogeneity: Not applicable								
Test for overall effect: Z = 2.24 (P = 0.03)								
**51.1.2 Spectrophotometry**								
Piatek-Guziewicz 2017, May	37.3	32.1	22	41.1	36.8	53	11.1%	−3.80 [−20.48, 12.88]
**Subtotal (95% CI)**			**22**			**53**	**11.1%**	**−3.80 [−20.48, 12.88]**
Heterogeneity: Not applicable								
Test for overall effect: Z = 0.45 (P = 0.66)								
**Total (95% CI)**			**34**			**71**	**100.0%**	**4.42 [−1.26, 10.10]**
Heterogeneity: Tau² = 3.55; Chi² = 1.09, df = 1 (P = 0.30); I² = 8%								
Test for overall effect: Z = 1.52 (P = 0.13)								
Test for subgroup differences: Chi² = 1.09, df = 1 (P = 0.30), I² = 8.3%								

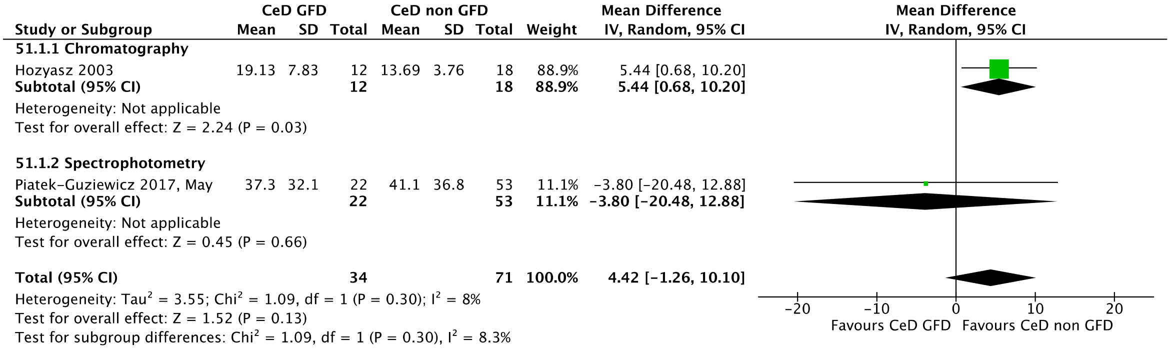

S8D- Forest plot comparison of means of serum Vitamin E levels controlled by serum cholesterol between CeD on GFD and CeD not on GFD

Study or Subgroup	CeD GFD Mean	SD	Total	CeD non GFD Mean	SD	Total	Weight	Mean Difference IV, Random, 95% CI
Hozyasz 2003	4.24	0.85	12	3.36	0.9	18	49.8%	0.88 [0.24, 1.52]
McGrogan 2021	5.76	0.93	36	1.98	1.02	25	50.2%	3.78 [3.28, 4.28]
**Total (95% CI)**			**48**			**43**	**100.0%**	**2.34 [−0.51, 5.18]**
Heterogeneity: Tau² = 4.12; Chi² = 49.22, df = 1 (P < 0.00001); I² = 98%								
Test for overall effect: Z = 1.61 (P = 0.11)								

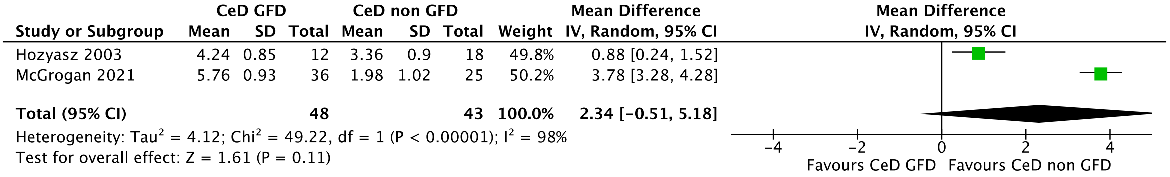

S8E- Forest plot comparison of means of serum Vitamin E levels controlled by serum cholesterol between CeD on GFD and CeD not on GFD by GFD length

Study or Subgroup	CeD GFD Mean	SD	Total	CeD non GFD Mean	SD	Total	Weight	Mean Difference IV, Random, 95% CI
**68.1.1 Short-Term GFD**								
McGrogan 2021	5.76	0.93	36	1.98	1.02	25	50.2%	3.78 [3.28, 4.28]
**Subtotal (95% CI)**			**36**			**25**	**50.2%**	**3.78 [3.28, 4.28]**
Heterogeneity: Not applicable								
Test for overall effect: Z = 14.75 (P < 0.00001)								
**68.1.2 Long-Term GFD**								
Hozyasz 2003	4.24	0.85	12	3.36	0.9	18	49.8%	0.88 [0.24, 1.52]
**Subtotal (95% CI)**			**12**			**18**	**49.8%**	**0.88 [0.24, 1.52]**
Heterogeneity: Not applicable								
Test for overall effect: Z = 2.71 (P = 0.007)								
**Total (95% CI)**			**48**			**43**	**100.0%**	**2.34 [−0.51, 5.18]**
Heterogeneity: Tau² = 4.12; Chi² = 49.22, df = 1 (P < 0.00001); I² = 98%								
Test for overall effect: Z = 1.61 (P = 0.11)								
Test for subgroup differences: Chi² = 49.22, df = 1 (P < 0.00001), I² = 98.0%								

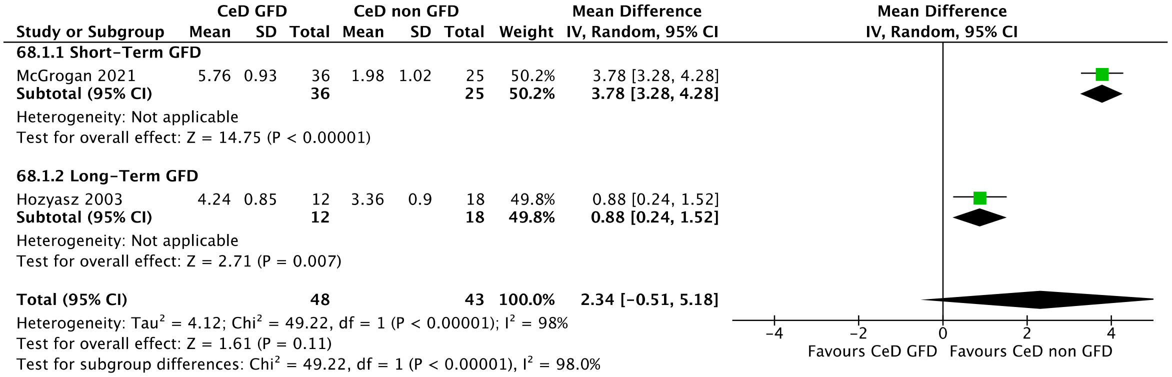

S8F- Forest plot comparison of means of Vitamin E in erythrocytes between CeD on GFD and CeD not on GFD

Study or Subgroup	CeD GFD Mean	SD	Total	CeD non GFD Mean	SD	Total	Weight	Mean Difference IV, Random, 95% CI
Hozyasz 2003	2.89	0.52	12	1.7	0.45	18	49.3%	1.19 [0.83, 1.55]
Romańczuk 2016	2.13	0.83	48	1.76	0.26	7	50.7%	0.37 [0.07, 0.67]
**Total (95% CI)**			**60**			**25**	**100.0%**	**0.77 [−0.03, 1.58]**
Heterogeneity: Tau² = 0.31; Chi² = 11.63, df = 1 (P = 0.0006); I² = 91%								
Test for overall effect: Z = 1.89 (P = 0.06)								

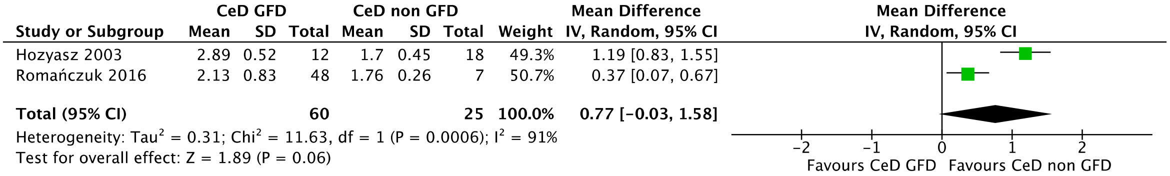

S8G- Forest plot comparison of means of Vitamin E in erythrocytes between CeD on GFD and CeD not on GFD by GFD length

Study or Subgroup	CeD GFD Mean	CeD GFD SD	CeD GFD Total	CeD non GFD Mean	CeD non GFD SD	CeD non GFD Total	Weight	Mean Difference IV, Random, 95% CI
**68.1.1 Long-Term GFD**								
Hozyasz 2003	2.89	0.52	12	1.7	0.45	18	49.3%	1.19 [0.83, 1.55]
**Subtotal (95% CI)**			**12**			**18**	**49.3%**	**1.19 [0.83, 1.55]**
Heterogeneity: Not applicable								
Test for overall effect: Z = 6.47 (P < 0.00001)								
**68.1.2 Unknown GFD**								
Romańczuk 2016	2.13	0.83	48	1.76	0.26	7	50.7%	0.37 [0.07, 0.67]
**Subtotal (95% CI)**			**48**			**7**	**50.7%**	**0.37 [0.07, 0.67]**
Heterogeneity: Not applicable								
Test for overall effect: Z = 2.39 (P = 0.02)								
**Total (95% CI)**			**60**			**25**	**100.0%**	**0.77 [−0.03, 1.58]**
Heterogeneity: $Tau^2 = 0.31$; $Chi^2 = 11.63$, df = 1 (P = 0.0006); $I^2 = 91\%$								
Test for overall effect: Z = 1.89 (P = 0.06)								
Test for subgroup differences: $Chi^2 = 11.63$, df = 1 (P = 0.0006), $I^2 = 91.4\%$								

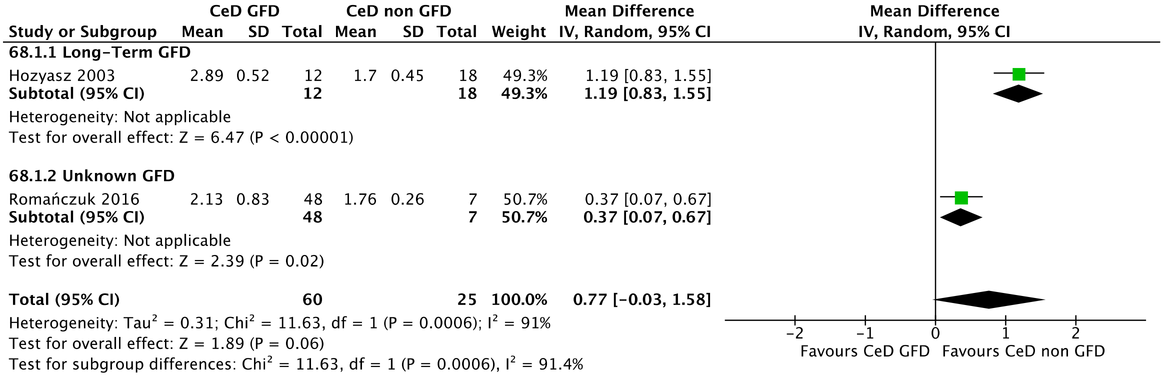

S8H- Forest plot comparison of means of Vitamin E in erythrocytes and plasma between CeD on GFD and non-CeD controls by methodology

Study or Subgroup	CeD GFD Mean	CeD GFD SD	CeD GFD Total	Controls Mean	Controls SD	Controls Total	Weight	Mean Difference IV, Random, 95% CI
**13.1.1 Erythrocytes level by Chromo**								
Romańczuk 2016	2.13	0.83	48	3.15	0.33	20	60.2%	−1.02 [−1.30, −0.74]
**Subtotal (95% CI)**			**48**			**20**	**60.2%**	**−1.02 [−1.30, −0.74]**
Heterogeneity: Not applicable								
Test for overall effect: Z = 7.25 (P < 0.00001)								
**13.1.2 Spectrophotography**								
Piatek-Guziewicz 2017, May	37.3	32.1	92	48.1	20.8	52	39.8%	−10.80 [−19.46, −2.14]
**Subtotal (95% CI)**			**92**			**52**	**39.8%**	**−10.80 [−19.46, −2.14]**
Heterogeneity: Not applicable								
Test for overall effect: Z = 2.44 (P = 0.01)								
**Total (95% CI)**			**140**			**72**	**100.0%**	**−4.91 [−14.30, 4.47]**
Heterogeneity: $Tau^2 = 38.05$; $Chi^2 = 4.90$, df = 1 (P = 0.03); $I^2 = 80\%$								
Test for overall effect: Z = 1.03 (P = 0.30)								
Test for subgroup differences: $Chi^2 = 4.90$, df = 1 (P = 0.03), $I^2 = 79.6\%$								

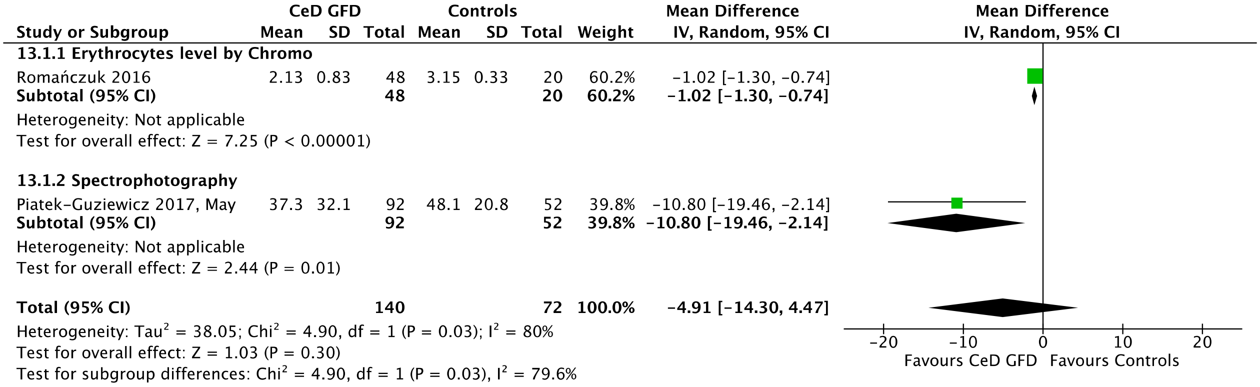

<u>Supplementary Figure S9. Vitamin K</u>

S9A- Forest plot comparison of Vitamin K levels between CeD on GFD and CeD not on GFD by methodology and study design

Study or Subgroup	CeD GFD Mean	CeD GFD SD	CeD GFD Total	CeD non GFD Mean	CeD non GFD SD	CeD non GFD Total	Weight	Mean Difference IV, Random, 95% CI
**17.1.1 Plasma/Triglyceride level (Cohort)**								
McGrogan 2021	0.78	0.48	38	0.25	0.13	24	98.1%	0.53 [0.37, 0.69]
**Subtotal (95% CI)**			**38**			**24**	**98.1%**	**0.53 [0.37, 0.69]**
Heterogeneity: Not applicable								
Test for overall effect: Z = 6.44 (P < 0.00001)								
**17.1.2 Plasma (Case-Control)**								
Volkan 2018	2.67	2.31	21	2.21	1.57	26	1.9%	0.46 [−0.70, 1.62]
**Subtotal (95% CI)**			**21**			**26**	**1.9%**	**0.46 [−0.70, 1.62]**
Heterogeneity: Not applicable								
Test for overall effect: Z = 0.78 (P = 0.44)								
**Total (95% CI)**			**59**			**50**	**100.0%**	**0.53 [0.37, 0.69]**
Heterogeneity: $Tau^2 = 0.00$; $Chi^2 = 0.01$, df = 1 (P = 0.91); $I^2 = 0\%$								
Test for overall effect: Z = 6.49 (P < 0.00001)								
Test for subgroup differences: $Chi^2 = 0.01$, df = 1 (P = 0.91), $I^2 = 0\%$								

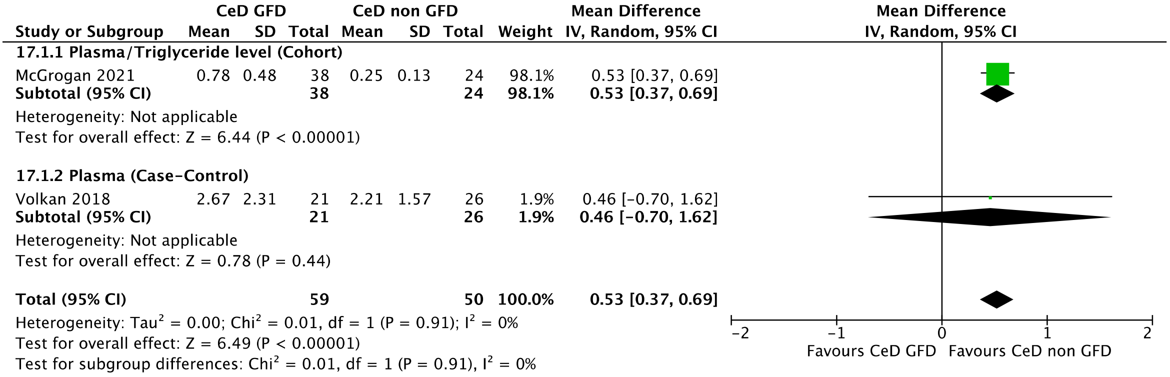

S9B- Forest plot comparison of means of serum Vitamin K levels between CeD on GFD and non-CeD controls

	CeD GFD			Controls				Mean Difference
Study or Subgroup	Mean	SD	Total	Mean	SD	Total	Weight	IV, Random, 95% CI
Volkan 2018	2.67	2.31	21	3.05	2.3	30	100.0%	−0.38 [−1.67, 0.91]
**Total (95% CI)**			**21**			**30**	**100.0%**	**−0.38 [−1.67, 0.91]**

Heterogeneity: Not applicable
Test for overall effect: Z = 0.58 (P = 0.56)

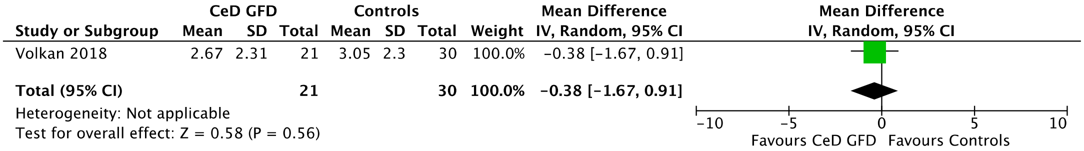

Supplementary Figure S10. Calcium

S10A- Forest plot comparison of Calcium deficiency between CeD on GFD and CeD not on GFD

	CeD GFD		CeD non GFD			Odds Ratio
Study or Subgroup	Events	Total	Events	Total	Weight	M-H, Fixed, 95% CI
Choudhary 2017	6	36	21	36	51.8%	0.14 [0.05, 0.43]
Ciacci 2020	3	55	5	50	14.7%	0.52 [0.12, 2.29]
González 1995	4	12	3	20	4.4%	2.83 [0.51, 15.77]
Kavak 2003	1	28	6	34	15.5%	0.17 [0.02, 1.53]
Margoni 2012	0	36	1	45	3.9%	0.41 [0.02, 10.28]
Mazure 1994	0	10	4	17	9.7%	0.14 [0.01, 2.96]
Selbuz 2021	0	20	0	32		Not estimable
**Total (95% CI)**		**197**		**234**	**100.0%**	**0.33 [0.17, 0.64]**
Total events	14		40			

Heterogeneity: $Chi^2 = 9.26$, df = 5 (P = 0.10); $I^2 = 46\%$
Test for overall effect: Z = 3.29 (P = 0.001)

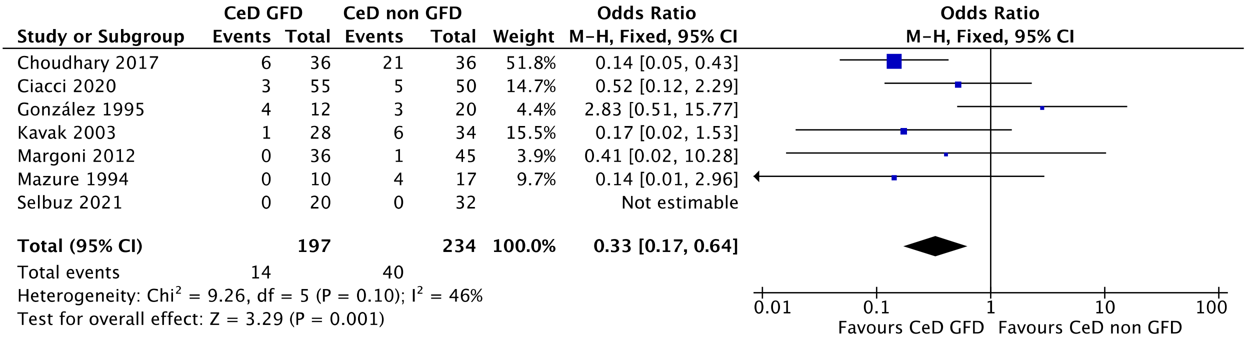

S10B- Forest plot comparison of Calcium deficiency between CeD on GFD and CeD not on GFD by study design

	CeD GFD		CeD non GFD			Odds Ratio
Study or Subgroup	Events	Total	Events	Total	Weight	M-H, Fixed, 95% CI
**6.1.1 Cohort**						
Margoni 2012	0	36	1	45	3.9%	0.41 [0.02, 10.28]
**Subtotal (95% CI)**		**36**		**45**	**3.9%**	**0.41 [0.02, 10.28]**
Total events	0		1			
Heterogeneity: Not applicable						
Test for overall effect: Z = 0.55 (P = 0.58)						
**6.1.2 Cross-sectional**						
Choudhary 2017	6	36	21	36	51.8%	0.14 [0.05, 0.43]
González 1995	4	12	3	20	4.4%	2.83 [0.51, 15.77]
**Subtotal (95% CI)**		**48**		**56**	**56.3%**	**0.36 [0.15, 0.83]**
Total events	10		24			
Heterogeneity: $Chi^2 = 8.26$, df = 1 (P = 0.004); $I^2 = 88\%$						
Test for overall effect: Z = 2.40 (P = 0.02)						
**6.1.3 Case-Control**						
Ciacci 2020	3	55	5	50	14.7%	0.52 [0.12, 2.29]
Kavak 2003	1	28	6	34	15.5%	0.17 [0.02, 1.53]
Mazure 1994	0	10	4	17	9.7%	0.14 [0.01, 2.96]
Selbuz 2021	0	20	0	32		Not estimable
**Subtotal (95% CI)**		**113**		**133**	**39.8%**	**0.29 [0.10, 0.88]**
Total events	4		15			
Heterogeneity: $Chi^2 = 1.01$, df = 2 (P = 0.60); $I^2 = 0\%$						
Test for overall effect: Z = 2.18 (P = 0.03)						
**Total (95% CI)**		**197**		**234**	**100.0%**	**0.33 [0.17, 0.64]**
Total events	14		40			

Heterogeneity: $Chi^2 = 9.26$, df = 5 (P = 0.10); $I^2 = 46\%$
Test for overall effect: Z = 3.29 (P = 0.001)
Test for subgroup differences: $Chi^2 = 0.09$, df = 2 (P = 0.96), $I^2 = 0\%$

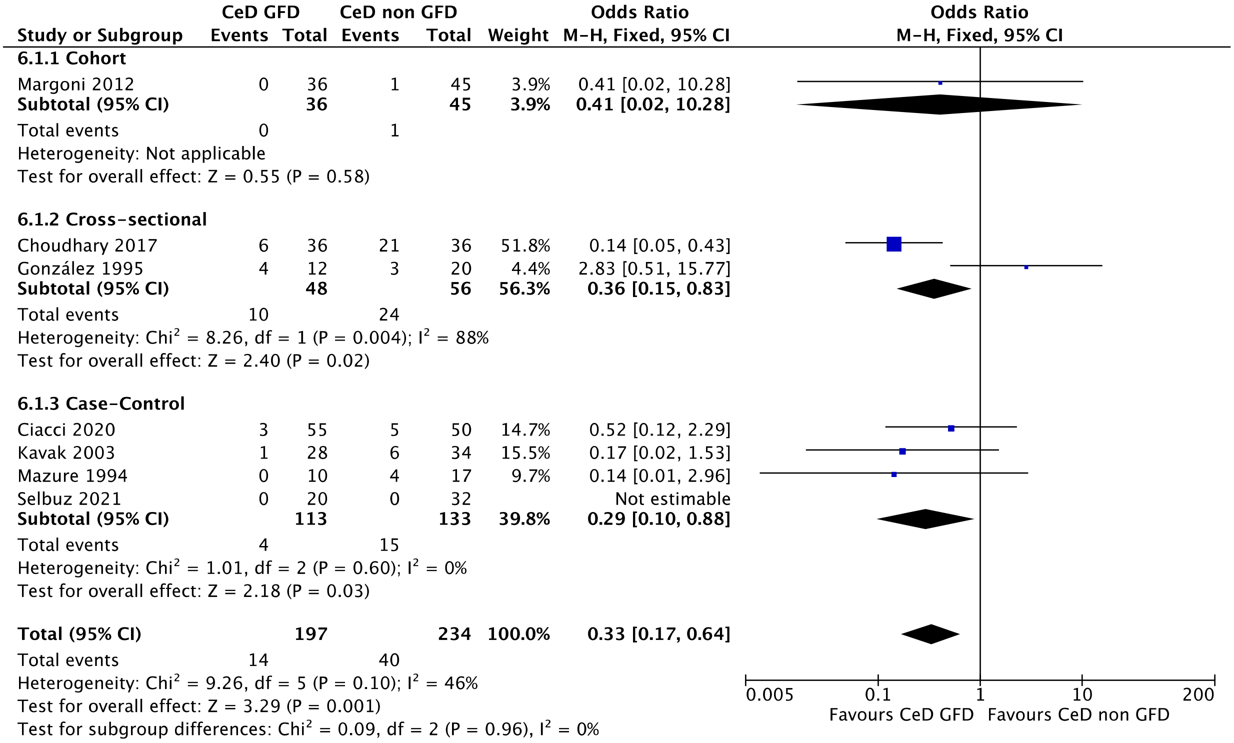

S10C- Forest plot comparison of Calcium deficiency between CeD on GFD and CeD not on GFD by GFD length

Study or Subgroup	CeD GFD Events	CeD GFD Total	CeD non GFD Events	CeD non GFD Total	Weight	Odds Ratio M-H, Fixed, 95% CI
**33.1.1 Short-Term GFD**						
Choudhary 2017	6	36	21	36	51.8%	0.14 [0.05, 0.43]
Kavak 2003	1	28	6	34	15.5%	0.17 [0.02, 1.53]
Selbuz 2021	0	20	0	32		Not estimable
**Subtotal (95% CI)**		**84**		**102**	**67.3%**	**0.15 [0.06, 0.40]**
Total events	7		27			
Heterogeneity: $Chi^2 = 0.02, df = 1 (P = 0.88); I^2 = 0\%$						
Test for overall effect: Z = 3.76 (P = 0.0002)						
**33.1.2 Long-Term GFD**						
Ciacci 2020	3	55	5	50	14.7%	0.52 [0.12, 2.29]
González 1995	4	12	3	20	4.4%	2.83 [0.51, 15.77]
Margoni 2012	0	36	1	45	3.9%	0.41 [0.02, 10.28]
Mazure 1994	0	10	4	17	9.7%	0.14 [0.01, 2.96]
**Subtotal (95% CI)**		**113**		**132**	**32.7%**	**0.71 [0.28, 1.78]**
Total events	7		13			
Heterogeneity: $Chi^2 = 3.86, df = 3 (P = 0.28); I^2 = 22\%$						
Test for overall effect: Z = 0.73 (P = 0.47)						
**Total (95% CI)**		**197**		**234**	**100.0%**	**0.33 [0.17, 0.64]**
Total events	14		40			
Heterogeneity: $Chi^2 = 9.26, df = 5 (P = 0.10); I^2 = 46\%$						
Test for overall effect: Z = 3.29 (P = 0.001)						
Test for subgroup differences: $Chi^2 = 5.07, df = 1 (P = 0.02), I^2 = 80.3\%$						

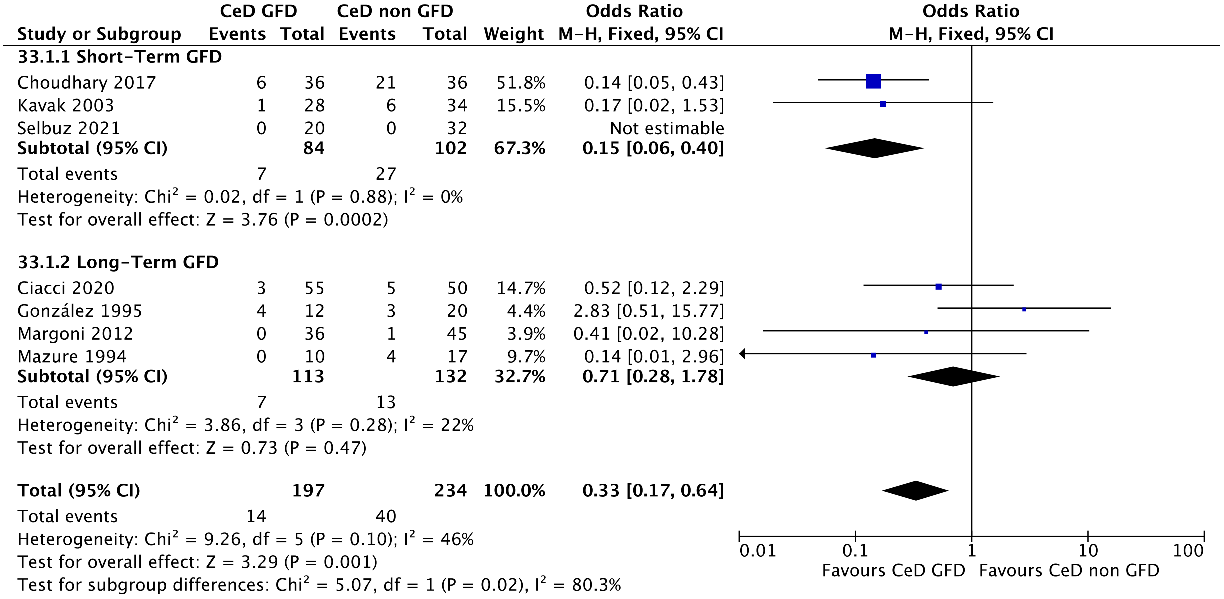

S10D- Forest plot comparison of Calcium deficiency between CeD on GFD and CeD not on GFD by population

Study or Subgroup	CeD GFD Events	CeD GFD Total	CeD non GFD Events	CeD non GFD Total	Weight	Odds Ratio M-H, Fixed, 95% CI
**34.1.1 Children**						
Choudhary 2017	6	36	21	36	51.8%	0.14 [0.05, 0.43]
Kavak 2003	1	28	6	34	15.5%	0.17 [0.02, 1.53]
Margoni 2012	0	36	1	45	3.9%	0.41 [0.02, 10.28]
Selbuz 2021	0	20	0	32		Not estimable
**Subtotal (95% CI)**		**120**		**147**	**71.2%**	**0.16 [0.06, 0.42]**
Total events	7		28			
Heterogeneity: $Chi^2 = 0.37, df = 2 (P = 0.83); I^2 = 0\%$						
Test for overall effect: Z = 3.74 (P = 0.0002)						
**34.1.2 Adults**						
Ciacci 2020	3	55	5	50	14.7%	0.52 [0.12, 2.29]
González 1995	4	12	3	20	4.4%	2.83 [0.51, 15.77]
Mazure 1994	0	10	4	17	9.7%	0.14 [0.01, 2.96]
**Subtotal (95% CI)**		**77**		**87**	**28.8%**	**0.75 [0.29, 1.97]**
Total events	7		12			
Heterogeneity: $Chi^2 = 3.69, df = 2 (P = 0.16); I^2 = 46\%$						
Test for overall effect: Z = 0.58 (P = 0.56)						
**Total (95% CI)**		**197**		**234**	**100.0%**	**0.33 [0.17, 0.64]**
Total events	14		40			
Heterogeneity: $Chi^2 = 9.26, df = 5 (P = 0.10); I^2 = 46\%$						
Test for overall effect: Z = 3.29 (P = 0.001)						
Test for subgroup differences: $Chi^2 = 4.86, df = 1 (P = 0.03), I^2 = 79.4\%$						

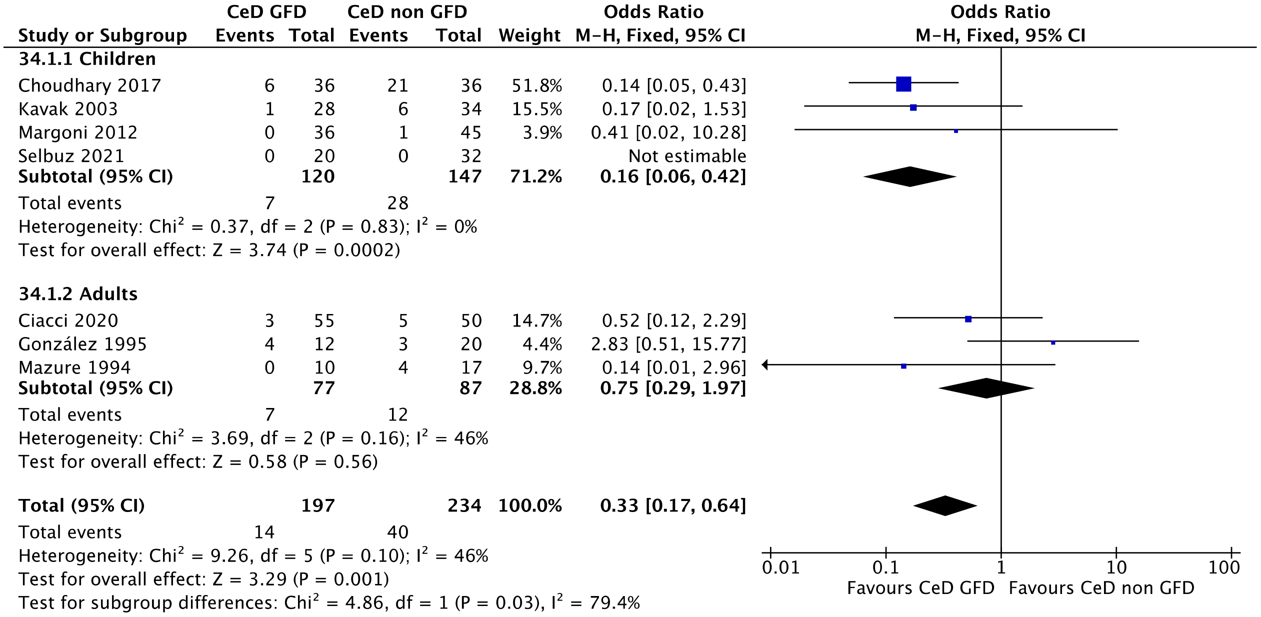

S10E- Forest plot comparison of Calcium deficiency between CeD on GFD and non-CeD controls

Study or Subgroup	CeD GFD Events	CeD GFD Total	Controls Events	Controls Total	Weight	Odds Ratio M-H, Random, 95% CI
Szaflarska-Poplawska 2022	0	48	1	50	100.0%	0.34 [0.01, 8.56]
**Total (95% CI)**		**48**		**50**	**100.0%**	**0.34 [0.01, 8.56]**
Total events	0		1			

Heterogeneity: Not applicable
Test for overall effect: Z = 0.66 (P = 0.51)

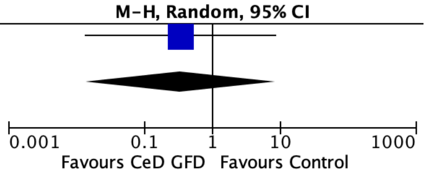

S10Fi- Forest plot comparison of means of serum Calcium levels between CeD on GFD and CeD not on GFD

Study or Subgroup	CeD GFD Mean	CeD GFD SD	CeD GFD Total	CeD non GFD Mean	CeD non GFD SD	CeD non GFD Total	Weight	Mean Difference IV, Random, 95% CI
Bulut, 2023	2.47	0.01	31	2.44	0.02	18	10.2%	0.03 [0.02, 0.04]
Choudhary 2017	2.33	0.16	36	2.18	0.18	36	6.5%	0.15 [0.07, 0.23]
Corazza 1995	2.23	0.09	14	1.75	0.23	17	4.4%	0.48 [0.36, 0.60]
González 1995	2.27	0.12	12	2.32	0.2	20	4.8%	−0.05 [−0.16, 0.06]
Kalayci 2001	2.47	0.12	16	2.35	0.15	16	5.6%	0.12 [0.03, 0.21]
Karkoszka 2000	2.34	0.03	33	2.4	0.03	40	10.1%	−0.06 [−0.07, −0.05]
Kavak 2003	2.47	0.15	28	2.35	0.17	34	6.4%	0.12 [0.04, 0.20]
Keaveny 1996	1.27	0.01	16	1.26	0.02	19	10.2%	0.01 [−0.00, 0.02]
Kemppainen 1995	2.3	0.1	51	2.3	0.1	37	8.8%	0.00 [−0.04, 0.04]
Margoni 2012	2.42	0.07	36	2.37	0.12	45	8.8%	0.05 [0.01, 0.09]
Mazure 1994	2.27	0.02	14	2.25	0.05	20	9.8%	0.02 [−0.00, 0.04]
Sabel'nikova 2013	2.4	0.3	109	2.2	0.2	33	5.9%	0.20 [0.11, 0.29]
Szymczak 2012	2.26	0.11	19	2.07	0.28	16	3.4%	0.19 [0.04, 0.34]
Volkan 2018	2.52	0.22	21	2.4	0.12	26	5.0%	0.12 [0.02, 0.22]
**Total (95% CI)**			**436**			**377**	**100.0%**	**0.07 [0.04, 0.10]**

Heterogeneity: $Tau^2 = 0.00$; $Chi^2 = 232.05$, df = 13 (P < 0.00001); $I^2 = 94\%$
Test for overall effect: Z = 4.26 (P < 0.0001)

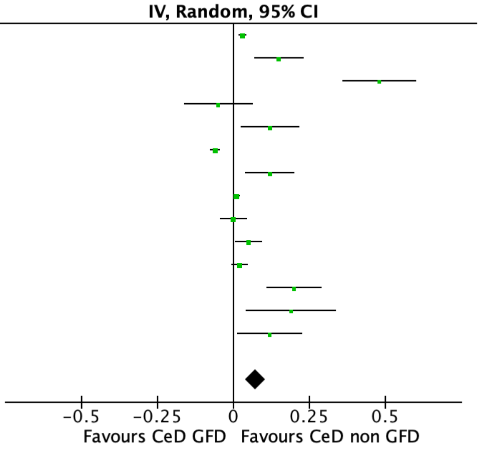

S10Fii- Funnel plot of studies within Fi comparing serum levels of calcium between CeD on GFD and CeD not on GFD

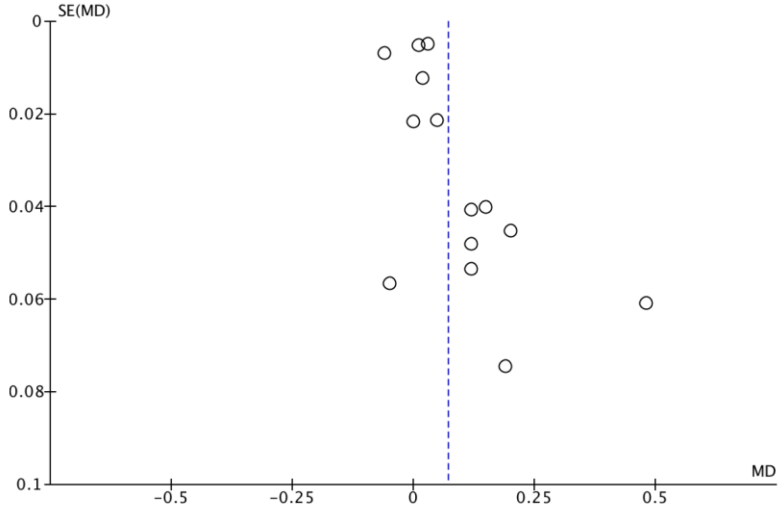

S10G- Forest plot comparison of means of serum Calcium levels between CeD on GFD and CeD not on GFD by study design

	CeD GFD			CeD non GFD				Mean Difference
Study or Subgroup	Mean	SD	Total	Mean	SD	Total	Weight	IV, Random, 95% CI
**32.1.1 Cohort**								
Karkoszka 2000	2.34	0.03	33	2.4	0.03	40	10.1%	−0.06 [−0.07, −0.05]
Keaveny 1996	1.27	0.01	16	1.26	0.02	19	10.2%	0.01 [−0.00, 0.02]
Kemppainen 1995	2.3	0.1	51	2.3	0.1	37	8.8%	0.00 [−0.04, 0.04]
Margoni 2012	2.42	0.07	36	2.37	0.12	45	8.8%	0.05 [0.01, 0.09]
Sabel'nikova 2013	2.4	0.3	109	2.2	0.2	33	5.9%	0.20 [0.11, 0.29]
**Subtotal (95% CI)**			**245**			**174**	**43.9%**	**0.03 [−0.02, 0.08]**
Heterogeneity: $Tau^2 = 0.00$; $Chi^2 = 94.62$, df = 4 ($P < 0.00001$); $I^2 = 96\%$								
Test for overall effect: Z = 1.00 (P = 0.32)								
**32.1.2 Cross-sectional**								
Choudhary 2017	2.33	0.16	36	2.18	0.18	36	6.5%	0.15 [0.07, 0.23]
Corazza 1995	2.23	0.09	14	1.75	0.23	17	4.4%	0.48 [0.36, 0.60]
González 1995	2.27	0.12	12	2.32	0.2	20	4.8%	−0.05 [−0.16, 0.06]
**Subtotal (95% CI)**			**62**			**73**	**15.6%**	**0.19 [−0.08, 0.46]**
Heterogeneity: $Tau^2 = 0.05$; $Chi^2 = 41.41$, df = 2 ($P < 0.00001$); $I^2 = 95\%$								
Test for overall effect: Z = 1.41 (P = 0.16)								
**32.1.3 Case-Control**								
Bulut, 2023	2.47	0.01	31	2.44	0.02	18	10.2%	0.03 [0.02, 0.04]
Kalayci 2001	2.47	0.12	16	2.35	0.15	16	5.6%	0.12 [0.03, 0.21]
Kavak 2003	2.47	0.15	28	2.35	0.17	34	6.4%	0.12 [0.04, 0.20]
Mazure 1994	2.27	0.02	14	2.25	0.05	20	9.8%	0.02 [−0.00, 0.04]
Szymczak 2012	2.26	0.11	19	2.07	0.28	16	3.4%	0.19 [0.04, 0.34]
Volkan 2018	2.52	0.22	21	2.4	0.12	26	5.0%	0.12 [0.02, 0.22]
**Subtotal (95% CI)**			**129**			**130**	**40.5%**	**0.06 [0.03, 0.09]**
Heterogeneity: $Tau^2 = 0.00$; $Chi^2 = 16.35$, df = 5 ($P = 0.006$); $I^2 = 69\%$								
Test for overall effect: Z = 3.54 (P = 0.0004)								
**Total (95% CI)**			**436**			**377**	**100.0%**	**0.07 [0.04, 0.10]**
Heterogeneity: $Tau^2 = 0.00$; $Chi^2 = 232.05$, df = 13 ($P < 0.00001$); $I^2 = 94\%$								
Test for overall effect: Z = 4.26 (P < 0.0001)								
Test for subgroup differences: $Chi^2 = 2.42$, df = 2 (P = 0.30), $I^2 = 17.2\%$								

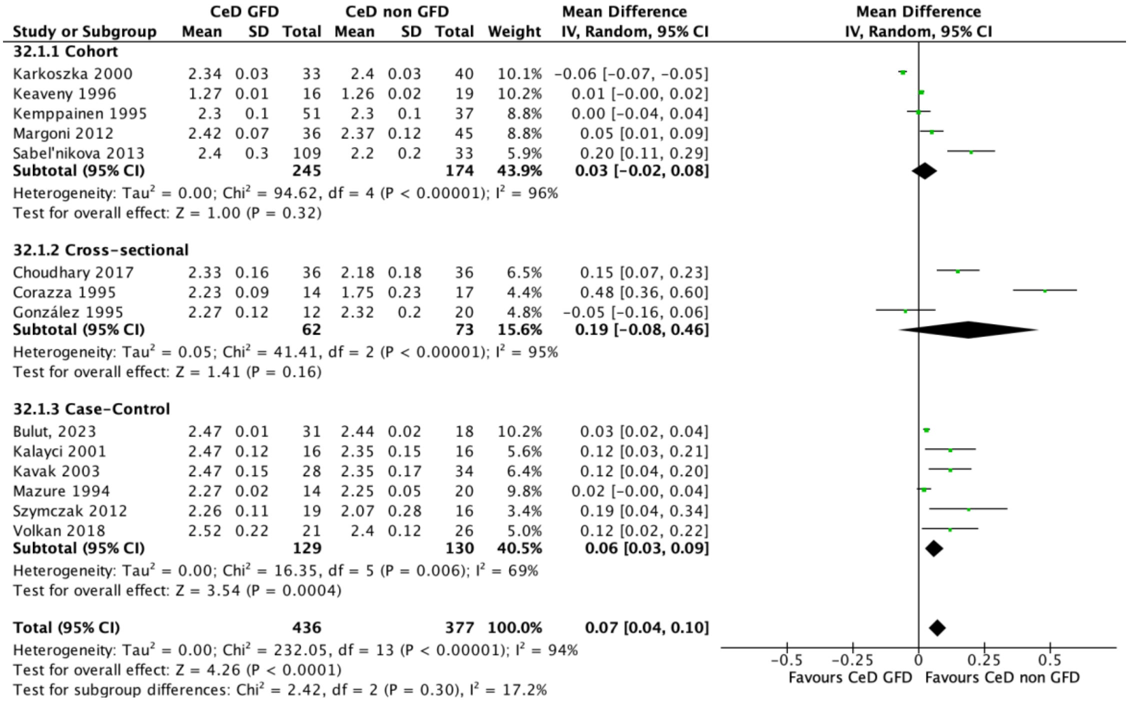

S10H- Forest plot comparison of means of serum Calcium levels between CeD on GFD and CeD not on GFD by GFD length

Study or Subgroup	CeD GFD Mean	CeD GFD SD	CeD GFD Total	CeD non GFD Mean	CeD non GFD SD	CeD non GFD Total	Weight	Mean Difference IV, Random, 95% CI
**71.1.1 Short-Term GFD**								
Bulut, 2023	2.47	0.01	31	2.44	0.02	18	10.2%	0.03 [0.02, 0.04]
Choudhary 2017	2.33	0.16	36	2.18	0.18	36	6.5%	0.15 [0.07, 0.23]
Corazza 1995	2.23	0.09	14	1.75	0.23	17	4.4%	0.48 [0.36, 0.60]
Kavak 2003	2.47	0.15	28	2.35	0.17	34	6.4%	0.12 [0.04, 0.20]
Kemppainen 1995	2.3	0.1	51	2.3	0.1	37	8.8%	0.00 [−0.04, 0.04]
Szymczak 2012	2.26	0.11	19	2.07	0.28	16	3.4%	0.19 [0.04, 0.34]
Volkan 2018	2.52	0.22	21	2.4	0.12	26	5.0%	0.12 [0.02, 0.22]
**Subtotal (95% CI)**			**200**			**184**	**44.8%**	**0.14 [0.06, 0.22]**
Heterogeneity: $Tau^2 = 0.01$; $Chi^2 = 76.69$, df = 6 (P < 0.00001); $I^2 = 92\%$								
Test for overall effect: Z = 3.55 (P = 0.0004)								
**71.1.2 Long-Term GFD**								
González 1995	2.27	0.12	12	2.32	0.2	20	4.8%	−0.05 [−0.16, 0.06]
Kalayci 2001	2.47	0.12	16	2.35	0.15	16	5.6%	0.12 [0.03, 0.21]
Karkoszka 2000	2.34	0.03	33	2.4	0.03	40	10.1%	−0.06 [−0.07, −0.05]
Keaveny 1996	1.27	0.01	16	1.26	0.02	19	10.2%	0.01 [−0.00, 0.02]
Margoni 2012	2.42	0.07	36	2.37	0.12	45	8.8%	0.05 [0.01, 0.09]
Mazure 1994	2.27	0.02	14	2.25	0.05	20	9.8%	0.02 [−0.00, 0.04]
**Subtotal (95% CI)**			**127**			**160**	**49.3%**	**0.01 [−0.03, 0.05]**
Heterogeneity: $Tau^2 = 0.00$; $Chi^2 = 86.31$, df = 5 (P < 0.00001); $I^2 = 94\%$								
Test for overall effect: Z = 0.49 (P = 0.62)								
**71.1.3 Unknown GFD**								
Sabel'nikova 2013	2.4	0.3	109	2.2	0.2	33	5.9%	0.20 [0.11, 0.29]
**Subtotal (95% CI)**			**109**			**33**	**5.9%**	**0.20 [0.11, 0.29]**
Heterogeneity: Not applicable								
Test for overall effect: Z = 4.43 (P < 0.00001)								
**Total (95% CI)**			**436**			**377**	**100.0%**	**0.07 [0.04, 0.10]**
Heterogeneity: $Tau^2 = 0.00$; $Chi^2 = 232.05$, df = 13 (P < 0.00001); $I^2 = 94\%$								
Test for overall effect: Z = 4.26 (P < 0.0001)								
Test for subgroup differences: $Chi^2 = 19.14$, df = 2 (P < 0.0001), $I^2 = 89.6\%$								

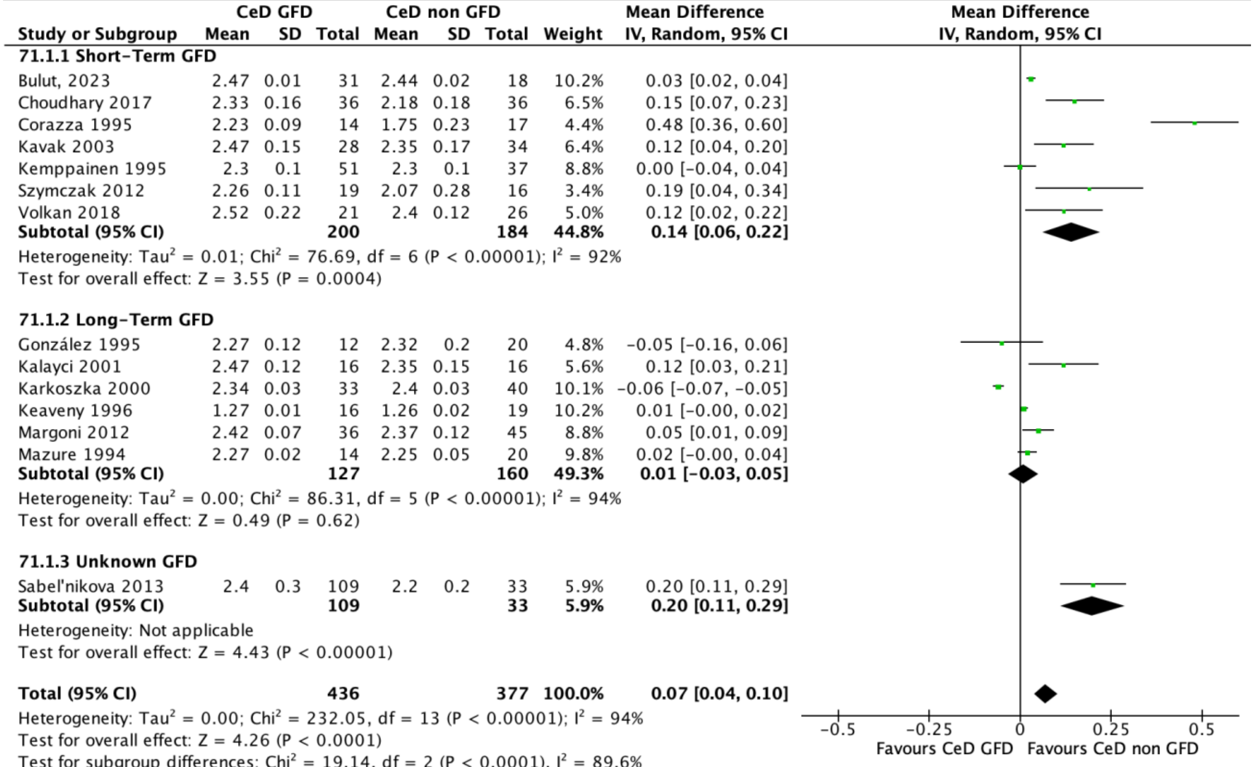

S10I- Forest plot comparison of means of serum Calcium levels between CeD on GFD and CeD not on GFD by population

Study or Subgroup	CeD GFD Mean	CeD GFD SD	CeD GFD Total	CeD non GFD Mean	CeD non GFD SD	CeD non GFD Total	Weight	Mean Difference IV, Random, 95% CI
**46.1.1 Children**								
Bulut, 2023	2.47	0.01	31	2.44	0.02	18	10.2%	0.03 [0.02, 0.04]
Choudhary 2017	2.33	0.16	36	2.18	0.18	36	6.5%	0.15 [0.07, 0.23]
Kalayci 2001	2.47	0.12	16	2.35	0.15	16	5.6%	0.12 [0.03, 0.21]
Karkoszka 2000	2.34	0.03	33	2.4	0.03	40	10.1%	−0.06 [−0.07, −0.05]
Kavak 2003	2.47	0.15	28	2.35	0.17	34	6.4%	0.12 [0.04, 0.20]
Kemppainen 1995	2.3	0.1	51	2.3	0.1	37	8.8%	0.00 [−0.04, 0.04]
Margoni 2012	2.42	0.07	36	2.37	0.12	45	8.8%	0.05 [0.01, 0.09]
Volkan 2018	2.52	0.22	21	2.4	0.12	26	5.0%	0.12 [0.02, 0.22]
**Subtotal (95% CI)**			**252**			**252**	**61.6%**	**0.05 [0.01, 0.10]**
Heterogeneity: $Tau^2 = 0.00$; $Chi^2 = 145.34$, df = 7 (P < 0.00001); $I^2 = 95\%$								
Test for overall effect: Z = 2.23 (P = 0.03)								
**46.1.2 Adults**								
Corazza 1995	2.23	0.09	14	1.75	0.23	17	4.4%	0.48 [0.36, 0.60]
González 1995	2.27	0.12	12	2.32	0.2	20	4.8%	−0.05 [−0.16, 0.06]
Keaveny 1996	1.27	0.01	16	1.26	0.02	19	10.2%	0.01 [−0.00, 0.02]
Mazure 1994	2.27	0.02	14	2.25	0.05	20	9.8%	0.02 [−0.00, 0.04]
Sabel'nikova 2013	2.4	0.3	109	2.2	0.2	33	5.9%	0.20 [0.11, 0.29]
Szymczak 2012	2.26	0.11	19	2.07	0.28	16	3.4%	0.19 [0.04, 0.34]
**Subtotal (95% CI)**			**184**			**125**	**38.4%**	**0.12 [0.05, 0.19]**
Heterogeneity: $Tau^2 = 0.01$; $Chi^2 = 83.18$, df = 5 (P < 0.00001); $I^2 = 94\%$								
Test for overall effect: Z = 3.31 (P = 0.0009)								
**Total (95% CI)**			**436**			**377**	**100.0%**	**0.07 [0.04, 0.10]**
Heterogeneity: $Tau^2 = 0.00$; $Chi^2 = 232.05$, df = 13 (P < 0.00001); $I^2 = 94\%$								
Test for overall effect: Z = 4.26 (P < 0.0001)								
Test for subgroup differences: $Chi^2 = 2.22$, df = 1 (P = 0.14), $I^2 = 54.9\%$								

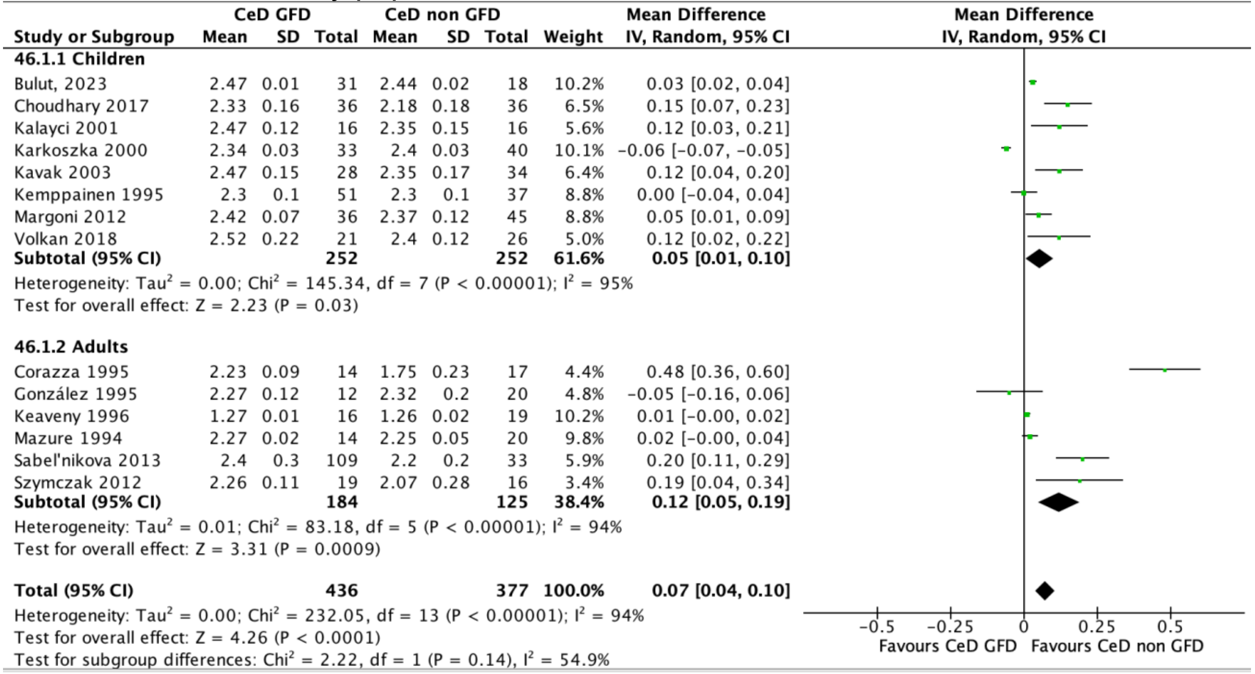

S10J- Forest plot comparison of means of serum calcium levels between CeD on GFD and non-CeD controls

Study or Subgroup	CeD GFD Mean	CeD GFD SD	CeD GFD Total	Controls Mean	Controls SD	Controls Total	Weight	Mean Difference IV, Random, 95% CI
Ballestero-Fernández 2019	2.45	0.07	70	2.44	0.05	67	15.3%	0.01 [−0.01, 0.03]
Ballestero-Fernández 2021	2.34	0.1	64	2.32	0.11	74	14.5%	0.02 [−0.02, 0.06]
Corazza 1995	2.23	0.09	14	2.38	0.06	24	13.2%	−0.15 [−0.20, −0.10]
Kalayci 2001	2.47	0.12	16	2.45	0.15	82	12.0%	0.02 [−0.05, 0.09]
Pazianas 2005	2.3	0.12	24	2.3	0.06	20	13.0%	0.00 [−0.05, 0.05]
Szaflarska-Poplawska 2022	2.4	0.1	48	2.5	0.1	50	14.2%	−0.10 [−0.14, −0.06]
Szymczak 2012	2.26	0.11	19	2.29	0.14	36	12.0%	−0.03 [−0.10, 0.04]
Volkan 2018	2.52	0.22	21	2.37	0.35	30	5.8%	0.15 [−0.01, 0.31]
**Total (95% CI)**			**276**			**383**	**100.0%**	**−0.02 [−0.07, 0.03]**

Heterogeneity: Tau^2 = 0.00; Chi^2 = 58.14, df = 7 (P < 0.00001); I^2 = 88%
Test for overall effect: Z = 0.91 (P = 0.36)

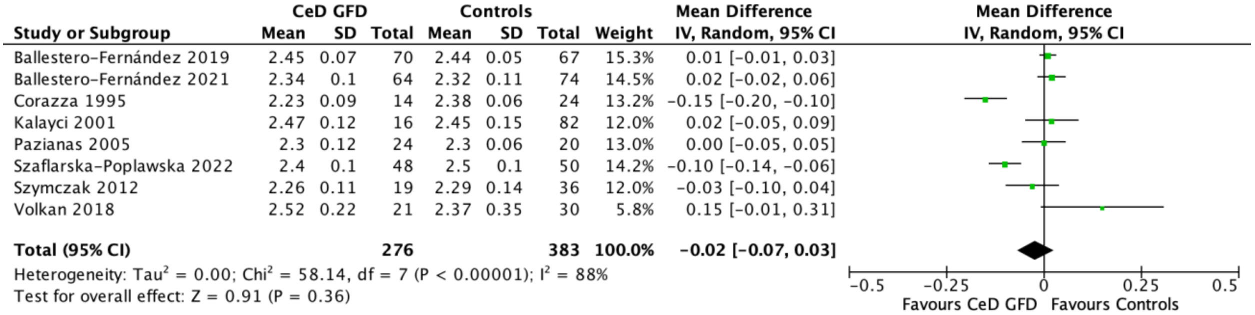

S10K- Forest plot comparison of means of serum calcium levels between CeD on GFD and non-CeD controls by study design

Study or Subgroup	CeD GFD Mean	CeD GFD SD	CeD GFD Total	Controls Mean	Controls SD	Controls Total	Weight	Mean Difference IV, Random, 95% CI
**25.2.1 Cross-sectional**								
Ballestero-Fernández 2019	2.45	0.07	67	2.44	0.05	66	15.3%	0.01 [−0.01, 0.03]
Ballestero-Fernández 2021	2.34	0.1	64	2.32	0.11	74	14.5%	0.02 [−0.02, 0.06]
Corazza 1995	2.23	0.09	14	2.38	0.06	24	13.2%	−0.15 [−0.20, −0.10]
**Subtotal (95% CI)**			**145**			**164**	**43.0%**	**−0.04 [−0.12, 0.04]**
Heterogeneity: Tau^2 = 0.00; Chi^2 = 32.82, df = 2 (P < 0.00001); I^2 = 94%								
Test for overall effect: Z = 0.89 (P = 0.37)								
**25.2.2 Case-Control**								
Kalayci 2001	2.47	0.12	16	2.45	0.15	82	12.0%	0.02 [−0.05, 0.09]
Pazianas 2005	2.3	0.12	24	2.3	0.06	20	13.0%	0.00 [−0.05, 0.05]
Szymczak 2012	2.26	0.11	19	2.29	0.14	36	12.0%	−0.03 [−0.10, 0.04]
Volkan 2018	2.52	0.22	21	2.37	0.35	30	5.8%	0.15 [−0.01, 0.31]
**Subtotal (95% CI)**			**80**			**168**	**42.8%**	**0.01 [−0.04, 0.05]**
Heterogeneity: Tau^2 = 0.00; Chi^2 = 4.55, df = 3 (P = 0.21); I^2 = 34%								
Test for overall effect: Z = 0.37 (P = 0.71)								
**25.2.3 Cohort**								
Szaflarska-Poplawska 2022	2.4	0.1	48	2.5	0.1	50	14.2%	−0.10 [−0.14, −0.06]
**Subtotal (95% CI)**			**48**			**50**	**14.2%**	**−0.10 [−0.14, −0.06]**
Heterogeneity: Not applicable								
Test for overall effect: Z = 4.95 (P < 0.00001)								
**Total (95% CI)**			**273**			**382**	**100.0%**	**−0.02 [−0.07, 0.03]**

Heterogeneity: Tau^2 = 0.00; Chi^2 = 57.95, df = 7 (P < 0.00001); I^2 = 88%
Test for overall effect: Z = 0.91 (P = 0.36)
Test for subgroup differences: Chi^2 = 12.59, df = 2 (P = 0.002), I^2 = 84.1%

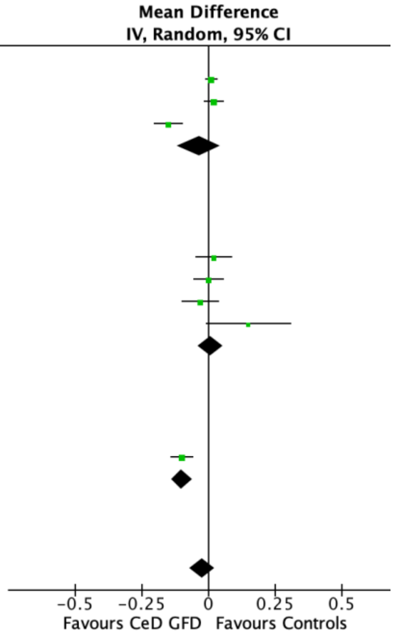

S10L- Forest plot comparison of means of serum calcium levels between CeD on GFD and non-CeD controls by GFD length

Study or Subgroup	CeD GFD Mean	CeD GFD SD	CeD GFD Total	Controls Mean	Controls SD	Controls Total	Weight	Mean Difference IV, Random, 95% CI
**50.2.1 Short-Term GFD**								
Ballestero-Fernández 2019	2.45	0.07	70	2.44	0.05	67	15.3%	0.01 [−0.01, 0.03]
Ballestero-Fernández 2021	2.34	0.1	64	2.32	0.11	74	14.5%	0.02 [−0.02, 0.06]
Corazza 1995	2.23	0.09	14	2.38	0.06	24	13.2%	−0.15 [−0.20, −0.10]
Szymczak 2012	2.26	0.11	19	2.29	0.14	36	12.0%	−0.03 [−0.10, 0.04]
Volkan 2018	2.52	0.22	21	2.37	0.35	30	5.8%	0.15 [−0.01, 0.31]
**Subtotal (95% CI)**			**188**			**231**	**60.8%**	**−0.02 [−0.08, 0.05]**
Heterogeneity: Tau^2 = 0.00; Chi^2 = 37.17, df = 4 (P < 0.00001); I^2 = 89%								
Test for overall effect: Z = 0.50 (P = 0.61)								
**50.2.2 Long-Term GFD**								
Kalayci 2001	2.47	0.12	16	2.45	0.15	82	12.0%	0.02 [−0.05, 0.09]
Pazianas 2005	2.3	0.12	24	2.3	0.06	20	13.0%	0.00 [−0.05, 0.05]
Szaflarska-Poplawska 2022	2.4	0.1	48	2.5	0.1	50	14.2%	−0.10 [−0.14, −0.06]
**Subtotal (95% CI)**			**88**			**152**	**39.2%**	**−0.03 [−0.11, 0.05]**
Heterogeneity: Tau^2 = 0.00; Chi^2 = 13.50, df = 2 (P = 0.001); I^2 = 85%								
Test for overall effect: Z = 0.73 (P = 0.46)								
**Total (95% CI)**			**276**			**383**	**100.0%**	**−0.02 [−0.07, 0.03]**

Heterogeneity: Tau^2 = 0.00; Chi^2 = 58.14, df = 7 (P < 0.00001); I^2 = 88%
Test for overall effect: Z = 0.91 (P = 0.36)
Test for subgroup differences: Chi^2 = 0.07, df = 1 (P = 0.79), I^2 = 0%

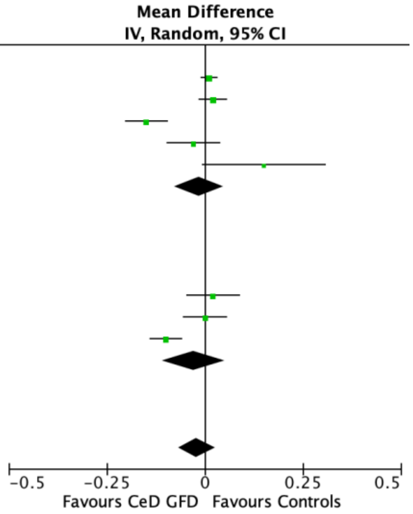

S10M- Forest plot comparison of means of serum calcium levels between CeD on GFD and non-CeD controls by population

Study or Subgroup	CeD GFD Mean	CeD GFD SD	CeD GFD Total	Controls Mean	Controls SD	Controls Total	Weight	Mean Difference IV, Random, 95% CI
**32.2.1 Children**								
Ballestero-Fernández 2019	2.45	0.07	67	2.44	0.05	66	15.3%	0.01 [-0.01, 0.03]
Kalayci 2001	2.47	0.12	16	2.45	0.15	82	12.0%	0.02 [-0.05, 0.09]
Szaflarska-Poplawska 2022	2.4	0.1	48	2.5	0.1	50	14.2%	-0.10 [-0.14, -0.06]
Volkan 2018	2.52	0.22	21	2.37	0.35	30	5.8%	0.15 [-0.01, 0.31]
**Subtotal (95% CI)**			**152**			**228**	**47.3%**	**-0.00 [-0.08, 0.07]**
Heterogeneity: $Tau^2 = 0.00$; $Chi^2 = 28.21$, df = 3 ($P < 0.00001$); $I^2 = 89\%$								
Test for overall effect: Z = 0.02 (P = 0.98)								
**32.2.2 Adults**								
Ballestero-Fernández 2021	2.34	0.1	64	2.32	0.11	74	14.5%	0.02 [-0.02, 0.06]
Corazza 1995	2.23	0.09	14	2.38	0.06	24	13.2%	-0.15 [-0.20, -0.10]
Pazianas 2005	2.3	0.12	24	2.3	0.06	20	13.0%	0.00 [-0.05, 0.05]
Szymczak 2012	2.26	0.11	19	2.29	0.14	36	12.0%	-0.03 [-0.10, 0.04]
**Subtotal (95% CI)**			**121**			**154**	**52.7%**	**-0.04 [-0.12, 0.04]**
Heterogeneity: $Tau^2 = 0.01$; $Chi^2 = 28.60$, df = 3 ($P < 0.00001$); $I^2 = 90\%$								
Test for overall effect: Z = 0.98 (P = 0.33)								
**Total (95% CI)**			**273**			**382**	**100.0%**	**-0.02 [-0.07, 0.03]**
Heterogeneity: $Tau^2 = 0.00$; $Chi^2 = 57.95$, df = 7 ($P < 0.00001$); $I^2 = 88\%$								
Test for overall effect: Z = 0.91 (P = 0.36)								
Test for subgroup differences: $Chi^2 = 0.47$, df = 1 (P = 0.49), $I^2 = 0\%$								

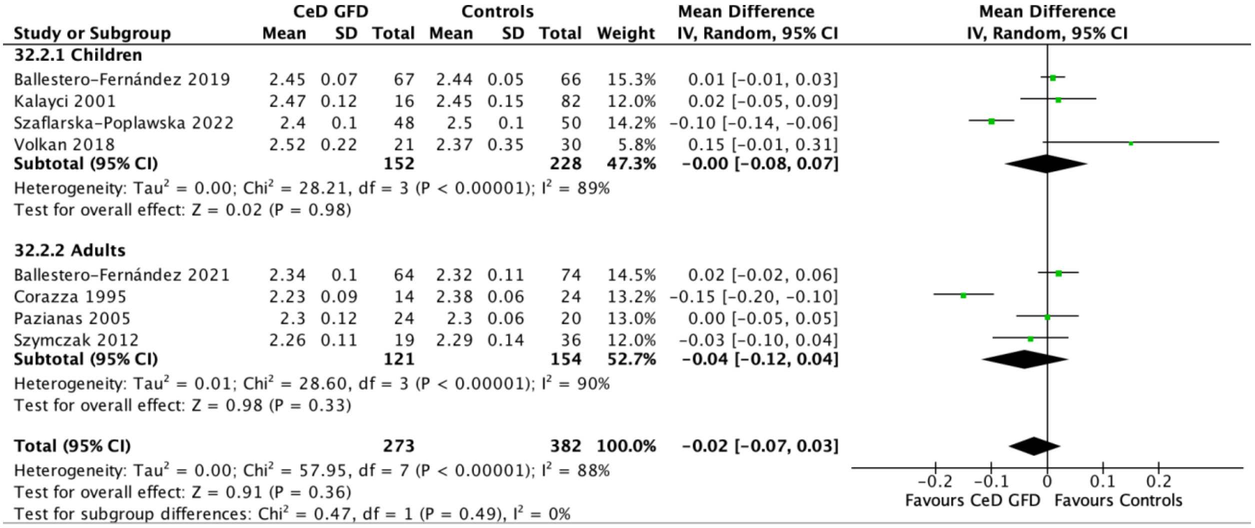

Supplementary Figure S11. Copper

S11A- Forest plot comparison of Copper deficiency between CeD on GFD and CeD not on GFD

Study or Subgroup	CeD GFD Events	CeD GFD Total	CeD non GFD Events	CeD non GFD Total	Weight	Odds Ratio M-H, Random, 95% CI
McGrogan 2021	6	41	1	24	100.0%	3.94 [0.45, 34.93]
**Total (95% CI)**		**41**		**24**	**100.0%**	**3.94 [0.45, 34.93]**
Total events	6		1			
Heterogeneity: Not applicable						
Test for overall effect: Z = 1.23 (P = 0.22)						

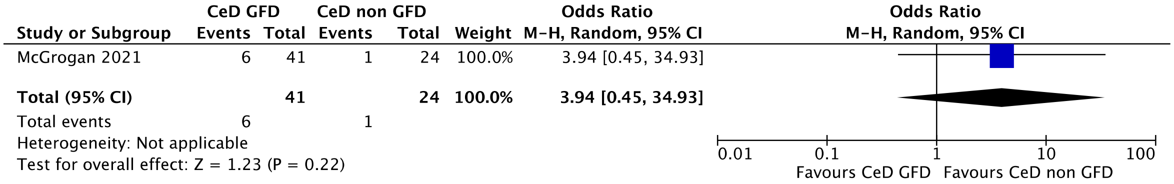

S11B- Forest plot comparison of means of serum copper levels between CeD on GFD and CeD not on GFD

Study or Subgroup	CeD GFD Mean	CeD GFD SD	CeD GFD Total	CeD non GFD Mean	CeD non GFD SD	CeD non GFD Total	Weight	Mean Difference IV, Random, 95% CI
McGrogan 2021	16.37	3.93	41	16.8	3.24	24	100.0%	-0.43 [-2.20, 1.34]
**Total (95% CI)**			**41**			**24**	**100.0%**	**-0.43 [-2.20, 1.34]**
Heterogeneity: Not applicable								
Test for overall effect: Z = 0.48 (P = 0.63)								

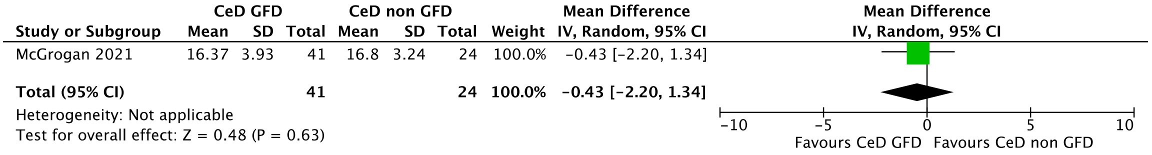

Supplementary Figure S12. Folate

S12A- Forest plot comparison of serum Folate deficiency between CeD on GFD and CeD not on GFD

Study or Subgroup	CeD GFD Events	CeD GFD Total	CeD CeD non GFD Events	CeD CeD non GFD Total	Weight	Risk Ratio M-H, Random, 95% CI
McGrogan 2021	0	13	0	9		Not estimable
Selbuz 2021	0	18	3	27	100.0%	0.21 [0.01, 3.85]
**Total (95% CI)**		**31**		**36**	**100.0%**	**0.21 [0.01, 3.85]**
Total events	0		3			

Heterogeneity: Not applicable
Test for overall effect: Z = 1.05 (P = 0.29)

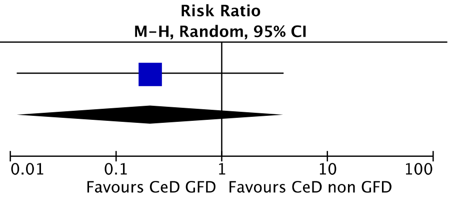

S12B- Forest plot comparison of Folate deficiency in erythrocytes between CeD on GFD and CeD not on GFD

Study or Subgroup	CeD GFD Events	CeD GFD Total	CeD CeD non GFD Events	CeD CeD non GFD Total	Weight	Odds Ratio M-H, Random, 95% CI
Kemppainen 1995	2	52	14	40	100.0%	0.07 [0.02, 0.35]
**Total (95% CI)**		**52**		**40**	**100.0%**	**0.07 [0.02, 0.35]**
Total events	2		14			

Heterogeneity: Not applicable
Test for overall effect: Z = 3.28 (P = 0.001)

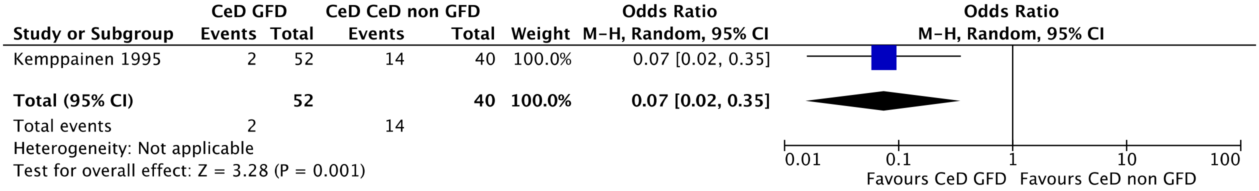

S12C- Forest plot comparison of serum Folate deficiency between CeD on GFD and non-CeD controls

Study or Subgroup	CeD GFD Events	CeD GFD Total	Controls Events	Controls Total	Weight	Odds Ratio M-H, Random, 95% CI
Bayrak 2020	14	103	8	135	37.8%	2.50 [1.01, 6.20]
Mansueto 2023	75	174	0	124	26.3%	188.94 [11.57, 3086.71]
Szaflarska-Poplawska 2022	7	48	4	50	35.9%	1.96 [0.54, 7.19]
**Total (95% CI)**		**325**		**309**	**100.0%**	**7.16 [0.61, 84.15]**
Total events	96		12			

Heterogeneity: $Tau^2 = 3.97$; $Chi^2 = 16.96$, df = 2 (P = 0.0002); $I^2 = 88\%$
Test for overall effect: Z = 1.57 (P = 0.12)

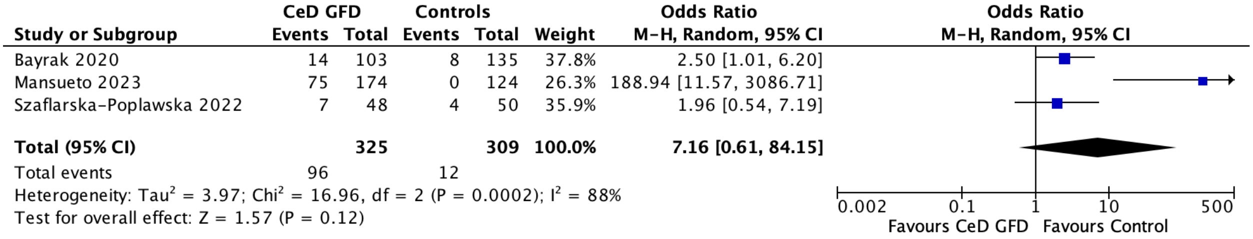

S12D- Forest plot comparison of means of serum Folate levels between CeD on GFD and CeD not on GFD

Study or Subgroup	CeD GFD Mean	CeD GFD SD	CeD GFD Total	CeD non GFD Mean	CeD non GFD SD	CeD non GFD Total	Weight	Mean Difference IV, Random, 95% CI
Dickey 2008	25.7	31.5	41	12.9	10	35	23.9%	12.80 [2.60, 23.00]
McGrogan 2021	23.11	11.6	13	24.92	10.53	9	26.5%	-1.81 [-11.14, 7.52]
Volkan 2018	18.12	5.1	21	13.6	8.5	26	49.6%	4.52 [0.59, 8.45]
**Total (95% CI)**			**75**			**70**	**100.0%**	**4.82 [-1.57, 11.21]**

Heterogeneity: $Tau^2 = 17.39$; $Chi^2 = 4.30$, df = 2 (P = 0.12); $I^2 = 53\%$
Test for overall effect: Z = 1.48 (P = 0.14)

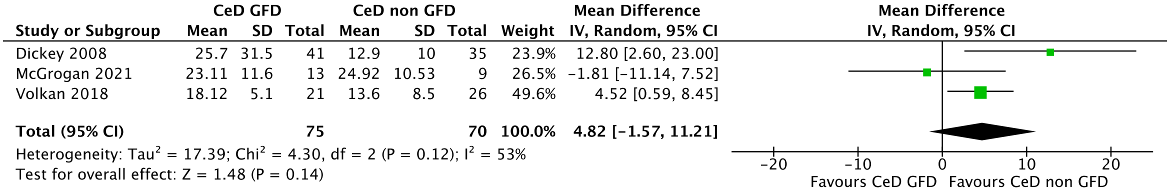

S12E- Forest plot comparison of means of serum Folate levels between CeD on GFD and CeD not on GFD by study design

Study or Subgroup	CeD GFD Mean	CeD GFD SD	CeD GFD Total	CeD non GFD Mean	CeD non GFD SD	CeD non GFD Total	Weight	Mean Difference IV, Random, 95% CI
**27.1.1 Cohort**								
McGrogan 2021	23.11	11.6	13	24.92	10.53	9	26.5%	−1.81 [−11.14, 7.52]
**Subtotal (95% CI)**			**13**			**9**	**26.5%**	**−1.81 [−11.14, 7.52]**
Heterogeneity: Not applicable								
Test for overall effect: Z = 0.38 (P = 0.70)								
**27.1.3 Case-Control**								
Dickey 2008	25.7	31.5	41	12.9	10	35	23.9%	12.80 [2.60, 23.00]
Volkan 2018	18.12	5.1	21	13.6	8.5	26	49.6%	4.52 [0.59, 8.45]
**Subtotal (95% CI)**			**62**			**61**	**73.5%**	**7.27 [−0.37, 14.91]**
Heterogeneity: Tau^2 = 18.74; Chi^2 = 2.21, df = 1 (P = 0.14); I^2 = 55%								
Test for overall effect: Z = 1.86 (P = 0.06)								
**Total (95% CI)**			**75**			**70**	**100.0%**	**4.82 [−1.57, 11.21]**
Heterogeneity: Tau^2 = 17.39; Chi^2 = 4.30, df = 2 (P = 0.12); I^2 = 53%								
Test for overall effect: Z = 1.48 (P = 0.14)								
Test for subgroup differences: Chi^2 = 2.18, df = 1 (P = 0.14), I^2 = 54.0%								

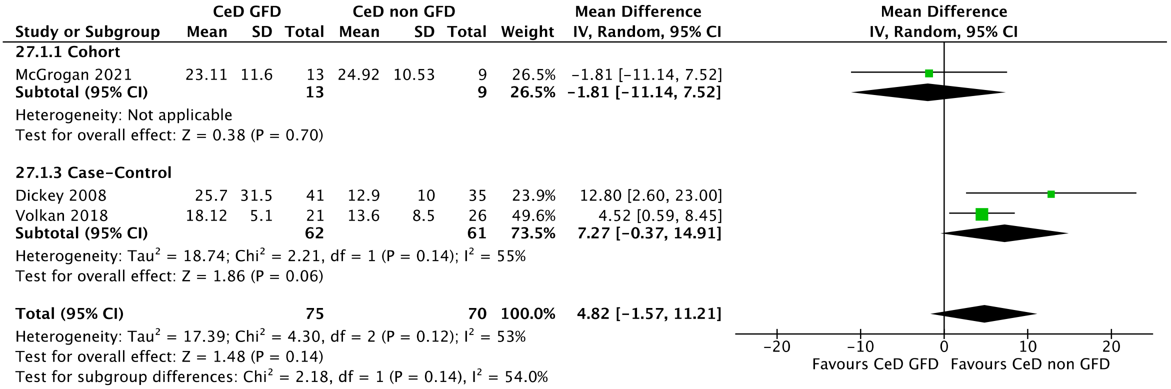

S12F- Forest plot comparison of means of serum Folate levels between CeD on GFD and CeD not on GFD by population

Study or Subgroup	CeD GFD Mean	CeD GFD SD	CeD GFD Total	CeD non GFD Mean	CeD non GFD SD	CeD non GFD Total	Weight	Mean Difference IV, Random, 95% CI
**44.1.1 Children**								
McGrogan 2021	23.11	11.6	13	24.92	10.53	9	26.5%	−1.81 [−11.14, 7.52]
Volkan 2018	18.12	5.1	21	13.6	8.5	26	49.6%	4.52 [0.59, 8.45]
**Subtotal (95% CI)**			**34**			**35**	**76.1%**	**2.83 [−2.66, 8.32]**
Heterogeneity: Tau^2 = 6.69; Chi^2 = 1.50, df = 1 (P = 0.22); I^2 = 33%								
Test for overall effect: Z = 1.01 (P = 0.31)								
**44.1.2 Adults**								
Dickey 2008	25.7	31.5	41	12.9	10	35	23.9%	12.80 [2.60, 23.00]
**Subtotal (95% CI)**			**41**			**35**	**23.9%**	**12.80 [2.60, 23.00]**
Heterogeneity: Not applicable								
Test for overall effect: Z = 2.46 (P = 0.01)								
**Total (95% CI)**			**75**			**70**	**100.0%**	**4.82 [−1.57, 11.21]**
Heterogeneity: Tau^2 = 17.39; Chi^2 = 4.30, df = 2 (P = 0.12); I^2 = 53%								
Test for overall effect: Z = 1.48 (P = 0.14)								
Test for subgroup differences: Chi^2 = 2.85, df = 1 (P = 0.09), I^2 = 64.9%								

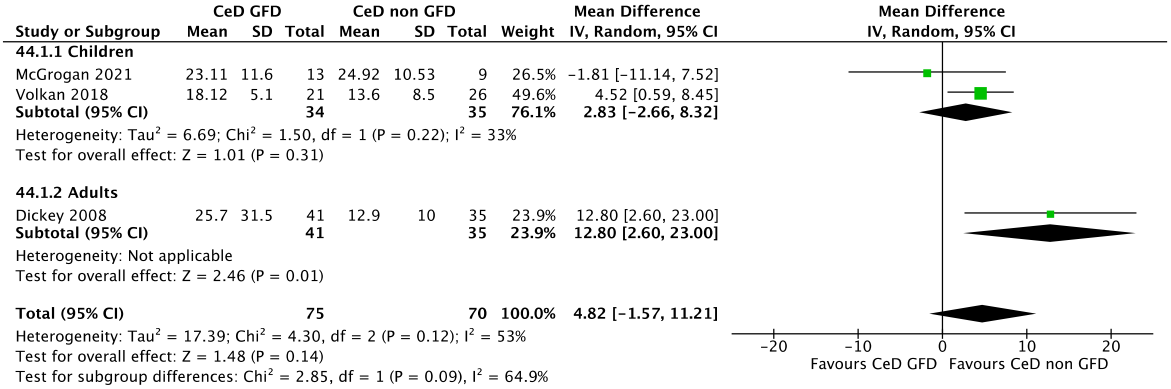

S12G- Forest plot comparison of means of serum Folate levels between CeD on GFD and non-CeD controls

Study or Subgroup	CeD GFD Mean	CeD GFD SD	CeD GFD Total	Controls Mean	Controls SD	Controls Total	Weight	Mean Difference IV, Random, 95% CI
Ballestero-Fernández 2019	15.18	8.06	70	14.12	6.21	67	12.1%	1.06 [−1.34, 3.46]
Ballestero-Fernández 2021	17.06	10.07	64	14.74	6.75	74	11.9%	2.32 [−0.59, 5.23]
Bayrak 2020	19.64	13.71	103	18.53	6.09	135	11.9%	1.11 [−1.73, 3.95]
Dickey 2008	25.7	31.5	41	24.1	14.2	200	7.9%	1.60 [−8.24, 11.44]
Isikay 2018	28.64	36.43	226	37.38	27.5	268	10.4%	−8.74 [−14.52, −2.96]
Mansueto 2023	11.8	6.14	174	23.64	8.86	124	12.3%	−11.84 [−13.65, −10.03]
Szaflarska-Poplawska 2022	17.95	6.59	48	23.18	9.77	50	11.7%	−5.23 [−8.52, −1.94]
Valente 2015	17.5	8	20	29	9.4	39	11.1%	−11.50 [−16.08, −6.92]
Volkan 2018	18.12	5.1	21	18.51	14.04	30	10.6%	−0.39 [−5.87, 5.09]
**Total (95% CI)**			**767**			**987**	**100.0%**	**−3.63 [−8.21, 0.94]**
Heterogeneity: Tau^2 = 43.59; Chi^2 = 134.90, df = 8 (P < 0.00001); I^2 = 94%								
Test for overall effect: Z = 1.56 (P = 0.12)								

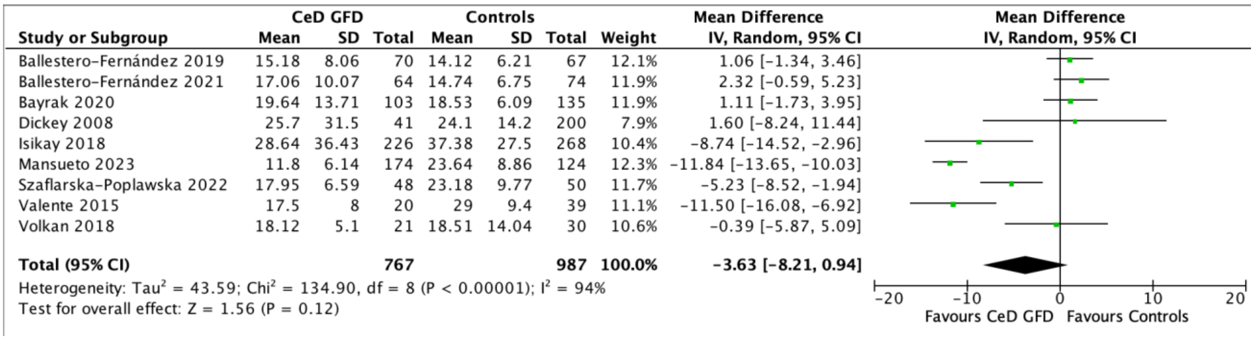

S12H- Forest plot comparison of means of serum Folate levels between CeD on GFD and non-CeD controls by study design

Study or Subgroup	CeD GFD Mean	CeD GFD SD	CeD GFD Total	Controls Mean	Controls SD	Controls Total	Weight	Mean Difference IV, Random, 95% CI
**20.1.1 Cohort**								
Szaflarska-Poplawska 2022	17.95	6.59	48	23.18	9.77	50	11.7%	-5.23 [-8.52, -1.94]
**Subtotal (95% CI)**			**48**			**50**	**11.7%**	**-5.23 [-8.52, -1.94]**
Heterogeneity: Not applicable								
Test for overall effect: Z = 3.12 (P = 0.002)								
**20.1.2 Cross-sectional**								
Ballestero-Fernández 2019	15.18	8.06	67	14.12	6.21	66	12.1%	1.06 [-1.38, 3.50]
Ballestero-Fernández 2021	17.06	10.07	64	14.74	6.75	74	11.9%	2.32 [-0.59, 5.23]
Bayrak 2020	19.64	13.71	103	18.53	6.09	135	11.9%	1.11 [-1.73, 3.95]
Isikay 2018	28.64	36.43	226	37.38	27.5	268	10.4%	-8.74 [-14.52, -2.96]
Valente 2015	17.5	8	20	29	9.4	39	11.1%	-11.50 [-16.08, -6.92]
**Subtotal (95% CI)**			**480**			**582**	**57.5%**	**-2.68 [-7.23, 1.87]**
Heterogeneity: $Tau^2 = 23.20$; $Chi^2 = 36.36$, df = 4 (P < 0.00001); $I^2 = 89\%$								
Test for overall effect: Z = 1.15 (P = 0.25)								
**20.1.3 Case-Control**								
Dickey 2008	25.7	31.5	41	24.1	14.2	200	7.9%	1.60 [-8.24, 11.44]
Mansueto 2023	11.8	6.14	174	23.64	8.86	124	12.3%	-11.84 [-13.65, -10.03]
Volkan 2018	18.12	5.1	21	18.51	14.04	30	10.6%	-0.39 [-5.87, 5.09]
**Subtotal (95% CI)**			**236**			**354**	**30.8%**	**-4.20 [-13.87, 5.46]**
Heterogeneity: $Tau^2 = 63.03$; $Chi^2 = 20.98$, df = 2 (P < 0.0001); $I^2 = 90\%$								
Test for overall effect: Z = 0.85 (P = 0.39)								
**Total (95% CI)**			**764**			**986**	**100.0%**	**-3.63 [-8.21, 0.94]**
Heterogeneity: $Tau^2 = 43.61$; $Chi^2 = 134.20$, df = 8 (P < 0.00001); $I^2 = 94\%$								
Test for overall effect: Z = 1.56 (P = 0.12)								
Test for subgroup differences: $Chi^2 = 0.79$, df = 2 (P = 0.67), $I^2 = 0\%$								

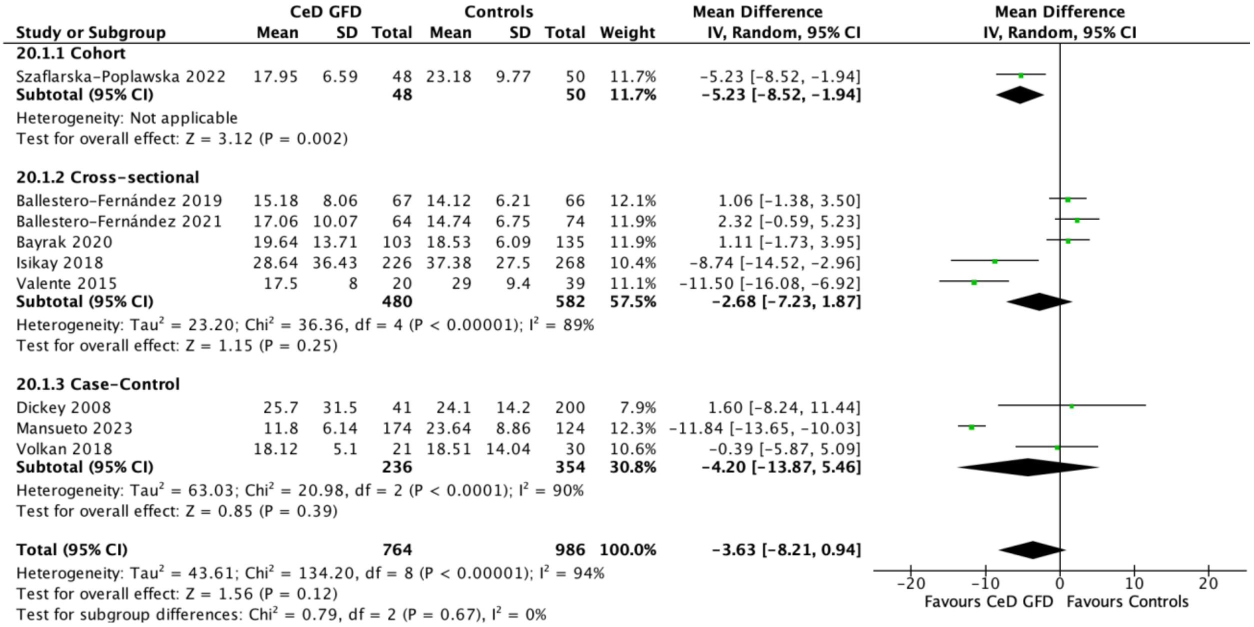

S12I- Forest plot comparison of means of serum Folate levels between CeD on GFD and non-CeD controls by GFD length

Study or Subgroup	CeD GFD Mean	CeD GFD SD	CeD GFD Total	Controls Mean	Controls SD	Controls Total	Weight	Mean Difference IV, Random, 95% CI
**52.1.2 Short-Term GFD**								
Ballestero-Fernández 2019	15.18	8.06	67	14.12	6.21	66	12.1%	1.06 [-1.38, 3.50]
Ballestero-Fernández 2021	17.06	10.07	64	14.74	6.75	74	11.9%	2.32 [-0.59, 5.23]
Dickey 2008	25.7	31.5	41	24.1	14.2	200	7.9%	1.60 [-8.24, 11.44]
Isikay 2018	28.64	36.43	226	37.38	27.5	268	10.4%	-8.74 [-14.52, -2.96]
Mansueto 2023	11.8	6.14	174	23.64	8.86	124	12.3%	-11.84 [-13.65, -10.03]
Valente 2015	17.5	8	20	29	9.4	39	11.1%	-11.50 [-16.08, -6.92]
Volkan 2018	18.12	5.1	21	18.51	14.04	30	10.6%	-0.39 [-5.87, 5.09]
**Subtotal (95% CI)**			**613**			**801**	**76.3%**	**-4.10 [-9.93, 1.73]**
Heterogeneity: $Tau^2 = 55.37$; $Chi^2 = 115.91$, df = 6 (P < 0.00001); $I^2 = 95\%$								
Test for overall effect: Z = 1.38 (P = 0.17)								
**52.1.3 Long-Term GFD**								
Bayrak 2020	19.64	13.71	103	18.53	6.09	135	11.9%	1.11 [-1.73, 3.95]
Szaflarska-Poplawska 2022	17.95	6.59	48	23.18	9.77	50	11.7%	-5.23 [-8.52, -1.94]
**Subtotal (95% CI)**			**151**			**185**	**23.7%**	**-2.00 [-8.22, 4.21]**
Heterogeneity: $Tau^2 = 17.64$; $Chi^2 = 8.18$, df = 1 (P = 0.004); $I^2 = 88\%$								
Test for overall effect: Z = 0.63 (P = 0.53)								
**Total (95% CI)**			**764**			**986**	**100.0%**	**-3.63 [-8.21, 0.94]**
Heterogeneity: $Tau^2 = 43.61$; $Chi^2 = 134.20$, df = 8 (P < 0.00001); $I^2 = 94\%$								
Test for overall effect: Z = 1.56 (P = 0.12)								
Test for subgroup differences: $Chi^2 = 0.23$, df = 1 (P = 0.63), $I^2 = 0\%$								

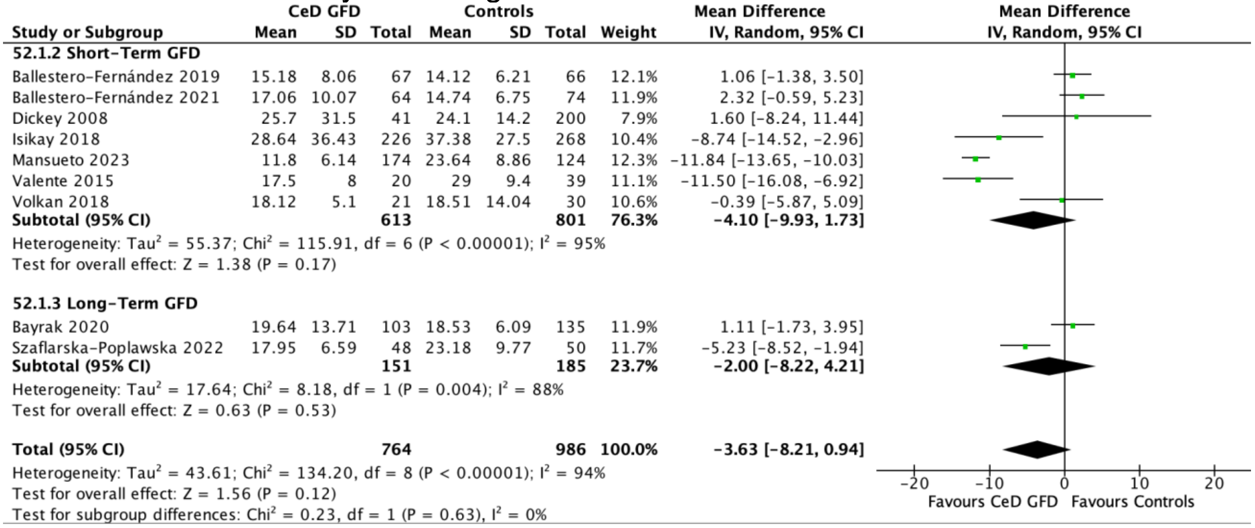

S12J- Forest plot comparison of means of serum Folate levels between CeD on GFD and non-CeD controls by population

Study or Subgroup	CeD GFD Mean	CeD GFD SD	CeD GFD Total	Controls Mean	Controls SD	Controls Total	Weight	Mean Difference IV, Random, 95% CI
**30.1.2 Children**								
Ballestero-Fernández 2019	15.18	8.06	67	14.12	6.21	66	12.1%	1.06 [-1.38, 3.50]
Bayrak 2020	19.64	13.71	103	18.53	6.09	135	11.9%	1.11 [-1.73, 3.95]
Isikay 2018	28.64	36.43	226	37.38	27.5	268	10.4%	-8.74 [-14.52, -2.96]
Szaflarska-Poplawska 2022	17.95	6.59	48	23.18	9.77	50	11.7%	-5.23 [-8.52, -1.94]
Volkan 2018	18.12	5.1	21	18.51	14.04	30	10.6%	-0.39 [-5.87, 5.09]
**Subtotal (95% CI)**			**465**			**549**	**56.8%**	**-2.03 [-5.47, 1.40]**
Heterogeneity: Tau² = 11.28; Chi² = 18.15, df = 4 (P = 0.001); I² = 78%								
Test for overall effect: Z = 1.16 (P = 0.25)								
**30.1.3 Adults**								
Ballestero-Fernández 2021	17.06	10.07	64	14.74	6.75	74	11.9%	2.32 [-0.59, 5.23]
Dickey 2008	25.7	31.5	41	24.1	14.2	200	7.9%	1.60 [-8.24, 11.44]
Mansueto 2023	11.8	6.14	174	23.64	8.86	124	12.3%	-11.84 [-13.65, -10.03]
Valente 2015	17.5	8	20	29	9.4	39	11.1%	-11.50 [-16.08, -6.92]
**Subtotal (95% CI)**			**299**			**437**	**43.2%**	**-5.23 [-13.87, 3.40]**
Heterogeneity: Tau² = 70.30; Chi² = 71.65, df = 3 (P < 0.00001); I² = 96%								
Test for overall effect: Z = 1.19 (P = 0.24)								
**Total (95% CI)**			**764**			**986**	**100.0%**	**-3.63 [-8.21, 0.94]**
Heterogeneity: Tau² = 43.61; Chi² = 134.20, df = 8 (P < 0.00001); I² = 94%								
Test for overall effect: Z = 1.56 (P = 0.12)								
Test for subgroup differences: Chi² = 0.45, df = 1 (P = 0.50), I² = 0%								

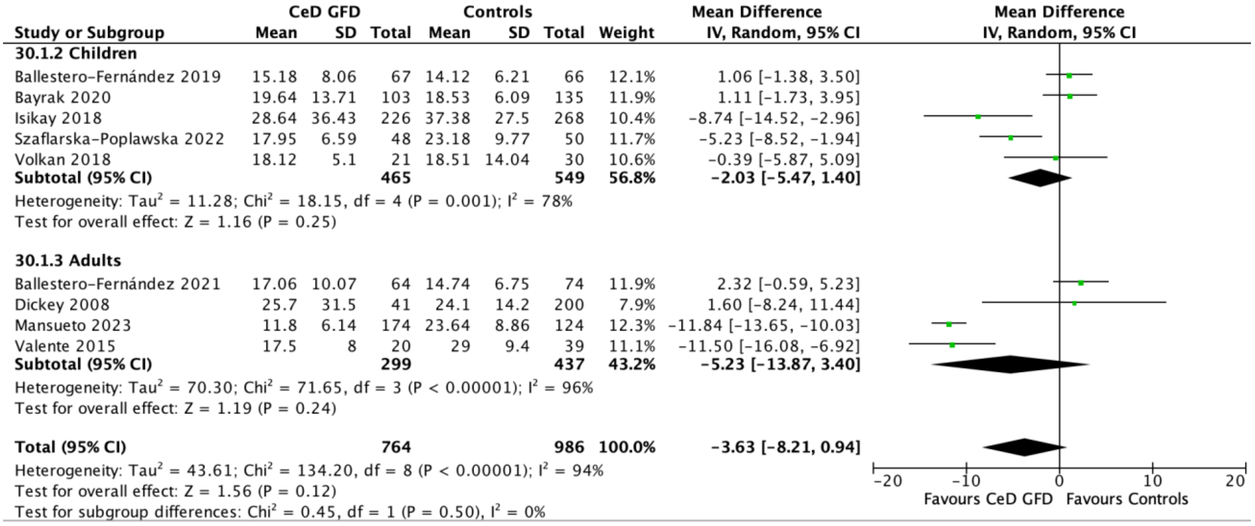

Supplementary Figure S13. Iron studies- Ferritin

S13A- Forest plot comparison of low serum ferritin levels between CeD on GFD and non-CeD controls

Study or Subgroup	CeD GFD Events	CeD GFD Total	Controls Events	Controls Total	Weight	Odds Ratio M-H, Random, 95% CI
Bayrak 2020	56	103	62	135	50.3%	1.40 [0.84, 2.35]
Mansueto 2023	106	174	16	124	49.7%	10.52 [5.73, 19.31]
**Total (95% CI)**		**277**		**259**	**100.0%**	**3.82 [0.53, 27.63]**
Total events	162		78			
Heterogeneity: Tau² = 1.96; Chi² = 24.75, df = 1 (P < 0.00001); I² = 96%						
Test for overall effect: Z = 1.33 (P = 0.18)						

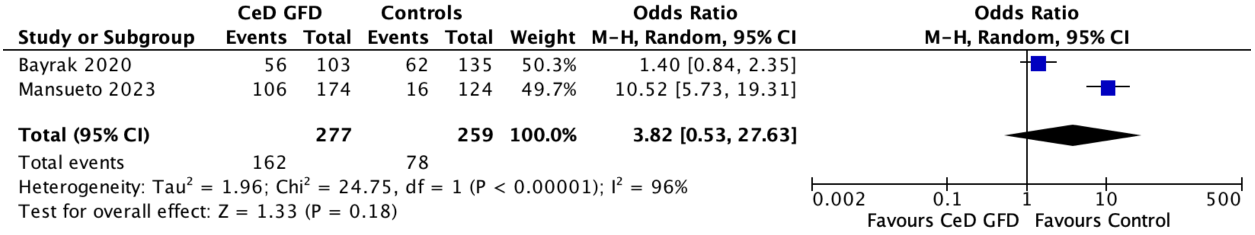

S13B- Forest plot comparison of means of serum Ferritin levels between CeD on GFD and CeD not on GFD

Study or Subgroup	CeD GFD Mean	CeD GFD SD	CeD GFD Total	CeD non GFD Mean	CeD non GFD SD	CeD non GFD Total	Weight	Mean Difference IV, Random, 95% CI
Kemppainen 1995	70	70	51	55	135	37	1.1%	15.00 [-32.55, 62.55]
McGrogan 2021	27.5	13.8	43	15.5	7.86	24	88.3%	12.00 [6.81, 17.19]
Piatek-Guziewicz 2017, May	59.5	81.9	92	52.3	163.2	53	1.1%	7.20 [-39.82, 54.22]
Volkan 2018	39.1	20.5	21	39	34	26	9.6%	0.10 [-15.64, 15.84]
**Total (95% CI)**			**207**			**140**	**100.0%**	**10.84 [5.97, 15.71]**
Heterogeneity: Tau² = 0.00; Chi² = 2.03, df = 3 (P = 0.57); I² = 0%								
Test for overall effect: Z = 4.36 (P < 0.0001)								

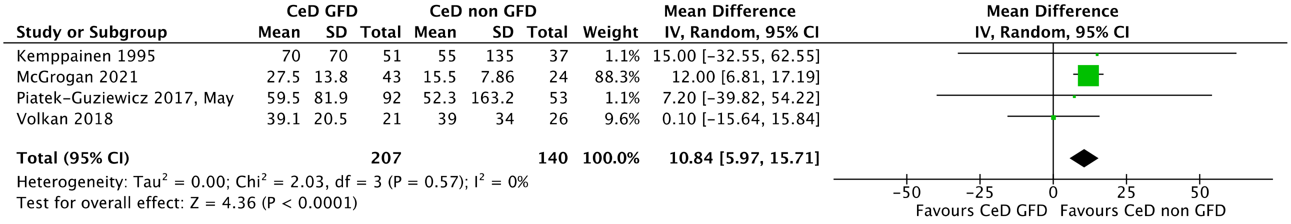

S13C- Forest plot comparison of means of serum ferritin levels between CeD on GFD and non-CeD controls

Study or Subgroup	CeD GFD Mean	SD	Total	Controls Mean	SD	Total	Weight	Mean Difference IV, Random, 95% CI
Bayrak 2020	23.1	20.8	103	28.23	16.76	135	14.6%	−5.13 [−10.04, −0.22]
Efe 2023	25.66	5.72	50	28	3.43	72	14.9%	−2.34 [−4.11, −0.57]
El Amrousy 2024	15.1	4.3	40	80.5	18.2	40	14.4%	−65.40 [−71.20, −59.60]
Elli 2015	63.2	102.2	38	74.1	51.4	76	6.7%	−10.90 [−45.39, 23.59]
Isikay 2018	16.48	12.57	226	24.11	5.78	268	14.9%	−7.63 [−9.41, −5.85]
Mansueto 2023	78.3	70.5	174	92.1	70.8	124	11.7%	−13.80 [−30.08, 2.48]
Piatek-Guziewicz 2017, May	59.5	81.9	92	53.9	24.5	52	11.2%	5.60 [−12.41, 23.61]
Volkan 2018	39.1	20.5	21	39.6	38.4	30	11.7%	−0.50 [−16.80, 15.80]
**Total (95% CI)**			**744**			**797**	**100.0%**	**−13.45 [−25.39, −1.50]**

Heterogeneity: $Tau^2 = 248.61$; $Chi^2 = 420.25$, df = 7 ($P < 0.00001$); $I^2 = 98\%$
Test for overall effect: Z = 2.21 (P = 0.03)

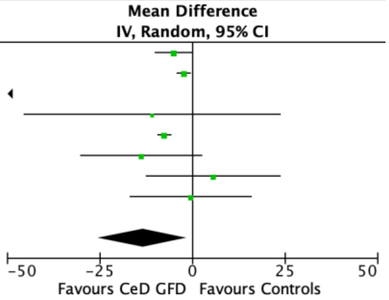

S13D- Forest plot comparison of means of serum ferritin levels between CeD on GFD and non-CeD controls by study design

Study or Subgroup	CeD GFD Mean	SD	Total	Controls Mean	SD	Total	Weight	Mean Difference IV, Random, 95% CI
**16.1.2 Cross-sectional**								
Bayrak 2020	23.1	20.8	103	28.23	16.76	135	14.6%	−5.13 [−10.04, −0.22]
El Amrousy 2024	15.1	4.3	40	80.5	18.2	40	14.4%	−65.40 [−71.20, −59.60]
Isikay 2018	16.48	12.57	226	24.11	5.78	268	14.9%	−7.63 [−9.41, −5.85]
**Subtotal (95% CI)**			**369**			**443**	**43.9%**	**−25.97 [−57.13, 5.20]**
Heterogeneity: $Tau^2 = 753.24$; $Chi^2 = 356.78$, df = 2 ($P < 0.00001$); $I^2 = 99\%$								
Test for overall effect: Z = 1.63 (P = 0.10)								
**16.1.3 Case-Control**								
Efe 2023	25.66	5.72	50	28	3.43	72	14.9%	−2.34 [−4.11, −0.57]
Elli 2015	63.2	102.2	38	74.1	51.4	76	6.7%	−10.90 [−45.39, 23.59]
Mansueto 2023	78.3	70.5	174	92.1	70.8	124	11.7%	−13.80 [−30.08, 2.48]
Piatek-Guziewicz 2017, May	59.5	81.9	92	53.9	24.5	52	11.2%	5.60 [−12.41, 23.61]
Volkan 2018	39.1	20.5	21	39.6	38.4	30	11.7%	−0.50 [−16.80, 15.80]
**Subtotal (95% CI)**			**375**			**354**	**56.1%**	**−2.40 [−4.14, −0.66]**
Heterogeneity: $Tau^2 = 0.00$; $Chi^2 = 2.93$, df = 4 ($P = 0.57$); $I^2 = 0\%$								
Test for overall effect: Z = 2.70 (P = 0.007)								
**Total (95% CI)**			**744**			**797**	**100.0%**	**−13.45 [−25.39, −1.50]**

Heterogeneity: $Tau^2 = 248.61$; $Chi^2 = 420.25$, df = 7 ($P < 0.00001$); $I^2 = 98\%$
Test for overall effect: Z = 2.21 (P = 0.03)
Test for subgroup differences: $Chi^2 = 2.19$, df = 1 (P = 0.14), $I^2 = 54.3\%$

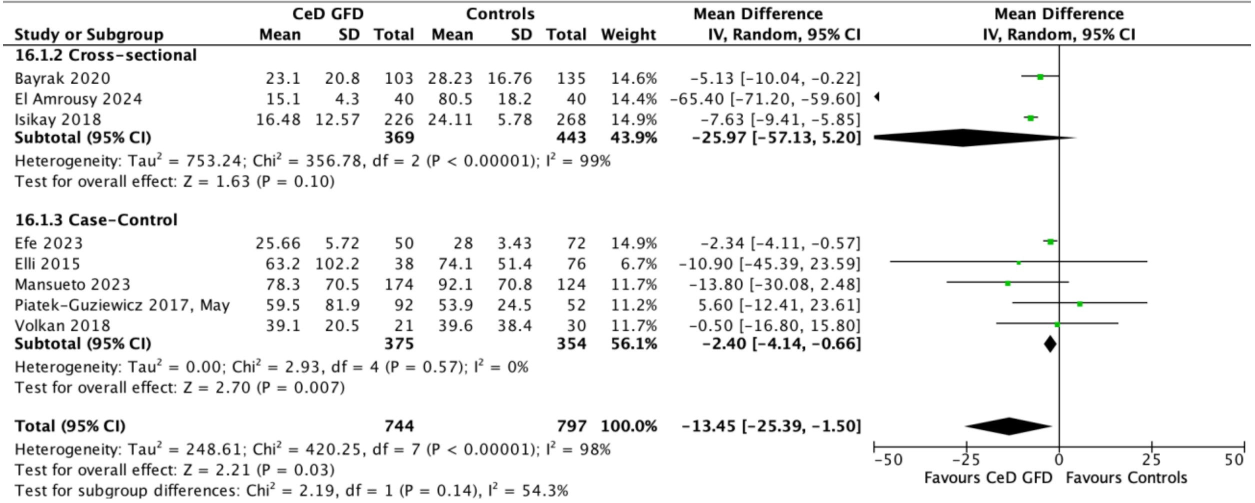

S13E- Forest plot comparison of means of serum ferritin levels between CeD on GFD and non-CeD controls by GFD length

Study or Subgroup	CeD GFD Mean	SD	Total	Controls Mean	SD	Total	Weight	Mean Difference IV, Random, 95% CI
**51.1.1 Short-Term GFD**								
Efe 2023	25.66	5.72	50	28	3.43	72	14.9%	−2.34 [−4.11, −0.57]
El Amrousy 2024	15.1	4.3	40	80.5	18.2	40	14.4%	−65.40 [−71.20, −59.60]
Isikay 2018	16.48	12.57	226	24.11	5.78	268	14.9%	−7.63 [−9.41, −5.85]
Mansueto 2023	78.3	70.5	174	92.1	70.8	124	11.7%	−13.80 [−30.08, 2.48]
Volkan 2018	39.1	20.5	21	39.6	38.4	30	11.7%	−0.50 [−16.80, 15.80]
**Subtotal (95% CI)**			**511**			**534**	**67.6%**	**−18.55 [−34.15, −2.95]**
Heterogeneity: $Tau^2 = 290.15$; $Chi^2 = 417.21$, df = 4 ($P < 0.00001$); $I^2 = 99\%$								
Test for overall effect: Z = 2.33 (P = 0.02)								
**51.1.2 Long-Term GFD**								
Bayrak 2020	23.1	20.8	103	28.23	16.76	135	14.6%	−5.13 [−10.04, −0.22]
Elli 2015	63.2	102.2	38	74.1	51.4	76	6.7%	−10.90 [−45.39, 23.59]
Piatek-Guziewicz 2017, May	59.5	81.9	92	53.9	24.5	52	11.2%	5.60 [−12.41, 23.61]
**Subtotal (95% CI)**			**233**			**263**	**32.4%**	**−4.51 [−9.20, 0.19]**
Heterogeneity: $Tau^2 = 0.00$; $Chi^2 = 1.40$, df = 2 ($P = 0.50$); $I^2 = 0\%$								
Test for overall effect: Z = 1.88 (P = 0.06)								
**Total (95% CI)**			**744**			**797**	**100.0%**	**−13.45 [−25.39, −1.50]**

Heterogeneity: $Tau^2 = 248.61$; $Chi^2 = 420.25$, df = 7 ($P < 0.00001$); $I^2 = 98\%$
Test for overall effect: Z = 2.21 (P = 0.03)
Test for subgroup differences: $Chi^2 = 2.86$, df = 1 (P = 0.09), $I^2 = 65.0\%$

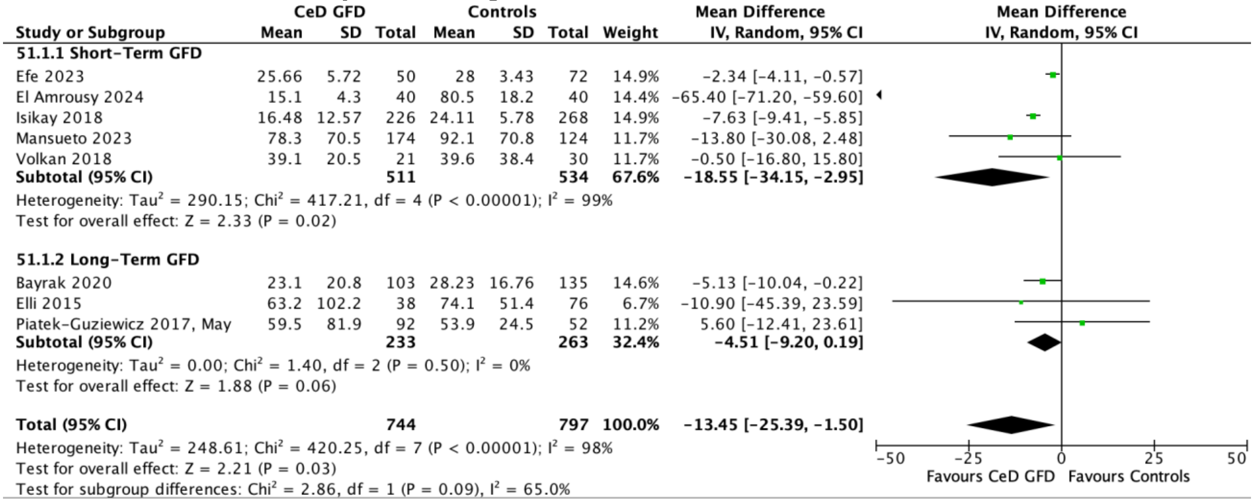

S13F- Forest plot comparison of means of serum ferritin levels between CeD on GFD and non-CeD controls by population

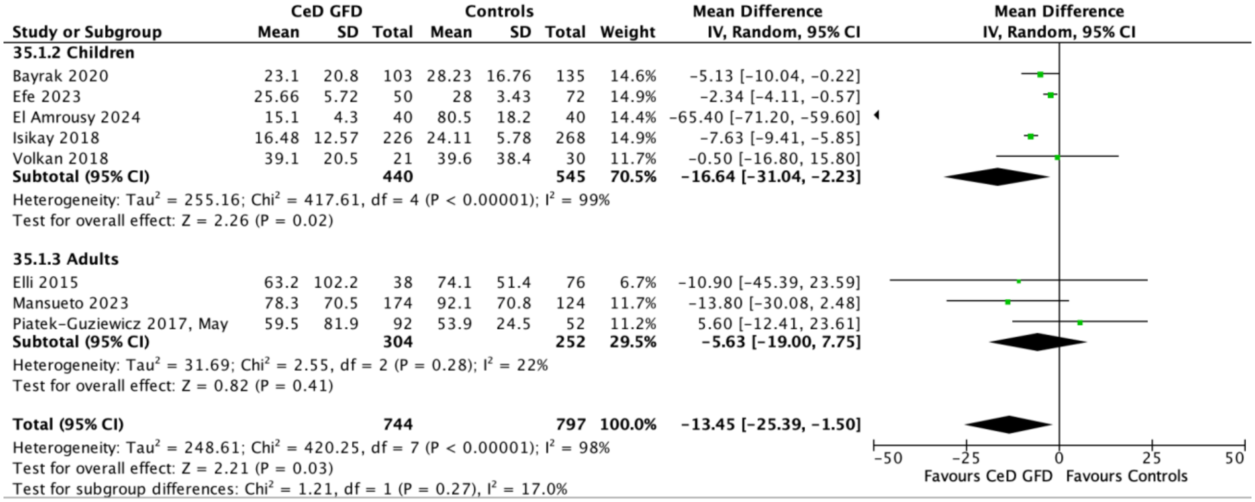

Supplementary Figure S14. Iron

S14A- Forest plot comparison of serum iron deficiency between CeD on GFD and non-CeD controls

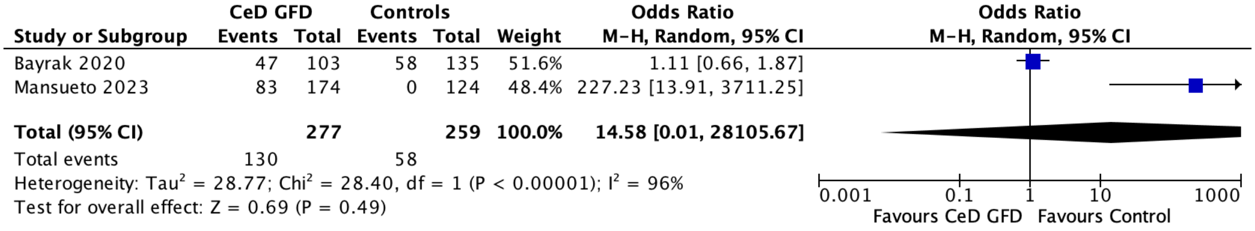

S14B- Forest plot comparison of means of serum iron levels between CeD on GFD and CeD not on GFD

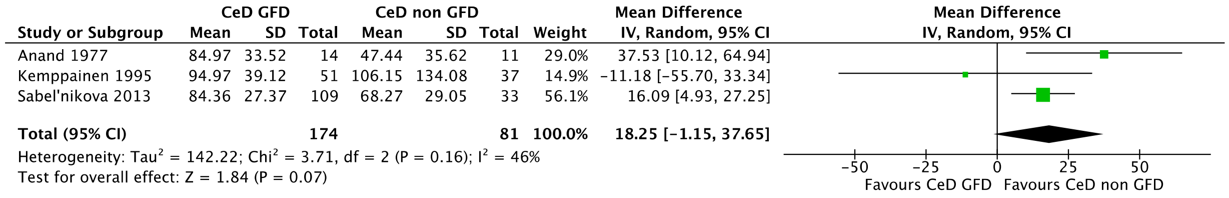

S14C- Forest plot comparison of means of serum iron levels between CeD on GFD and CeD not on GFD by study design

Study or Subgroup	CeD GFD Mean	SD	Total	CeD non GFD Mean	SD	Total	Weight	Mean Difference IV, Random, 95% CI
**2.1.1 Cohort**								
Kemppainen 1995	94.97	39.12	51	106.15	134.08	37	14.9%	−11.18 [−55.70, 33.34]
Sabel'nikova 2013	84.36	27.37	109	68.27	29.05	33	56.1%	16.09 [4.93, 27.25]
**Subtotal (95% CI)**			**160**			**70**	**71.0%**	**11.32 [−8.99, 31.63]**
Heterogeneity: Tau² = 97.66; Chi² = 1.36, df = 1 (P = 0.24); I² = 26%								
Test for overall effect: Z = 1.09 (P = 0.27)								
**2.1.3 Case-Control**								
Anand 1977	84.97	33.52	14	47.44	35.62	11	29.0%	37.53 [10.12, 64.94]
**Subtotal (95% CI)**			**14**			**11**	**29.0%**	**37.53 [10.12, 64.94]**
Heterogeneity: Not applicable								
Test for overall effect: Z = 2.68 (P = 0.007)								
**Total (95% CI)**			**174**			**81**	**100.0%**	**18.25 [−1.15, 37.65]**
Heterogeneity: Tau² = 142.22; Chi² = 3.71, df = 2 (P = 0.16); I² = 46%								
Test for overall effect: Z = 1.84 (P = 0.07)								
Test for subgroup differences: Chi² = 2.27, df = 1 (P = 0.13), I² = 55.9%								

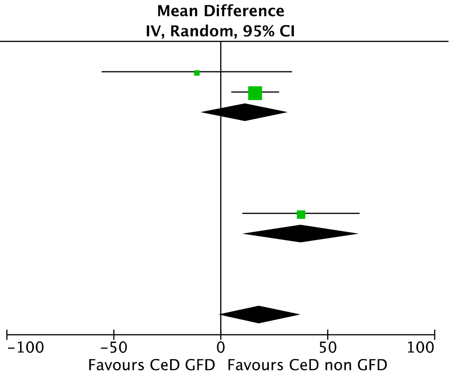

S14D- Forest plot comparison of means of serum iron levels between CeD on GFD and CeD not on GFD by GFD length

Study or Subgroup	CeD GFD Mean	SD	Total	CeD non GFD Mean	SD	Total	Weight	Mean Difference IV, Random, 95% CI
**78.1.1 Short-Term GFD**								
Anand 1977	84.97	33.52	14	47.44	35.62	11	29.0%	37.53 [10.12, 64.94]
Kemppainen 1995	94.97	39.12	51	106.15	134.08	37	14.9%	−11.18 [−55.70, 33.34]
**Subtotal (95% CI)**			**65**			**48**	**43.9%**	**16.46 [−30.84, 63.76]**
Heterogeneity: Tau² = 830.59; Chi² = 3.33, df = 1 (P = 0.07); I² = 70%								
Test for overall effect: Z = 0.68 (P = 0.50)								
**78.1.2 Unknown GFD**								
Sabel'nikova 2013	84.36	27.37	109	68.27	29.05	33	56.1%	16.09 [4.93, 27.25]
**Subtotal (95% CI)**			**109**			**33**	**56.1%**	**16.09 [4.93, 27.25]**
Heterogeneity: Not applicable								
Test for overall effect: Z = 2.82 (P = 0.005)								
**Total (95% CI)**			**174**			**81**	**100.0%**	**18.25 [−1.15, 37.65]**
Heterogeneity: Tau² = 142.22; Chi² = 3.71, df = 2 (P = 0.16); I² = 46%								
Test for overall effect: Z = 1.84 (P = 0.07)								
Test for subgroup differences: Chi² = 0.00, df = 1 (P = 0.99), I² = 0%								

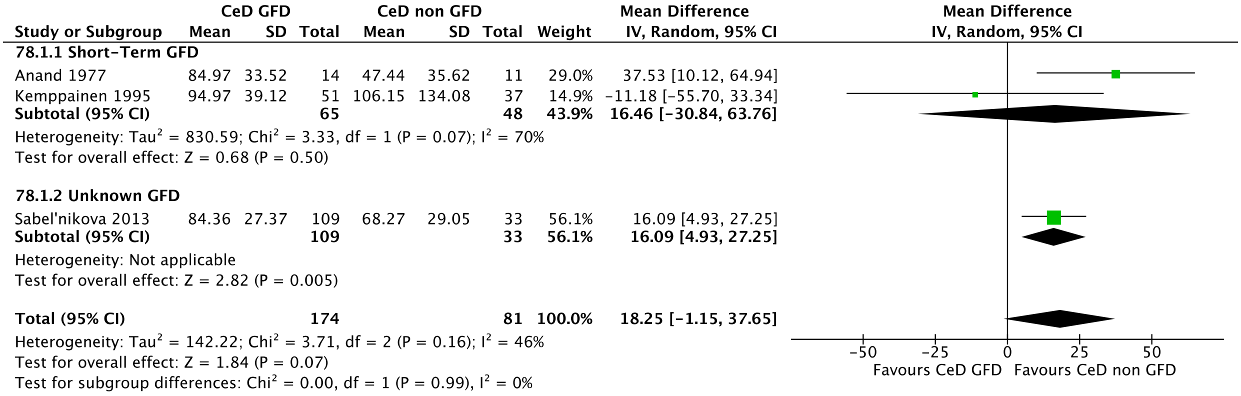

S14E- Forest plot comparison of means of serum iron levels between CeD on GFD and non-CeD controls

Study or Subgroup	CeD GFD Mean	SD	Total	Controls Mean	SD	Total	Weight	Mean Difference IV, Random, 95% CI
Ballestero-Fernández 2019	98.67	35.56	67	102.83	29.63	66	15.7%	−4.16 [−15.28, 6.96]
Ballestero-Fernández 2021	101.13	30	64	108.07	35.04	74	15.8%	−6.94 [−17.79, 3.91]
Bayrak 2020	68.6	38.5	103	71.39	37.7	135	16.4%	−2.79 [−12.57, 6.99]
Efe 2023	72.15	3.6	50	75.5	4.3	72	19.8%	−3.35 [−4.76, −1.94]
Elli 2015	57.6	33.9	38	103.4	28.1	76	14.8%	−45.80 [−58.29, −33.31]
Mansueto 2023	71.5	34.8	174	83.3	34.3	124	17.5%	−11.80 [−19.75, −3.85]
**Total (95% CI)**			**496**			**547**	**100.0%**	**−11.72 [−21.28, −2.17]**
Heterogeneity: Tau² = 119.64; Chi² = 47.82, df = 5 (P < 0.00001); I² = 90%								
Test for overall effect: Z = 2.40 (P = 0.02)								

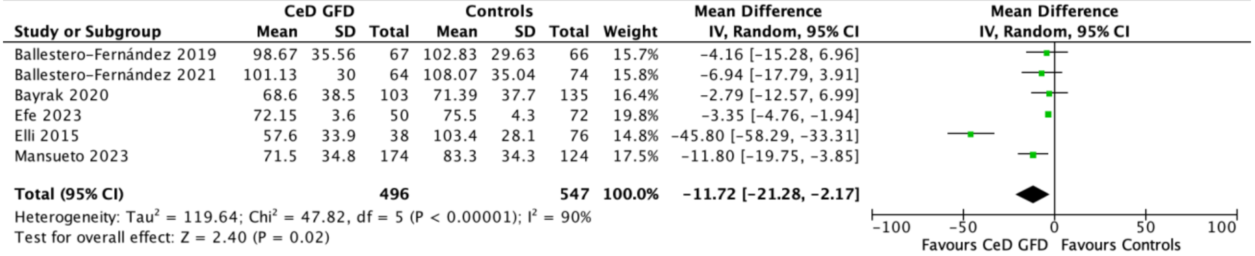

S14F- Forest plot comparison of means of serum iron levels between CeD on GFD and non-CeD controls by study design

Study or Subgroup	CeD GFD Mean	CeD GFD SD	CeD GFD Total	Controls Mean	Controls SD	Controls Total	Weight	Mean Difference IV, Random, 95% CI
**1.1.1 Cross-sectional**								
Ballestero-Fernández 2019	98.67	35.56	67	102.83	29.63	66	15.7%	-4.16 [-15.28, 6.96]
Ballestero-Fernández 2021	101.13	30	64	108.07	35.04	74	15.8%	-6.94 [-17.79, 3.91]
Bayrak 2020	68.6	38.5	103	71.39	37.7	135	16.4%	-2.79 [-12.57, 6.99]
**Subtotal (95% CI)**			**234**			**275**	**47.9%**	**-4.50 [-10.59, 1.58]**
Heterogeneity: $Tau^2 = 0.00$; $Chi^2 = 0.32$, df = 2 (P = 0.85); $I^2 = 0\%$								
Test for overall effect: Z = 1.45 (P = 0.15)								
**1.1.2 Case-Control**								
Efe 2023	72.15	3.6	50	75.5	4.3	72	19.8%	-3.35 [-4.76, -1.94]
Elli 2015	57.6	33.9	38	103.4	28.1	76	14.8%	-45.80 [-58.29, -33.31]
Mansueto 2023	71.5	34.8	174	83.3	34.3	124	17.5%	-11.80 [-19.75, -3.85]
**Subtotal (95% CI)**			**262**			**272**	**52.1%**	**-19.33 [-38.73, 0.06]**
Heterogeneity: $Tau^2 = 275.55$; $Chi^2 = 47.49$, df = 2 (P < 0.00001); $I^2 = 96\%$								
Test for overall effect: Z = 1.95 (P = 0.05)								
**Total (95% CI)**			**496**			**547**	**100.0%**	**-11.72 [-21.28, -2.17]**
Heterogeneity: $Tau^2 = 119.64$; $Chi^2 = 47.82$, df = 5 (P < 0.00001); $I^2 = 90\%$								
Test for overall effect: Z = 2.40 (P = 0.02)								
Test for subgroup differences: $Chi^2 = 2.04$, df = 1 (P = 0.15), $I^2 = 51.1\%$								

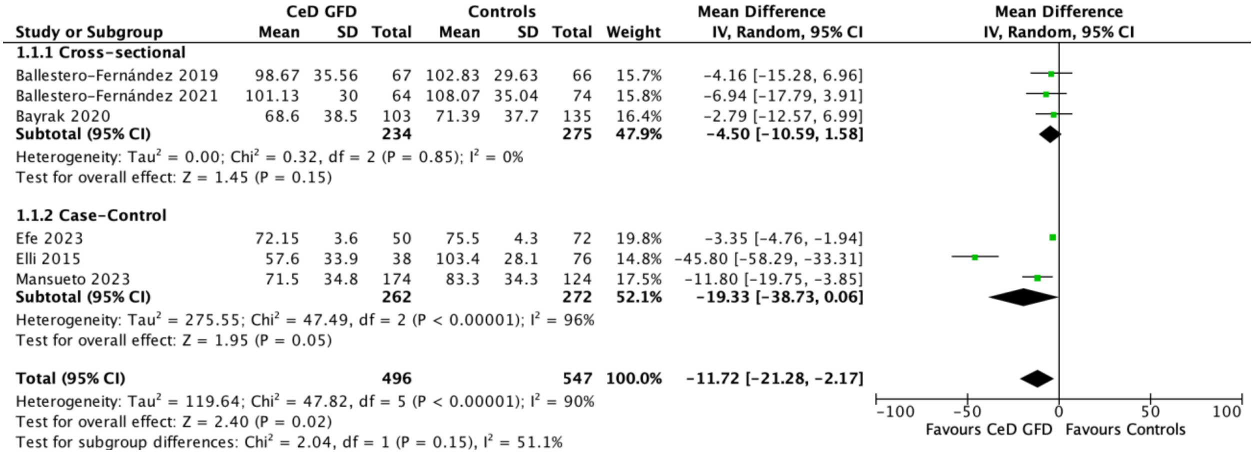

S14G- Forest plot comparison of means of serum iron levels between CeD on GFD and non-CeD controls by GFD Length

Study or Subgroup	CeD GFD Mean	CeD GFD SD	CeD GFD Total	Controls Mean	Controls SD	Controls Total	Weight	Mean Difference IV, Random, 95% CI
**53.1.1 Short-Term GFD**								
Ballestero-Fernández 2019	98.67	35.56	67	102.83	29.63	66	15.7%	-4.16 [-15.28, 6.96]
Ballestero-Fernández 2021	101.13	30	64	108.07	35.04	74	15.8%	-6.94 [-17.79, 3.91]
Efe 2023	72.15	3.6	50	75.5	4.3	72	19.8%	-3.35 [-4.76, -1.94]
Mansueto 2023	71.5	34.8	174	83.3	34.3	124	17.5%	-11.80 [-19.75, -3.85]
**Subtotal (95% CI)**			**355**			**336**	**68.7%**	**-5.39 [-9.42, -1.37]**
Heterogeneity: $Tau^2 = 6.59$; $Chi^2 = 4.57$, df = 3 (P = 0.21); $I^2 = 34\%$								
Test for overall effect: Z = 2.62 (P = 0.009)								
**53.1.2 Long-Term GFD**								
Bayrak 2020	68.6	38.5	103	71.39	37.7	135	16.4%	-2.79 [-12.57, 6.99]
Elli 2015	57.6	33.9	38	103.4	28.1	76	14.8%	-45.80 [-58.29, -33.31]
**Subtotal (95% CI)**			**141**			**211**	**31.3%**	**-24.11 [-66.26, 18.04]**
Heterogeneity: $Tau^2 = 892.15$; $Chi^2 = 28.22$, df = 1 (P < 0.00001); $I^2 = 96\%$								
Test for overall effect: Z = 1.12 (P = 0.26)								
**Total (95% CI)**			**496**			**547**	**100.0%**	**-11.72 [-21.28, -2.17]**
Heterogeneity: $Tau^2 = 119.64$; $Chi^2 = 47.82$, df = 5 (P < 0.00001); $I^2 = 90\%$								
Test for overall effect: Z = 2.40 (P = 0.02)								
Test for subgroup differences: $Chi^2 = 0.75$, df = 1 (P = 0.39), $I^2 = 0\%$								

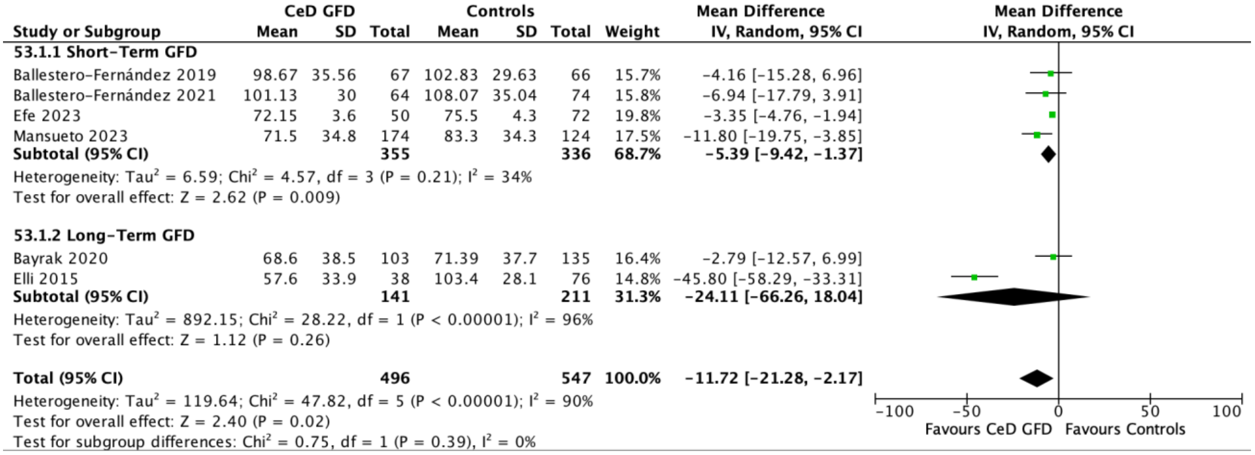

S14H- Forest plot comparison of means of serum iron levels between CeD on GFD and non-CeD controls by population

Study or Subgroup	CeD GFD Mean	CeD GFD SD	CeD GFD Total	Controls Mean	Controls SD	Controls Total	Weight	Mean Difference IV, Random, 95% CI
**33.1.1 Children**								
Ballestero-Fernández 2019	98.67	35.56	70	102.83	29.63	67	15.5%	-4.16 [-15.10, 6.78]
Bayrak 2020	68.6	38.5	103	71.39	37.7	135	16.2%	-2.79 [-12.57, 6.99]
Efe 2023	72.15	3.6	50	75.5	4.3	72	20.0%	-3.35 [-4.76, -1.94]
**Subtotal (95% CI)**			**223**			**274**	**51.6%**	**-3.35 [-4.73, -1.97]**
Heterogeneity: $Tau^2 = 0.00$; $Chi^2 = 0.03$, df = 2 (P = 0.98); $I^2 = 0\%$								
Test for overall effect: Z = 4.75 (P < 0.00001)								
**33.1.2 Adults**								
Ballestero-Fernández 2021	101.13	30	64	108.07	35.04	74	15.5%	-6.94 [-17.79, 3.91]
Elli 2015	57.6	33.9	38	103.4	28.1	76	14.4%	-45.80 [-58.29, -33.31]
Mansueto 2023	71.5	4.3	174	83.3	34.3	124	18.4%	-11.80 [-17.87, -5.73]
**Subtotal (95% CI)**			**276**			**274**	**48.4%**	**-21.05 [-41.20, -0.90]**
Heterogeneity: $Tau^2 = 290.71$; $Chi^2 = 26.37$, df = 2 (P < 0.00001); $I^2 = 92\%$								
Test for overall effect: Z = 2.05 (P = 0.04)								
**Total (95% CI)**			**499**			**548**	**100.0%**	**-11.63 [-20.59, -2.67]**
Heterogeneity: $Tau^2 = 104.13$; $Chi^2 = 50.32$, df = 5 (P < 0.00001); $I^2 = 90\%$								
Test for overall effect: Z = 2.54 (P = 0.01)								
Test for subgroup differences: $Chi^2 = 2.95$, df = 1 (P = 0.09), $I^2 = 66.1\%$								

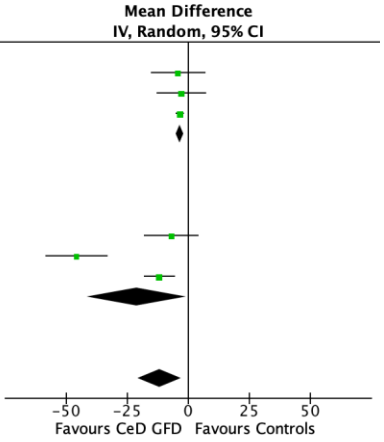

Supplementary Figure 15. Magnesium

S15A- Forest plot comparison of Magnesium deficiency between CeD on GFD and CeD not on GFD

	CeD GFD		CeD non GFD			Odds Ratio
Study or Subgroup	Events	Total	Events	Total	Weight	M-H, Random, 95% CI
McGrogan 2021	1	41	1	24	54.7%	0.57 [0.03, 9.64]
Selbuz 2021	0	18	2	26	45.3%	0.26 [0.01, 5.85]
**Total (95% CI)**		**59**		**50**	**100.0%**	**0.40 [0.05, 3.25]**
Total events	1		3			

Heterogeneity: $Tau^2 = 0.00$; $Chi^2 = 0.13$, df = 1 (P = 0.71); $I^2 = 0\%$
Test for overall effect: Z = 0.85 (P = 0.39)

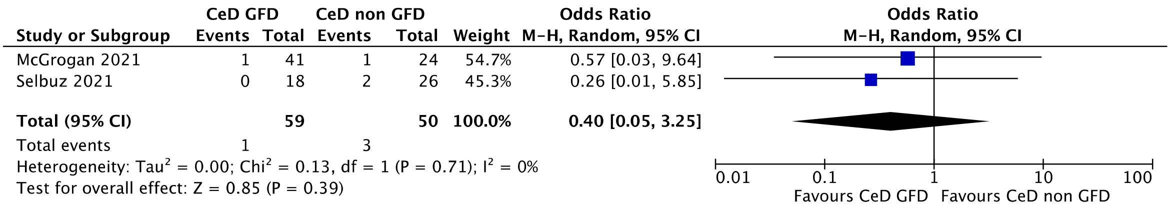

S15B- Forest plot comparison of means of serum Magnesium levels between CeD on GFD and CeD not on GFD

	CeD GFD			CeD non GFD				Mean Difference
Study or Subgroup	Mean	SD	Total	Mean	SD	Total	Weight	IV, Random, 95% CI
Kemppainen 1995	0.8	0.1	51	0.8	0.1	37	28.7%	0.00 [−0.04, 0.04]
McGrogan 2021	0.88	0.06	41	0.82	0.07	24	32.8%	0.06 [0.03, 0.09]
Volkan 2018	0.78	0.04	21	0.78	0.03	26	38.5%	0.00 [−0.02, 0.02]
**Total (95% CI)**			**113**			**87**	**100.0%**	**0.02 [−0.02, 0.06]**

Heterogeneity: $Tau^2 = 0.00$; $Chi^2 = 9.44$, df = 2 (P = 0.009); $I^2 = 79\%$
Test for overall effect: Z = 0.98 (P = 0.33)

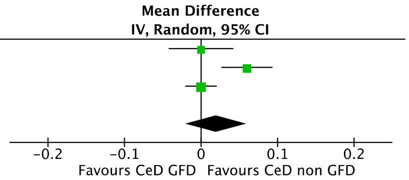

S15C- Forest plot comparison of means of serum Magnesium levels between CeD on GFD and CeD not on GFD by study design

	CeD GFD			CeD non GFD				Mean Difference
Study or Subgroup	Mean	SD	Total	Mean	SD	Total	Weight	IV, Random, 95% CI
**34.1.1 Cohort**								
Kemppainen 1995	0.8	0.1	51	0.8	0.1	37	28.7%	0.00 [−0.04, 0.04]
McGrogan 2021	0.88	0.06	41	0.82	0.07	24	32.8%	0.06 [0.03, 0.09]
**Subtotal (95% CI)**			**92**			**61**	**61.5%**	**0.03 [−0.03, 0.09]**
Heterogeneity: $Tau^2 = 0.00$; $Chi^2 = 4.75$, df = 1 (P = 0.03); $I^2 = 79\%$								
Test for overall effect: Z = 1.05 (P = 0.29)								
**34.1.2 Case-Control**								
Volkan 2018	0.78	0.04	21	0.78	0.03	26	38.5%	0.00 [−0.02, 0.02]
**Subtotal (95% CI)**			**21**			**26**	**38.5%**	**0.00 [−0.02, 0.02]**
Heterogeneity: Not applicable								
Test for overall effect: Z = 0.00 (P = 1.00)								
**Total (95% CI)**			**113**			**87**	**100.0%**	**0.02 [−0.02, 0.06]**

Heterogeneity: $Tau^2 = 0.00$; $Chi^2 = 9.44$, df = 2 (P = 0.009); $I^2 = 79\%$
Test for overall effect: Z = 0.98 (P = 0.33)
Test for subgroup differences: $Chi^2 = 0.98$, df = 1 (P = 0.32), $I^2 = 0\%$

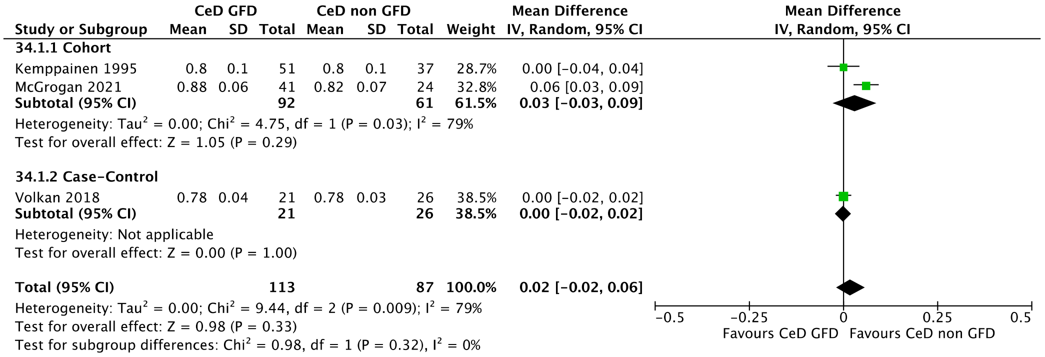

S15D- Forest plot comparison of means of serum Magnesium levels between CeD on GFD and CeD not on GFD by population

	CeD GFD			CeD non GFD				Mean Difference
Study or Subgroup	Mean	SD	Total	Mean	SD	Total	Weight	IV, Random, 95% CI
**50.1.1 Children**								
McGrogan 2021	0.88	0.06	41	0.82	0.07	24	32.8%	0.06 [0.03, 0.09]
Volkan 2018	0.78	0.04	21	0.78	0.03	26	38.5%	0.00 [−0.02, 0.02]
**Subtotal (95% CI)**			**62**			**50**	**71.3%**	**0.03 [−0.03, 0.09]**
Heterogeneity: $Tau^2 = 0.00$; $Chi^2 = 8.94$, df = 1 (P = 0.003); $I^2 = 89\%$								
Test for overall effect: Z = 0.95 (P = 0.34)								
**50.1.2 Adults**								
Kemppainen 1995	0.8	0.1	51	0.8	0.1	37	28.7%	0.00 [−0.04, 0.04]
**Subtotal (95% CI)**			**51**			**37**	**28.7%**	**0.00 [−0.04, 0.04]**
Heterogeneity: Not applicable								
Test for overall effect: Z = 0.00 (P = 1.00)								
**Total (95% CI)**			**113**			**87**	**100.0%**	**0.02 [−0.02, 0.06]**

Heterogeneity: $Tau^2 = 0.00$; $Chi^2 = 9.44$, df = 2 (P = 0.009); $I^2 = 79\%$
Test for overall effect: Z = 0.98 (P = 0.33)
Test for subgroup differences: $Chi^2 = 0.60$, df = 1 (P = 0.44), $I^2 = 0\%$

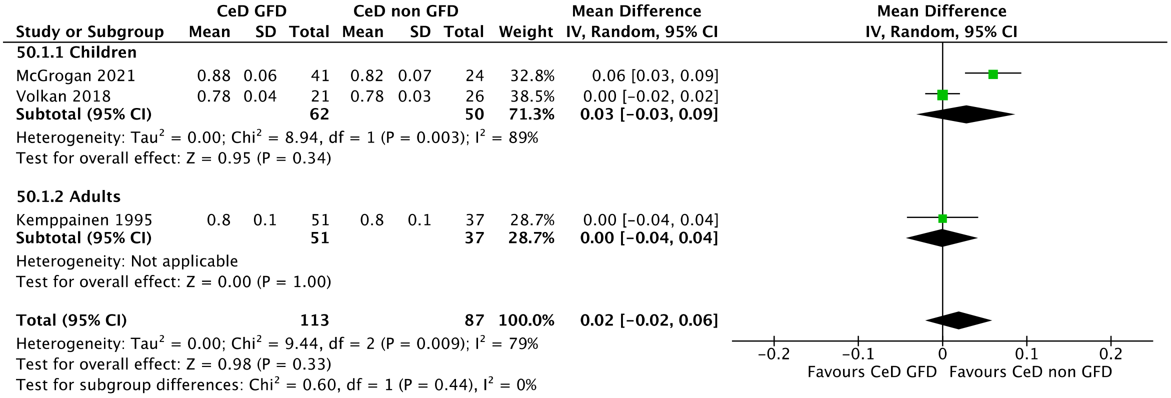

S15E- Forest plot comparison of means of serum Magnesium levels between CeD on GFD and non-CeD controls

Study or Subgroup	CeD GFD Mean	CeD GFD SD	CeD GFD Total	Controls Mean	Controls SD	Controls Total	Weight	Mean Difference IV, Random, 95% CI
Szaflarska-Poplawska 2022	0.9	0.1	48	0.8	0.1	50	51.1%	0.10 [0.06, 0.14]
Volkan 2018	1.9	0.1	21	2.05	0.2	30	48.9%	-0.15 [-0.23, -0.07]
**Total (95% CI)**			**69**			**80**	**100.0%**	**-0.02 [-0.27, 0.22]**

Heterogeneity: $Tau^2 = 0.03$; $Chi^2 = 28.18$, df = 1 ($P < 0.00001$); $I^2 = 96\%$
Test for overall effect: Z = 0.18 (P = 0.86)

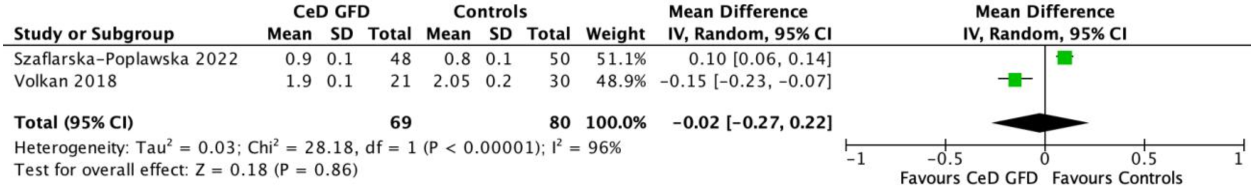

Supplementary Figure S16. Selenium

S16A- Forest plot comparison of Selenium deficiency between CeD on GFD and CeD not on GFD

Study or Subgroup	CeD GFD Events	CeD GFD Total	CeD non GFD Events	CeD non GFD Total	Weight	Odds Ratio M-H, Random, 95% CI
McGrogan 2021	4	41	2	24	100.0%	1.19 [0.20, 7.03]
**Total (95% CI)**		**41**		**24**	**100.0%**	**1.19 [0.20, 7.03]**
Total events	4		2			

Heterogeneity: Not applicable
Test for overall effect: Z = 0.19 (P = 0.85)

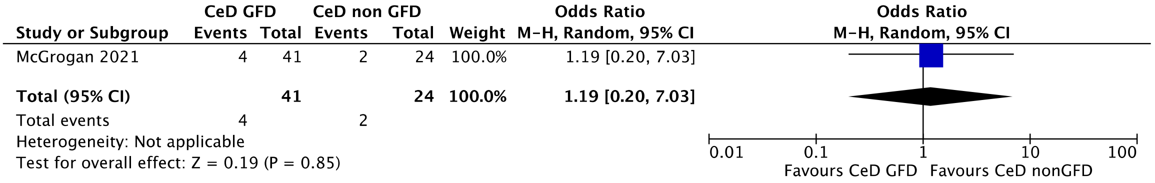

S16B- Forest plot comparison of Selenium deficiency between CeD on GFD and non-CeD controls

Study or Subgroup	CeD GFD Events	CeD GFD Total	Controls Events	Controls Total	Weight	Odds Ratio M-H, Random, 95% CI
Ünal 2012	1	17	3	20	100.0%	0.35 [0.03, 3.77]
**Total (95% CI)**		**17**		**20**	**100.0%**	**0.35 [0.03, 3.77]**
Total events	1		3			

Heterogeneity: Not applicable
Test for overall effect: Z = 0.86 (P = 0.39)

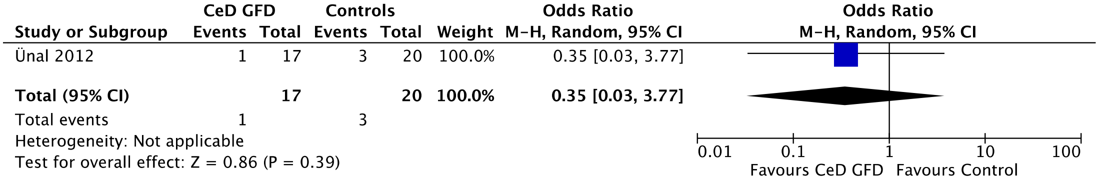

S16C- Forest plot comparison of means of serum Selenium levels between CeD on GFD and CeD not on GFD

Study or Subgroup	CeD GFD Mean	CeD GFD SD	CeD GFD Total	CeD non GFD Mean	CeD non GFD SD	CeD non GFD Total	Weight	Mean Difference IV, Random, 95% CI
Cortigiani 1989	7.11	1.26	36	5.99	0.94	37	33.6%	1.12 [0.61, 1.63]
Kalita 2002	6.43	1.04	31	2.93	0.32	9	33.8%	3.50 [3.08, 3.92]
McGrogan 2021	7.42	1.47	41	7.26	1.66	24	32.6%	0.16 [-0.64, 0.96]
**Total (95% CI)**			**108**			**70**	**100.0%**	**1.61 [-0.39, 3.61]**

Heterogeneity: $Tau^2 = 3.03$; $Chi^2 = 78.53$, df = 2 ($P < 0.00001$); $I^2 = 97\%$
Test for overall effect: Z = 1.58 (P = 0.11)

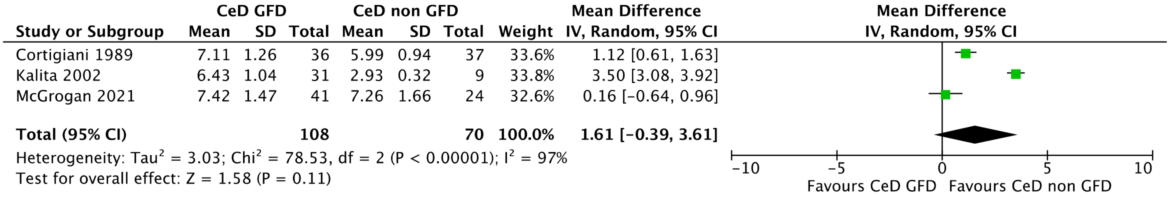

S16D- Forest plot comparison of means of serum Selenium levels between CeD on GFD and CeD not on GFD by study design

Study or Subgroup	CeD GFD Mean	CeD GFD SD	CeD GFD Total	CeD non GFD Mean	CeD non GFD SD	CeD non GFD Total	Weight	Mean Difference IV, Random, 95% CI
**31.1.1 Cohort**								
Kalita 2002	6.43	1.04	31	2.93	0.32	9	33.9%	3.50 [3.08, 3.92]
McGrogan 2021	7.42	1.47	41	7.26	1.66	21	32.4%	0.16 [−0.68, 1.00]
**Subtotal (95% CI)**			**72**			**30**	**66.3%**	**1.85 [−1.42, 5.12]**
Heterogeneity: Tau² = 5.46; Chi² = 48.46, df = 1 (P < 0.00001); I² = 98%								
Test for overall effect: Z = 1.11 (P = 0.27)								
**31.1.2 Case-Control**								
Cortigiani 1989	7.11	1.26	36	5.99	0.94	37	33.7%	1.12 [0.61, 1.63]
**Subtotal (95% CI)**			**36**			**37**	**33.7%**	**1.12 [0.61, 1.63]**
Heterogeneity: Not applicable								
Test for overall effect: Z = 4.30 (P < 0.0001)								
**Total (95% CI)**			**108**			**67**	**100.0%**	**1.62 [−0.38, 3.61]**
Heterogeneity: Tau² = 3.01; Chi² = 76.28, df = 2 (P < 0.00001); I² = 97%								
Test for overall effect: Z = 1.59 (P = 0.11)								
Test for subgroup differences: Chi² = 0.19, df = 1 (P = 0.67), I² = 0%								

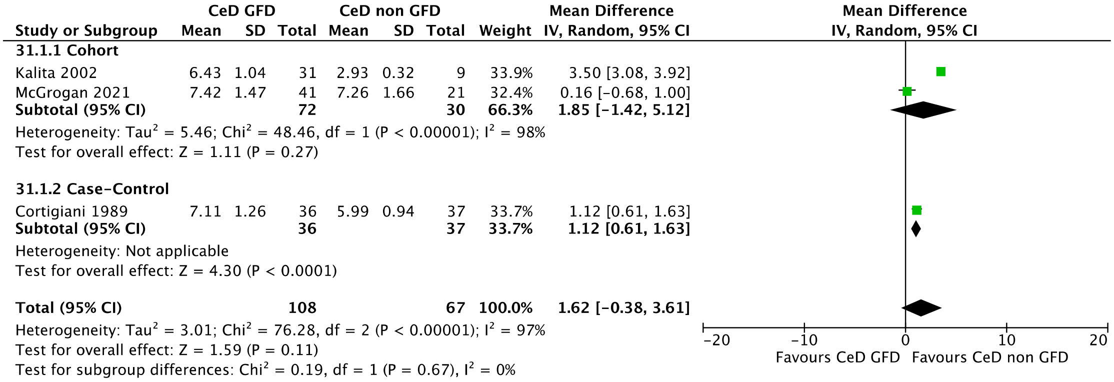

S16E- Forest plot comparison of means of serum Selenium levels between CeD on GFD and non-CeD controls

Study or Subgroup	CeD GFD Mean	CeD GFD SD	CeD GFD Total	Controls Mean	Controls SD	Controls Total	Weight	Mean Difference IV, Random, 95% CI
Cortigiani 1989	7.11	1.26	36	8.34	1.34	51	33.5%	−1.23 [−1.78, −0.68]
Kalita 2002	6.44	1.04	31	7.19	1	27	33.6%	−0.75 [−1.28, −0.22]
Ünal 2012	12.42	1.23	17	9.25	1.21	20	32.9%	3.17 [2.38, 3.96]
**Total (95% CI)**			**84**			**98**	**100.0%**	**0.38 [−1.95, 2.71]**
Heterogeneity: Tau² = 4.13; Chi² = 87.60, df = 2 (P < 0.00001); I² = 98%								
Test for overall effect: Z = 0.32 (P = 0.75)								

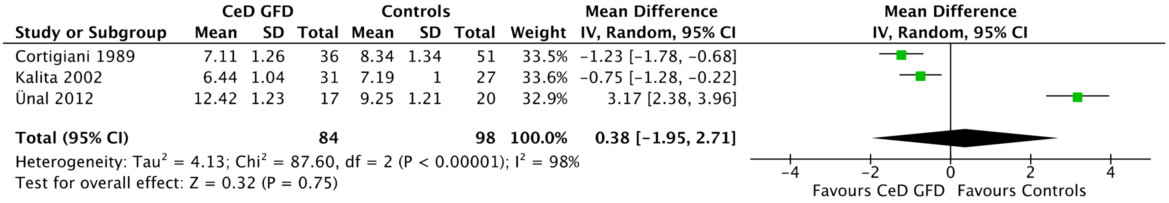

4

S16F- Forest plot comparison of means of serum Selenium levels between CeD on GFD and non-CeD controls by study design

Study or Subgroup	CeD GFD Mean	CeD GFD SD	CeD GFD Total	Controls Mean	Controls SD	Controls Total	Weight	Mean Difference IV, Random, 95% CI
**24.1.1 Cohort**								
Kalita 2002	6.44	1.04	31	7.19	1	27	33.6%	−0.75 [−1.28, −0.22]
**Subtotal (95% CI)**			**31**			**27**	**33.6%**	**−0.75 [−1.28, −0.22]**
Heterogeneity: Not applicable								
Test for overall effect: Z = 2.80 (P = 0.005)								
**24.1.2 Case-Control**								
Cortigiani 1989	7.11	1.26	36	8.34	1.34	51	33.5%	−1.23 [−1.78, −0.68]
Ünal 2012	12.42	1.23	17	9.25	1.21	20	32.9%	3.17 [2.38, 3.96]
**Subtotal (95% CI)**			**53**			**71**	**66.4%**	**0.96 [−3.35, 5.27]**
Heterogeneity: Tau² = 9.56; Chi² = 80.16, df = 1 (P < 0.00001); I² = 99%								
Test for overall effect: Z = 0.44 (P = 0.66)								
**Total (95% CI)**			**84**			**98**	**100.0%**	**0.38 [−1.95, 2.71]**
Heterogeneity: Tau² = 4.13; Chi² = 87.60, df = 2 (P < 0.00001); I² = 98%								
Test for overall effect: Z = 0.32 (P = 0.75)								
Test for subgroup differences: Chi² = 0.60, df = 1 (P = 0.44), I² = 0%								

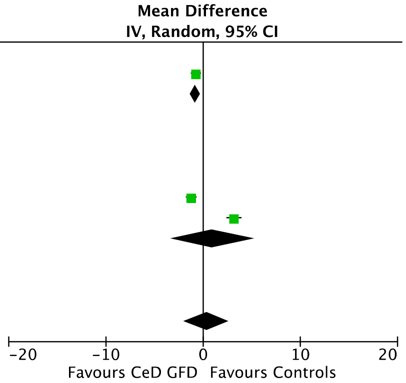

S16G- Forest plot comparison of means of serum Selenium levels between CeD on GFD and non-CeD controls by GFD length

	CeD GFD			Controls				Mean Difference
Study or Subgroup	Mean	SD	Total	Mean	SD	Total	Weight	IV, Random, 95% CI
**58.1.1 Short-Term GFD**								
Cortigiani 1989	7.11	1.26	36	8.34	1.34	51	33.5%	-1.23 [-1.78, -0.68]
Ünal 2012	12.42	1.23	17	9.25	1.21	20	32.9%	3.17 [2.38, 3.96]
**Subtotal (95% CI)**			**53**			**71**	**66.4%**	**0.96 [-3.35, 5.27]**
Heterogeneity: $Tau^2 = 9.56$; $Chi^2 = 80.16$, df = 1 (P < 0.00001); $I^2 = 99\%$								
Test for overall effect: Z = 0.44 (P = 0.66)								
**58.1.2 Unknown GFD**								
Kalita 2002	6.44	1.04	31	7.19	1	27	33.6%	-0.75 [-1.28, -0.22]
**Subtotal (95% CI)**			**31**			**27**	**33.6%**	**-0.75 [-1.28, -0.22]**
Heterogeneity: Not applicable								
Test for overall effect: Z = 2.80 (P = 0.005)								
**Total (95% CI)**			**84**			**98**	**100.0%**	**0.38 [-1.95, 2.71]**
Heterogeneity: $Tau^2 = 4.13$; $Chi^2 = 87.60$, df = 2 (P < 0.00001); $I^2 = 98\%$								
Test for overall effect: Z = 0.32 (P = 0.75)								
Test for subgroup differences: $Chi^2 = 0.60$, df = 1 (P = 0.44), $I^2 = 0\%$								

Mean Difference
IV, Random, 95% CI

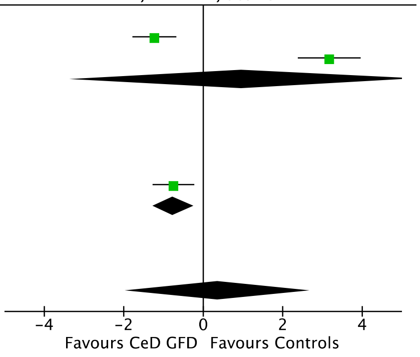

S16H- Forest plot comparison of means of erythrocyte Selenium levels between CeD on GFD and CeD not on GFD

	CeD on GFD			CeD nonGFD				Mean Difference
Study or Subgroup	Mean	SD	Total	Mean	SD	Total	Weight	IV, Random, 95% CI
Boda 1989	298	129	25	228	158	24	100.0%	70.00 [-10.95, 150.95]
**Total (95% CI)**			**25**			**24**	**100.0%**	**70.00 [-10.95, 150.95]**
Heterogeneity: Not applicable								
Test for overall effect: Z = 1.69 (P = 0.09)								

Mean Difference
IV, Random, 95% CI

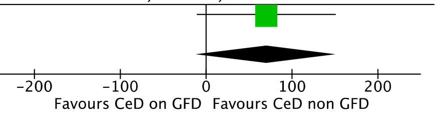

S16I- Forest plot comparison of means of erythrocyte Selenium levels between CeD on GFD and non-CeD controls

	CeD on GFD			Control				Mean Difference
Study or Subgroup	Mean	SD	Total	Mean	SD	Total	Weight	IV, Random, 95% CI
Boda 1989	298	129	25	465	136	15	100.0%	-167.00 [-252.40, -81.60]
**Total (95% CI)**			**25**			**15**	**100.0%**	**-167.00 [-252.40, -81.60]**
Heterogeneity: Not applicable								
Test for overall effect: Z = 3.83 (P = 0.0001)								

Mean Difference
IV, Random, 95% CI

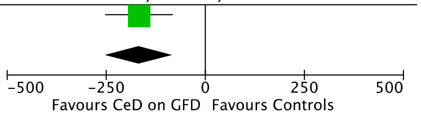

<u>Supplementary Figure S17. Zinc</u>

S17A- Forest plot comparison of Zinc deficiency between CeD on GFD and CeD not on GFD by study design

Study or Subgroup	CeD GFD Events	CeD GFD Total	CeD non GFD Events	CeD non GFD Total	Weight	Odds Ratio M-H, Random, 95% CI
**31.1.1 Cohort**						
Högberg 2009	0	14	9	11	31.4%	0.01 [0.00, 0.21]
McGrogan 2021	20	44	0	25	32.4%	42.67 [2.44, 744.90]
**Subtotal (95% CI)**		**58**		**36**	**63.9%**	**0.64 [0.00, 2638.90]**
Total events	20		9			
Heterogeneity: $Tau^2 = 33.74$; $Chi^2 = 15.35$, df = 1 (P < 0.0001); $I^2 = 93\%$						
Test for overall effect: Z = 0.11 (P = 0.92)						
**31.1.2 Case-Control**						
Selbuz 2021	4	11	10	16	36.1%	0.34 [0.07, 1.68]
**Subtotal (95% CI)**		**11**		**16**	**36.1%**	**0.34 [0.07, 1.68]**
Total events	4		10			
Heterogeneity: Not applicable						
Test for overall effect: Z = 1.32 (P = 0.19)						
**Total (95% CI)**		**69**		**52**	**100.0%**	**0.52 [0.01, 35.25]**
Total events	24		19			
Heterogeneity: $Tau^2 = 12.11$; $Chi^2 = 17.13$, df = 2 (P = 0.0002); $I^2 = 88\%$						
Test for overall effect: Z = 0.30 (P = 0.76)						
Test for subgroup differences: $Chi^2 = 0.02$, df = 1 (P = 0.89), $I^2 = 0\%$						

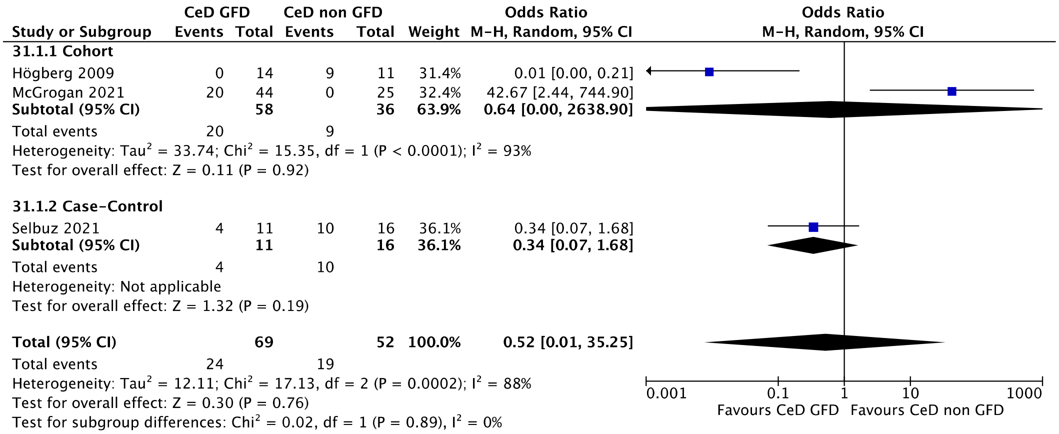

S17B- Forest plot comparison of means of serum zinc levels between CeD on GFD and CeD not on GFD

Study or Subgroup	CeD GFD Mean	CeD GFD SD	CeD GFD Total	CeD non GFD Mean	CeD non GFD SD	CeD non GFD Total	Weight	Mean Difference IV, Random, 95% CI
Corazza 1988	13.78	2.34	18	9.48	2.63	30	22.2%	4.30 [2.87, 5.73]
Henker 1985	18.1	3.8	63	14.3	3.5	48	22.3%	3.80 [2.44, 5.16]
Högberg 2009	14.2	1.6	14	9.7	2	11	22.1%	4.50 [3.05, 5.95]
McGrogan 2021	10.44	1.84	41	10.85	1.3	24	23.3%	-0.41 [-1.18, 0.36]
Singhal 2008	12.84	7.95	7	9.79	5.2	23	10.1%	3.05 [-3.21, 9.31]
**Total (95% CI)**			**143**			**136**	**100.0%**	**3.01 [0.38, 5.64]**
Heterogeneity: $Tau^2 = 7.61$; $Chi^2 = 65.33$, df = 4 (P < 0.00001); $I^2 = 94\%$								
Test for overall effect: Z = 2.24 (P = 0.03)								

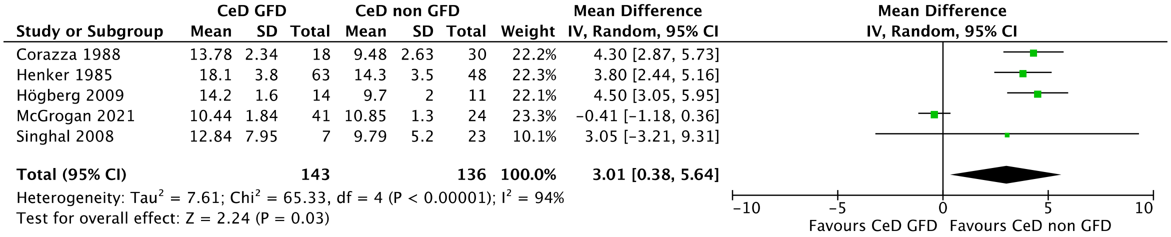

S17C- Forest plot comparison of means of serum zinc levels between CeD on GFD and CeD not on GFD by study design

Study or Subgroup	CeD GFD Mean	CeD GFD SD	CeD GFD Total	CeD non GFD Mean	CeD non GFD SD	CeD non GFD Total	Weight	Mean Difference IV, Random, 95% CI
**25.1.1 Cohort**								
Henker 1985	18.1	3.8	63	14.3	3.5	48	22.3%	3.80 [2.44, 5.16]
Högberg 2009	14.2	1.6	14	9.7	2	11	22.1%	4.50 [3.05, 5.95]
McGrogan 2021	10.44	1.84	41	10.85	1.3	24	23.3%	-0.41 [-1.18, 0.36]
**Subtotal (95% CI)**			**118**			**83**	**67.7%**	**2.59 [-0.82, 6.00]**
Heterogeneity: $Tau^2 = 8.70$; $Chi^2 = 50.75$, df = 2 (P < 0.00001); $I^2 = 96\%$								
Test for overall effect: Z = 1.49 (P = 0.14)								
**25.1.2 Case-Control**								
Corazza 1988	13.78	2.34	18	9.48	2.63	30	22.2%	4.30 [2.87, 5.73]
Singhal 2008	12.84	7.95	7	9.79	5.2	23	10.1%	3.05 [-3.21, 9.31]
**Subtotal (95% CI)**			**25**			**53**	**32.3%**	**4.24 [2.84, 5.63]**
Heterogeneity: $Tau^2 = 0.00$; $Chi^2 = 0.15$, df = 1 (P = 0.70); $I^2 = 0\%$								
Test for overall effect: Z = 5.94 (P < 0.00001)								
**Total (95% CI)**			**143**			**136**	**100.0%**	**3.01 [0.38, 5.64]**
Heterogeneity: $Tau^2 = 7.61$; $Chi^2 = 65.33$, df = 4 (P < 0.00001); $I^2 = 94\%$								
Test for overall effect: Z = 2.24 (P = 0.03)								
Test for subgroup differences: $Chi^2 = 0.77$, df = 1 (P = 0.38), $I^2 = 0\%$								

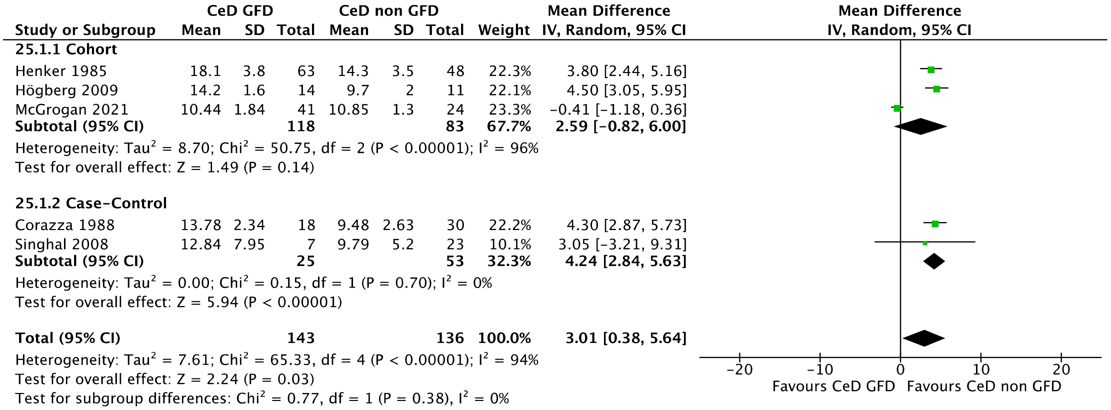

S17D- Forest plot comparison of means of serum zinc levels between CeD on GFD and non-CeD controls

Study or Subgroup	CeD GFD Mean	CeD GFD SD	CeD GFD Total	Controls Mean	Controls SD	Controls Total	Weight	Mean Difference IV, Random, 95% CI
Corazza 1988	13.78	2.34	18	15.67	1.51	30	96.0%	−1.89 [−3.10, −0.68]
Singhal 2008	12.84	7.95	7	14.37	2.14	27	4.0%	−1.53 [−7.47, 4.41]
**Total (95% CI)**			**25**			**57**	**100.0%**	**−1.88 [−3.06, −0.69]**

Heterogeneity: $Tau^2 = 0.00$; $Chi^2 = 0.01$, df = 1 (P = 0.91); $I^2 = 0\%$
Test for overall effect: Z = 3.10 (P = 0.002)

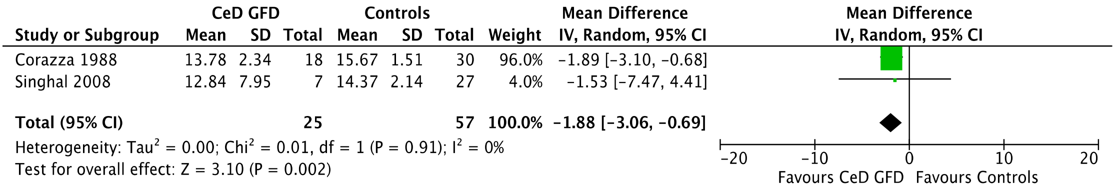

Supplementary Figure S18. NCWS data

S18A- Forest plot comparison of Vitamin B12 deficiency between NCWS and controls

Study or Subgroup	NCWS Events	NCWS Total	Control Events	Control Total	Weight	Odds Ratio M-H, Random, 95% CI
Mansueto 2023	30	244	2	124	100.0%	8.55 [2.01, 36.40]
**Total (95% CI)**		**244**		**124**	**100.0%**	**8.55 [2.01, 36.40]**
Total events	30		2			

Heterogeneity: Not applicable
Test for overall effect: Z = 2.90 (P = 0.004)

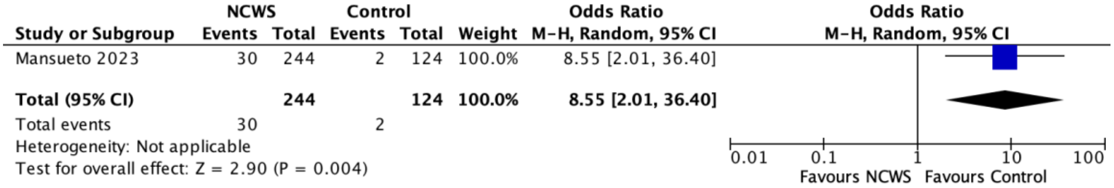

S18B- Forest plot comparison of Vitamin B12 serum levels between NCWS and controls

Study or Subgroup	NCWS Mean	NCWS SD	NCWS Total	Control Mean	Control SD	Control Total	Weight	Mean Difference IV, Fixed, 95% CI
Mansueto 2023	259.3	105.8	244	394.3	124.8	124	100.0%	−135.00 [−160.67, −109.33]
**Total (95% CI)**			**244**			**124**	**100.0%**	**−135.00 [−160.67, −109.33]**

Heterogeneity: Not applicable
Test for overall effect: Z = 10.31 (P < 0.00001)

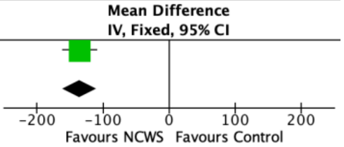

S18C- Forest plot comparison of Ferritin deficiency between NCWS and controls

Study or Subgroup	NCWS Events	NCWS Total	Control Events	Control Total	Weight	Odds Ratio M-H, Random, 95% CI
Mansueto 2023	99	244	16	124	100.0%	4.61 [2.57, 8.26]
**Total (95% CI)**		**244**		**124**	**100.0%**	**4.61 [2.57, 8.26]**
Total events	99		16			

Heterogeneity: Not applicable
Test for overall effect: Z = 5.13 (P < 0.00001)

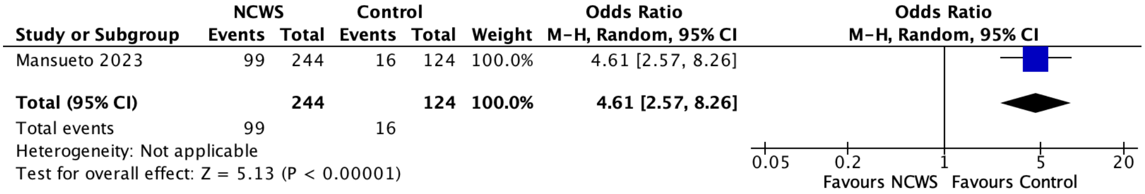

S18D- Forest plot comparison of Ferritin serum levels between NCWS and controls

Study or Subgroup	NCWS Mean	NCWS SD	NCWS Total	Control Mean	Control SD	Control Total	Weight	Mean Difference IV, Random, 95% CI
Mansueto 2023	74.25	66.3	244	92.1	70.8	124	100.0%	−17.85 [−32.83, −2.87]
**Total (95% CI)**			**244**			**124**	**100.0%**	**−17.85 [−32.83, −2.87]**

Heterogeneity: Not applicable
Test for overall effect: Z = 2.33 (P = 0.02)

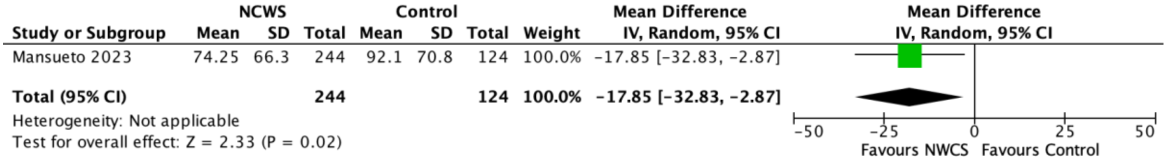

S18E- Forest plot comparison of Folate deficiency between NCWS and controls

Study or Subgroup	NCWS Events	NCWS Total	Control Events	Control Total	Weight	Odds Ratio M-H, Random, 95% CI
Mansueto 2023	34	244	0	124	100.0%	40.81 [2.48, 671.51]
**Total (95% CI)**		**244**		**124**	**100.0%**	**40.81 [2.48, 671.51]**
Total events	34		0			
Heterogeneity: Not applicable						
Test for overall effect: Z = 2.60 (P = 0.009)						

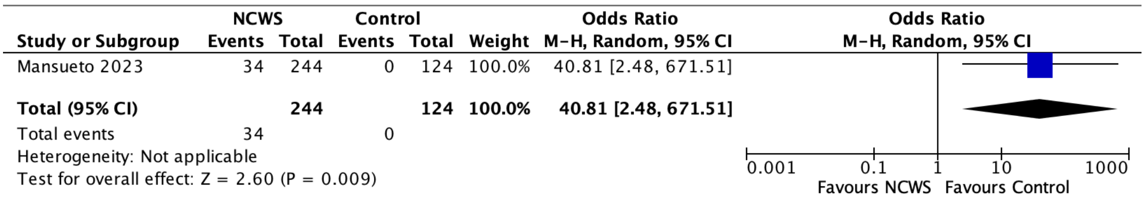

S18F- Forest plot comparison of Folate serum levels between NCWS and controls

Study or Subgroup	NCWS Mean	NCWS SD	NCWS Total	Control Mean	Control SD	Control Total	Weight	Mean Difference IV, Fixed, 95% CI
Mansueto 2023	7	2.4	244	10.4	3.9	124	100.0%	-3.40 [-4.15, -2.65]
**Total (95% CI)**			**244**			**124**	**100.0%**	**-3.40 [-4.15, -2.65]**
Heterogeneity: Not applicable								
Test for overall effect: Z = 8.89 (P < 0.00001)								

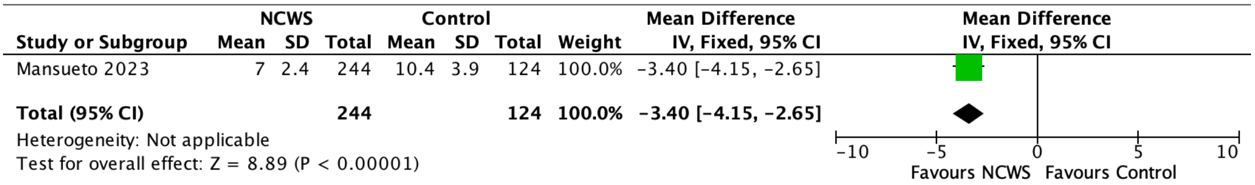

S18G- Forest plot comparison of Iron deficiency between NCWS and controls

Study or Subgroup	NCWS Events	NCWS Total	Control Events	Control Total	Weight	Odds Ratio M-H, Random, 95% CI
Mansueto 2023	47	244	0	124	100.0%	59.89 [3.66, 980.26]
**Total (95% CI)**		**244**		**124**	**100.0%**	**59.89 [3.66, 980.26]**
Total events	47		0			
Heterogeneity: Not applicable						
Test for overall effect: Z = 2.87 (P = 0.004)						

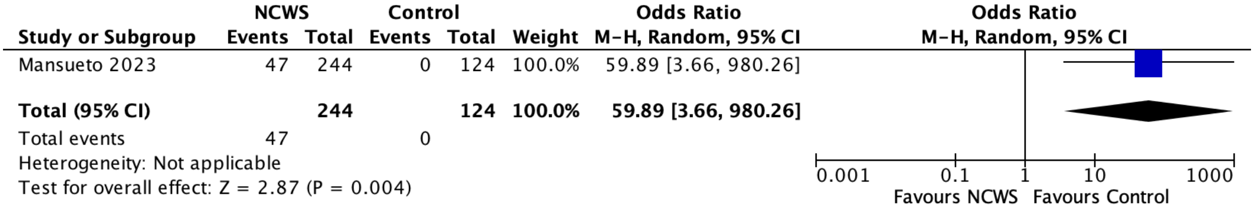

S18H- Forest plot comparison of Iron serum levels between NCWS and controls

Study or Subgroup	NCWS Mean	NCWS SD	NCWS Total	Control Mean	Control SD	Control Total	Weight	Mean Difference IV, Random, 95% CI
Mansueto 2023	74.7	35.4	244	83.3	34.3	124	100.0%	-8.60 [-16.10, -1.10]
**Total (95% CI)**			**244**			**124**	**100.0%**	**-8.60 [-16.10, -1.10]**
Heterogeneity: Not applicable								
Test for overall effect: Z = 2.25 (P = 0.02)								

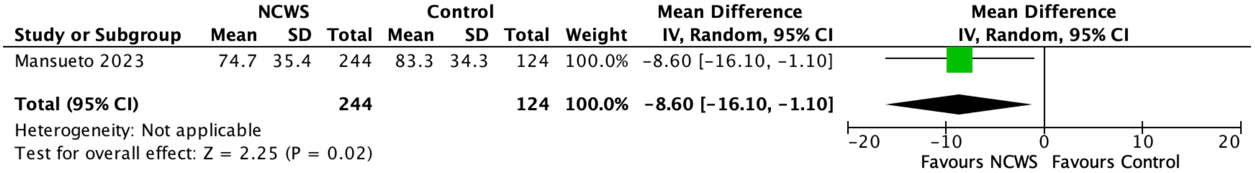

Supplementary Figure S19. - Forest plot of micronutrient deficiencies in CeD on GFD compared to CeD not on GFD with corresponding risk of Bias. **A-** Vitamin 25 (OH) deficiency sub-grouped by duration of GFD **B-** Vitamin E deficiency sub-grouped by study design **C-** Vitamin K deficiency as controlled by triglycerides **D-** Ferritin deficiency **E-** Iron deficiency **F-** Zinc deficiency

A- Vitamin 25 (OH) D

Study or Subgroup	CeD GFD Events	CeD GFD Total	CeD non GFD Events	CeD non GFD Total	Weight	Odds Ratio M-H, Random, 95% CI
**32.1.1 Short-Term GFD**						
Klimov 2017	30	37	16	22	9.3%	1.61 [0.46, 5.60]
McGrogan 2021	25	44	5	25	10.1%	5.26 [1.67, 16.58]
Selbuz 2021	11	20	23	31	9.8%	0.43 [0.13, 1.40]
Uyanikoglu 2021	32	40	31	40	10.7%	1.16 [0.40, 3.40]
**Subtotal (95% CI)**		**141**		**118**	**39.9%**	**1.44 [0.52, 3.96]**
Total events	98		75			
Heterogeneity: Tau² = 0.72; Chi² = 9.12, df = 3 (P = 0.03); I² = 67%						
Test for overall effect: Z = 0.70 (P = 0.48)						
**32.1.2 Long-Term GFD**						
Ciacci 2020	15	55	25	50	13.1%	0.38 [0.17, 0.84]
González 1995	0	12	1	20	2.4%	0.52 [0.02, 13.80]
Keaveny 1996	4	16	11	19	7.9%	0.24 [0.06, 1.04]
Margoni 2012	12	36	16	45	12.0%	0.91 [0.36, 2.28]
Mazure 1994	0	6	2	18	2.5%	0.51 [0.02, 12.08]
Piatek-Guziewicz 2017, May	61	92	37	53	13.9%	0.85 [0.41, 1.76]
Piatek-Guziewicz 2017, Nov	21	31	26	29	8.2%	0.24 [0.06, 1.00]
**Subtotal (95% CI)**		**248**		**234**	**60.1%**	**0.55 [0.36, 0.84]**
Total events	113		118			
Heterogeneity: Tau² = 0.00; Chi² = 5.88, df = 6 (P = 0.44); I² = 0%						
Test for overall effect: Z = 2.80 (P = 0.005)						
**Total (95% CI)**		**389**		**352**	**100.0%**	**0.76 [0.44, 1.30]**
Total events	211		193			
Heterogeneity: Tau² = 0.41; Chi² = 21.98, df = 10 (P = 0.02); I² = 55%						
Test for overall effect: Z = 1.01 (P = 0.31)						
Test for subgroup differences: Chi² = 2.92, df = 1 (P = 0.09), I² = 65.8%						

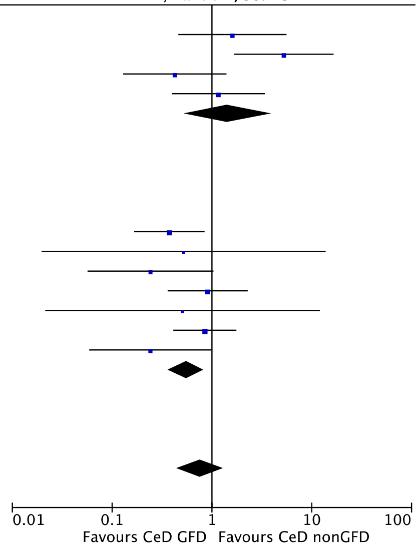

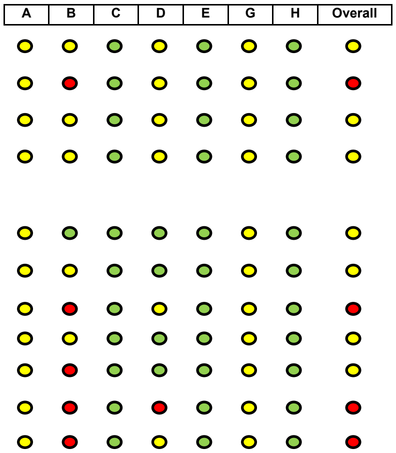

B- Vitamin E

Study or Subgroup	CeD GFD Events	CeD GFD Total	CeD non GFD Events	CeD non GFD Total	Weight	Odds Ratio M-H, Random, 95% CI
**46.1.1 Erythrocyte level**						
Hozyasz 2003	2	12	18	18	24.7%	0.01 [0.00, 0.15]
Romańczuk 2016	29	48	7	7	25.7%	0.10 [0.01, 1.87]
**Subtotal (95% CI)**		**60**		**25**	**50.4%**	**0.03 [0.00, 0.41]**
Total events	31		25			
Heterogeneity: Tau² = 1.49; Chi² = 1.62, df = 1 (P = 0.20); I² = 38%						
Test for overall effect: Z = 2.60 (P = 0.009)						
**46.1.2 Plasma level**						
Selbuz 2021	1	14	0	14	23.9%	3.22 [0.12, 86.09]
**Subtotal (95% CI)**		**14**		**14**	**23.9%**	**3.22 [0.12, 86.09]**
Total events	1		0			
Heterogeneity: Not applicable						
Test for overall effect: Z = 0.70 (P = 0.49)						
**46.1.3 Plasma by cholesterol**						
McGrogan 2021	0	36	18	25	25.7%	0.01 [0.00, 0.10]
**Subtotal (95% CI)**		**36**		**25**	**25.7%**	**0.01 [0.00, 0.10]**
Total events	0		18			
Heterogeneity: Not applicable						
Test for overall effect: Z = 3.49 (P = 0.0005)						
**Total (95% CI)**		**110**		**64**	**100.0%**	**0.06 [0.00, 0.94]**
Total events	32		43			
Heterogeneity: Tau² = 5.87; Chi² = 10.24, df = 3 (P = 0.02); I² = 71%						
Test for overall effect: Z = 2.00 (P = 0.05)						
Test for subgroup differences: Chi² = 8.53, df = 2 (P = 0.01), I² = 76.6%						

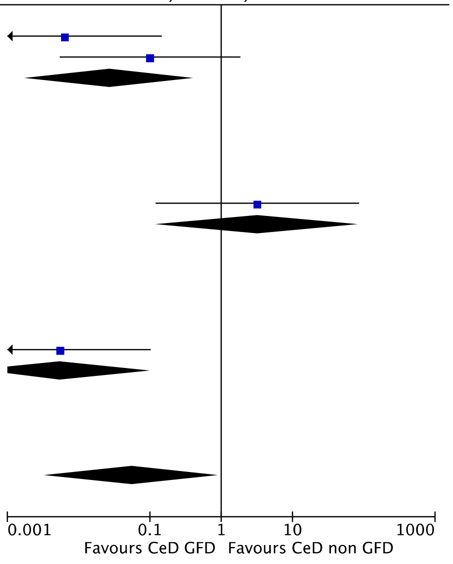

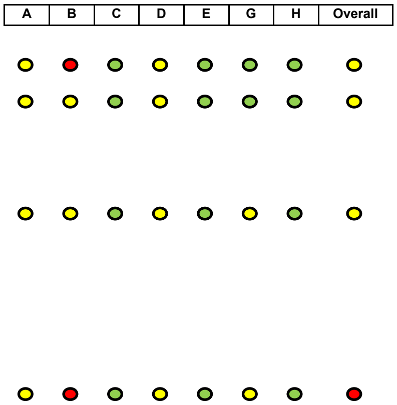

C- Vitamin K

Study or Subgroup	CeD GFD Events	CeD GFD Total	CeD nonGFD Events	CeD nonGFD Total	Weight	Odds Ratio M-H, Random, 95% CI
McGrogan 2021	0	38	5	24	100.0%	0.05 [0.00, 0.88]
**Total (95% CI)**		**38**		**24**	**100.0%**	**0.05 [0.00, 0.88]**
Total events	0		5			

Heterogeneity: Not applicable
Test for overall effect: Z = 2.05 (P = 0.04)

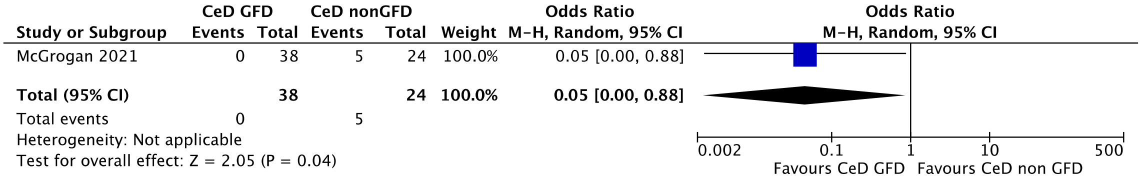

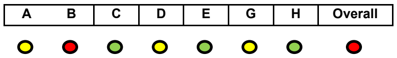

D- Ferritin

Study or Subgroup	CeD GFD Events	CeD GFD Total	CeD non GFD Events	CeD non GFD Total	Weight	Odds Ratio M-H, Random, 95% CI
Kemppainen 1995	4	52	12	40	51.2%	0.19 [0.06, 0.66]
McGrogan 2021	6	43	19	24	48.8%	0.04 [0.01, 0.16]
**Total (95% CI)**		**95**		**64**	**100.0%**	**0.09 [0.02, 0.41]**
Total events	10		31			

Heterogeneity: $Tau^2 = 0.73$; $Chi^2 = 2.76$, df = 1 (P = 0.10); $I^2 = 64\%$
Test for overall effect: Z = 3.13 (P = 0.002)

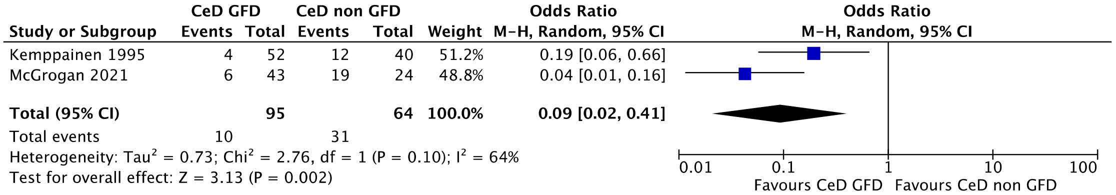

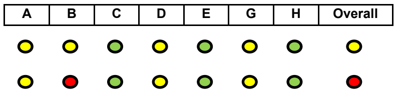

E- Iron

Study or Subgroup	CeD GFD Events	CeD GFD Total	CeD non GFD Events	CeD non GFD Total	Weight	Odds Ratio M-H, Random, 95% CI
Anand 1977	3	14	9	11	14.7%	0.06 [0.01, 0.45]
Kemppainen 1995	9	52	15	40	48.5%	0.35 [0.13, 0.91]
Selbuz 2021	6	22	18	33	36.8%	0.31 [0.10, 1.00]
**Total (95% CI)**		**88**		**84**	**100.0%**	**0.26 [0.12, 0.58]**
Total events	18		42			

Heterogeneity: $Tau^2 = 0.11$; $Chi^2 = 2.49$, df = 2 (P = 0.29); $I^2 = 20\%$
Test for overall effect: Z = 3.30 (P = 0.0010)

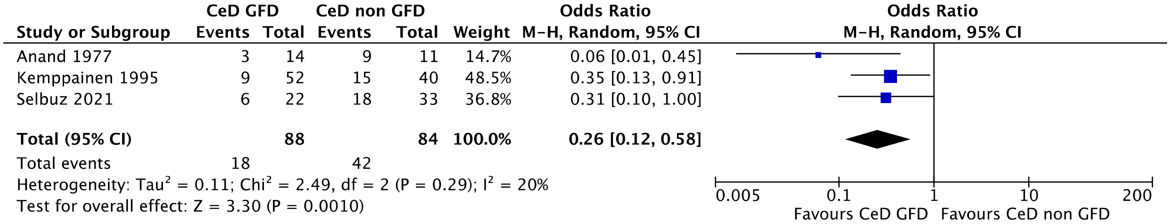

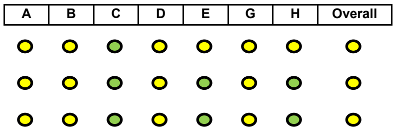

F- Zinc

Study or Subgroup	CeD GFD Events	CeD GFD Total	CeD non GFD Events	CeD non GFD Total	Weight	Odds Ratio M-H, Random, 95% CI
Högberg 2009	0	14	9	11	31.4%	0.01 [0.00, 0.21]
McGrogan 2021	19	41	0	24	32.4%	42.47 [2.42, 745.15]
Selbuz 2021	4	11	10	16	36.2%	0.34 [0.07, 1.68]
**Total (95% CI)**		**66**		**51**	**100.0%**	**0.52 [0.01, 34.68]**
Total events	23		19			

Heterogeneity: $Tau^2 = 12.01$; $Chi^2 = 16.99$, df = 2 (P = 0.0002); $I^2 = 88\%$
Test for overall effect: Z = 0.30 (P = 0.76)

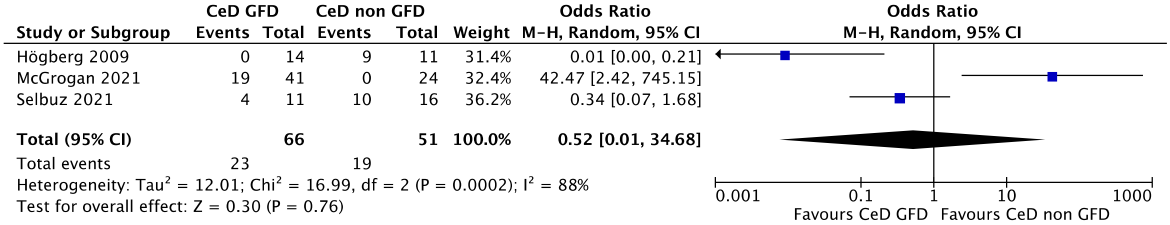

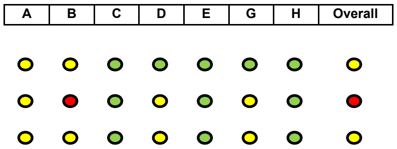

* A- Bias due to confounding, B- Bias due to selection of participants into the study, C- Bias in classification of interventions, D- Bias due to deviation from intervention, E- Bias due to missing data, G- Bias in measurements of outcomes, H- Bias in selection of the reported result, Overall Bias. Green- low risk of bias, yellow- moderate risk of bias, red- severe risk of bias

Supplementary Figure S20. Forest plots comparison of micronutrient deficiencies in CeD on GFD compared to non-CeD controls with corresponding risk of Bias. **A-**Vitamin D deficiency **B-**Vitamin D deficiency stratified by study design **C-**Vitamin D deficiency stratified by duration of GFD length, **D-** Vitamin E deficiency in case-control study

A- Vitamin 25 (OH) D

Study or Subgroup	CeD GFD Events	CeD GFD Total	Control Events	Control Total	Weight	Odds Ratio M-H, Random, 95% CI
Ballestero-Fernández 2021	22	64	31	74	20.5%	0.73 [0.36, 1.45]
Bayrak 2020	97	103	108	135	18.5%	4.04 [1.60, 10.20]
Klimov 2017	30	37	0	14	6.1%	117.93 [6.30, 2209.02]
Piatek-Guziewicz 2017, May	61	92	18	52	20.3%	3.72 [1.82, 7.61]
Piatek-Guziewicz 2017, Nov	21	31	14	25	17.0%	1.65 [0.55, 4.91]
Uyanikoglu 2021	32	40	26	40	17.7%	2.15 [0.78, 5.92]
**Total (95% CI)**		**367**		**340**	**100.0%**	**2.64 [1.14, 6.08]**
Total events	263		197			

Heterogeneity: Tau² = 0.76; Chi² = 21.29, df = 5 (P = 0.0007); I² = 77%
Test for overall effect: Z = 2.27 (P = 0.02)

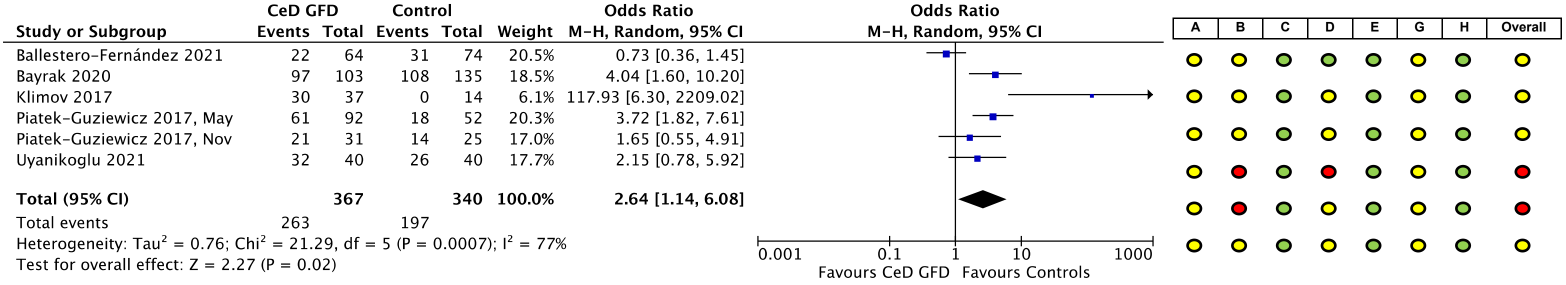

B- Vitamin 25 (OH) D

Study or Subgroup	CeD GFD Events	CeD GFD Total	Control Events	Control Total	Weight	Odds Ratio M-H, Random, 95% CI
**53.1.1 Cross-Sectional**						
Ballestero-Fernández 2021	22	64	31	74	20.5%	0.73 [0.36, 1.45]
Bayrak 2020	97	103	108	135	18.5%	4.04 [1.60, 10.20]
**Subtotal (95% CI)**		**167**		**209**	**39.0%**	**1.67 [0.31, 9.03]**
Total events	119		139			
Heterogeneity: Tau² = 1.32; Chi² = 8.56, df = 1 (P = 0.003); I² = 88%						
Test for overall effect: Z = 0.59 (P = 0.55)						
**53.1.2 Case-Control**						
Klimov 2017	30	37	0	14	6.1%	117.93 [6.30, 2209.02]
Piatek-Guziewicz 2017, May	61	92	18	52	20.3%	3.72 [1.82, 7.61]
Piatek-Guziewicz 2017, Nov	21	31	14	25	17.0%	1.65 [0.55, 4.91]
Uyanikoglu 2021	32	40	26	40	17.7%	2.15 [0.78, 5.92]
**Subtotal (95% CI)**		**200**		**131**	**61.0%**	**3.42 [1.30, 8.97]**
Total events	144		58			
Heterogeneity: Tau² = 0.57; Chi² = 8.39, df = 3 (P = 0.04); I² = 64%						
Test for overall effect: Z = 2.49 (P = 0.01)						
**Total (95% CI)**		**367**		**340**	**100.0%**	**2.64 [1.14, 6.08]**
Total events	263		197			

Heterogeneity: Tau² = 0.76; Chi² = 21.29, df = 5 (P = 0.0007); I² = 77%
Test for overall effect: Z = 2.27 (P = 0.02)
Test for subgroup differences: Chi² = 0.52, df = 1 (P = 0.47), I² = 0%

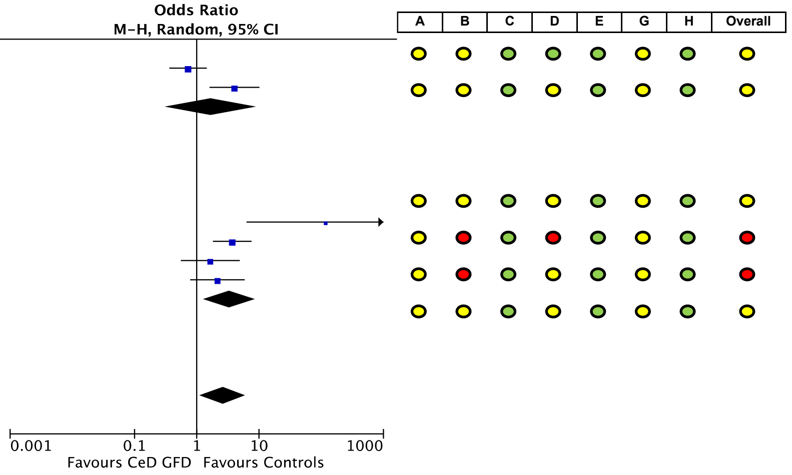

C- Vitamin 25 (OH) D

Study or Subgroup	CeD GFD Events	CeD GFD Total	Control Events	Control Total	Weight	Odds Ratio M-H, Random, 95% CI
**52.1.1 Short-Term GFD**						
Ballestero-Fernández 2021	22	64	31	74	20.5%	0.73 [0.36, 1.45]
Klimov 2017	30	37	0	14	6.1%	117.93 [6.30, 2209.02]
Uyanikoglu 2021	32	40	26	40	17.7%	2.15 [0.78, 5.92]
**Subtotal (95% CI)**		**141**		**128**	**44.3%**	**3.19 [0.49, 20.60]**
Total events	84		57			
Heterogeneity: Tau² = 2.09; Chi² = 14.13, df = 2 (P = 0.0009); I² = 86%						
Test for overall effect: Z = 1.22 (P = 0.22)						
**52.1.2 Long-Term GFD**						
Bayrak 2020	97	103	108	135	18.5%	4.04 [1.60, 10.20]
Piatek-Guziewicz 2017, May	61	92	18	52	20.3%	3.72 [1.82, 7.61]
Piatek-Guziewicz 2017, Nov	21	31	14	25	17.0%	1.65 [0.55, 4.91]
**Subtotal (95% CI)**		**226**		**212**	**55.7%**	**3.21 [1.94, 5.30]**
Total events	179		140			
Heterogeneity: Tau² = 0.00; Chi² = 1.83, df = 2 (P = 0.40); I² = 0%						
Test for overall effect: Z = 4.54 (P < 0.00001)						
**Total (95% CI)**		**367**		**340**	**100.0%**	**2.64 [1.14, 6.08]**
Total events	263		197			

Heterogeneity: Tau² = 0.76; Chi² = 21.29, df = 5 (P = 0.0007); I² = 77%
Test for overall effect: Z = 2.27 (P = 0.02)
Test for subgroup differences: Chi² = 0.00, df = 1 (P = 1.00), I² = 0%

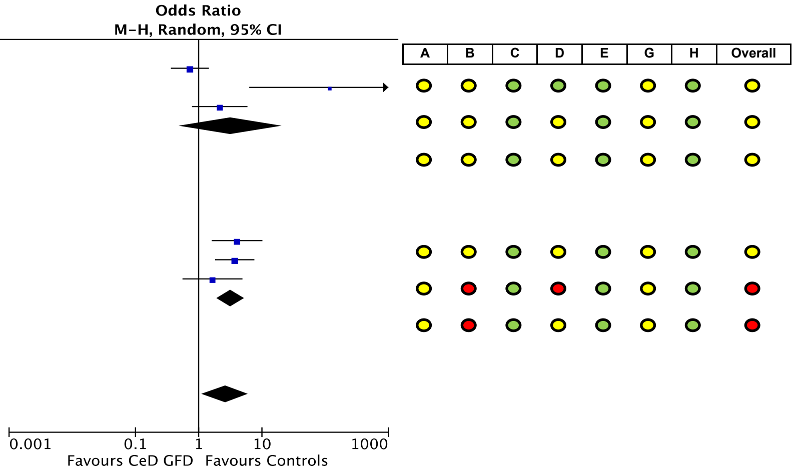

D- Vitamin E

Study or Subgroup	CeD GFD Events	CeD GFD Total	Control Events	Control Total	Weight	Odds Ratio M-H, Random, 95% CI
**28.1.1 Case- Control**						
Romańczuk 2016	29	48	1	20	100.0%	29.00 [3.58, 235.03]
**Subtotal (95% CI)**		**48**		**20**	**100.0%**	**29.00 [3.58, 235.03]**
Total events	29		1			
Heterogeneity: Not applicable						
Test for overall effect: Z = 3.15 (P = 0.002)						
**Total (95% CI)**		**48**		**20**	**100.0%**	**29.00 [3.58, 235.03]**
Total events	29		1			
Heterogeneity: Not applicable						
Test for overall effect: Z = 3.15 (P = 0.002)						
Test for subgroup differences: Not applicable						

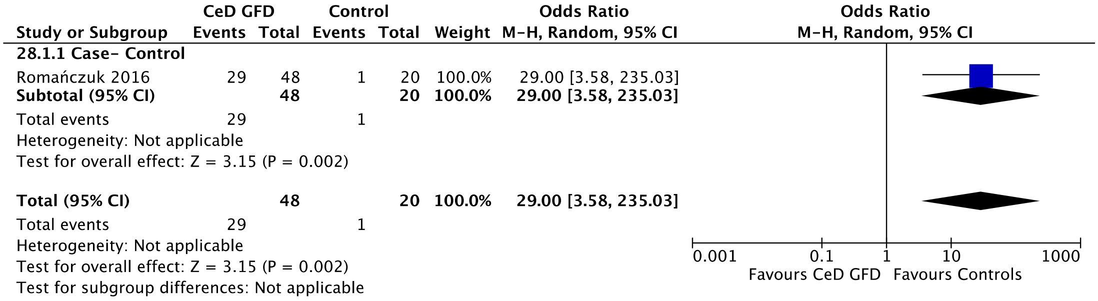

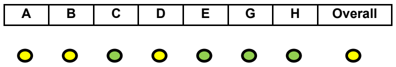

* A- Bias due to confounding, B- Bias due to selection of participants into the study, C- Bias in classification of interventions, D- Bias due to deviation from intervention, E- Bias due to missing data, G- Bias in measurements of outcomes, H- Bias in selection of the reported result, Overall Bias. Green- low risk of bias, yellow- moderate risk of bias, red- severe risk of bias

Supplementary Table ST7. Summary table of nutrient deficiencies in treated CeD compared with untreated CeD or non-celiac controls and NCWS. Number of studies in brackets.

*- statistically significant

Treated vs untreated CeD			
**Higher risk**	**Lower risk**	**No difference**	**No data**
Vitamin A (3)	Vitamin B1(1), Vitamin B6 (1), Vitamin B12 (3)		Vitamin B2
Selenium (1)	Vitamin E (4)*		Vitamin C
Vitamin D short term GFD (4)	Vitamin D (11)<br>Vitamin D long term GFD (7)*		
Copper (1)	Vitamin K (1)*		
	Calcium short (3)* + long term GFD (3)		
	Vitamin 1,25 OH D (1)		
	Folate (2)		
	Iron (3)*		
	Magnesium (2)		
	Ferritin (2)*		
	Zinc(1)		
**Treated CeD vs non-CeD controls**			
**Higher risk**	**Lower risk**	**No difference**	**No data**
Vitamin D (5)	Selenium(1)	Vitamin B1 (1)	Copper, Vitamin A, Vitamin C
Vitamin D long term GFD (2)*	Vitamin B12 (1)	Vitamin B2 (1)	
Vitamin E(1)*		Vitamin B6 (1)	
Folate (1)		Calcium	
Ferritin (1)		Magnesium	
Iron (1)			
**NCWS vs non-CeD controls**			
**Higher risk**	**Lower risk**	**No difference**	**No data**
Vitamin B12(1)*			Magnesium. Calcium Copper, Vitamin A, Vitamin C, Vitamin D, Vitamin B1/B2/B6 Selenium, Vitamin E
Ferritin (1)*			
Folate (1)*			
Iron (1)*			

Supplementary Table S8. Summary table of nutrients found in each included study

Micronutrient		CeD Not on GFD		Non CeD Controls		NCWS	
		Study	Characteristics *	Study	Characteristics	Study	Characteristics
Vitamin A	Dichotomous	Hozyasz<br>McGrogan<br>Selbuz	C/ LT/CH<br>C/ ST/CH<br>CC/ST/CH	NA	NA	NA	NA
	Continuous	Hozyasz<br>McGrogan	C/ LT/CH<br>C/ ST/CH	NA	NA	NA	NA
Vitamin B1	Dichotomous	McGrogan	C/ ST/CH	Szaflarska-Poplawska	C/ST/CH	NA	NA
	Continuous	McGrogan	C/ ST/CH	Szaflarska-Poplawska	C/ST/CH	NA	NA
Vitamin B2	Dichotomous	McGrogan	C/ ST/CH	Szaflarska-Poplawska	C/ST/CH	NA	NA
	Continuous	McGrogan<br>Dickey 2008	C/ ST/CH<br>CC/ST/AD	Dickey 2008<br>Szaflarska-Poplawska	CC/ST/AD<br>C/ST/CH	NA	NA
Vitamin B6	Dichotomous	McGrogan	C/ ST/CH	Szaflarska-Poplawska	C/ST/CH	NA	NA
	Continuous	McGrogan<br>Reinken	C/ ST/CH<br>CC/?/CH	Reinken<br>Szaflarska-Poplawska<br>Valente	CC/?/CH<br>C/ST/CH<br>CS/ST/AD	NA	NA
Vitamin B12	Dichotomous	McGrogan<br>Selbuz<br>Kemppainen	C/ ST/CH<br>CC/ST/CH<br>C/ST/AD	Bayrak<br>Szaflarska-Poplawska<br>Mansueto	CS/LT/CH<br>C/ST/CH<br>CC/ST/AD	Mansueto	CC/ST/AD
	Continuous	McGrogan<br>Kemppainen (RBCs)<br>Dickey<br>Volkan	C/ ST/CH<br>C/ST/AD<br>CC/ST/AD<br>CC/ST/CH	Efe<br>Dickey<br>Bayrak<br>Isikay<br>Szaflarska-Poplawska<br>Valente<br>Volkan<br>Mansueto	CC/ST/CH<br>CC/ST/AD<br>CS/LT/CH<br>CS/ST/CH<br>C/ST/CH<br>CS/ST/AD<br>CC/ST/CH<br>CC/ST/AD	Mansueto	CC/ST/AD
Vitamin C	Dichotomous	McGrogan<br>(No control)	C/ ST/CH (no control)	NA	NA	NA	NA
	Continuous	McGrogan<br>(No control)	C/ ST/CH (no control)	NA	NA	NA	NA
25(OH)Vitamin D	Dichotomous	McGrogan<br>Piatek May<br>Piatek Nov<br>Selbuz<br>Ciacci<br>Gonzalez<br>Keaveny<br>Klimov<br>Margoni<br>Mazure<br>Uyanikoglu	C/ ST/CH<br>CC/LT/AD<br>CC/LT/AD<br>CC/ST/CH<br>CC/LT/AD<br>CS/LT/AD<br>C/LT/AD<br>CC/ST/CH<br>C/LT/CH<br>CC/LT/AD<br>CC/ST/AD	Piatek May<br>Piatek Nov<br>Klimov<br>Uyanikoglu<br>Bayrak<br>Ballestero 2021	CC/LT/AD<br>CC/LT/AD<br>CC/ST/CH<br>CC/ST/AD<br>CS/LT/CH<br>CS/ST/AD	NA	NA
	Continuous	Bulut	CC/ST/CH	Efe	CC/ST/CH	NA	NA

		McGrogan<br>Piatek May<br>Piatek Nov<br>Gonzalez<br>Keaveny<br>Klimov<br>Margoni<br>Mazure<br>Uyanikoglu<br>Corazza 95<br>Kavak<br>Szymczak<br>Volkan	C/ ST/CH<br>CC/LT/AD<br>CC/LT/AD<br>CS/LT/AD<br>C/LT/AD<br>CC/ST/CH<br>C/LT/CH<br>CC/LT/AD<br>CC/ST/AD<br>CS ST/AD<br>CC/ST/CH<br>CC/ST/AD<br>CC/ST/CH	El Amrousy<br>Piatek May<br>Piatek Nov<br>Klimov<br>Uyanikoglu<br>Bayrak<br>Ballestero<br>2021<br>Ballestero<br>2019<br>Bjorck<br>Corazza 95<br>Isikay<br>Pazianas<br>Szymczak<br>Volkan	CS/ST/CH<br>CC/LT/AD<br>CC/LT/AD<br>CC/ST/CH<br>CC/ST/AD<br>CS/LT/CH<br>CS/ST/AD<br>CS/ST/CH<br>CC/LT/CH<br>CS/ST/AD<br>CS/ST/CH<br>CC/LT/AD<br>CC/ST/AD<br>CC/ST/CH		
1-25-OH Vitamin D	Dichotomous	Ciacci	CC/LT/AD	NA	NA	NA	NA
	Continuous	Corazza 95<br>Szymczak<br>Uyanikoglu<br>Keaveny	CS ST/AD<br>CC/ST/AD<br>CC/ST/AD<br>C/LT/AD	Corazza 95<br>Szymczak<br>Uyanikoglu	CS ST/AD<br>CC/ST/AD<br>CC/ST/AD	NA	NA
Vitamin E	Dichotomous	Románczuk<br>Hozyasz<br>McGrogan<br>Selbuz	CC/ST/Both<br>C/ LT/CH<br>C/ ST/CH<br>CC/ST/CH	Románczuk	CC/ST/Both	NA	NA
	Continuous	Románczuk<br>Hozyasz<br>McGrogan<br>Piatek May	CC/ST/Both<br>C/ LT/CH<br>C/ ST/CH<br>CC/LT/AD	Románczuk<br>Piatek May	CC/ST/Both<br>CC/LT/AD	NA	NA
Vitamin K	Dichotomous	McGrogan	C/ ST/CH	NA	NA	NA	NA
	Continuous	McGrogan<br>Volkan	C/ ST/CH<br>CC/ST/CH	Volkan	CC/ST/CH	NA	NA
Calcium	Dichotomous	Ciacci<br>Margoni<br>Mazure<br>Kavak<br>Gonzalez<br>Choudhary<br>Selbuz	CC/LT/AD<br>C/LT/CH<br>CC/LT/AD<br>CC/ST/CH<br>CS/LT/AD<br>CS/ST/CH<br>CC/ST/CH	Szaflarska-<br>Poplawska	C/ST/CH	NA	NA
	Continuous	Bulut<br>Ciacci<br>Margoni<br>Mazure<br>Kavak<br>Gonzalez<br>Choudhary<br>Corazza 95<br>Szymczak<br>Kalayci<br>Karkoszka<br>Keaveny<br>Sabel'nikova<br>Volken<br>Kemppanien	CC/ST/CH<br>CC/LT/AD<br>C/LT/CH<br>CC/LT/AD<br>CC/ST/CH<br>CS/LT/AD<br>CS/ST/CH<br>CS ST/AD<br>CC/ST/AD<br>CC/LT/CH<br>C/LT/CH<br>C/LT/AD<br>C/ST/AD<br>CC/ST/CH<br>C/ST/AD	Ballestero 21<br>Ballestero19<br>Corazza 95<br>Kalayci<br>Pazianas<br>Szaflarska-<br>Poplawska<br>Szymczak<br>Volkan	CS/ST/AD<br>CS/ST/CH<br>CS/ST/AD<br>CC/LT/CH<br>CC/LT/AD<br>C/ST/CH<br><br>CC/ST/AD<br>CC/ST/CH	NA	NA
Copper	Dichotomous	McGrogan	C/ ST/CH	NA	NA	NA	NA

	Continuous	McGrogan	C/ ST/CH	NA	NA	NA	NA
Ferritin	Dichotomous	McGrogan<br>Kemppanien	C/ ST/CH<br>C/ST/AD	Bayrak<br>Mansueto	CS/LT/CH<br>CC/ST/AD	Mansueto	CC/ST/AD
	Continuous	McGrogan<br>Kemppanien<br>Piatek May<br>Volkan	C/ ST/CH<br>C/ST/AD<br>CC/LT/AD<br>CC/ST/CH	Bayrak<br>Elli<br>Efe<br>El Amrousy<br>Isikay<br>Piatek May<br>Volkan<br>Mansueto	CS/LT/CH<br>CC/LT/AD<br>CC/ST/CH<br>CS/ST/CH<br>CS/ST/CH<br>CC/LT/AD<br>CC/ST/CH<br>CC/ST/AD	Mansueto	CC/ST/AD
Folate	Dichotomous	McGrogan<br>Selbuz	C/ ST/CH<br>CC/ST/CH	Bayrak<br>Szaflarska-Poplawska<br>Mansueto	CS/LT/CH<br>C/ST/CH<br>CC/ST/AD	Mansueto	CC/ST/AD
	Continuous	McGrogan<br>Dickey<br>Volkan<br>Kemppanien (RBCs)	C/ ST/CH<br>CC/ST/AD<br>CC/ST/CH<br>C/ST/AD	Ballestero 2021<br>Ballestero 2019<br>Isikay<br>Volkan<br>Valente<br>Bayrak<br>Dickey<br>Szaflarska-Poplawska<br>Mansueto	CS/ST/AD<br>CS/ST/CH<br>CS/ST/CH<br>CC/ST/CH<br>CS/ST/AD<br>CS/LT/CH<br>CC/ST/AD<br>C/ST/CH<br>CC/ST/AD	Mansueto	CC/ST/AD
Iron	Dichotomous	Kemppanien<br>Anand<br>Selbuz	C/ST/AD<br>CC/ST/AD<br>CC/ST/CH	Bayrak<br>Elli (No Control)<br>Mansueto	CS/LT/CH<br>CC/LT/AD<br>CC/ST/AD	Mansueto	CC/ST/AD
	Continuous	Kemppanien<br>Anand<br>Sabel'nikova	C/ST/AD<br>CC/ST/AD<br>C/ST/AD	Ballestero 2021<br>Ballestero 2019<br>Bayrak<br>Elli<br>Efe<br>Mansueto	CS/ST/AD<br>CS/ST/CH<br>CS/LT/CH<br>CC/LT/AD<br>CC/ST/CH<br>CC/ST/AD	Mansueto	CC/ST/AD
Magnesium	Dichotomous	McGrogan<br>Selbuz	C/ ST/CH<br>CC/ST/CH	Szaflarska-Poplawska	C/ST/CH	NA	NA
	Continuous	McGrogan<br>Kemppanien<br>Volkan	C/ ST/CH<br>C/ST/AD<br>CC/ST/CH	Volkan<br>Szaflarska-Poplawska	CC/ST/CH<br>C/ST/CH	NA	NA
Selenium	Dichotomous	McGrogan	C/ ST/CH	Unal	CC/ST/CH	NA	NA
	Continuous	McGrogan<br>Kalita<br>Cortigiani<br>Boda	C/ ST/CH<br>C/ST/CH<br>CC/ST/CH<br>CC/LT/CH	Kalita<br>Cortigiani<br>Boda<br>Unal	C/ST/CH<br>CC/ST/CH<br>CC/LT/CH<br>CC/ST/CH	NA	NA
Zinc	Dichotomous	McGrogan<br>Selbuz<br>Högberg	C/ ST/CH<br>CC/ST/CH<br>C/ST/CH	NA	NA	NA	NA
	Continuous	McGrogan<br>Corazza 88<br>Högberg	C/ ST/CH<br>CC/ST/AD<br>C/ST/CH	Corazza 88<br>Singhal	CC/ST/AD<br>CC/ST/CH	NA	NA

		Henker<br>Singhal	C/ST/CH<br>CC/ST/CH					

*- C=Cohort, CS= Cross-sectional, CC= case control, LT= long term GFD, ST= short-term GFD, CH-children, AD-Adults, Both=AD+CH, ?=unknown, NA=Not applicable

PRISMA 2020 CHECKLIST

Section and Topic	Item #	Checklist item	Location where item is reported
**TITLE**			
Title	1	Identify the report as a systematic review.	1
**ABSTRACT**			
Abstract	2	See the PRISMA 2020 for Abstracts checklist.	2
**INTRODUCTION**			
Rationale	3	Describe the rationale for the review in the context of existing knowledge.	4
Objectives	4	Provide an explicit statement of the objective(s) or question(s) the review addresses.	4,5
**METHODS**			
Eligibility criteria	5	Specify the inclusion and exclusion criteria for the review and how studies were grouped for the syntheses.	4
Information sources	6	Specify all databases, registers, websites, organisations, reference lists and other sources searched or consulted to identify studies. Specify the date when each source was last searched or consulted.	SUP.1
Search strategy	7	Present the full search strategies for all databases, registers and websites, including any filters and limits used.	SUP 2
Selection process	8	Specify the methods used to decide whether a study met the inclusion criteria of the review, including how many reviewers screened each record and each report retrieved, whether they worked independently, and if applicable, details of automation tools used in the process.	4,5,6
Data collection process	9	Specify the methods used to collect data from reports, including how many reviewers collected data from each report, whether they worked independently, any processes for obtaining or confirming data from study investigators, and if applicable, details of automation tools used in the process.	5,6
Data items	10a	List and define all outcomes for which data were sought. Specify whether all results that were compatible with each outcome domain in each study were sought (e.g. for all measures, time points, analyses), and if not, the methods used to decide which results to collect.	5,6
	10b	List and define all other variables for which data were sought (e.g. participant and intervention characteristics, funding sources). Describe any assumptions made about any missing or unclear information.	5,6
Study risk of bias assessment	11	Specify the methods used to assess risk of bias in the included studies, including details of the tool(s) used, how many reviewers assessed each study and whether they worked independently, and if applicable, details of automation tools used in the process.	6
Effect measures	12	Specify for each outcome the effect measure(s) (e.g. risk ratio, mean difference) used in the synthesis or presentation of results.	5,6
Synthesis methods	13a	Describe the processes used to decide which studies were eligible for each synthesis (e.g. tabulating the study intervention characteristics and comparing against the planned groups for each synthesis (item #5)).	6
	13b	Describe any methods required to prepare the data for presentation or synthesis, such as handling of missing summary statistics, or data conversions.	6
	13c	Describe any methods used to tabulate or visually display results of individual studies and syntheses.	6

Section and Topic	Item #	Checklist item	Location where item is reported
	13d	Describe any methods used to synthesize results and provide a rationale for the choice(s). If meta-analysis was performed, describe the model(s), method(s) to identify the presence and extent of statistical heterogeneity, and software package(s) used.	4,5,6
	13e	Describe any methods used to explore possible causes of heterogeneity among study results (e.g. subgroup analysis, meta-regression).	6
	13f	Describe any sensitivity analyses conducted to assess robustness of the synthesized results.	6
Reporting bias assessment	14	Describe any methods used to assess risk of bias due to missing results in a synthesis (arising from reporting biases).	5,6
Certainty assessment	15	Describe any methods used to assess certainty (or confidence) in the body of evidence for an outcome.	6,7
**RESULTS**			
Study selection	16a	Describe the results of the search and selection process, from the number of records identified in the search to the number of studies included in the review, ideally using a flow diagram.	7,8<br>24-27<br>SUP Table 5,6
	16b	Cite studies that might appear to meet the inclusion criteria, but which were excluded, and explain why they were excluded.	SUP pg. 4-17
Study characteristics	17	Cite each included study and present its characteristics.	24-27
Risk of bias in studies	18	Present assessments of risk of bias for each included study.	27
Results of individual studies	19	For all outcomes, present, for each study: (a) summary statistics for each group (where appropriate) and (b) an effect estimate and its precision (e.g. confidence/credible interval), ideally using structured tables or plots.	7-14<br>32-35
Results of syntheses	20a	For each synthesis, briefly summarise the characteristics and risk of bias among contributing studies.	Sup P. 63-66
	20b	Present results of all statistical syntheses conducted. If meta-analysis was done, present for each the summary estimate and its precision (e.g. confidence/credible interval) and measures of statistical heterogeneity. If comparing groups, describe the direction of the effect.	7-14<br>32-35
	20c	Present results of all investigations of possible causes of heterogeneity among study results.	7-14
	20d	Present results of all sensitivity analyses conducted to assess the robustness of the synthesized results.	NA
Reporting biases	21	Present assessments of risk of bias due to missing results (arising from reporting biases) for each synthesis assessed.	32-35
Certainty of evidence	22	Present assessments of certainty (or confidence) in the body of evidence for each outcome assessed.	32-35

Section and Topic	Item #	Checklist item	Location where item is reported
**DISCUSSION**			
Discussion	23a	Provide a general interpretation of the results in the context of other evidence.	14-17
	23b	Discuss any limitations of the evidence included in the review.	16,17
	23c	Discuss any limitations of the review processes used.	16
	23d	Discuss implications of the results for practice, policy, and future research.	17
**OTHER INFORMATION**			
Registration and protocol	24a	Provide registration information for the review, including register name and registration number, or state that the review was not registered.	2, 5
	24b	Indicate where the review protocol can be accessed, or state that a protocol was not prepared.	5
	24c	Describe and explain any amendments to information provided at registration or in the protocol.	NA
Support	25	Describe sources of financial or non-financial support for the review, and the role of the funders or sponsors in the review.	22,23
Competing interests	26	Declare any competing interests of review authors.	22,23
Availability of data, code and other materials	27	Report which of the following are publicly available and where they can be found: template data collection forms; data extracted from included studies; data used for all analyses; analytic code; any other materials used in the review.	23

From: Page MJ, McKenzie JE, Bossuyt PM, Boutron I, Hoffmann TC, Mulrow CD, et al. The PRISMA 2020 statement: an updated guideline for reporting systematic reviews. BMJ 2021;372:n71. doi: 10.1136/bmj.n71

For more information, visit: http://www.prisma-statement.org/
